# A C–H bond activation-based catalytic approach to tetrasubstituted chiral allenes

**DOI:** 10.1038/ncomms8946

**Published:** 2015-08-06

**Authors:** Shangze Wu, Xin Huang, Wangteng Wu, Pengbin Li, Chunling Fu, Shengming Ma

**Affiliations:** 1 Laboratory of Molecular Recognition and Synthesis, Department of Chemistry, Zhejiang University, Hangzhou, Zhejiang 310027, China

## Abstract

Enantioselective synthesis of fully substituted allenes has been a challenge due to the non-rigid nature of the axial chirality, which spreads over three carbon atoms. Here we show the commercially available simple Rh complex may catalyse the CMD (concerted metalation/deprotonation)-based reaction of the readily available arenes with sterically congested tertiary propargylic carbonates at ambient temperature affording fully substituted allenes. It is confirmed that the excellent designed regioselectivity for the C–C triple bond insertion is induced by the coordination of the carbonyl group in the directing carbonate group as well as the steric effect of the tertiary O-linked carbon atom. When an optically active carbonate was used, surprisingly high efficiency of chirality transfer was realized, affording fully substituted allenes in excellent enantiomeric excess (ee).

## Abstract (web summary)

The enantioselective synthesis of tetrasubstituted allenes is a challenging task. Here, the authors report a rhodium-catalysed process for the coupling of alkynes with arenes, allowing access to fully substituted allenes—including enantioenriched products.

## Graphical abstract

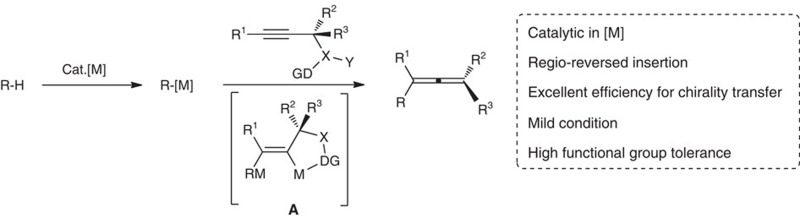

Allenes have been becoming more and more important due to their presence in nature and the recent demonstration of their great potentials in modern organic chemistry with very nice reactivities, which led to their versatile applications in the efficient total syntheses of some natural products and drugs^1^,^2^,^3^,^4^. Thus, the highly selective synthesis of allenes is of great importance^5^,^6^,^7^,^8^,^9^,^10^. On the basis of recent advancements, the enantioselective synthesis of 1,3-disubstituted or 1,1,3-trisubstituted allenes has been at least partially solved^6^, however, enantioselective synthesis of tetrasubstituted is still a challenge^6^,^7^,^8^,^9^,^10^. Here we propose that the well-established CMD (concerted metalation/deprotonation) process of arenes^11^,^12^,^13^,^14^,^15^,^16^ would generate organometallic intermediates *in situ*, which would, in principle, react with 2-alkynylic derivatives with an appropriate leaving group to afford allenes via regioselectivity-reversed insertion and thermodynamically non-favoured β-elimination (Fig. 1b). However, the challenge is the regioselectivity of the carbometalation of the C–C triple bond with the *in situ* generated aryl metallic species^17^,^18^,^19^,^20^,^21^,^22^ since the reported insertion reaction with the C–C triple bond in propargylic alcohols affording the [4+2]-products with the undesired regioselectivity^18^. To achieve our goal, we proposed to reverse the regioselectivity by installing a directing entity in the propargylic leaving group, thus avoiding the formation of undesired **B**-type insertion intermediate^18^. In addition, it should be noted that stereospecific β-elimination of the targeted intermediate **A** providing highly optically active allenes is challenging as the Rh-catalysed reaction of stoichiometric amount each of aryl boronic acids and optically active propargylic acetates with forming allenes in an extremely low efficiency of chirality transfer due to the mixed syn- and anti-non-stereospecific β-elimination^23^,^24^,^25^. In such a well-designed process, the oxidation state of the metal in the catalyst would remain the same, thus, making it catalytic in [M] and free of any oxidants for the regeneration of catalyst in many of C–H functionalization reactions^11^,^12^,^13^,^14^,^15^,^16^.

Herein, we wish to report the realization of such a concept for the synthesis of tetrasubstituted allenes via the commercially available [Cp*RhCl_2_]_2_ catalysed coupling between readily available essential chemicals, that is, arenes and 2-alkynylic carbonates with a surprisingly high efficiency for point-to-axial chirality transfer. After careful studies, it is believed that the directing group and the steric effect of the tertiary 2-alkynylic carbonates are responsible for the reversed regioselectivity for the insertion of the C–C triple bond.

## Results

### Identification of the leaving group

**Figure 2:**
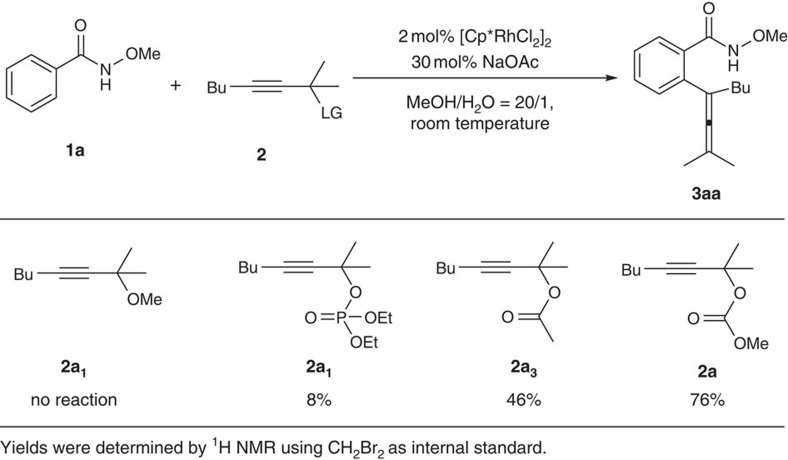
The effect of leaving groups. A carbonate was found to be the most efficient.

With the purpose of synthesizing the most challenging tetra-subtituted allenes, we first tried to identify a proper leaving group for the tertiary propargylic alcohol (Fig. 2): When *N*-methoxybenzamide **1a** was reacted with tertiary propargylic methyl ether **2a**_**1**_ in a mixed solvent of MeOH and water (20/1) under the catalysis of [Cp*RhCl_2_]_2_ at room temperature, no allene was formed with **1a** being recovered; when we introduced a phosphate as leaving group (**2a**_**2**_), interestingly the expected tetrasubstituted allene **3aa** was afforded in 8% NMR yield!^18^ When propargylic acetate **2a**_**3**_ was used, the yield was improved to 46%; with these results in hand, we envisioned to have a methoxy group to replace the methyl group in the acetyl unit to increase the electron density of the carbonyl oxygen, that is, carbonate: to our delight, the carbonate **2a** was indeed the best and the yield was further improved to 76%. After further optimization of the parameters of the reaction, the reaction of 1.2 equiv. **1a** and **2a** in a mixed solvent of MeOH and water (20/1) under the catalysis of [Cp*RhCl_2_]_2_ with 30 mol% NaOAc as base was chosen as the optimized reaction conditions for further study (for details of optimization, see Supplementary Table 1).

### Substrate scope

**Figure 3:**
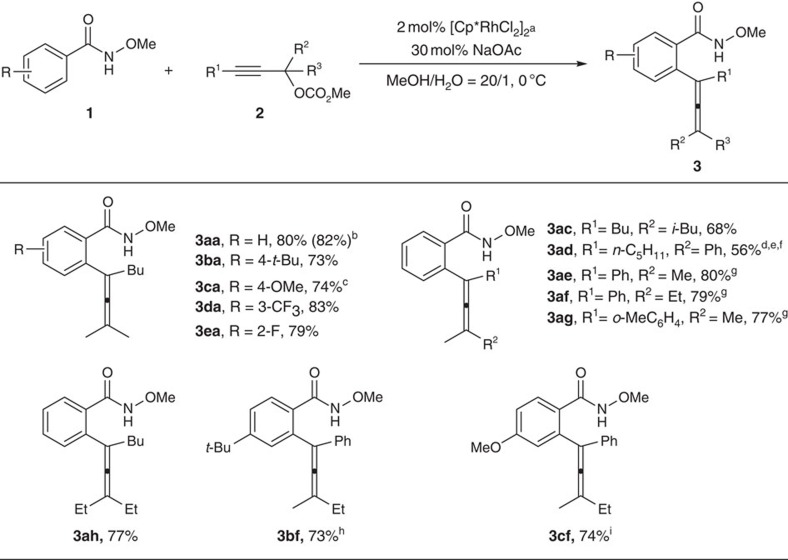
Substrate scope. Electron-withdrawing and electron-donating as well as bulky groups were all tolerated. ^a^The reaction was conducted with **1** (1.2 mmol), **2** (1 mmol), [Cp*RhCl_2_]_2_ (0.02 mmol), NaOAc (0.3 mmol), MeOH (6 ml), and H_2_O (0.3 ml) and monitored by TLC. ^b^Reaction was conducted on 7 mmol scale. ^c^Reaction was conducted at −10 °C, **1a** (1.3 mmol) and NaOAc (1 mmol) was used. ^d^Reaction was conducted at room temperature. ^e^**1a** (2 mmol), [Cp*RhCl_2_]_2_ (0.05 mmol) was used. ^f^The acetate was used instead because of the unstability of the carbonate. ^g^**1a** (1.5 mmol), [Cp*RhCl_2_]_2_ (0.04 mmol) was used. ^h^**1b** (1.5 mmol), [Cp*RhCl_2_]_2_ (0.04 mmol) was used. ^i^**1c** (1.5 mmol), [Cp*RhCl_2_]_2_ (0.04 mmol) was used.

The reaction proceeded well on a gram scale, affording **3aa** in 82% yield (Fig. 3). Having the optimized reaction conditions in hand, the generality of the reaction was then investigated. Electron-donating groups such as *t*-butyl and methoxy as well as electron-withdrawing groups such as CF_3_ and F in the aryl unit were all tolerated in the amides giving the corresponding fully substituted allenes in good to excellent yields (Fig. 3; **3aa**, **3ba**, **3ca**, **3da**, **3ea**, **3bf** and **3cf**). It was noteworthy that when a meta-substituted **1d** was used, the less hindered C–H bond was exclusively functionalized and an *o*-fluorine atom did not hamper the reaction affording **3ea** in 79% yield (Fig. 3, **3da** and **3ea**). The R^1^, R^2^, and R^3^ groups in the 2-alkynylic carbonate could be alkyl or aryl groups (Fig. 3; **3ac**, **3ad**, **3ae**, **3af**, **3ag**, **3ah**, **3bf** and **3cf**). Substrates with sterically hindered *i*-Bu or *o*-tolyl groups also worked (Fig. 3, **3ac** and **3ag**).

### Compatibility of functional groups

**Figure 4:**
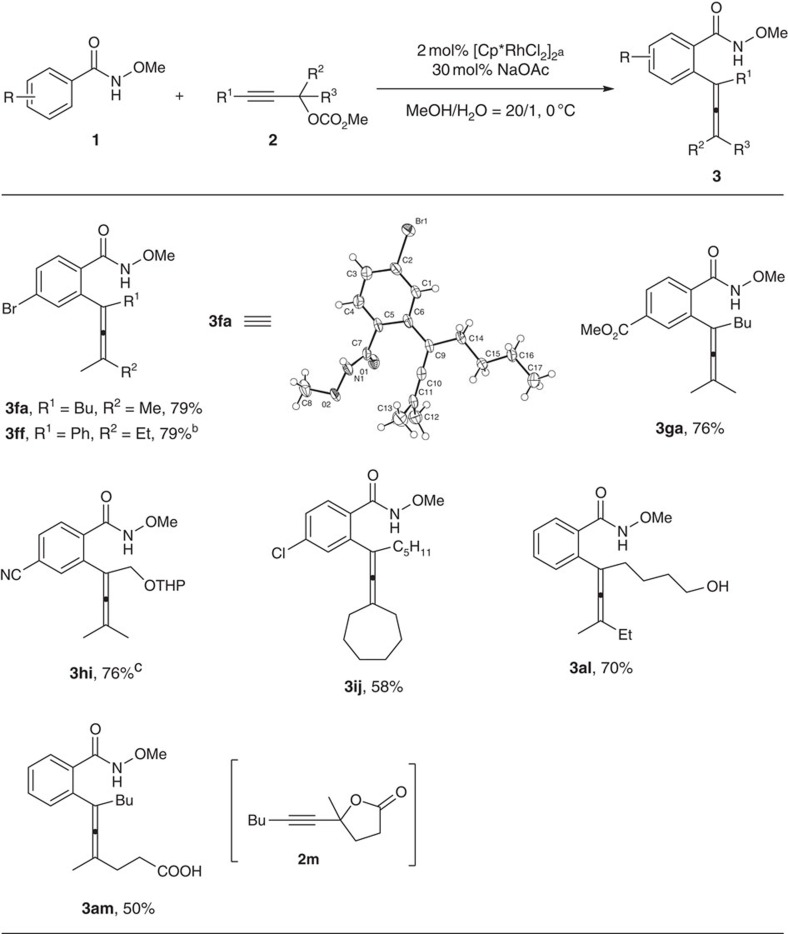
Functional group compatibility. Many synthetically useful yet sensitive functional groups survived without any protection. ^a^The reaction was conducted with **1** (1.2 mmol), **2** (1 mmol), [Cp*RhCl_2_]_2_ (0.02 mmol), NaOAc (0.3 mmol), MeOH (6 ml) and H_2_O (0.3 ml) and monitored by TLC. ^b^**1f** (1.5 mmol), [Cp*RhCl_2_]_2_ (0.04 mmol) was used. ^c^ [Cp*RhCl_2_]_2_ (0.04 mmol) was used.

To show the robustness of our newly developed catalytic allene synthesis strategy, substrates with synthetically useful yet sensitive functional groups were explored without any protection: to our delight, Cl, Br, CO_2_Me, CN and even unprotected free alcohol were all well tolerated, showing the broad synthetically attractive functional group compatibility (Fig. 4; **3fa**, **3ff**, **3ga**, **3hi**, **3ij** and **3al**). The structure of **3fa** was further confirmed by X-ray diffraction study. (For details of the X-ray diffraction study of **3fa**, see Supplementary Dataset 1 and pages 96–97 in the Supplementary File.) Interestingly, even *γ*-(1-alkynyl)-*γ*-lactone **2m** may be used to afford tetrasubstituted *γ*-allenoic acid **3am** directly in an atom-economic way (Fig. 4; **3am**).

### Regioselectivity

**Figure 5:**
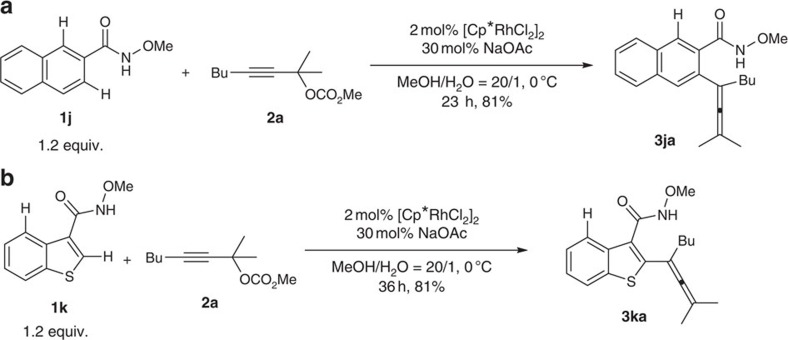
Regioselectivity of the reaction. (**a**) The less hindered C–H bond was exclusively functionalized. (**b**) The thiophene C–H bond was exclusively functionalized.

The regioselectivity is an important issue when >1 C–H bonds could be activated. When *N*-methoxy-2-naphthylcarboxylic amide **1j** was used to react with **2a**, the less hindered C–H bond was exclusively functionalized affording **3ja** (Fig. 5, equation (a)). A heteroaryl *N*-methoxyamide **1k** was also a suitable substrate and C–H bond in the thiene moiety was exclusively functionalized giving **3ka** in 81% yield (Fig. 5, equation (b); also see: Fig. 3; **3da**).

### Other directing groups

**Figure 6:**
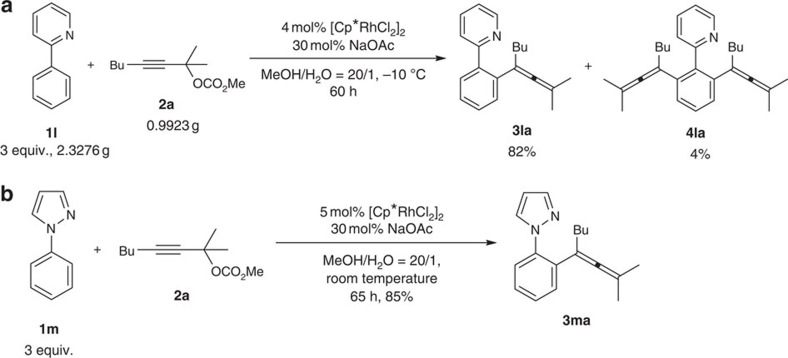
The scope of directing groups. Pyridine and pyrazole could also be suitable directing groups.

With this protocol in hand, we reasoned that such a concept should be working also with directing groups other than the *N*-methoxy amide. In fact, arenes **1l** and **1m** with directing groups of pyridine and pyrazole could also react with **2a** forming tetrasubstituted allenes **3la** and **3ma**. Thus, such a protocol may be applied to other type of substrates with functionalizable C–H bonds (Fig. 6).

### Synthetic applications

**Figure 7:**
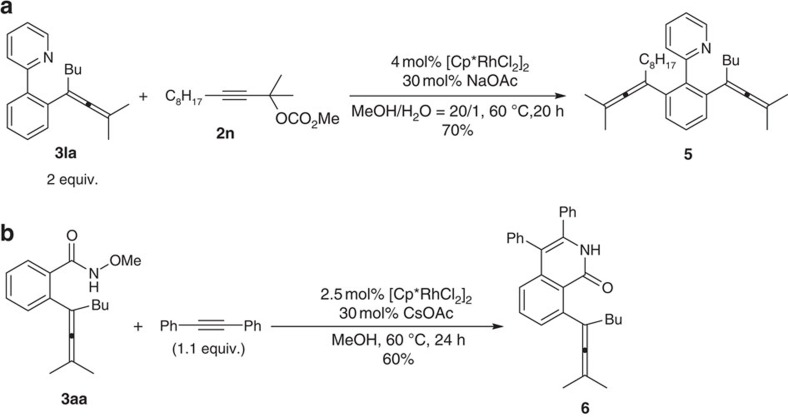
The synthetic applications. (**a**) A stepwise allenylation. (**b**) A [4+2] addition affording isoquinolinone.

The synthetic applications for the formed products were also conducted: **3la** could react with an extra propargyl carbonate, affording bis-allene **5**, which showed the possibility of a stepwise allenylation (Fig. 7, equation (a)). The *N*-methoxyamide moiety in **3aa** could direct a well-known [4+2] addition with 1,2-diphenylethyne giving isoquinolinone **6** with allene intact (Fig. 7, equation (b))^18^.

## Discussion

**Figure 8:**
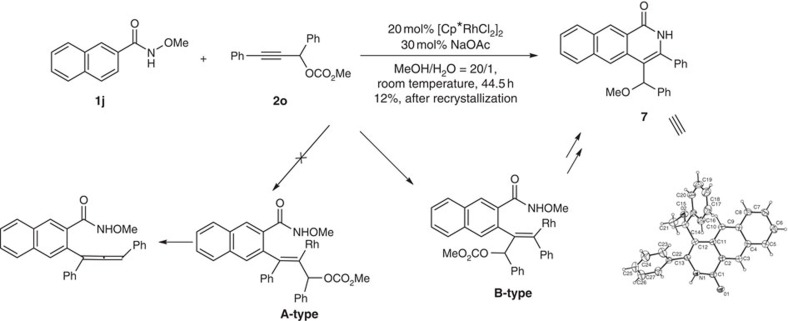
Striking tertiary carbon atom effect. Secondary propargylic carbonate **2o** only demonstrated the opposite regioselectivity.

In order to further study the mechanism, following experiments have been conducted. Firstly, *N*-methoxy-2-naphthamide **1j** was treated with 20 mol% of [Cp*RhCl_2_]_2_ to *in situ* generate the naphthyl Rh species, its reaction with secondary propargylic carbonate **2o** demonstrated the opposite regioselectivity, leading to the undesired reported **B**-type insertion regioisomer exclusively^18^, finally yielding the [4+2] product **7** observed by Fagnou, Glorius, Rovis, Cramer and other groups (Fig. 8; for details of the X-ray diffraction study of **7**, see Supplementary Dataset 2 and page 108 in the Supplementary File)^17^,^18^,^19^,^20^,^21^,^22^: the desired **A**-type insertion regioisomer, which would have led to the formation of allene via β-elimination, was not formed! Thus, we reasoned that there is a striking tertiary carbon atom effect here: in order to have the carbonyl group acts the directing group in this linear C–C triple bond environment, the tertiary carbon atom in the carbonate may push the carbonyl group for its easier coordination with Rh while having a weaker interaction with the to-be-inserted C–C triple bond.

**Figure 9:**
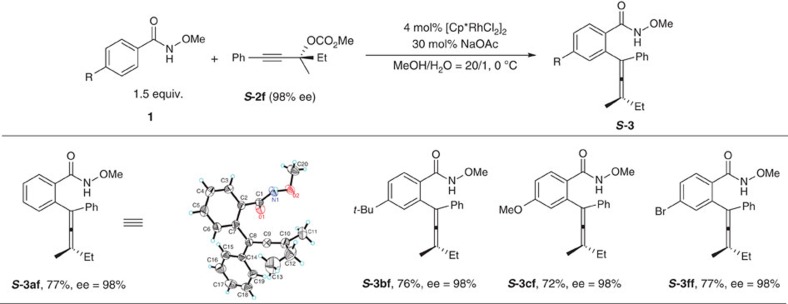
Highly entioselective synthesis of tetra-substituted allenes by chirality transfer. Excellent efficiency of chirality transfer was found when using chiral carbonate ***S*****-2f**. The reaction was conducted with **1** (0.3 mmol), **2** (0.2 mmol), [Cp*RhCl_2_]_2_ (0.008 mmol), NaOAc (0.06 mmol), MeOH (1.2 ml), and H_2_O (0.06 ml) and monitored by TLC. The ees were determined by HPLC.

In the second place, to our delight, when chiral carbonate ***S*****-2f** (98% ee) was applied, axial chiral allenes were formed with an excellent ee (98% ee) with a surprisingly high efficiency of chirality transfer (Fig. 9). The absolute configuration of chiral **3af** was determined as *S* by X-ray diffraction study and the absolute configurations of other chiral allenes ***S*****-3bf**, ***S*****-3cf** and ***S*****-3ff** were defined by analogy. (For details of the X-ray diffraction study of ***S*****-3af**, see Supplementary Dataset 3 and page 110 in the Supplementary File.) These results further excluded the possibility of oxidative addition of propargylic carbonate with Rh to form allenyl rhodium intermediate (leading to racemization)^24^,^26^,^27^, which would react with arene via a CMD process followed by reductive elimination to afford the allene.

**Figure 10:**
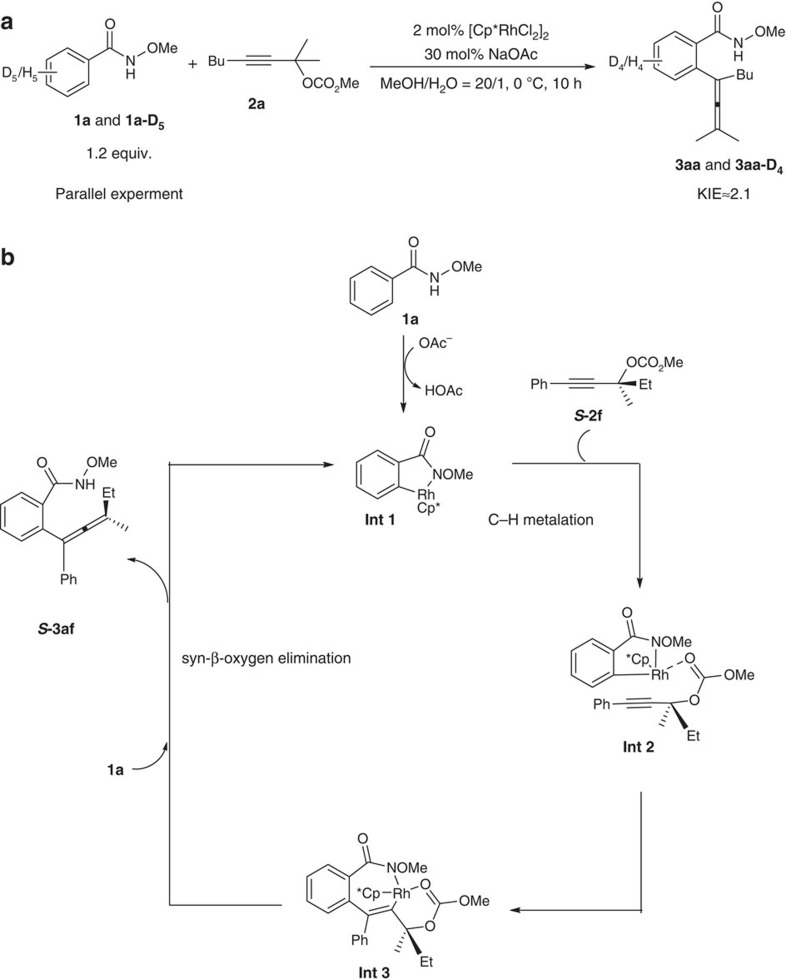
Mechanistic issues (**a**) The kinetic isotope effect. A kinetic isotope effect value of 2.1 was measured. (**b**) Plausible mechanism. The carbonate carbonyl and tertiary carbon atom effect accounts for the reversed regioselectivity and the excellent efficiency of chirality was realized via syn-β-elimination.

The kinetic isotope effect was measured by parallel experiments of **1a** and **1a-D**_**5**_ with **2a**. The kinetic isotope effect value of 2.1 demonstrated that C–H activation should be the rate-determining step (Fig. 10a)^28^.

According to these results, a plausible mechanism has been proposed as shown in Fig. 10b. The first step of the rate-determining CMD of arenes would form rhodabicyclic intermediate **Int 1** (ref. 29), which is followed by coordination with the carbonyl oxygen of the carbonate forming **Int 2**. Subsequent ‘reversed' regiospecific insertion of the alkyne moiety^18^ directed by the carbonyl group in propargyl carbonate ***S*****-2f** afforded with the carbonyl oxygen-coordinated rhoda-tricyclic **Int 3** (refs 11, 12, 13, 14). The steric effect may set the carbonyl group for its better coordination with Rh and the subsequent defined coordination/insertion with C–C triple bond leading to the formation of **Int 3-**type tricyclic intermediate, which directly underwent syn-β-oxygen elimination to form the final product ***S*****-3af** and relieves the catalytically active Rh(III) species^30^,^31^,^32^,^33^,^34^,^35^,^36^,^37^. It should be noted that usually anti-β-oxygen elimination was observed^23^,^24^,^25^. This syn-β-oxygen elimination explains the excellent efficiency for chirality transfer since it was reported that the Rh-catalysed reaction of optically active propargylic acetates with aryl boronic acids forming allenes in an extremely low efficiency of chirality transfer^23^,^24^,^25^.

In conclusion, a completely new protocol has been established for the efficient synthesis of allenes using the readily available arenes and 2-alkynylic carbonates as the starting materials at room temperature with very good yields. The reaction is compatible with ambient air, moisture and a broad array of synthetically useful functional groups such as Cl, Br, CO_2_Me, CN, free alcohol and even acid. The formed tetrasubstituted allenes could be transformed to isoquinolinone as while as bis-allene products. In fact, in some cases, very minor amount of cycloisomerisation products were observed in the NMR analysis of the crude products. Excellent selectivity for alkyne insertion must be induced by combination of carbonyl directing group as well as the steric effect of tertiary carbon centre. The surprisingly high efficiency of chirality transfer addresses the challenge for the synthesis of not-readily available highly optically active fully substituted allenes from a highly optically active carbonate. This protocol for the synthesis of allenes will trigger such reactions using other types of substrates with functionalizable C–H bonds and propargylic alcohol derivatives, thus, will be of high interest in organic chemistry and related disciplines. Further studies in this area are being pursued in our laboratory.

## Methods

### Materials

[Cp*RhCl_2_]_2_ was purchased from Strem Chemicals. *N*-methoxybenzamides^22^ and known propargylic carbonates^38^ were prepared according to the literature procedures. Other commercially available chemicals were purchased and used without additional purification unless noted otherwise.

### General spectroscopic methods

^1^H NMR spectra were recorded on a Bruker-300 MHz spectrometer and ^13^C NMR spectra were recorded at 75 MHz. All ^1^H NMR experiments were measured with tetramethylsilane (0 p.p.m.) or the signal of residual CHCl_3_ (7.26 p.p.m.) in CDCl_3_ as the internal reference, ^13^C NMR experiments were measured in relative to the signal of CDCl_3_ (77.0 p.p.m.), and ^19^F NMR experiments were measured in relative to the signal of residual CFCl_3_ (0 p.p.m.) in CDCl_3_. Infrared spectra were recorded from this films of pure samples on sodium chloride plates for liquid or in the form of KBr discs for the solid samples. Mass and high-resolution mass spectrometry (HRMS) spectra were carried out in Electron Ionization (EI) mode. Thin-layer chromatography (TLC) was performed on pre-coated glass-back plates and visualized with ultraviolet light at 254 nm. Flash column chromatography was performed on silica gel. ^1^H NMR, ^13^C NMR and High Performance Liquid Chromatography (HPLC) spectra (for chiral compounds) are supplied for all compounds: see Supplementary Figs 1–82. See Supplementary Methods for the characterization data of compounds not listed in this part.

### Synthesis of **3aa**

To a dried Schlenk tube equipped with a Teflon-coated magnetic stirring bar were added *N*-methoxybenzamide **1a** (182.1 mg, 1.2 mmol), [Cp*RhCl_2_]_2_ (12.6 mg, 0.02 mmol), NaOAc (24.3 mg, 0.3 mmol), methyl (2-methyloct-3-yn-2-yl) carbonate **2a** (198.5 mg, 1 mmol), MeOH (6 ml), and H_2_O (0.3 ml) sequentially at room temperature. After being stirred for 19 h at 0 °C, the reaction was complete as monitored by TLC. Filtration through a short column of silica gel (eluent: ethyl acetate 20 ml × 3) and evaporation afforded the crude product, which was purified by flash column chromatography on silica gel (eluent: petroleum/ethyl acetate/dichloromethane=10/1/0.1/ to 5/1/0.5) to afford **3aa** (218.6 mg, 80%): solid; melting point (m.p.) 66.9–68.1 °C (hexane/ethyl acetate); ^1^H NMR (300 MHz, CDCl_3_) *δ* 8.74 (brs, 1 H, NH), 7.58 (d, *J*=7.2 Hz, 1 H, Ar–H), 7.39 (td, *J*_*1*_=7.5 Hz, *J*_*2*_=1.3 Hz, 1 H, Ar–H), 7.32–7.21 (m, 2 H, Ar–H), 3.86 (s, 3 H, OCH_3_), 2.29 (t, *J*=7.1 Hz, 2 H, CH_2_), 1.76 (s, 6 H, 2 × CH_3_), 1.49–1.27 (m, 4 H, 2 × CH_2_), 0.89 (t, *J*=6.9 Hz, 3 H, CH_3_); ^13^C NMR (75 MHz, CDCl_3_) *δ* 201.3, 167.9, 138.3, 131.5, 130.5, 129.1, 126.7, 102.9, 97.6, 64.3, 33.6, 30.0, 22.1, 20.3, 13.9; IR (neat, cm^−1^) 3,188, 2,956, 2,931, 2,875, 2,859, 1,953, 1,659, 1,590, 1,495, 1,465, 1,440, 1,299, 1,156, 1,035; MS (EI, 70 eV) *m*/*z* (%) 273 (M^+^, 4.18), 242 (100); Anal. Calcd for C_17_H_23_NO_2_: C 74.69, H 8.48, N 5.12. Found: C 74.89, H 8.62, N 4.89.

### Synthesis of **5**

To a dried Schlenk tube equipped with a Teflon-coated magnetic stirring bar were added [Cp*RhCl_2_]_2_ (12.5 mg, 0.02 mmol), NaOAc (12.8 mg, 0.15 mmol), **3la** (277.5 mg, 1.0 mmol), **2n** (126.8 mg, 0.5 mmol), MeOH (3 ml), and H_2_O (0.15 ml) sequentially at room temperature. The Schlenk tube was then equipped with a condenser. After being stirred for 20 h at 60 °C, the reaction was complete as monitored by TLC (eluent: petroleum ether/ethyl acetate=20/1). Filtration through a short column of silica gel (eluent: ethyl acetate 20 ml × 3) and evaporation afforded the crude product, which was purified by flash column chromatography on silica gel (eluent: hexane/ethyl acetate=100/1) to afford **5** (158.4 mg, 70%): oil; ^1^H NMR (300 MHz, CDCl_3_) *δ* 8.61 (d, *J*=4.2 Hz, 1 H, Ar–H), 7.62 (td, *J*_*1*_=7.8 Hz, *J*_*2*_=1.8 Hz, 1 H, Ar–H), 7.30–7.22 (m, 2 H, Ar–H), 7.21–7.11 (m, 3 H, Ar–H), 2.01–1.90 (m, 4 H, CH_2_ × 2), 1.39 (s, 12 H, CH_3_ × 4), 1.32–1.10 (m, 16 H, CH_2_ × 8), 0.87 (t, *J*=6.8 Hz, 3 H, CH_3_), 0.80 (t, *J*=7.1 Hz, 3 H, CH_3_); ^13^C NMR (75 MHz, CDCl_3_) *δ* 201.2, 160.0, 148.6, 139.92, 139.89, 138.1, 134.9, 127.5, 127.4, 125.9, 121.1, 103.3, 103.2, 95.6, 34.2, 33.9, 31.8, 29.8, 29.5, 29.3, 29.0, 27.6, 22.6, 22.1, 20.4, 14.1, 14.0; IR (neat, cm^−1^) 3,059, 2,955, 2,926, 2,854, 1,962, 1,932, 1,588, 1,571, 1,561, 1,452, 1,419, 1,377, 1,361, 1,188, 1,023; MS (EI, 70 eV) *m*/*z* (%) 455 (M^+^, 18.96), 84 (100); HRMS Calcd for C_33_H_45_N (M^+^): 455.3552. Found: 455.3553.

### Synthesis of **6**

To a dried Schlenk tube equipped with a Teflon-coated magnetic stirring bar were added **3aa** (109.3 mg, 0.4 mmol), 1,2-diphenylethyne (78.6 mg, 0.44 mmol), [Cp*RhCl_2_]_2_ (6.2 mg, 0.01 mmol), CsOAc (22.4 mg, 0.12 mmol), and MeOH (2 ml) sequentially at room temperature. The Schlenk tube was then equipped with a condenser. After being stirred for 24 h at 60 °C, the reaction was complete as monitored by TLC (eluent: petroleum ether/ethyl acetate=3/1). Filtration through a short column of silica gel (eluent: (dichloromethane/ethyl acetate=1/1) (20 ml × 3)) and evaporation afforded the crude product, which was purified by flash column chromatography on silica gel (eluent: dichloromethane/ethyl acetate=20/1) to afford **6** (101.1 mg, 60%): solid; m.p. 199.0–200.4 °C (hexane/ethyl acetate); ^1^H NMR (300 MHz, CDCl_3_) *δ* 9.02 (s, 1 H, Ar–H), 7.44 (t, *J*=7.8 Hz, 1 H, Ar–H), 7.34–7.10 (m, 12 H, Ar–H), 2.33 (t, *J*=7.1 Hz, 2 H, CH_2_), 1.76 (s, 6 H, CH_3_ × 2), 1.53–1.29 (m, 4 H, CH_2_ × 2), 0.90 (t, *J*=7.1 Hz, 3 H, CH_3_); ^13^C NMR (75 MHz, CDCl_3_) *δ* 199.0, 161.6, 142.7, 140.2, 137.2, 136.4, 135.1, 131.9, 131.7, 129.8, 129.0, 128.5, 128.3, 127.1, 124.9, 122.5, 116.8, 106.9, 95.6, 34.7, 30.5, 22.5, 20.9, 14.2; IR (neat, cm^−1^) 3,453, 3,165, 3,027, 2,947, 2,929, 2,869, 2,849, 1,947, 1,642, 1,596, 1,584, 1,486, 1,462, 1,441, 1,311, 1,144; MS (EI, 70 eV) *m*/*z* (%) 419 (M^+^, 31.3), 376 (100); HRMS Calcd for C_30_H_29_NO (M^+^): 419.2249. Found: 419.2247. Anal. Calcd for C_30_H_29_NO: C 85.88, H 6.97, N 3.34. Found: C 84.90, H 6.96, N 3.31.

### Synthesis of **7**

Following procedure for the synthesis of **3aa**, the reaction of **1j** (303.1 mg, 1.5 mmol), [Cp*RhCl_2_]_2_ (185.5 mg, 0.3 mmol), NaOAc (36.9 mg, 0.45 mmol), **2o** (400.1 mg, 1.5 mmol), MeOH (9 ml), and H_2_O (0.45 ml) at room temperature afforded impure **7** with impurities (136.8 mg) (eluent: Hexane/ethyl acetate/dichloromethane=10/1/0.2 to 8/1/0.5), which was further purified by recrystallization (hexane/THF) afford pure **7** (69.7 mg, 12%): solid; m.p. 117.5–119.0 °C (hexane/THF); ^1^H NMR (300 MHz, CDCl_3_) *δ* 9.02 (s, 1 H, Ar–H), 8.91 (bs, 1 H, NH), 8.32 (s, 1 H, Ar–H), 8.06–7.94 (m, 1 H, Ar–H), 7.75–7.66 (m, 1 H, Ar–H), 7.55–7.40 (m, 8 H, Ar–H), 7.36–7.17 (m, 4 H, Ar–H), 5.57 (s, 1 H, CH), 3.31 (s, 3 H, CH_3_); ^13^C NMR (75 MHz, CDCl_3_) *δ* 163.2, 141.6, 139.4, 135.1, 134.8, 131.2, 131.1, 129.6, 129.04, 128.97, 128.81, 128.77, 128.5, 128.3, 127.8, 127.1, 126.6, 126.2, 124.5, 111.1, 80.3, 56.5; IR (neat, cm^−1^) 3,181, 3,057, 3,027, 2,927, 2,890, 2,819, 1,659, 1,625, 1,600, 1,493, 1,448, 1,355, 1,312, 1,089, 1022; MS (EI, 70 eV) m/z (%) 392 (M^+^+1, 30.89), 391 (M^+^, 100); HRMS Calcd for C_27_H_21_NO_2_ (M^+^): 391.1572. Found: 391.1571.

### Synthesis of ***S*** **-3af**

Following procedure for the synthesis of **3aa**, the reaction of **1a** (45.6 mg, 0.3 mmol), [Cp*RhCl_2_]_2_ (4.8 mg, 0.008 mmol), NaOAc (5.3 mg, 0.06 mmol), ***S*****-2f** (98% ee, 45.9 mg, 0.2 mmol), MeOH (1.2 ml), and H_2_O (0.06 ml) at 0 °C afforded ***S*****-3af** (46.6 mg, 77%) (eluent: petroleum/ethyl acetate/dichloromethane=5/1/0.5): 98% ee (HPLC conditions: Chiralcel AD-H column, hexane/*i*-PrOH=10/1, 1.0 ml min^−1^, *λ*=207 nm, *t*_R(minor)_=21.9 min, *t*_R(major)_=24.5 min); solid; m.p. 99.9–101.1 °C (hexane/ethyl acetate); ^1^H NMR (300 MHz, CDCl_3_) δ 8.66 (s, 1 H, NH), 7.78 (d, *J*=7.5 Hz, 1 H, Ar–H), 7.53–7.14 (m, 8 H, Ar–H), 3.45 (s, 3 H, OCH_3_), 2.27–2.06 (m, 2 H, CH_2_), 1.90 (s, 3 H, CH_3_), 1.13 (t, *J*=7.4 Hz, 3 H, CH_3_); ^13^C NMR (75 MHz, CDCl_3_) δ 202.0, 166.6, 137.2, 135.6, 132.5, 131.20, 131.18, 129.6, 128.6, 127.9, 127.1, 126.5, 107.5, 106.5, 63.9, 27.4, 18.7, 12.3; IR (neat, cm^−1^) 3,199, 3,058, 3,018, 2,966, 2,932, 1,947, 1,659, 1,595, 1,491, 1,456, 1,439, 1,310, 1,159, 1,031; MS (EI, 70 eV) *m*/*z* (%) 307 (M^+^, 1.69), 246 (100); Anal. Calcd for C_20_H_21_NO_2_: C 78.15, H 6.89, N 4.56. Found: C 77.98, H 6.88, N 4.34.

## Additional information

**How to cite this article:** Shangze, W. *et al*. A C–H bond activation-based catalytic approach to tetrasubstituted chiral allenes. *Nat. Commun.* 6:7946 doi: 10.1038/ncomms8946 (2015).

## Supplementary Material

Supplementary Information Supplementary Figures 1-82, Supplementary Table, Supplementary Methods and Supplementary References

Supplementary Data 1 X-ray diffraction data for compound 3fa

Supplementary Data 2 X-ray diffraction data for compound 7

Supplementary Data 3 X-ray diffraction data for compound 3af

## Figures and Tables

**Figure 1:**
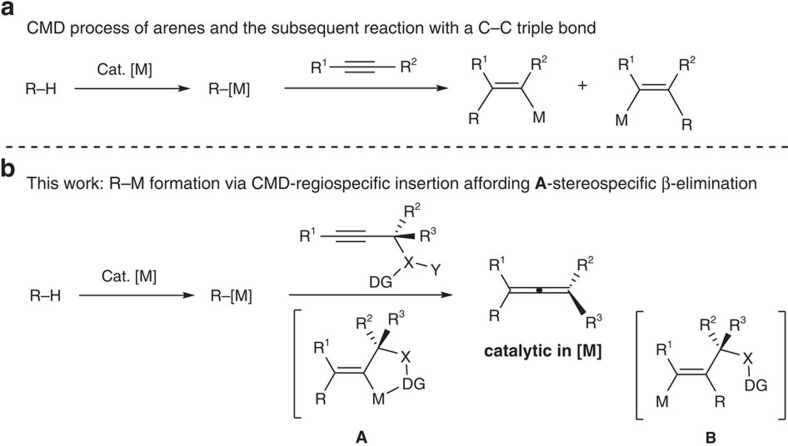
Carbometallations of alkynes. (**a**) CMD-based reaction of R–H with alkynes. (**b**) This work: new concept and challenge for allene synthesis.
