# Supplementary material for: A C–H bond activation-based catalytic approach to tetrasubstituted chiral allenes
Source: Nat Commun. 2015 Aug 6;6:7946. doi: 10.1038/ncomms8946 (PMC4918348; doi:10.1038/ncomms8946)
Supplement: Supplementary Information — Supplementary Figures 1-82, Supplementary Table, Supplementary Methods and Supplementary References [file ncomms8946-s1.pdf]

Supplementary Figures

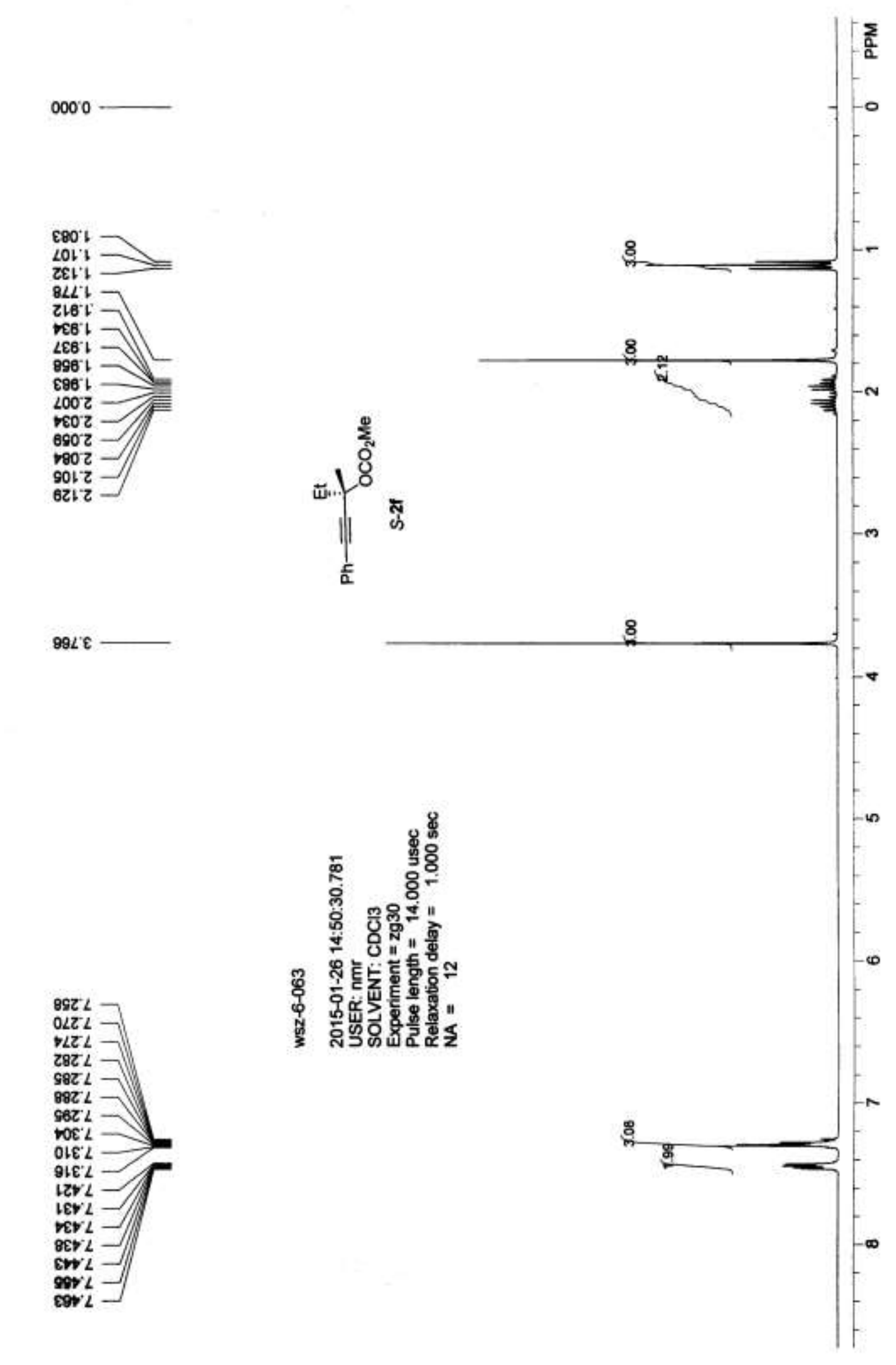

Supplementary Figure 1. ^{1}H NMR (300 MHz, $CDCl_3$) spectrum for *S*-2f.

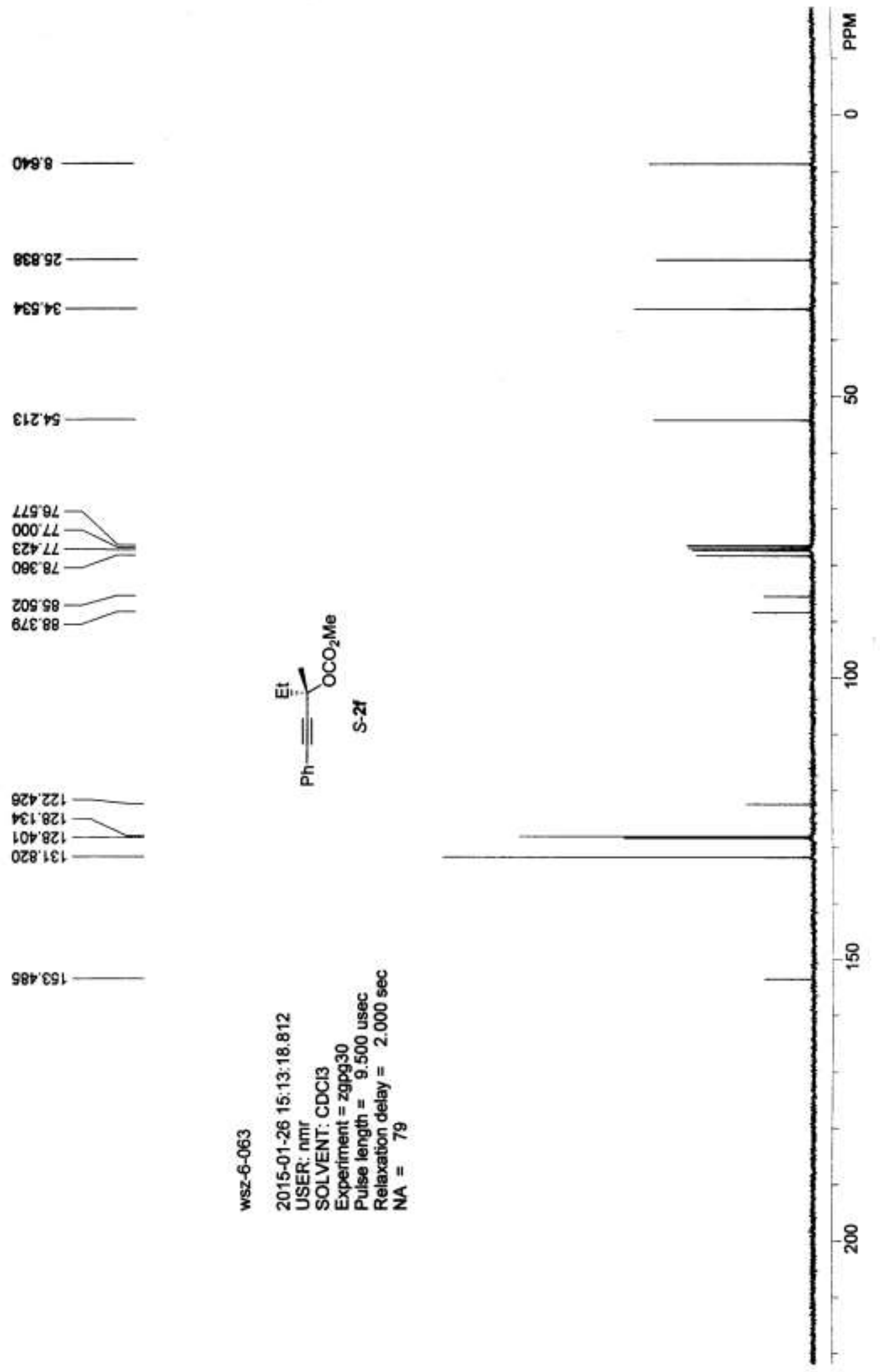

Supplementary Figure 2. ^{13}C NMR (75 MHz, $CDCl_3$) spectrum for *S*-2f.

N2000 数据工作站

wsz-6-63

实验单位：zju
实验时间：2015-01-25, 16:06:22
谱图文件:D:\浙大智达\N2000\样品\B0710.org

实验者：wsz
报告时间：2015-01-25, 16:47:07
积分方法:面积归一法

实验内容简介：
OD-H, n-hexane/iPrOH = 200/1, 207 nm, 0.8 ml/min

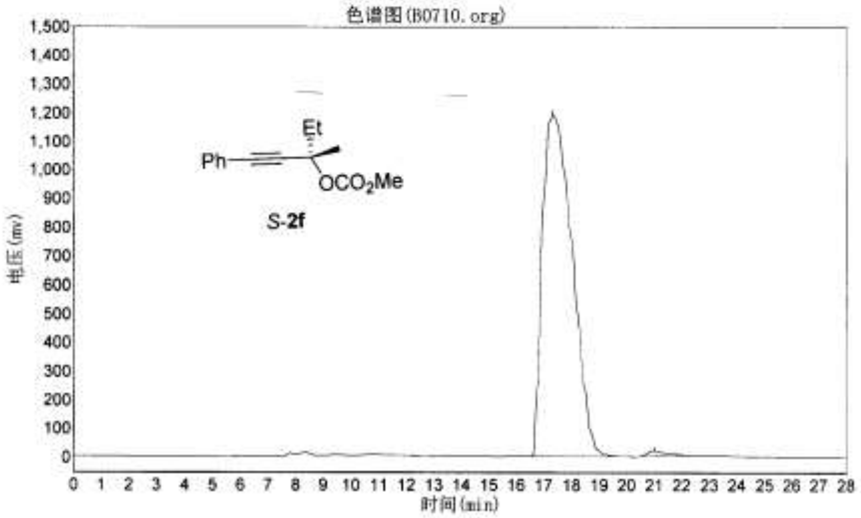

分析结果表

峰号	峰名	保留时间	峰高	峰面积	含量
1		17.347	1192142.750	89213056.000	98.9400
2		21.003	18933.926	955778.375	1.0600
总计			1211076.676	90168834.375	100.0000

2015-01-25 浙江大学智能信息研究所

Supplementary Figure 3. HPLC spectrum for *S*-2f.

N2000 数据工作站

wsz-5-164

实验单位：zju
实验时间：2015-01-25, 16:37:03
谱图文件:D:\浙大智达\N2000\样品\B0711.org

实验者：wsz
报告时间：2015-01-25, 17:22:50
积分方法:面积归一法

实验内容简介：
OD-H, n-hexane/iPrOH = 200/1, 207 nm, 0.8 ml/min

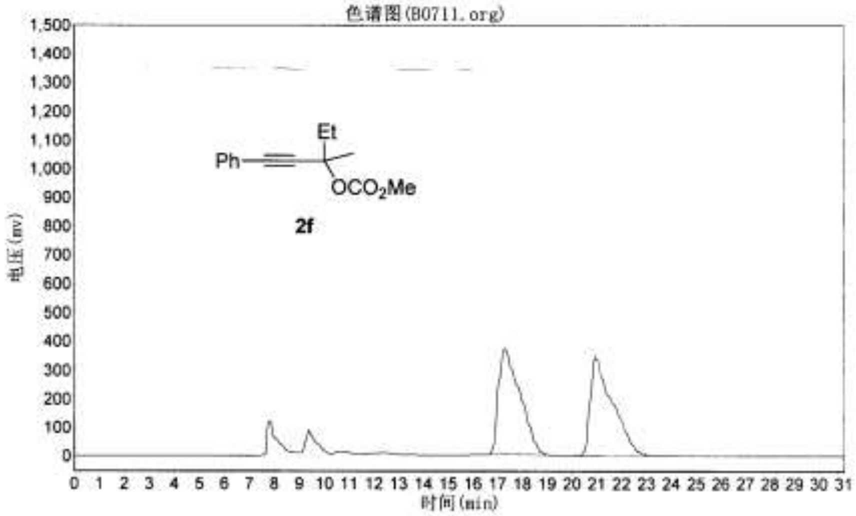

分析结果表

峰号	峰名	保留时间	峰高	峰面积	含量
1		17.272	363124.188	22242770.000	50.6509
2		20.950	339036.219	21671092.000	49.3491
总计			702160.406	43913862.000	100.0000

2015-01-25

浙江大学智能信息研究所

Supplementary Figure 4. HPLC spectrum for 2f.

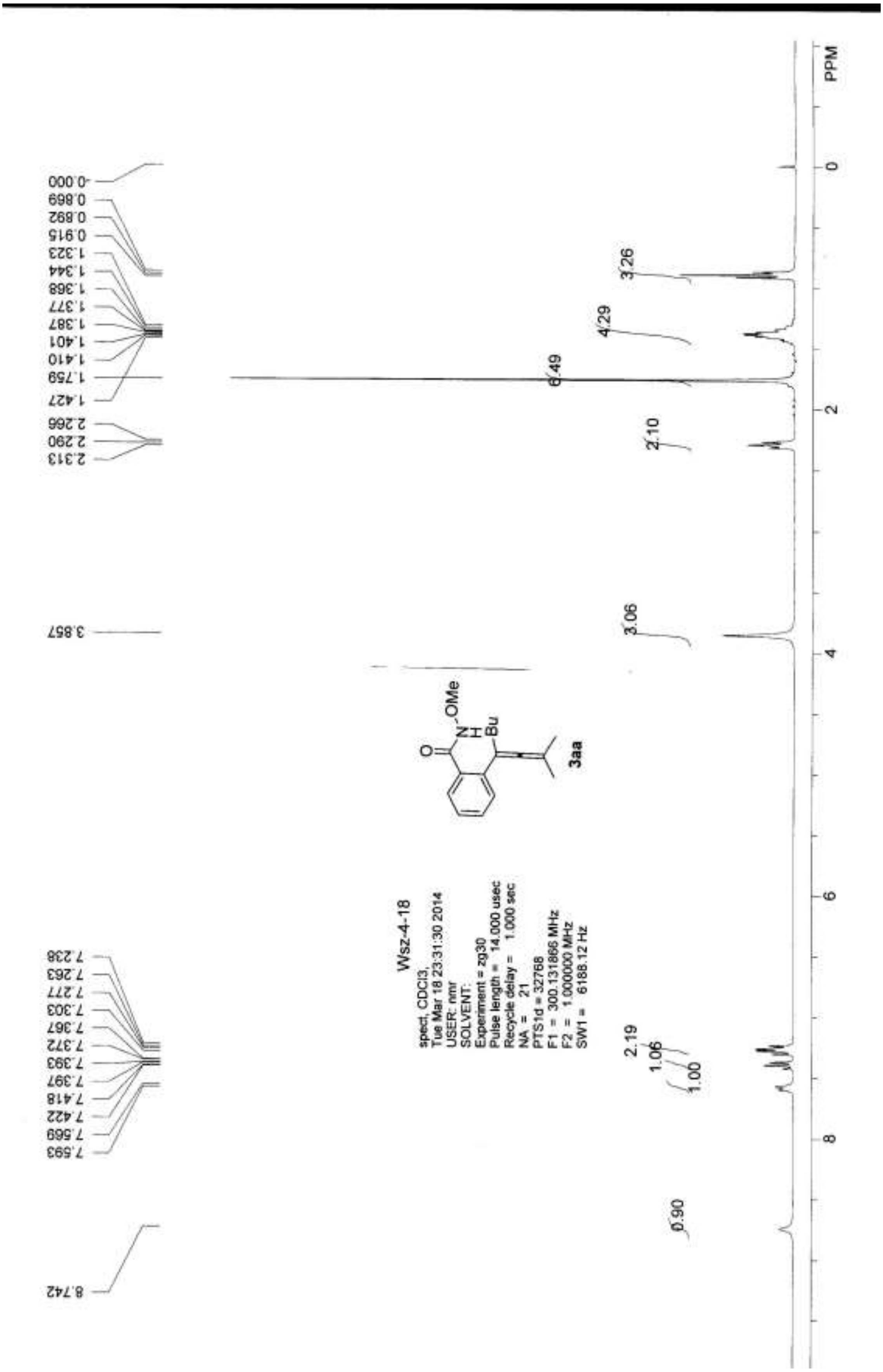

Supplementary Figure 5. ^{1}H NMR (300 MHz, $CDCl_3$) spectrum for 3aa.

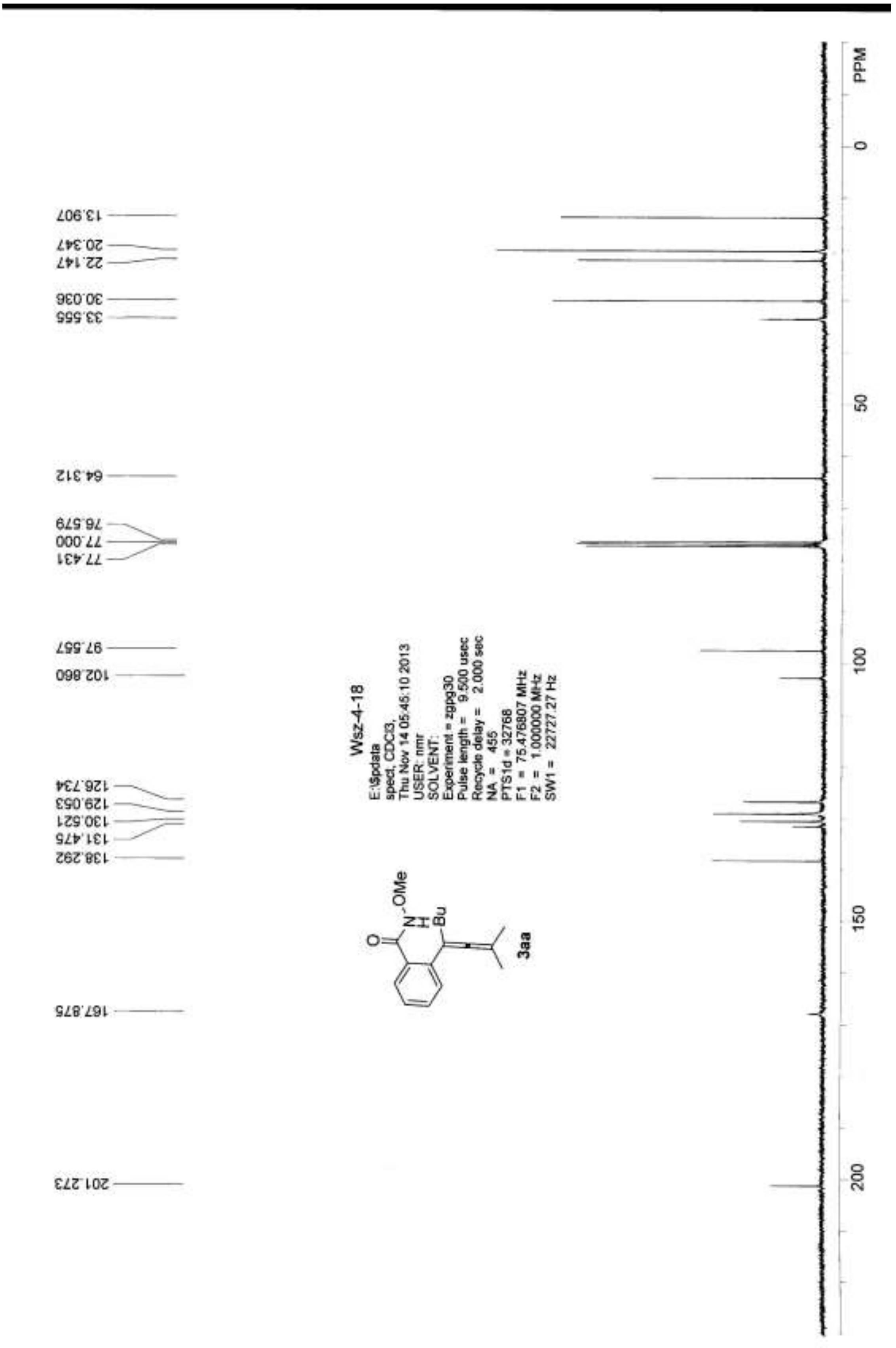

Supplementary Figure 6. ^{13}C NMR (75 MHz, $CDCl_3$) spectrum for 3aa.

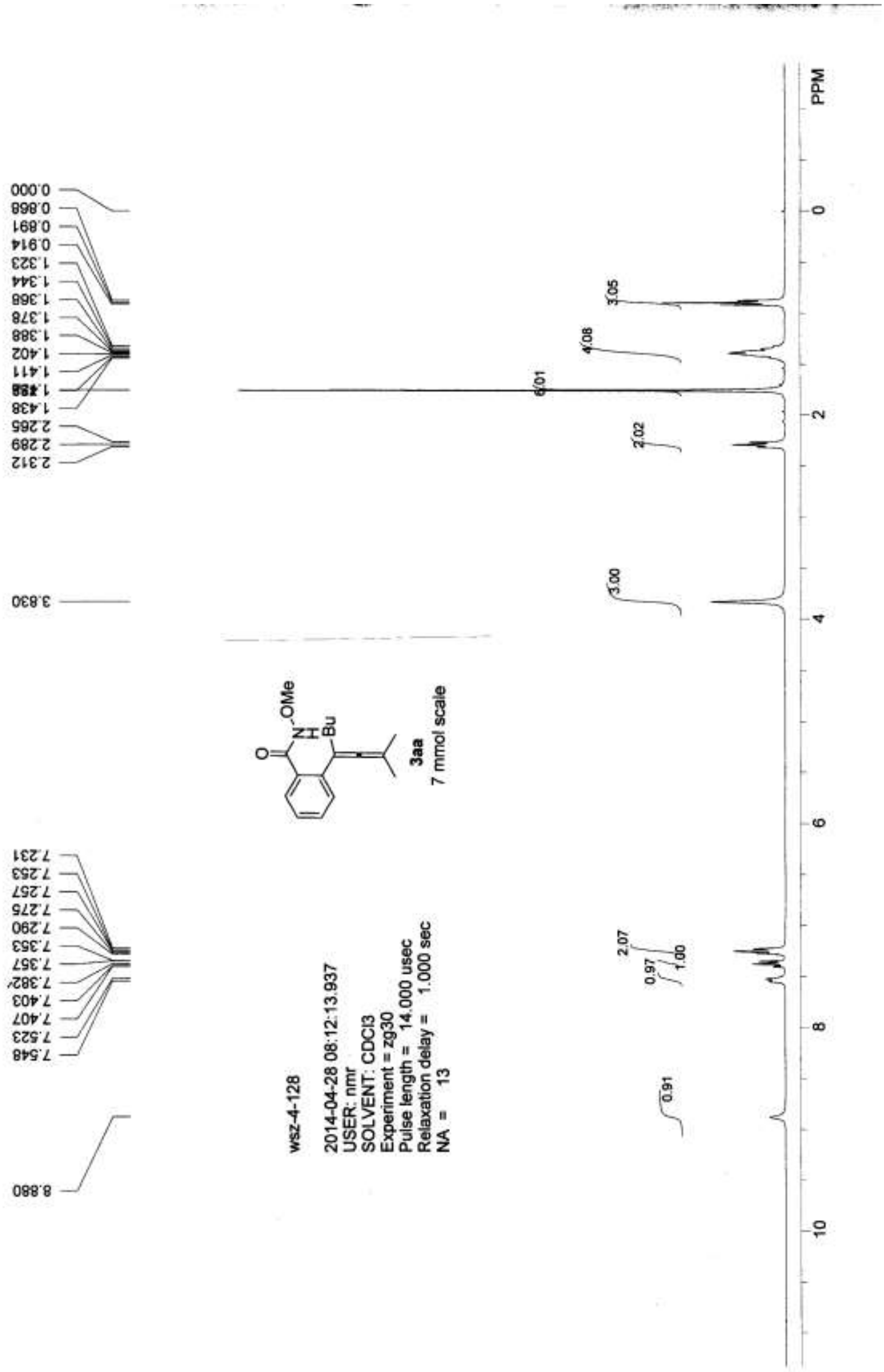

Supplementary Figure 7. ^{1}H NMR (300 MHz, $CDCl_3$) spectrum for 3aa (7 mmol scale).

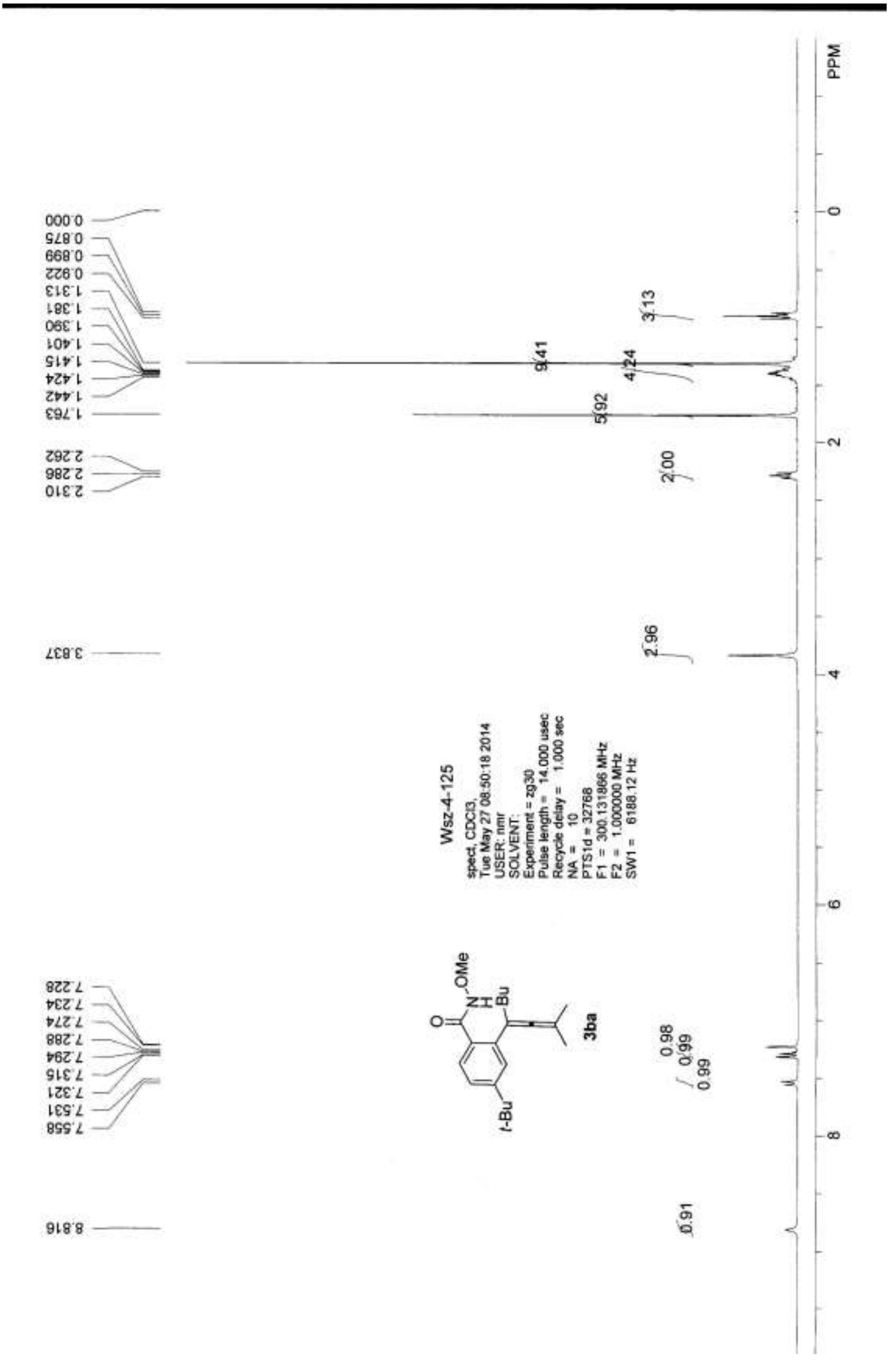

Supplementary Figure 8. ^{1}H NMR (300 MHz, $CDCl_3$) spectrum for 3ba.

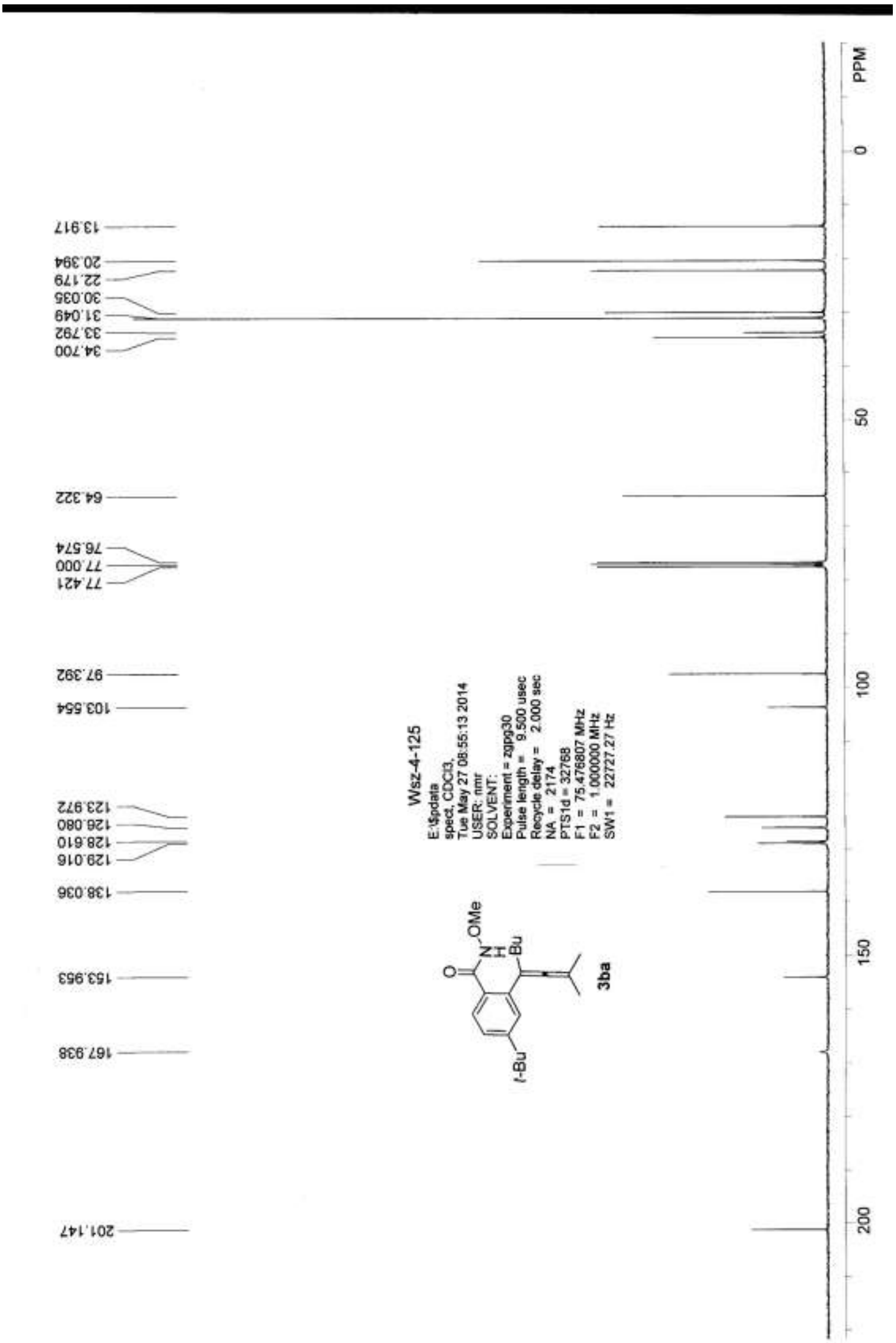

Supplementary Figure 9. ^{13}C NMR (75 MHz, $CDCl_3$) spectrum for 3ba.

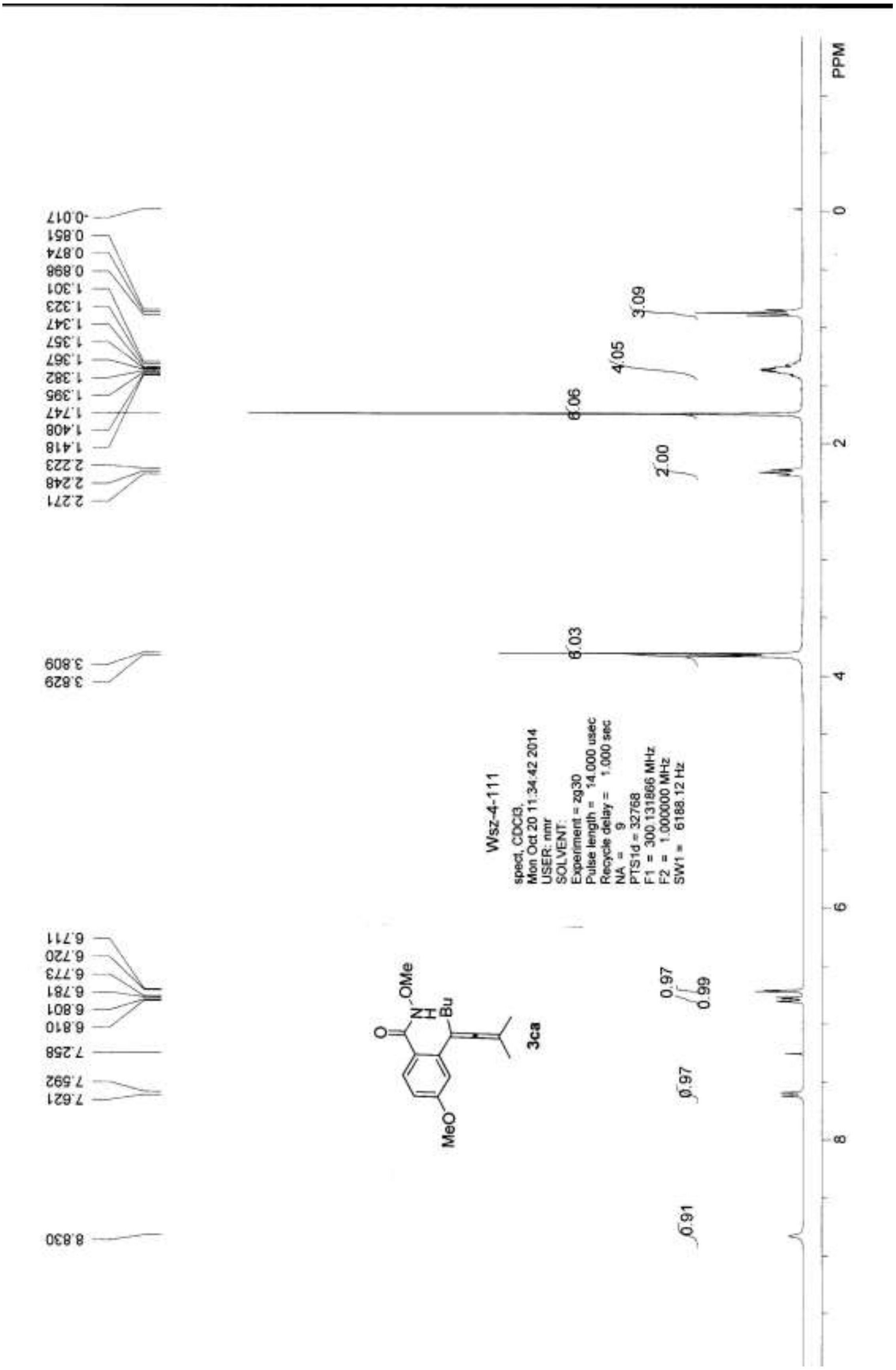

Supplementary Figure 10. ^{1}H NMR (300 MHz, $CDCl_3$) spectrum for 3ca.

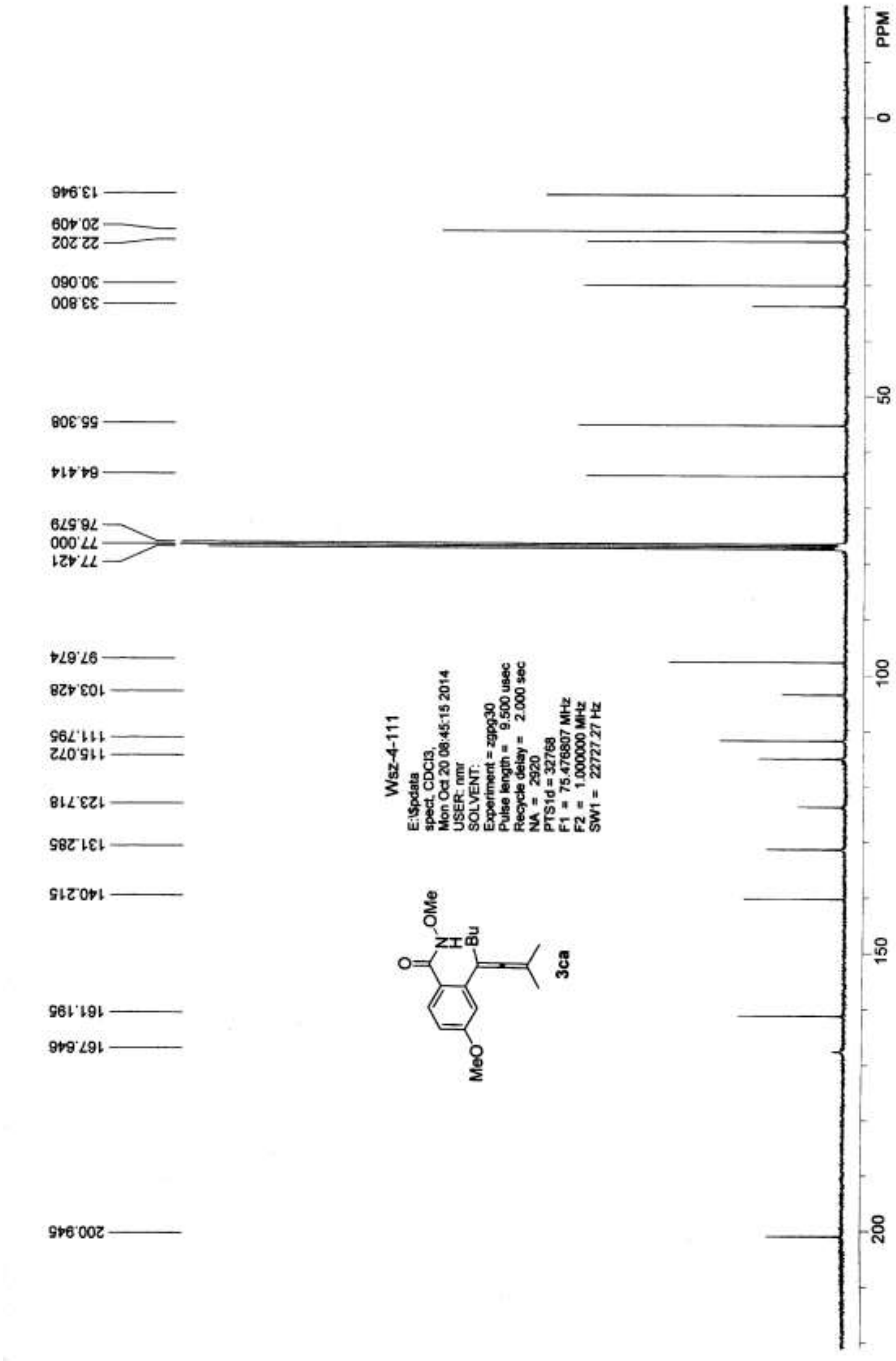

Supplementary Figure 11. ^{13}C NMR (75 MHz, $CDCl_3$) spectrum for 3ca.

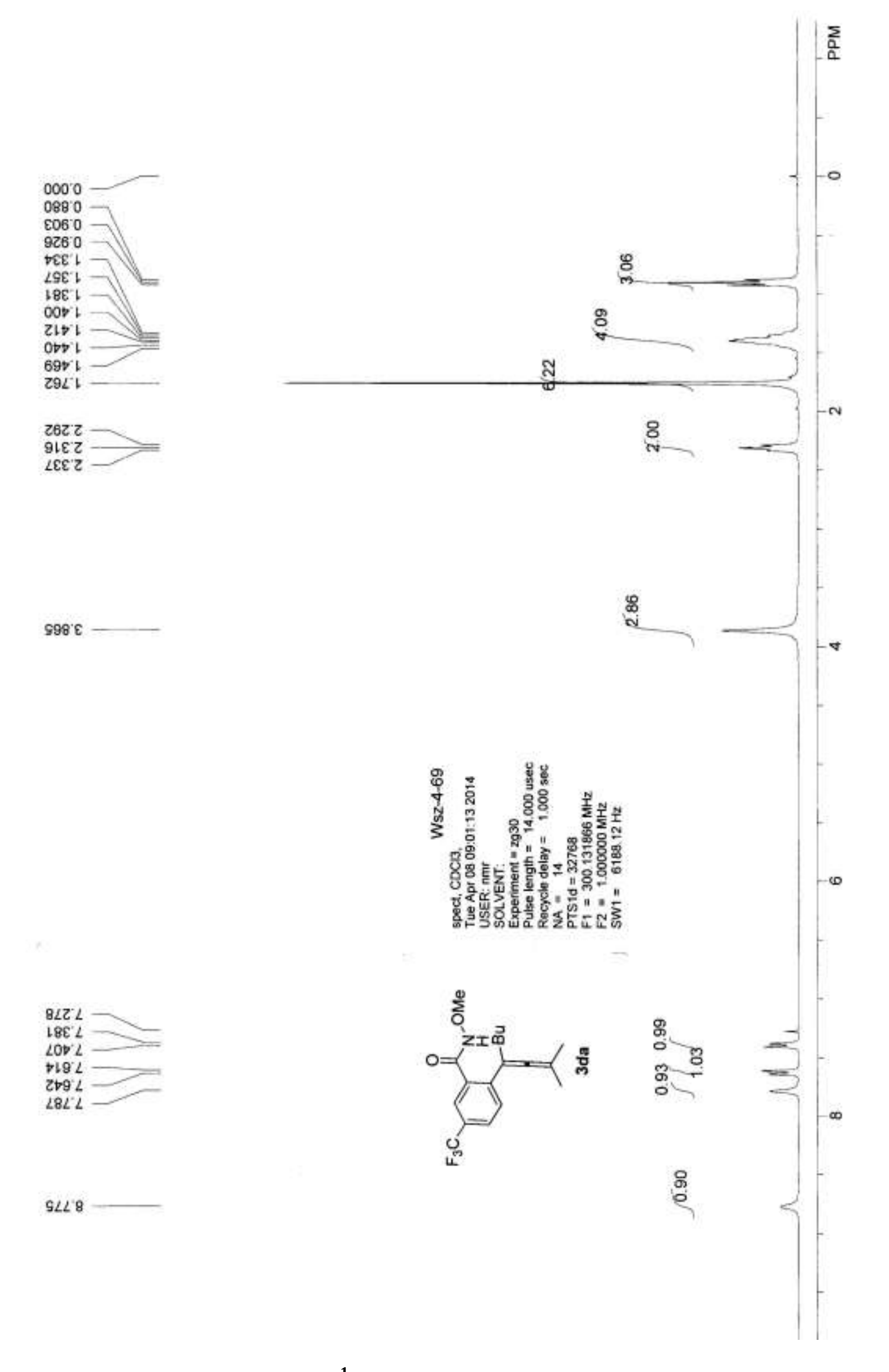

Supplementary Figure 12. ^{1}H NMR (300 MHz, $CDCl_3$) spectrum for 3da.

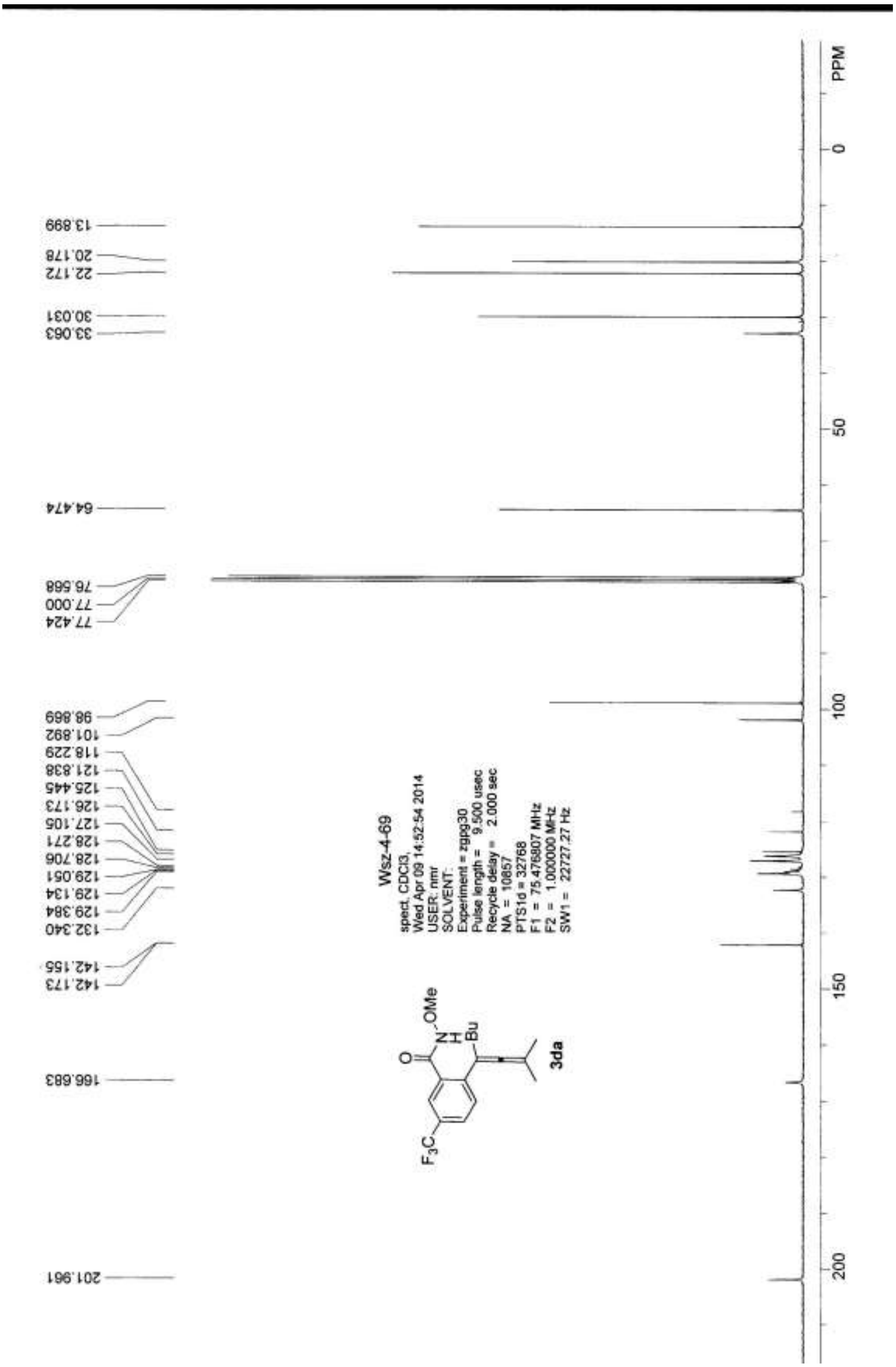

Supplementary Figure 13. ^{13}**C NMR (75 MHz,** $CDCl_3$**) spectrum for 3da.**

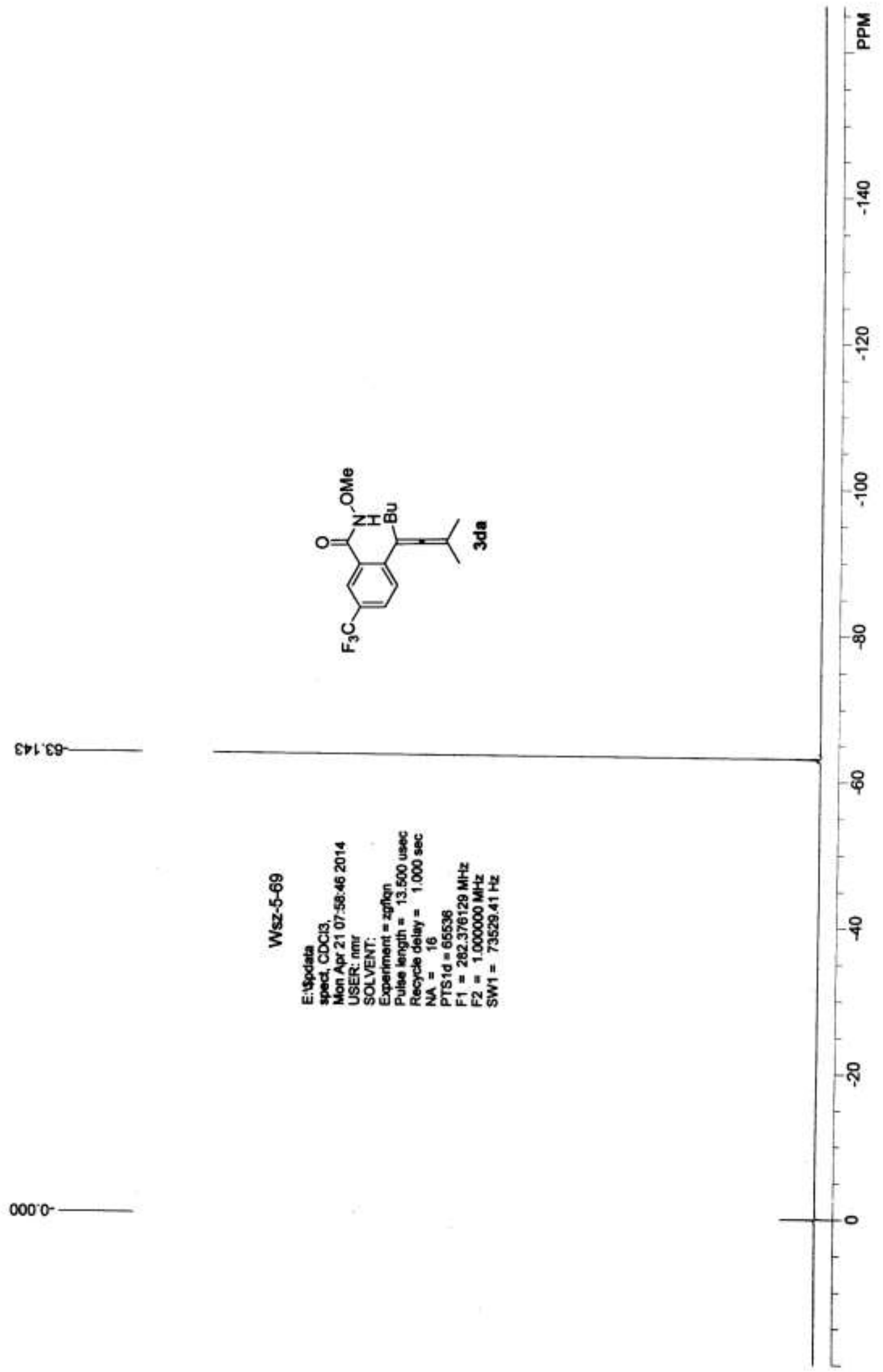

Supplementary Figure 14. ^{19}F **NMR (282 MHz,** $CDCl_3$**) spectrum for 3da.**

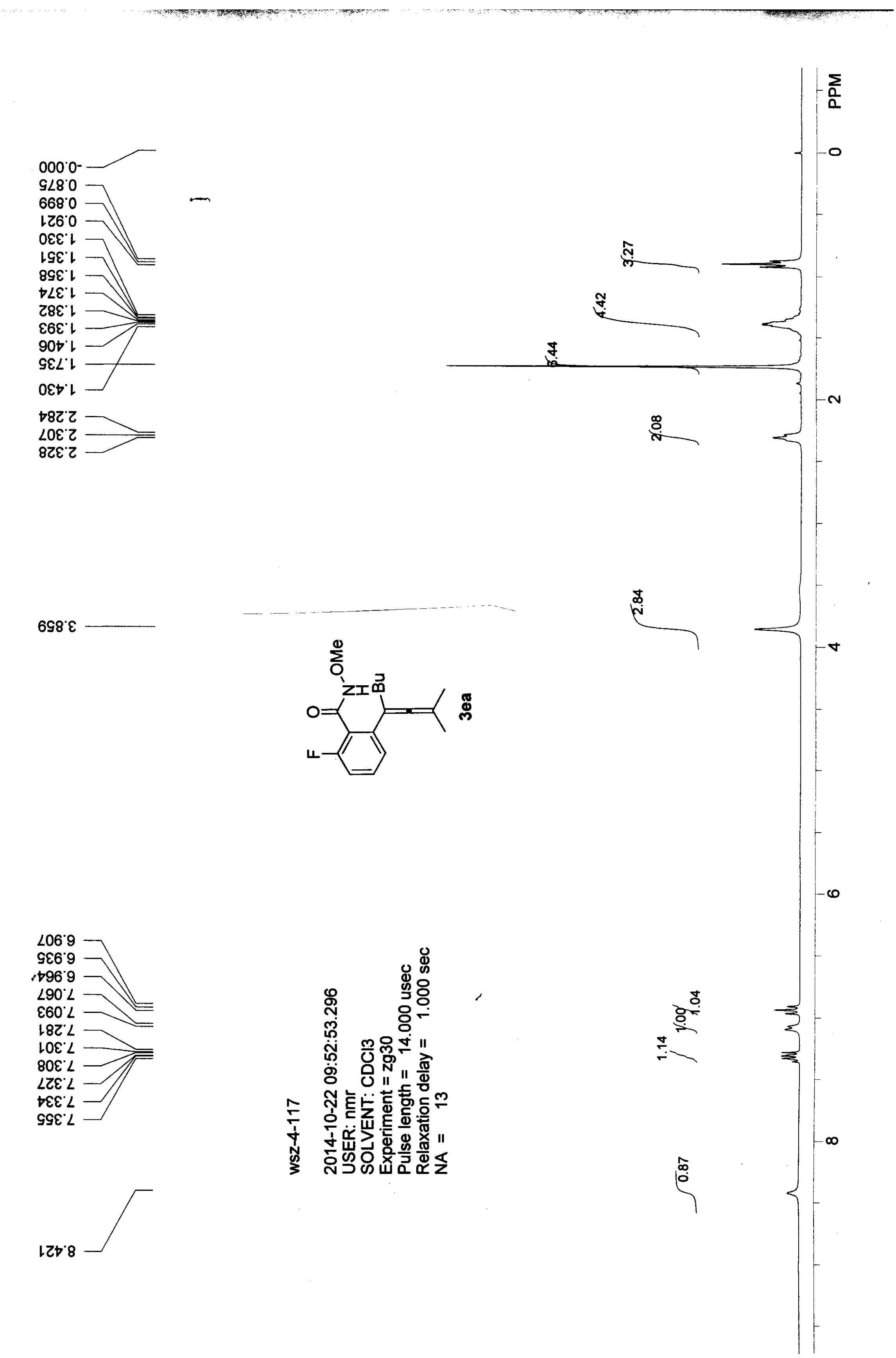

Supplementary Figure 15. ^{1}H NMR (300 MHz, $CDCl_3$) spectrum for 3ea.

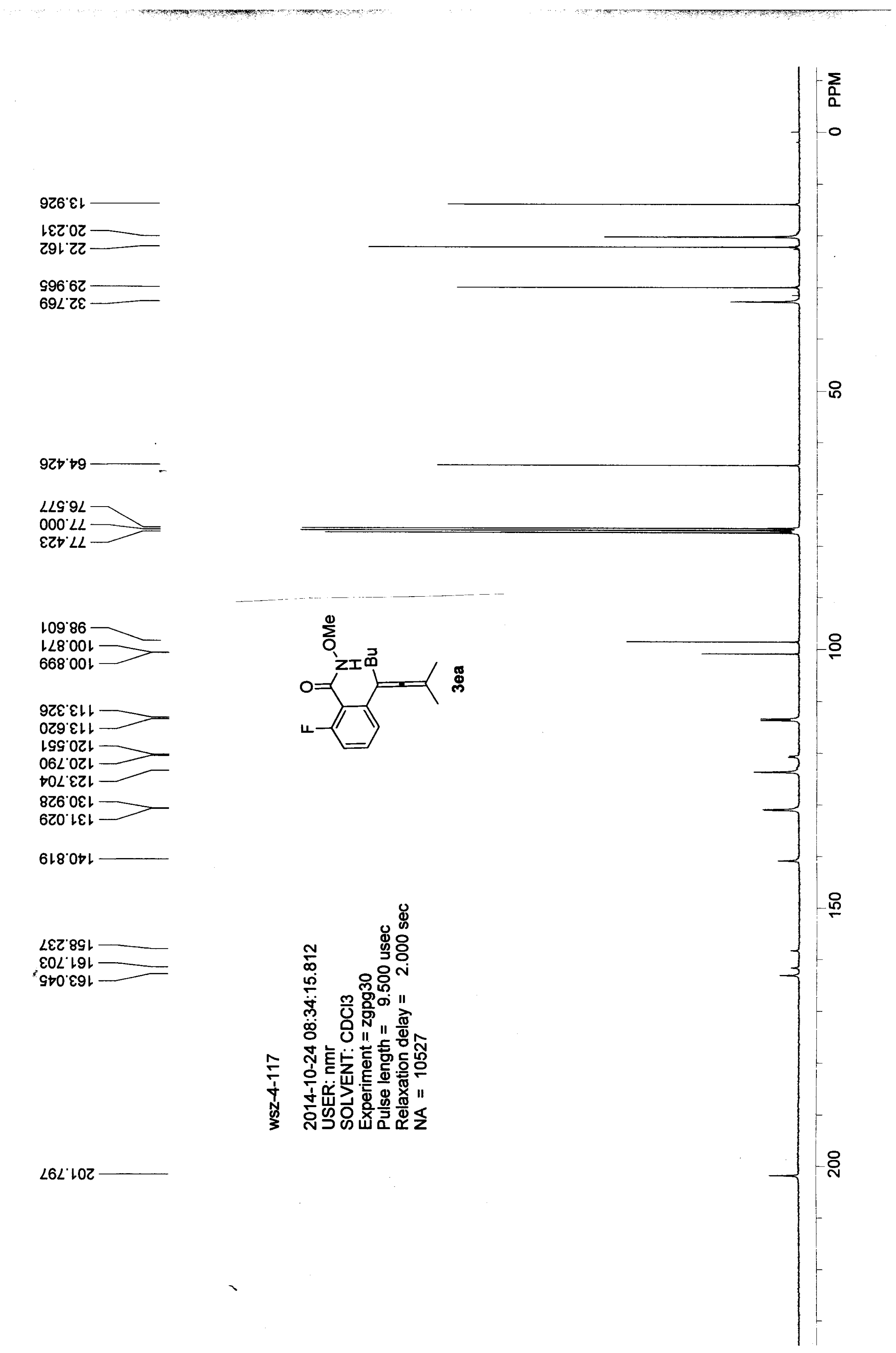

Supplementary Figure 16. ^{13}**C NMR (75 MHz,** $CDCl_3$**) spectrum for 3ea.**

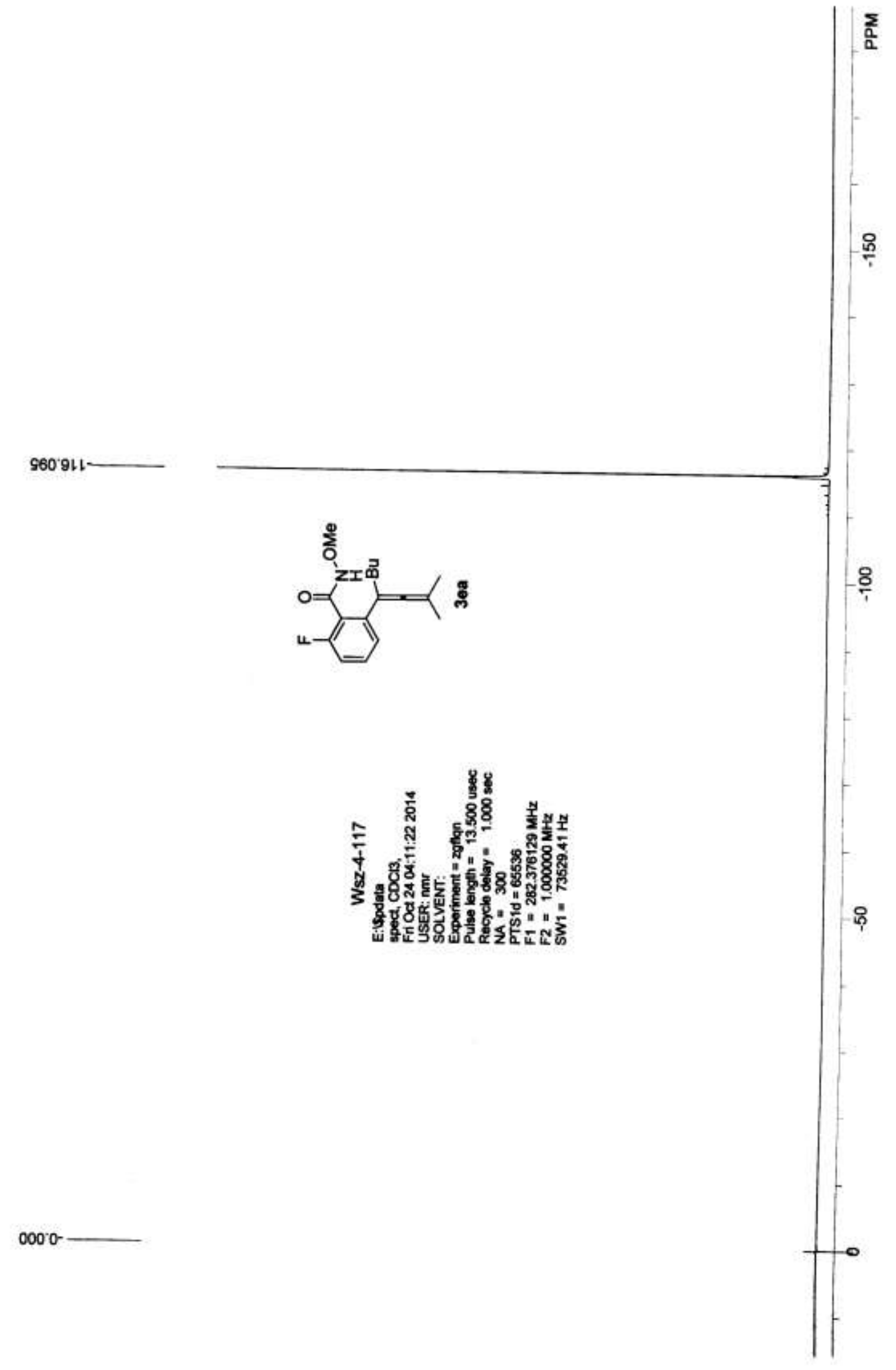

Supplementary Figure 17. ^{19}**F NMR (282 MHz,** $CDCl_3$**) spectrum for 3ea.**

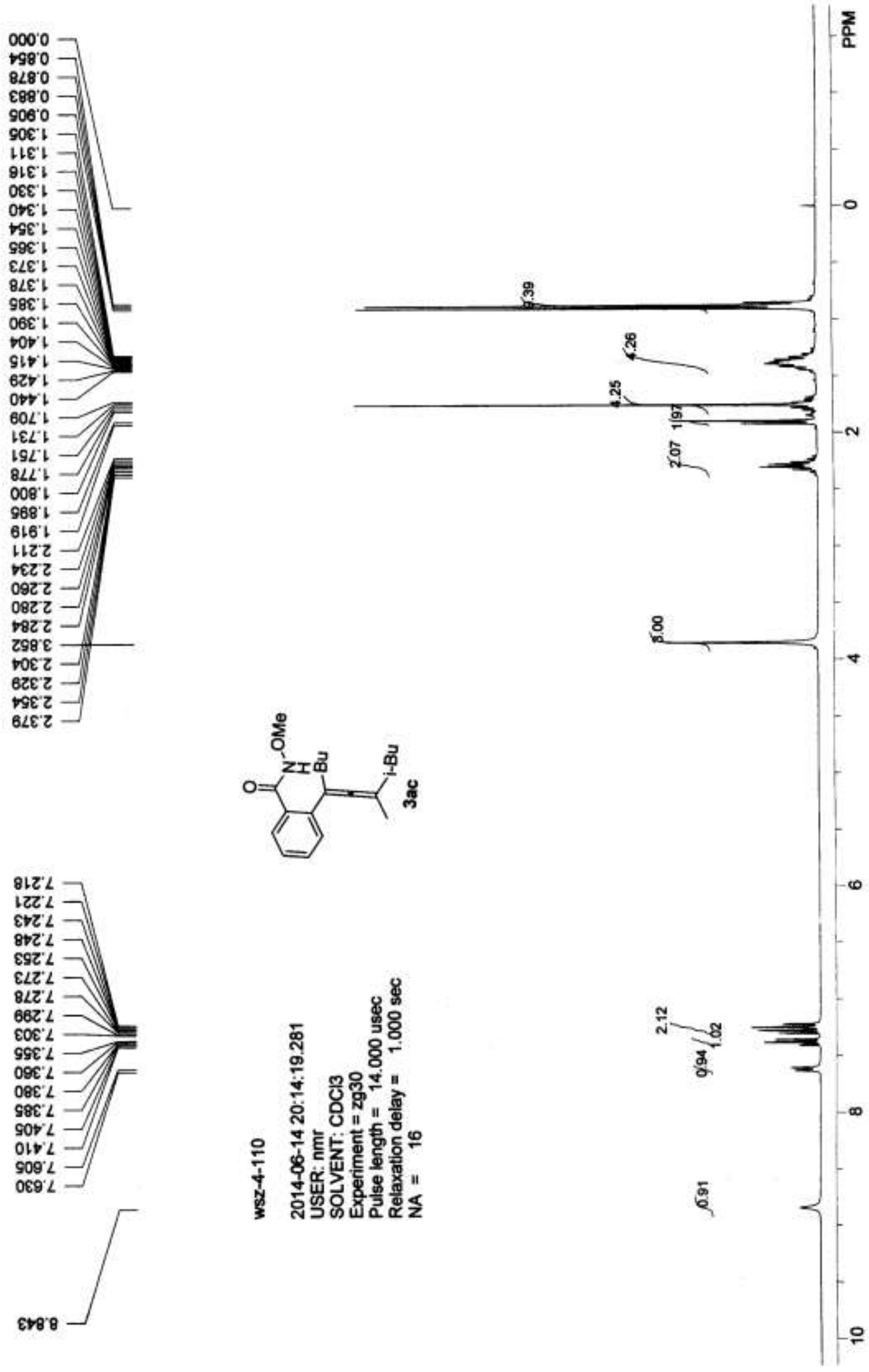

Supplementary Figure 18. ^{1}**H NMR (300 MHz, CDCl$_3$) spectrum for 3ac.**

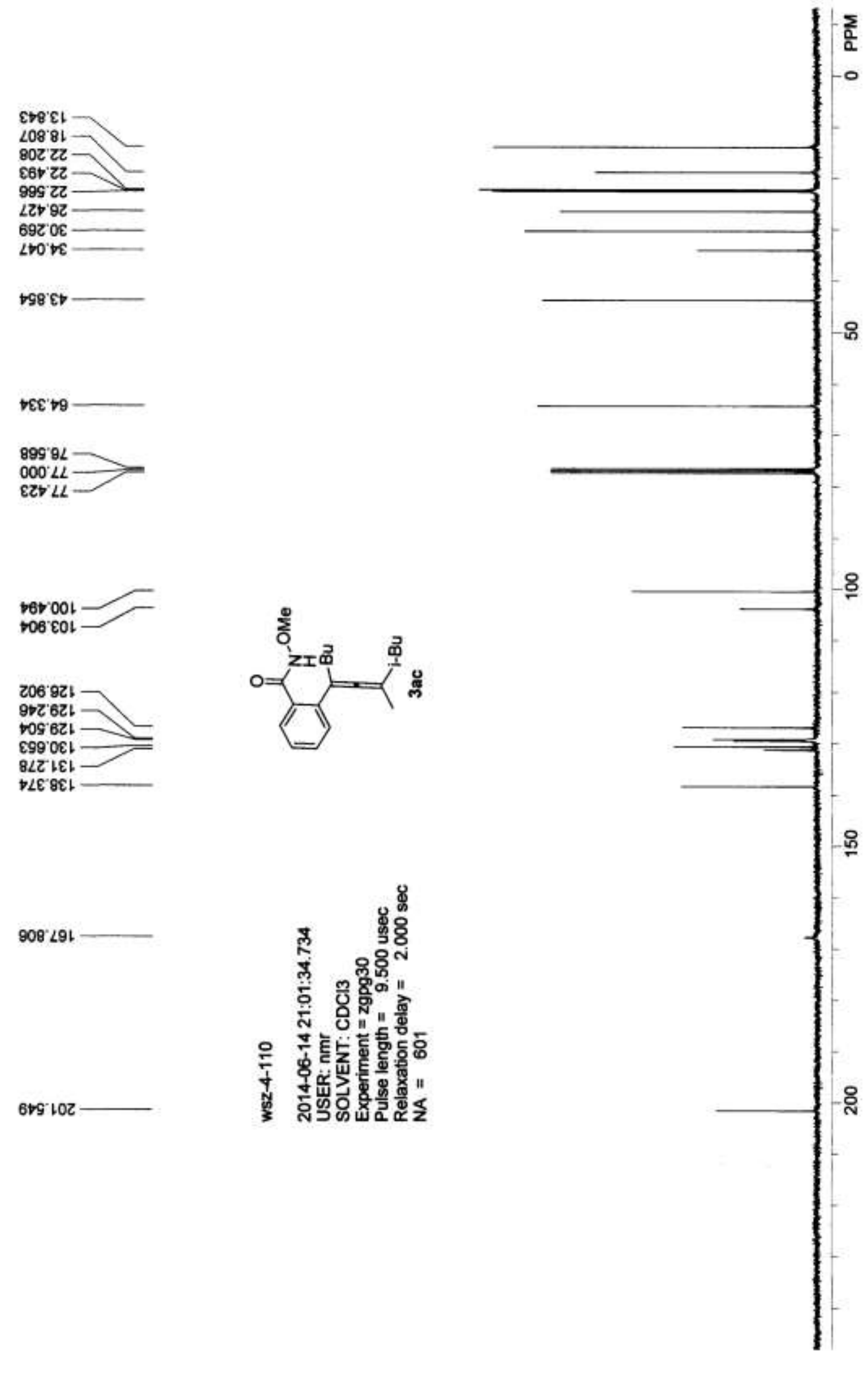

Supplementary Figure 19. ^{13}C NMR (75 MHz, $CDCl_3$) spectrum for 3ac.

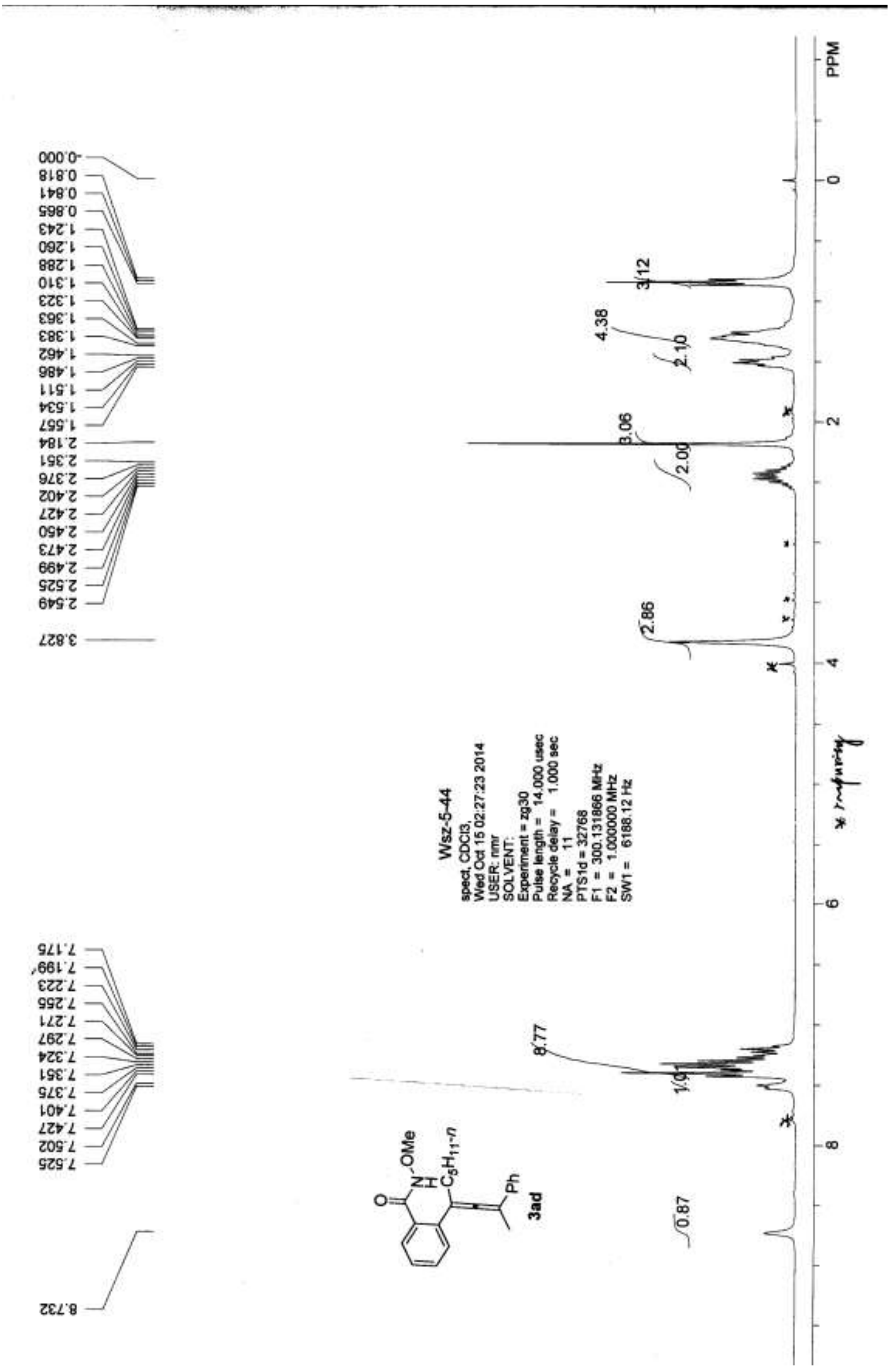

Supplementary Figure 20. ^{1}H NMR (300 MHz, $CDCl_3$) spectrum for 3ad.

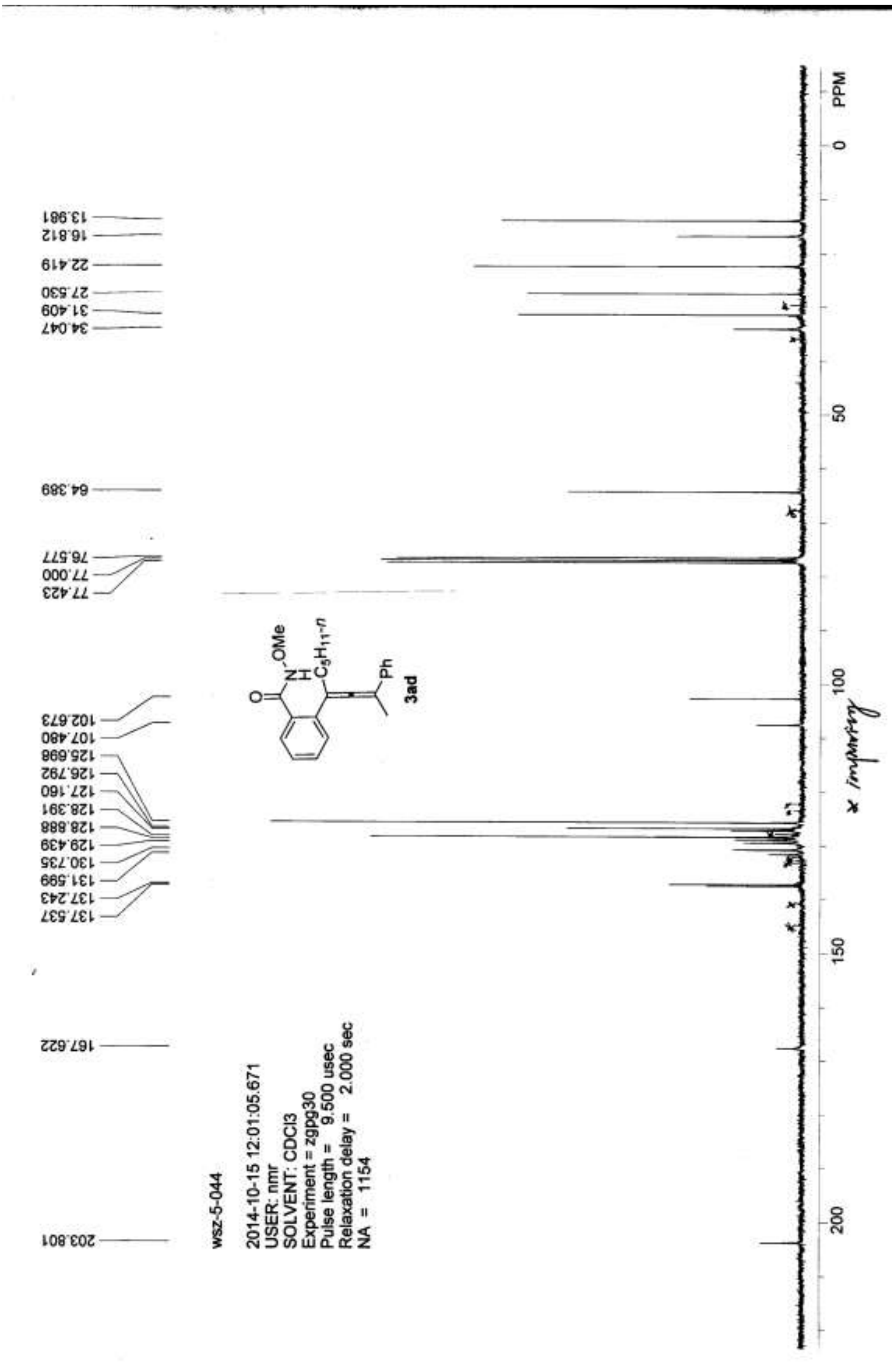

Supplementary Figure 21. ^{13}**C NMR (75 MHz, CDCl$_3$) spectrum for 3ad.**

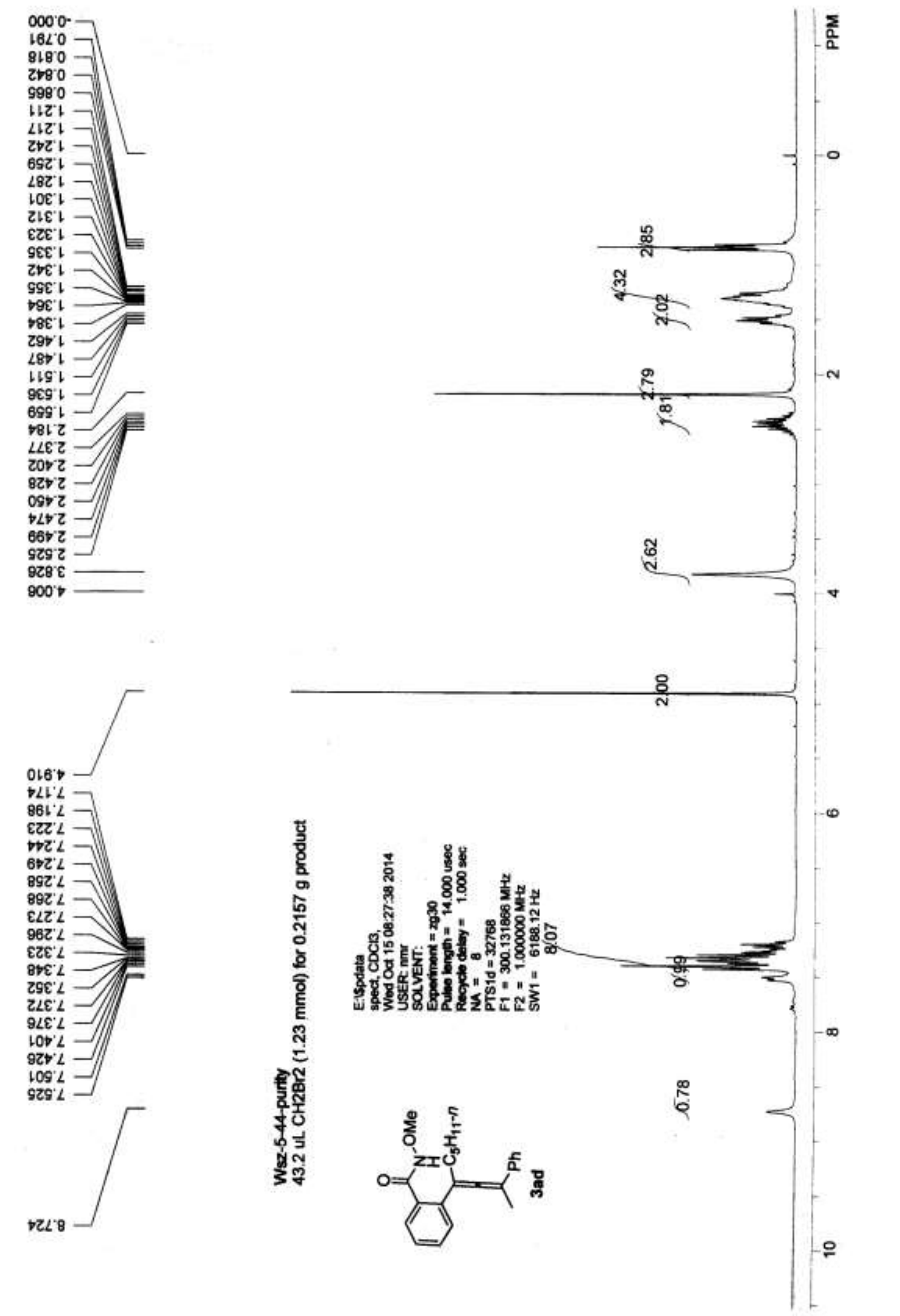

Supplementary Figure 22. **^{1}H NMR (300 MHz, $CDCl_3$) spectrum for the purity of 3ad.**

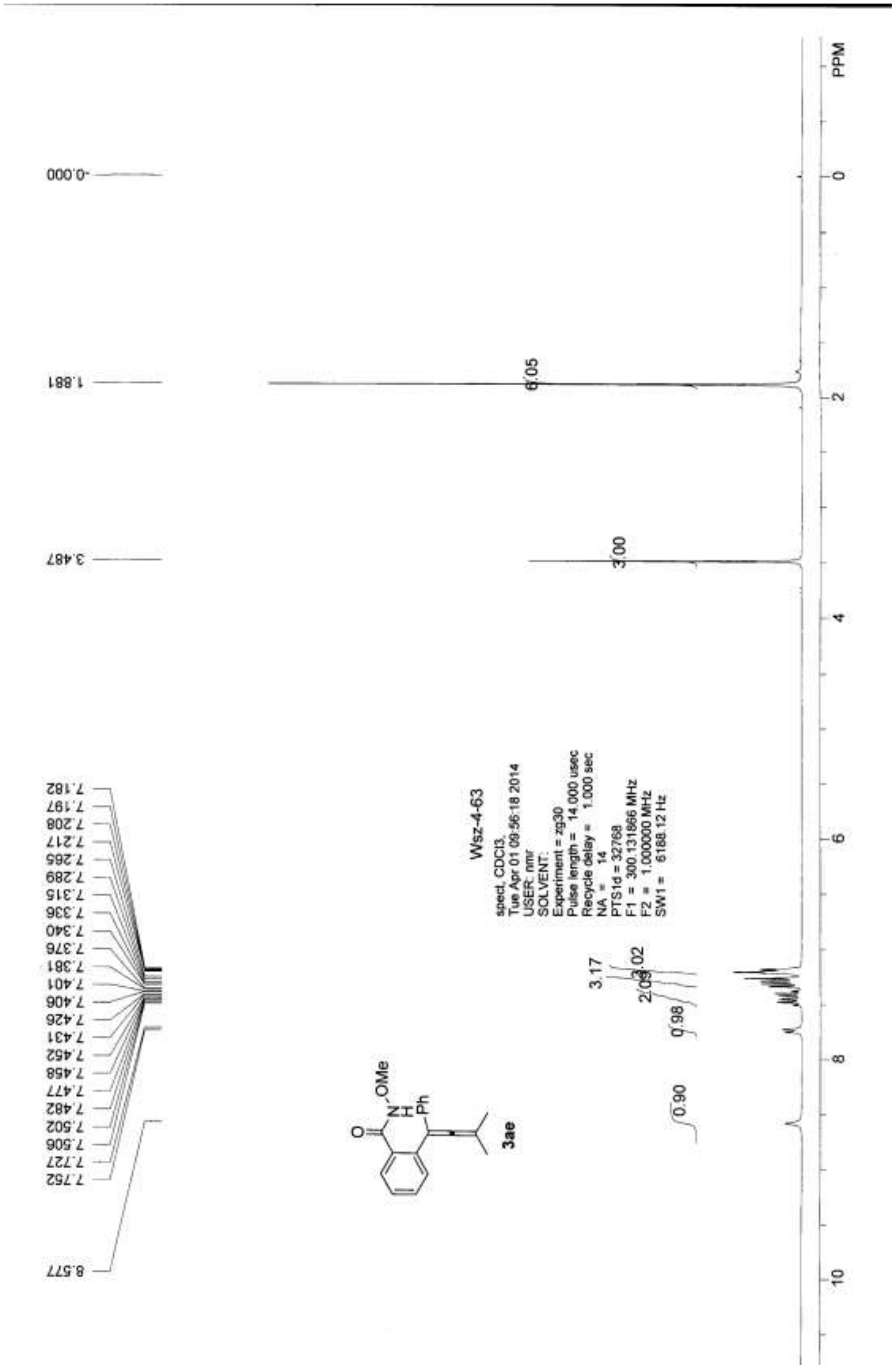

Supplementary Figure 23. ^{1}H NMR (300 MHz, $CDCl_3$) spectrum for 3ae.

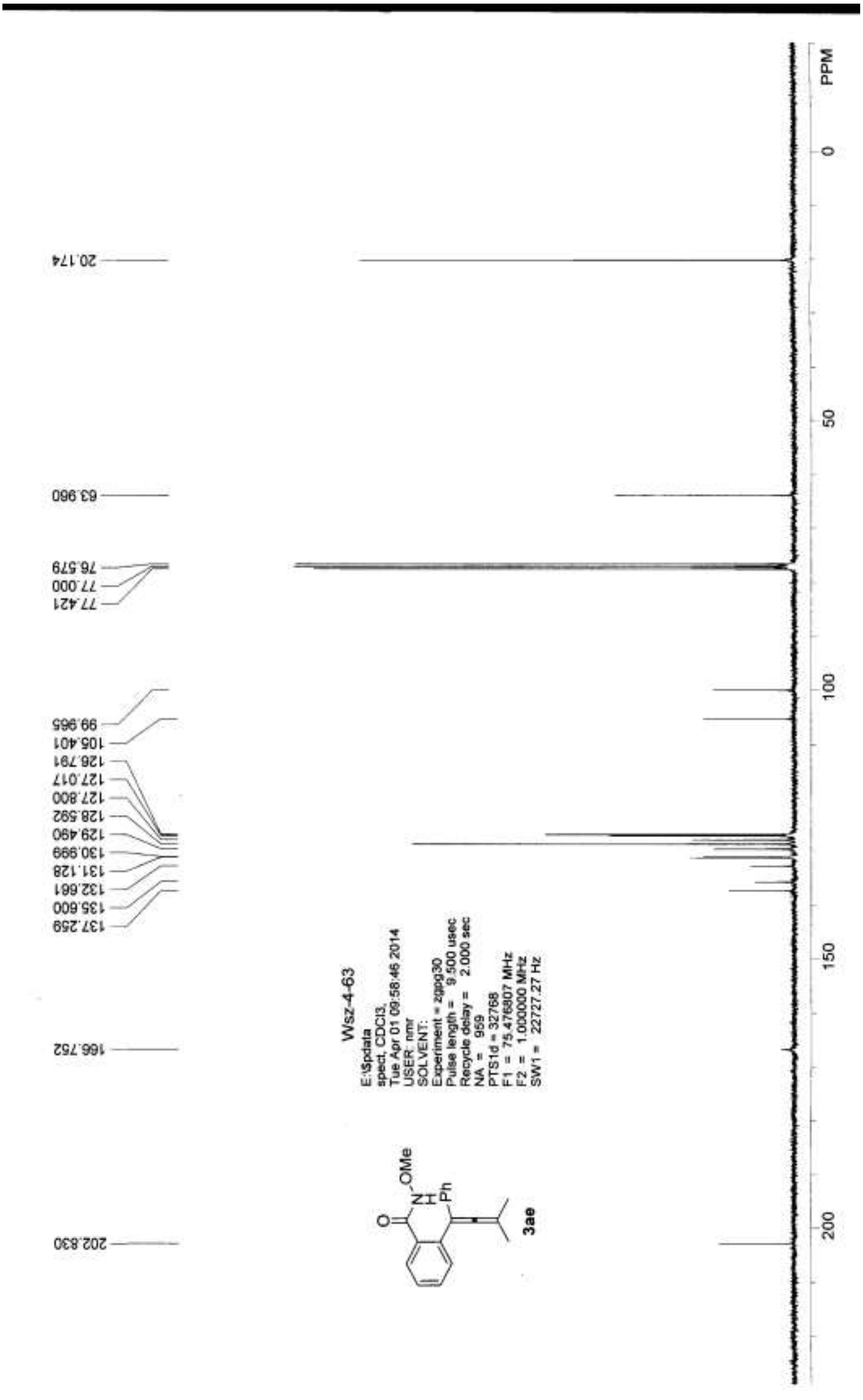

Supplementary Figure 24. ^{13}C NMR (75 MHz, $CDCl_3$) spectrum for 3ae.

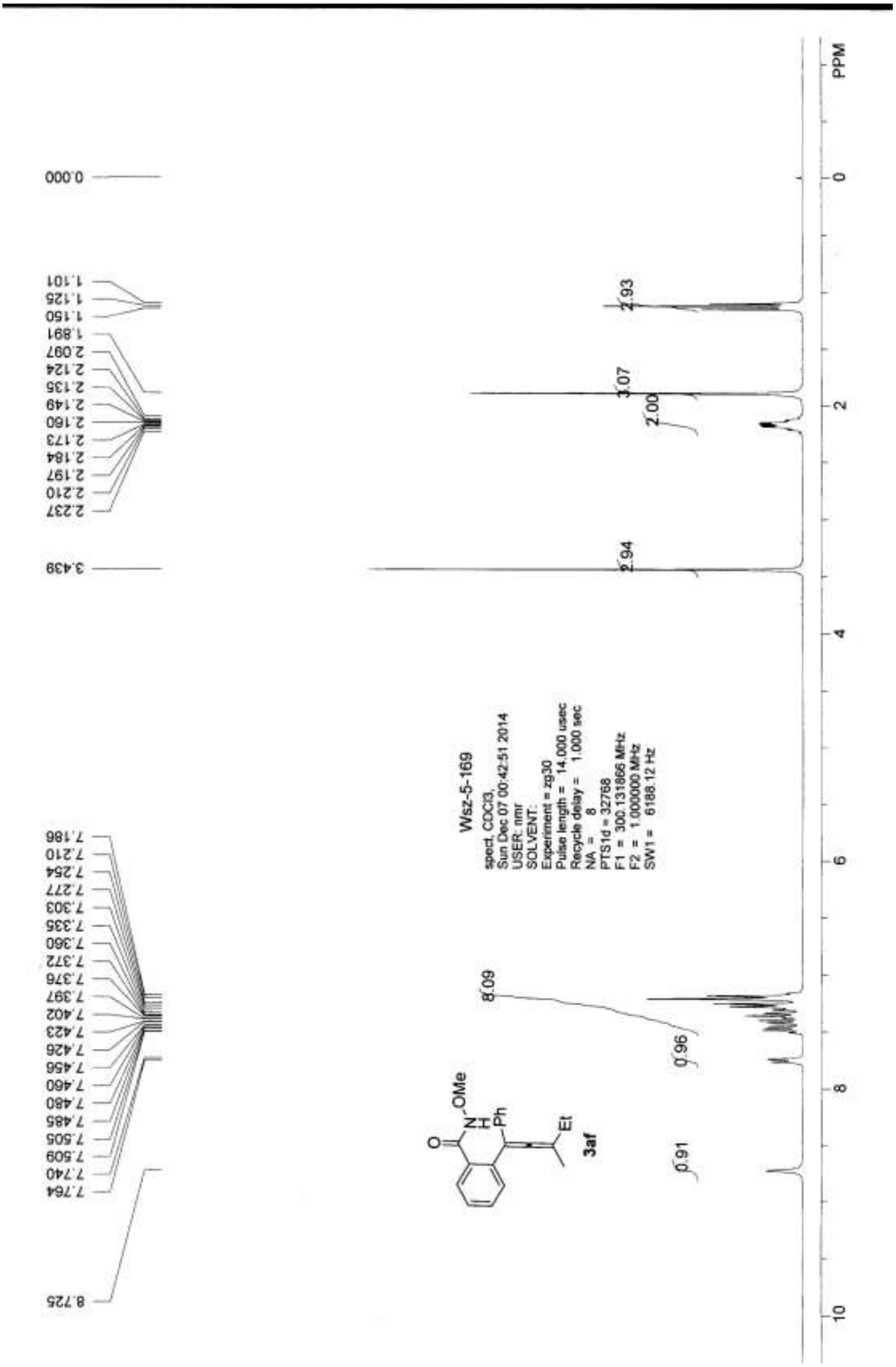

Supplementary Figure 25. ^{1}H NMR (300 MHz, $CDCl_3$) spectrum for 3af.

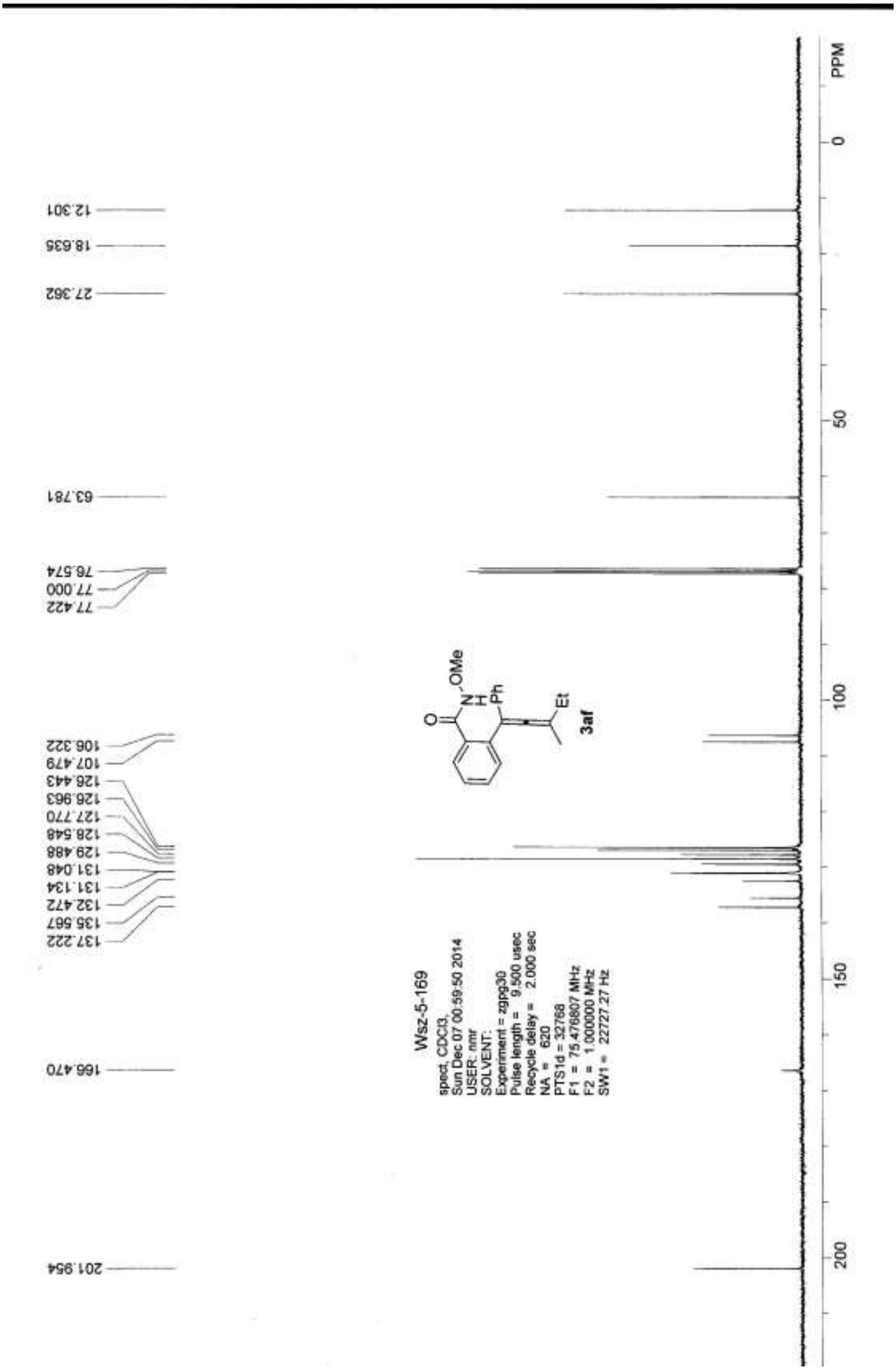

Supplementary Figure 26. ^{13}**C NMR (75 MHz,** $CDCl_3$**) spectrum for 3af.**

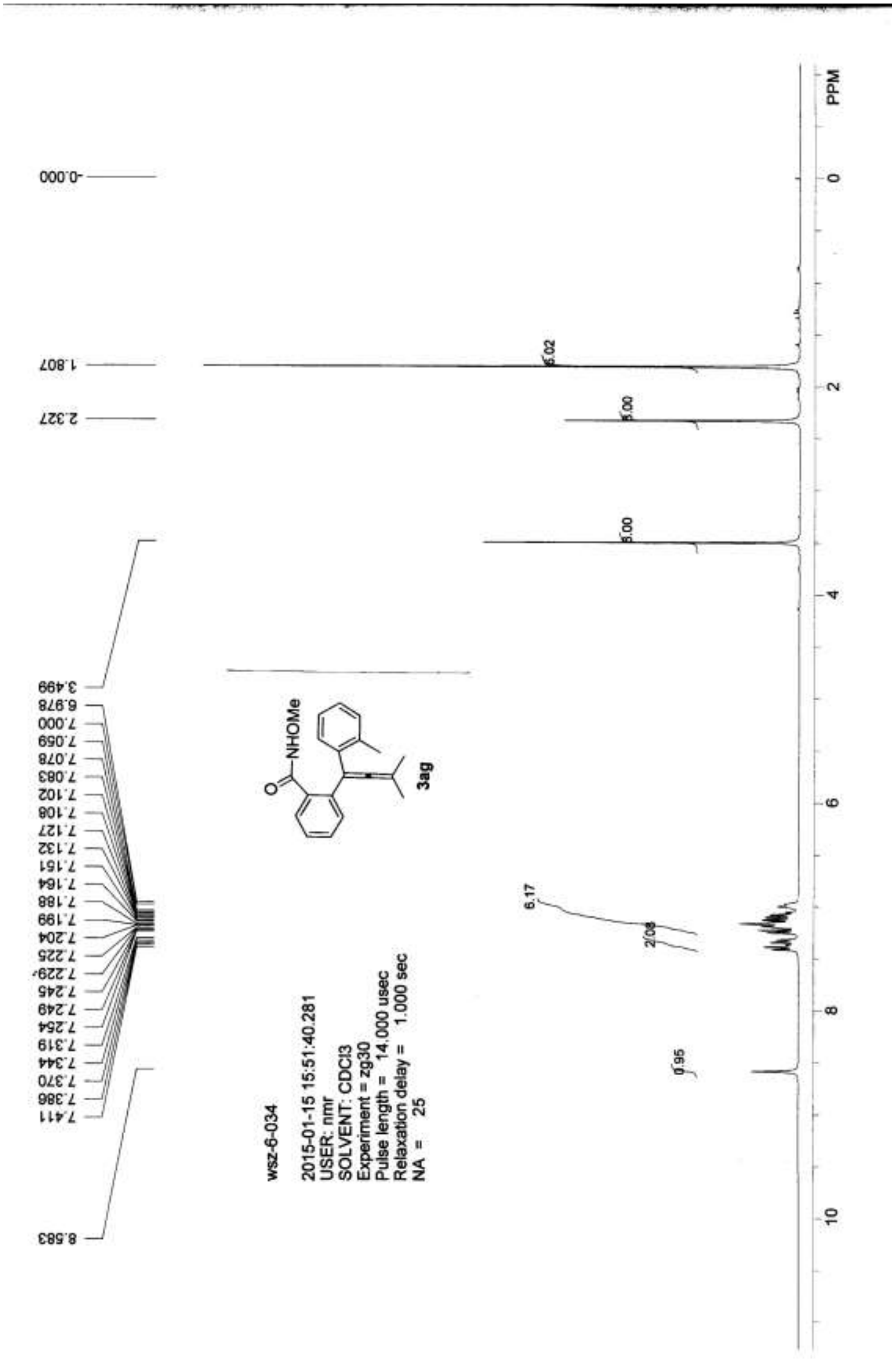

Supplementary Figure 27. ^{1}H NMR (300 MHz, $CDCl_3$) spectrum for 3ag.

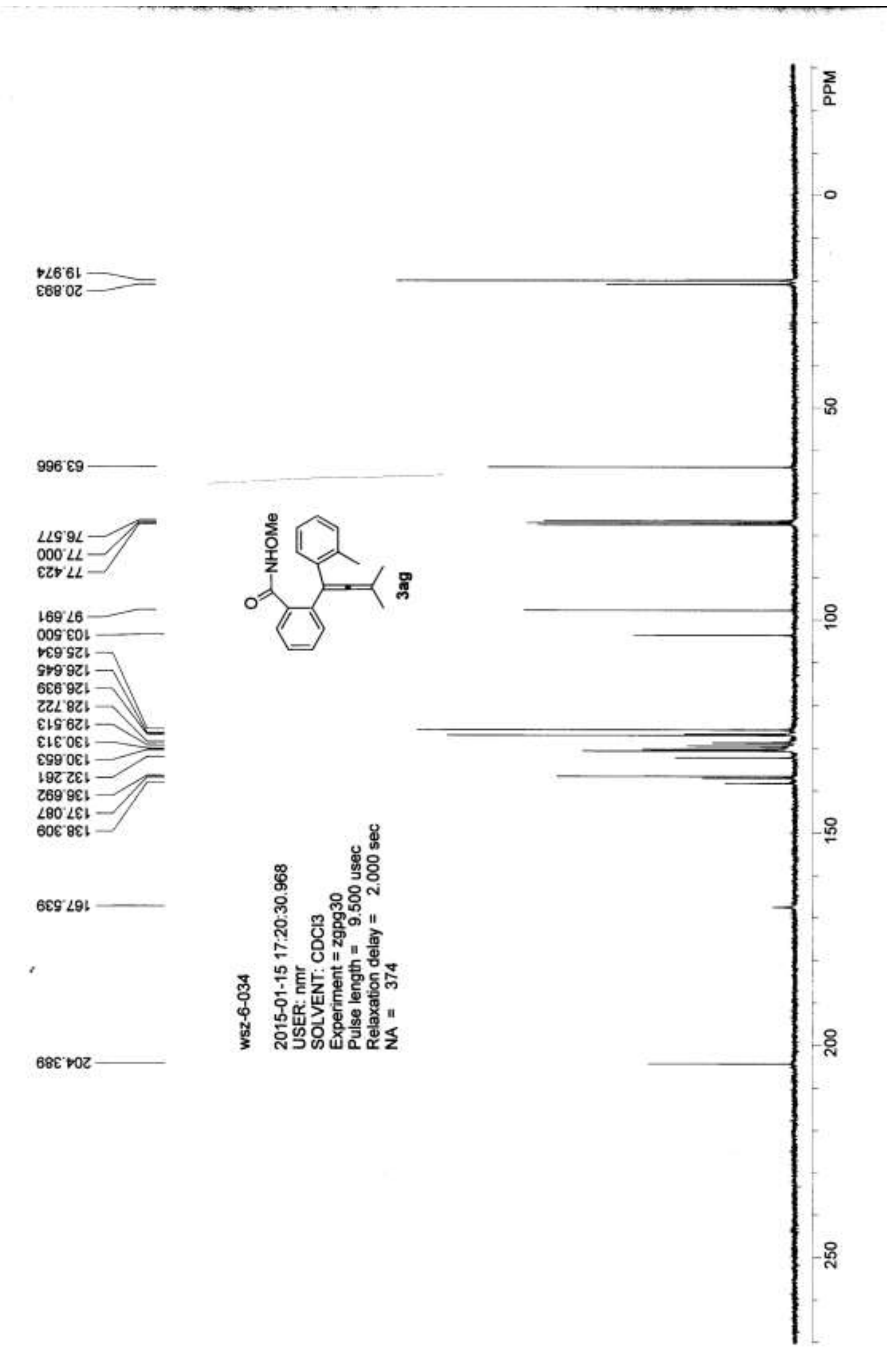

Supplementary Figure 28. ^{13}**C NMR (75 MHz, CDCl$_3$) spectrum for 3ag.**

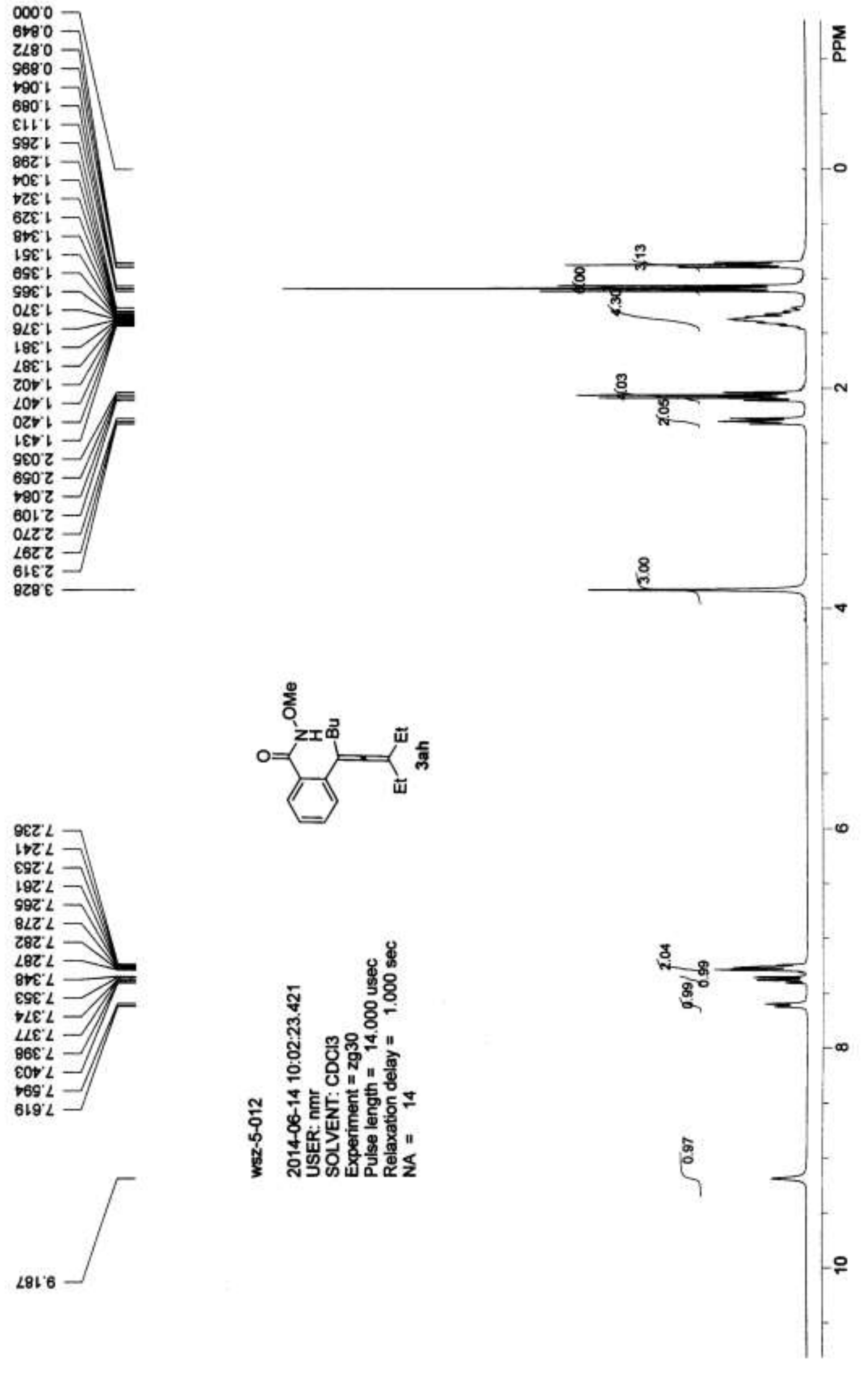

Supplementary Figure 29. **^{1}H NMR (300 MHz, $CDCl_3$) spectrum for 3ah.**

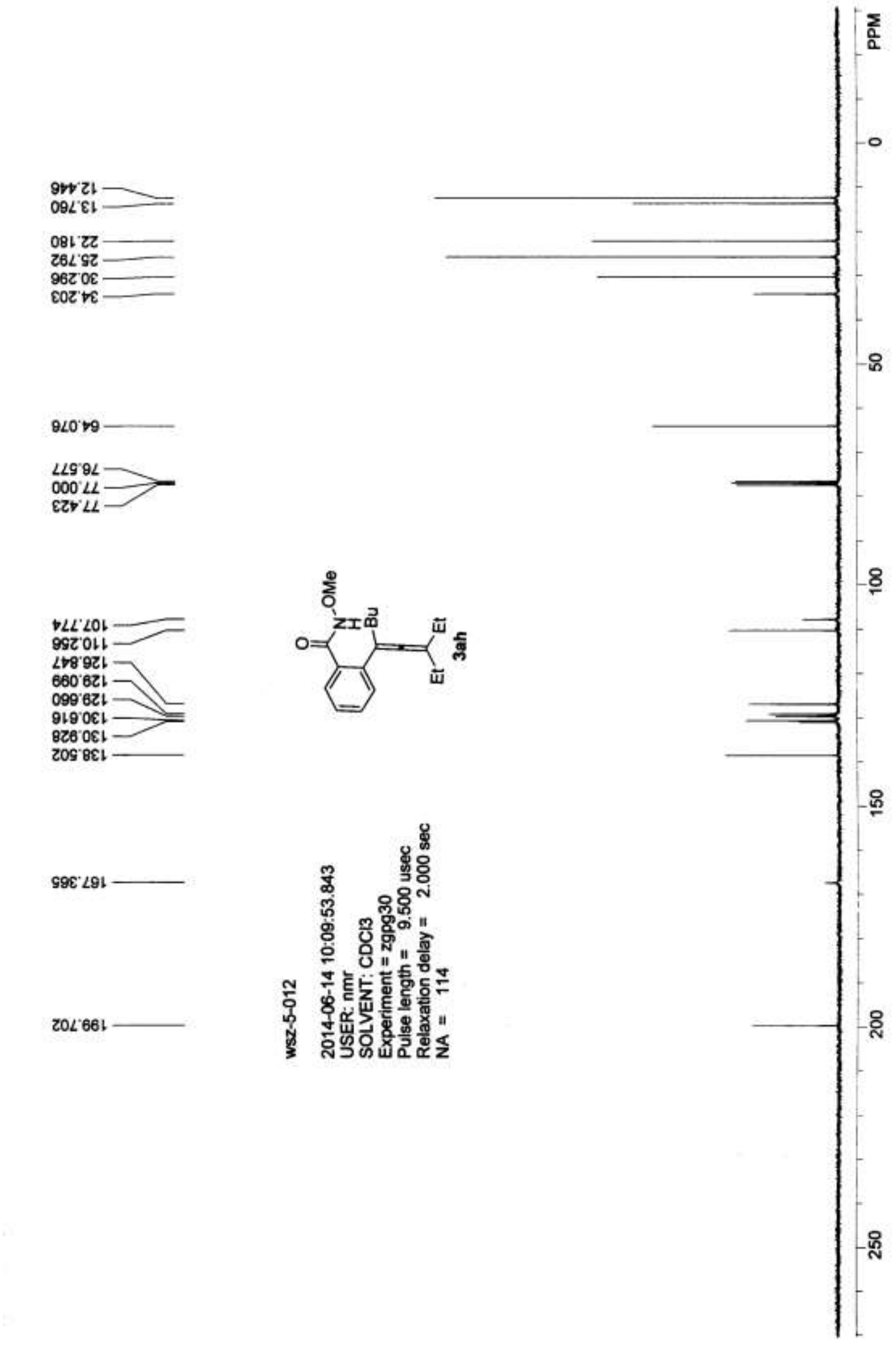

Supplementary Figure 30. ^{13}C NMR (75 MHz, $CDCl_3$) spectrum for 3ah.

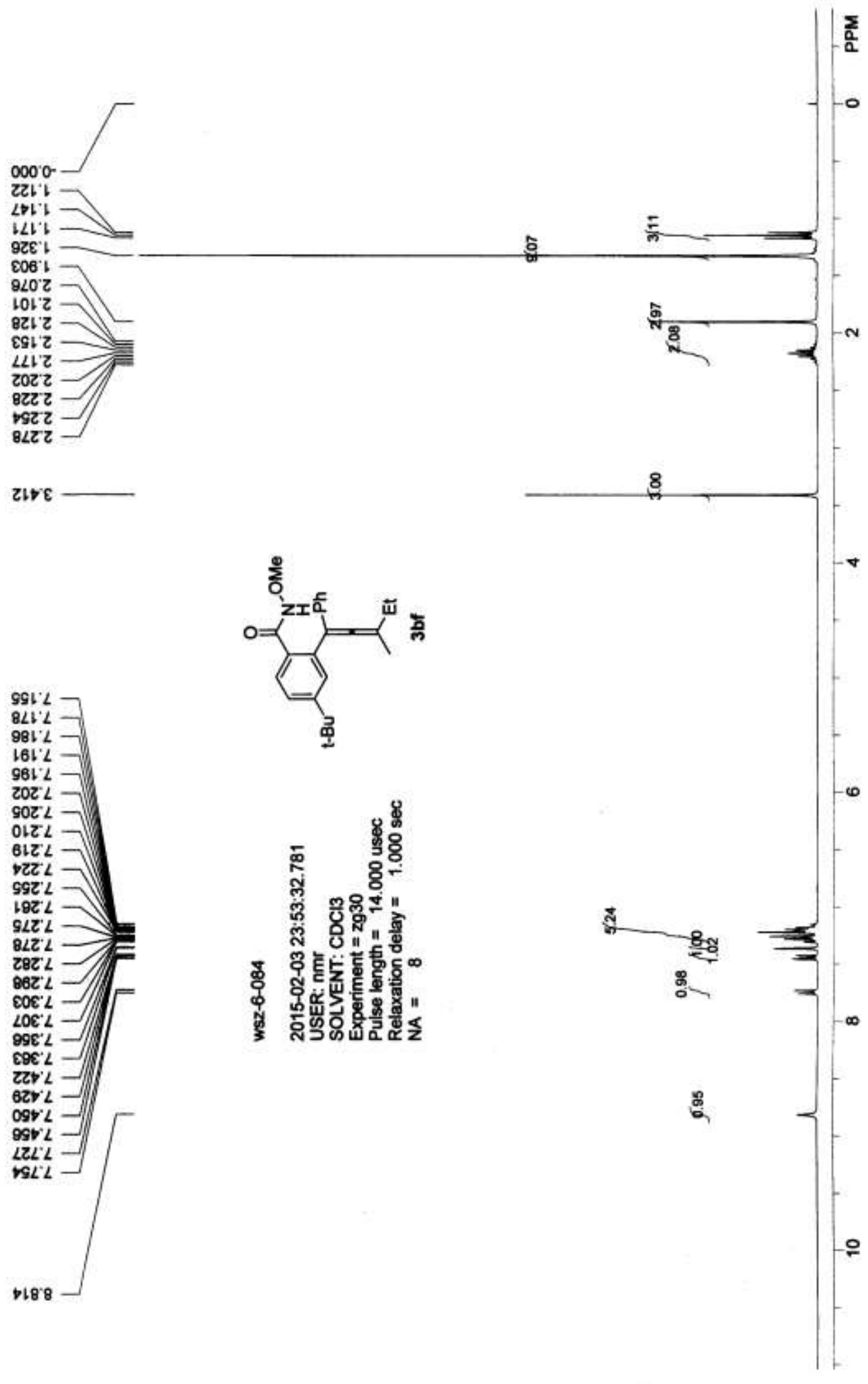

Supplementary Figure 31. ^{1}**H NMR (300 MHz,** $CDCl_3$**) spectrum for 3bf.**

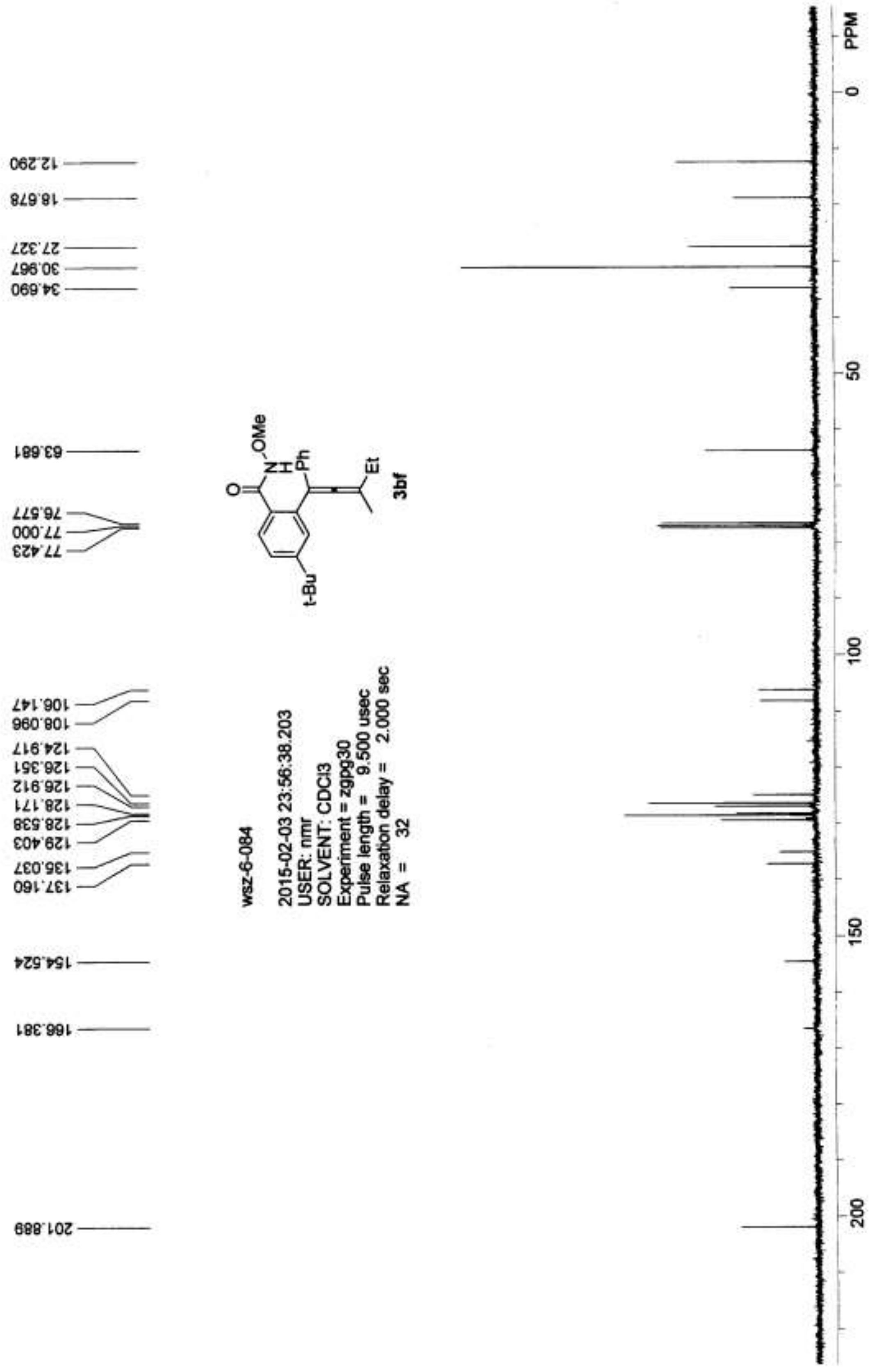

Supplementary Figure 32. ^{13}C NMR (75 MHz, $CDCl_3$) spectrum for 3bf.

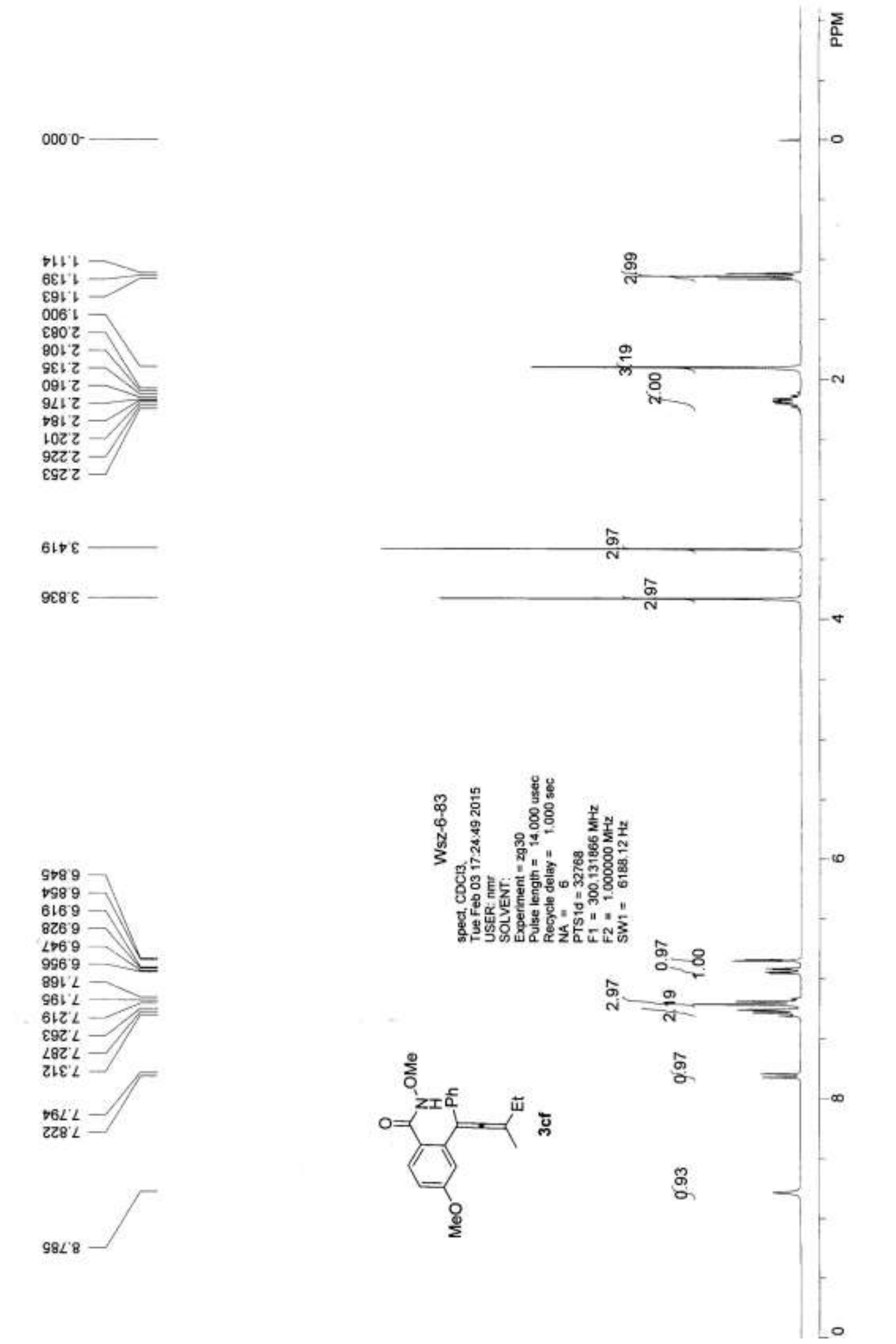

Supplementary Figure 33. ^{1}H NMR (300 MHz, $CDCl_3$) spectrum for 3cf.

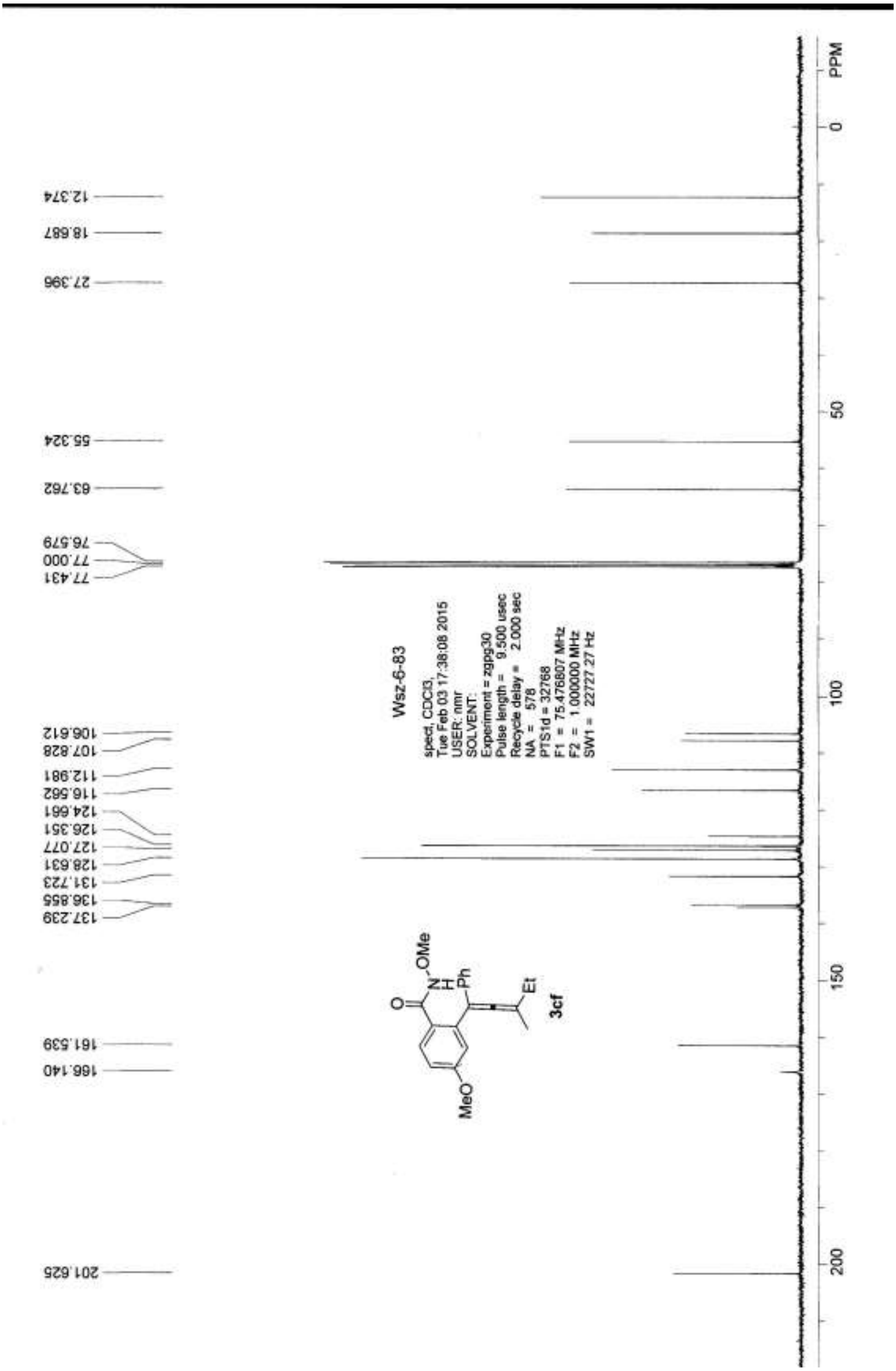

Supplementary Figure 34. ^{13}C NMR (75 MHz, $CDCl_3$) spectrum for 3cf.

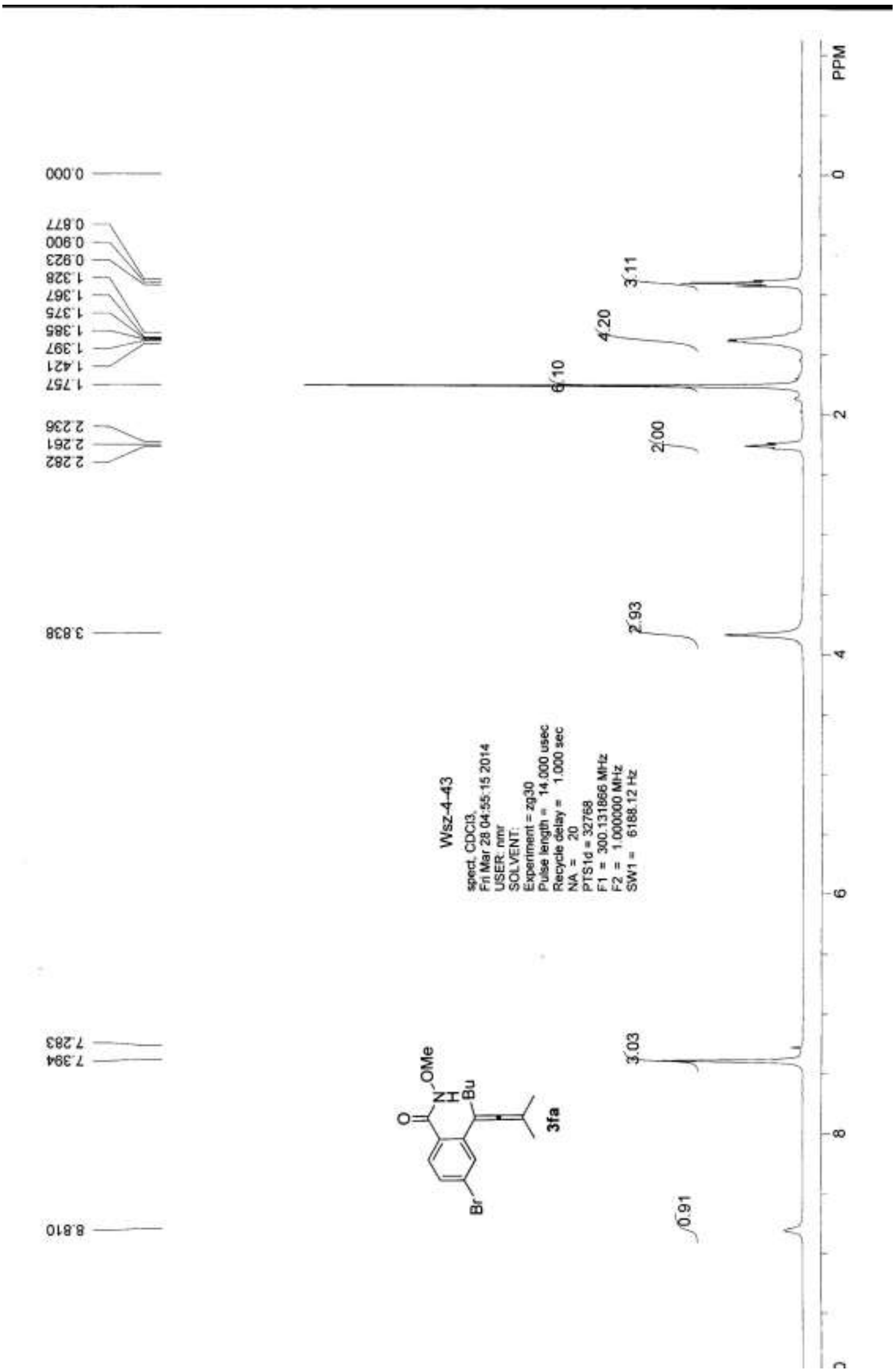

Supplementary Figure 35. ^{1}H NMR (300 MHz, $CDCl_3$) spectrum for 3fa.

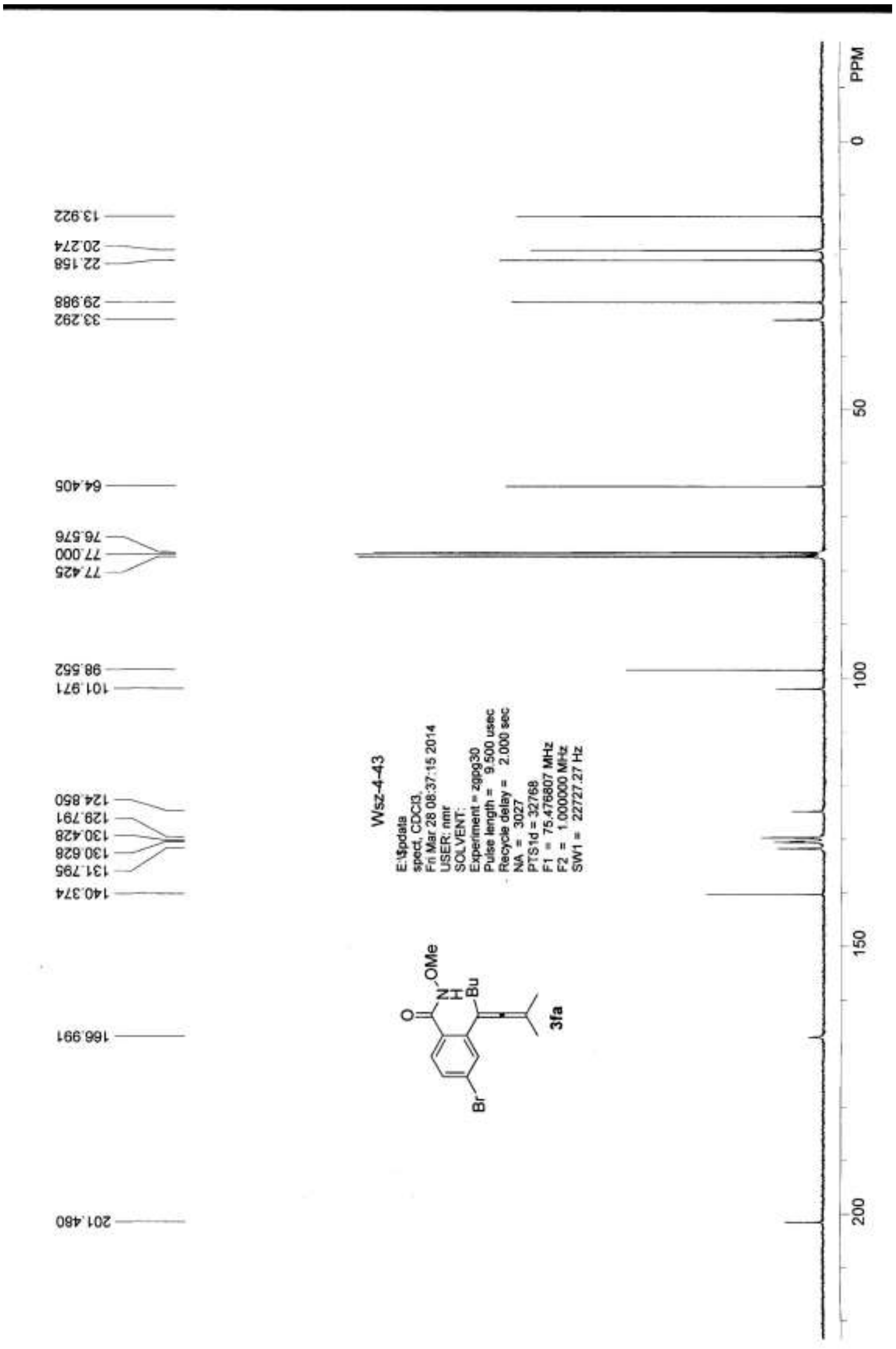

Supplementary Figure 36. ^{13}C NMR (75 MHz, $CDCl_3$) spectrum for 3fa.

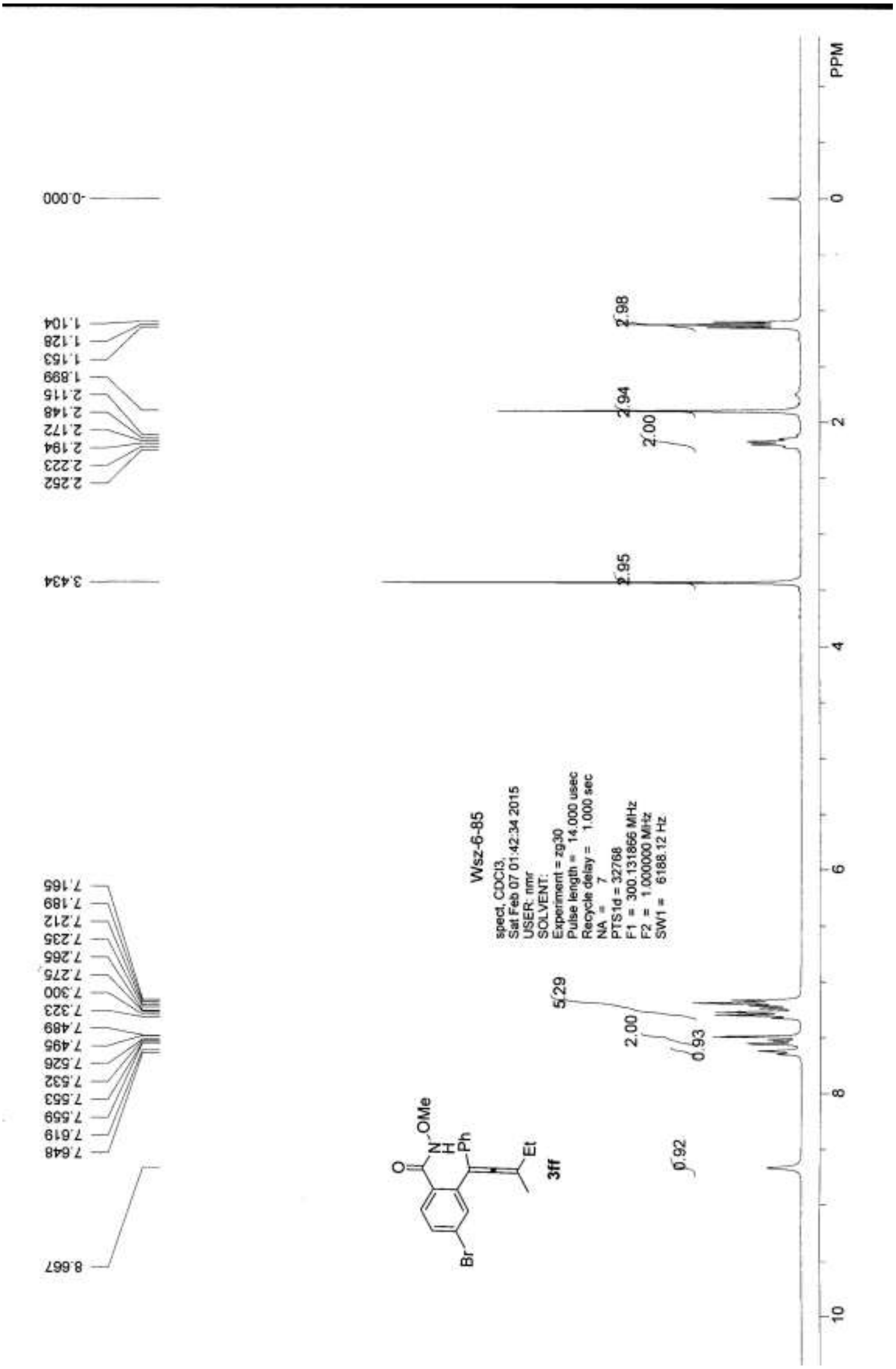

Supplementary Figure 37. ^{1}**H NMR (300 MHz,** $CDCl_3$**) spectrum for 3ff.**

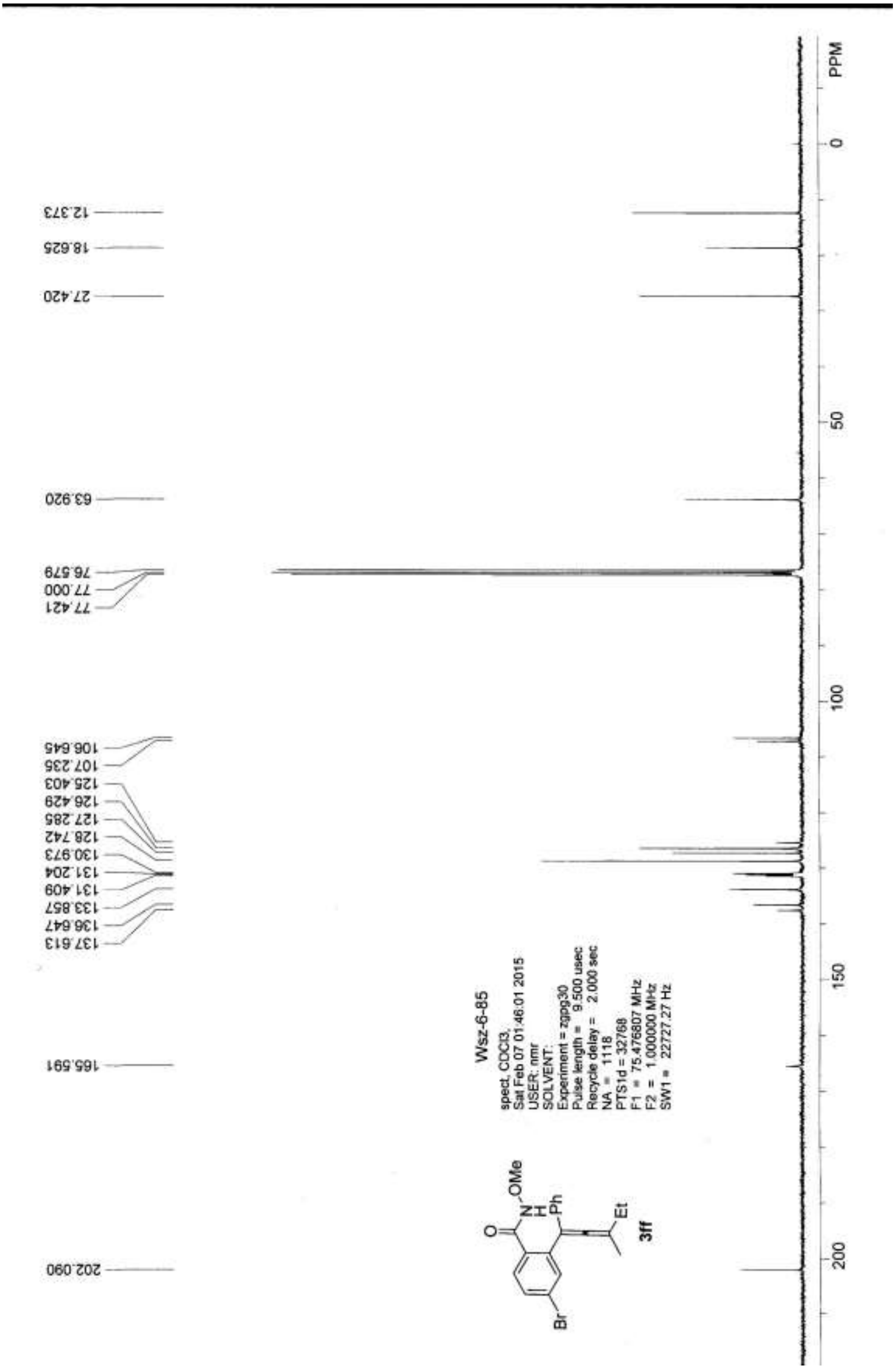

Supplementary Figure 38. ^{13}C **NMR (75 MHz,** $CDCl_3$**) spectrum for 3ff.**

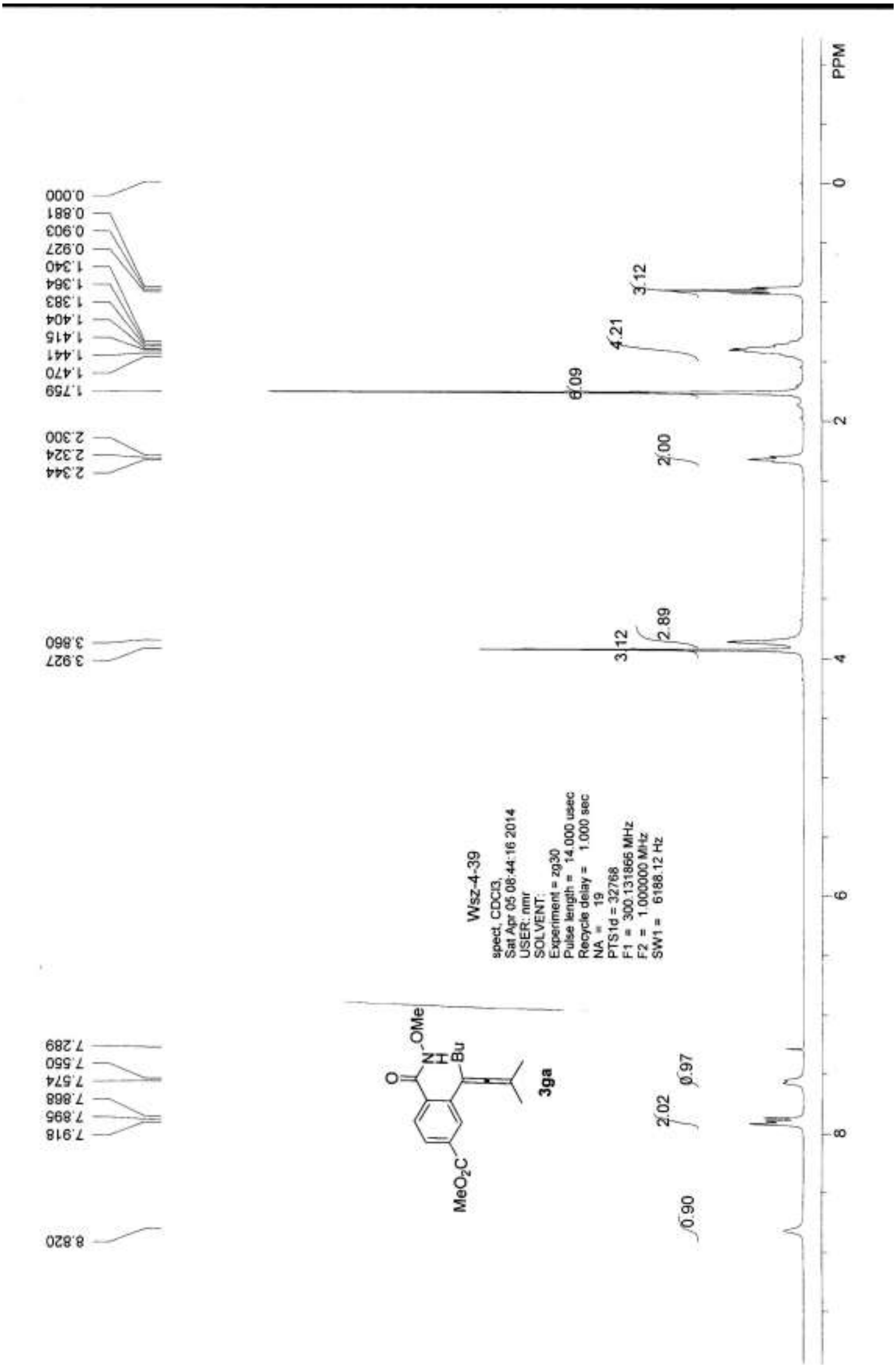

Supplementary Figure 39. ^{1}H NMR (300 MHz, $CDCl_3$) spectrum for 3ga.

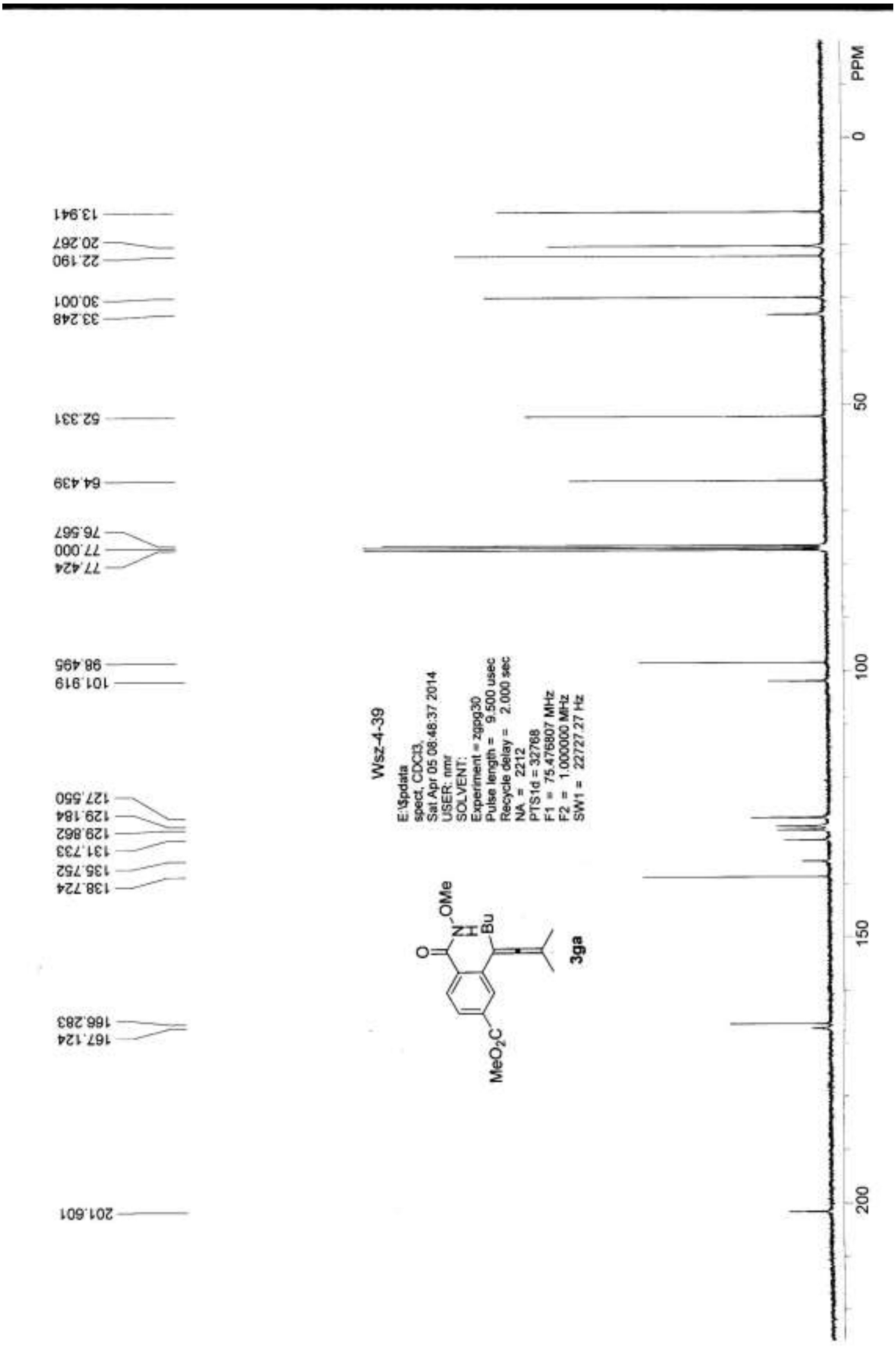

Supplementary Figure 40. ^{13}C NMR (75 MHz, $CDCl_3$) spectrum for 3ga.

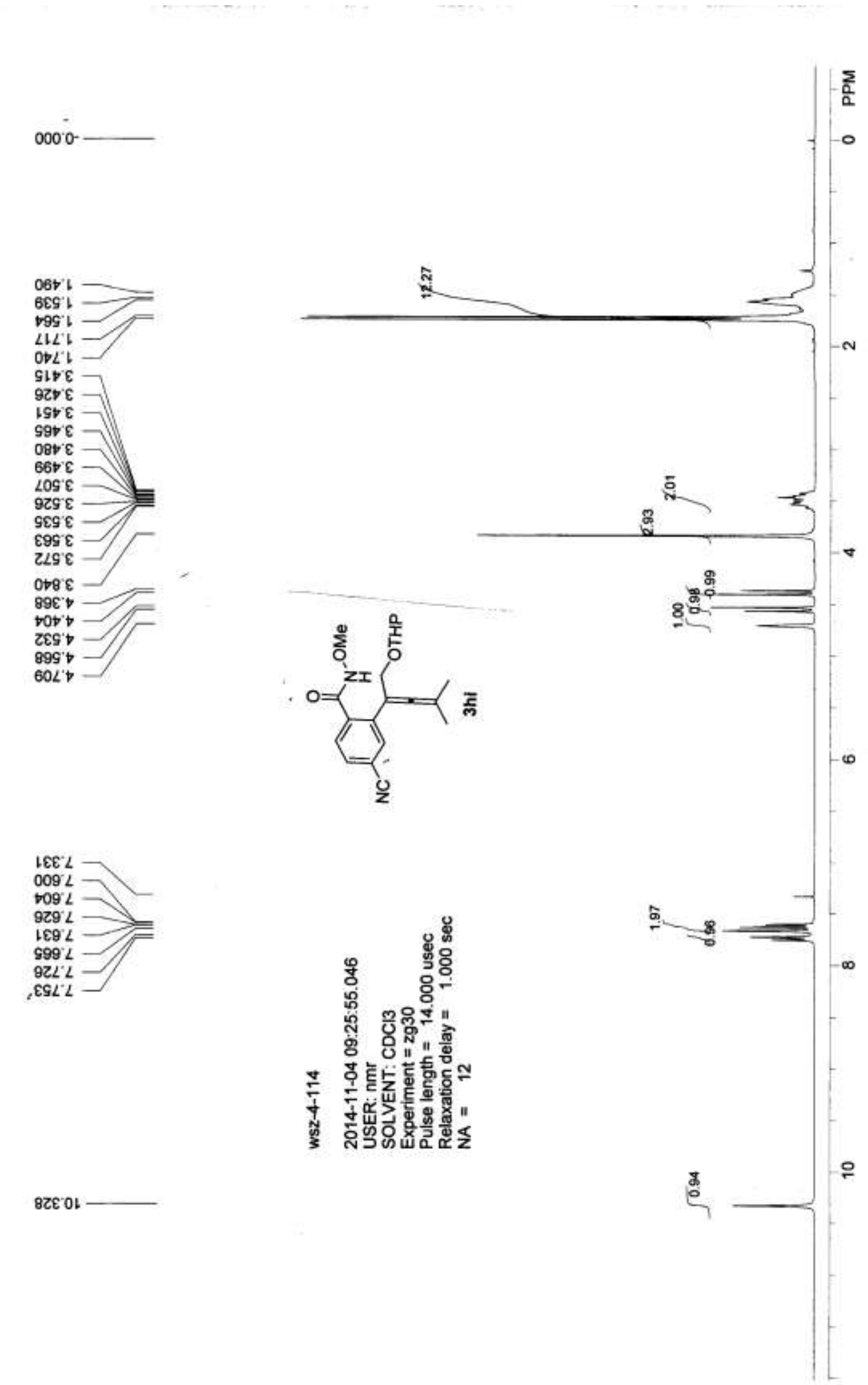

Supplementary Figure 41. ^{1}H NMR (300 MHz, $CDCl_3$) spectrum for 3hi.

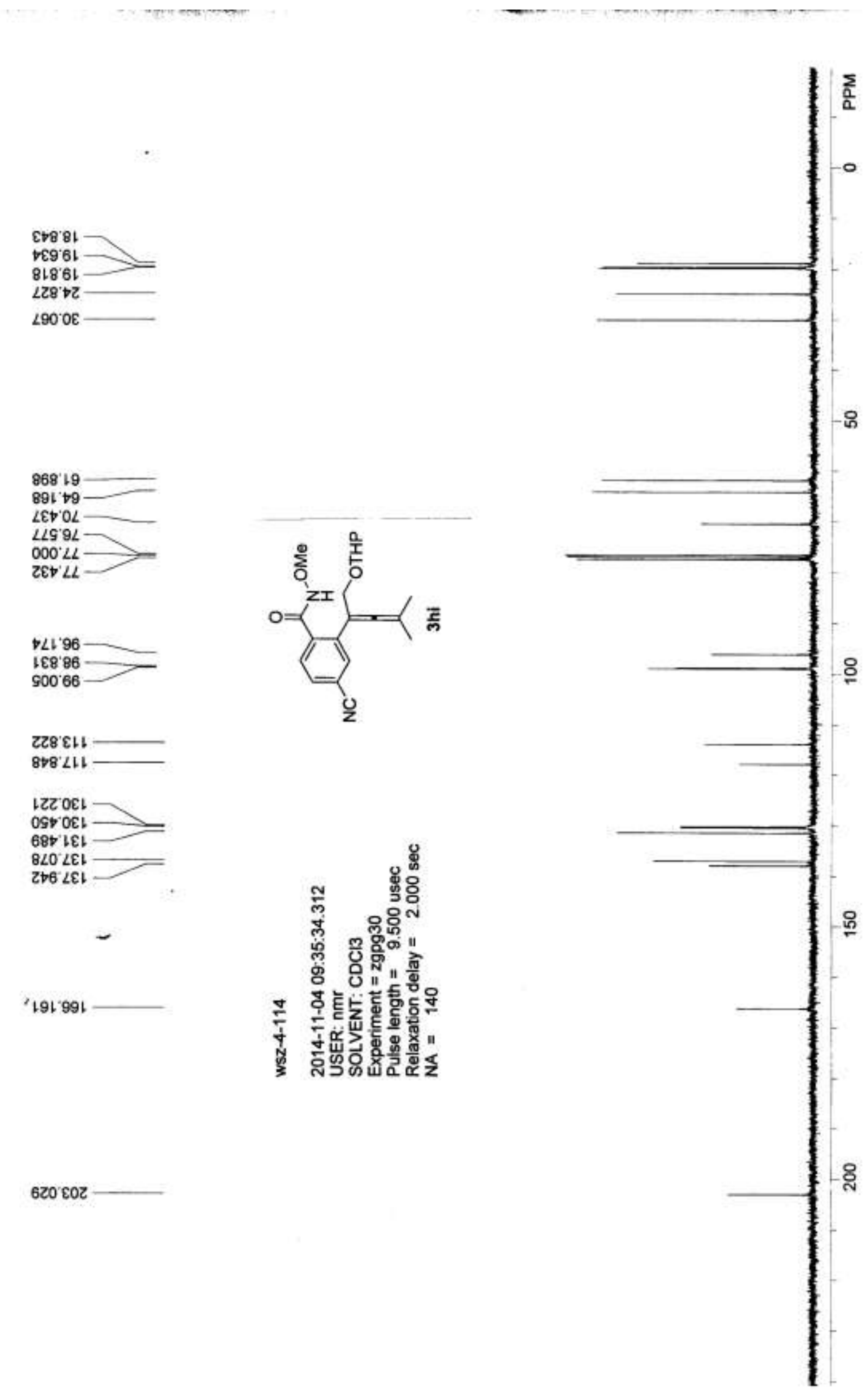

Supplementary Figure 42. ^{13}**C NMR (75 MHz,** $CDCl_3$**) spectrum for 3hi.**

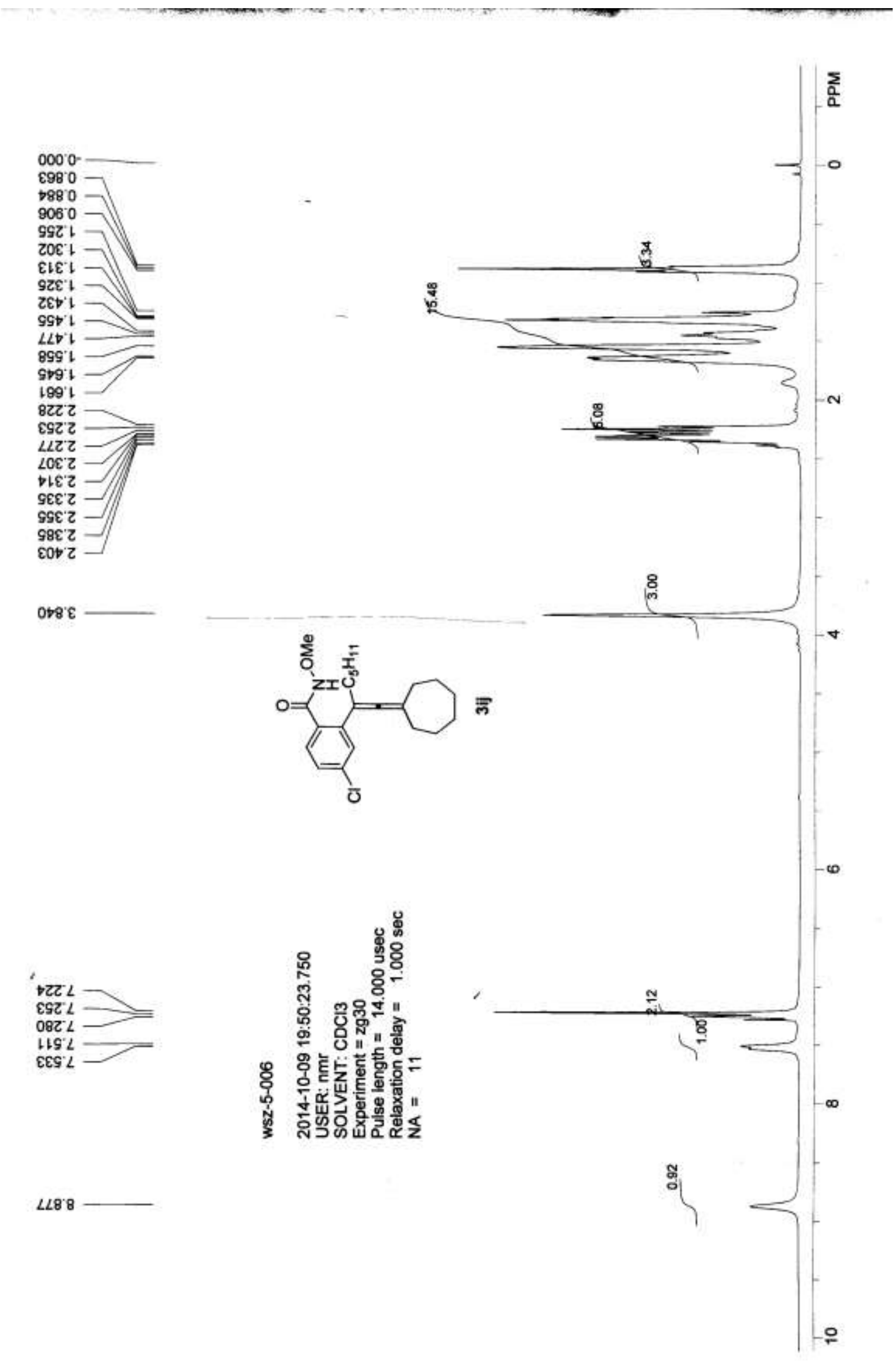

Supplementary Figure 43. ^{1}H NMR (300 MHz, $CDCl_3$) spectrum for 3ij.

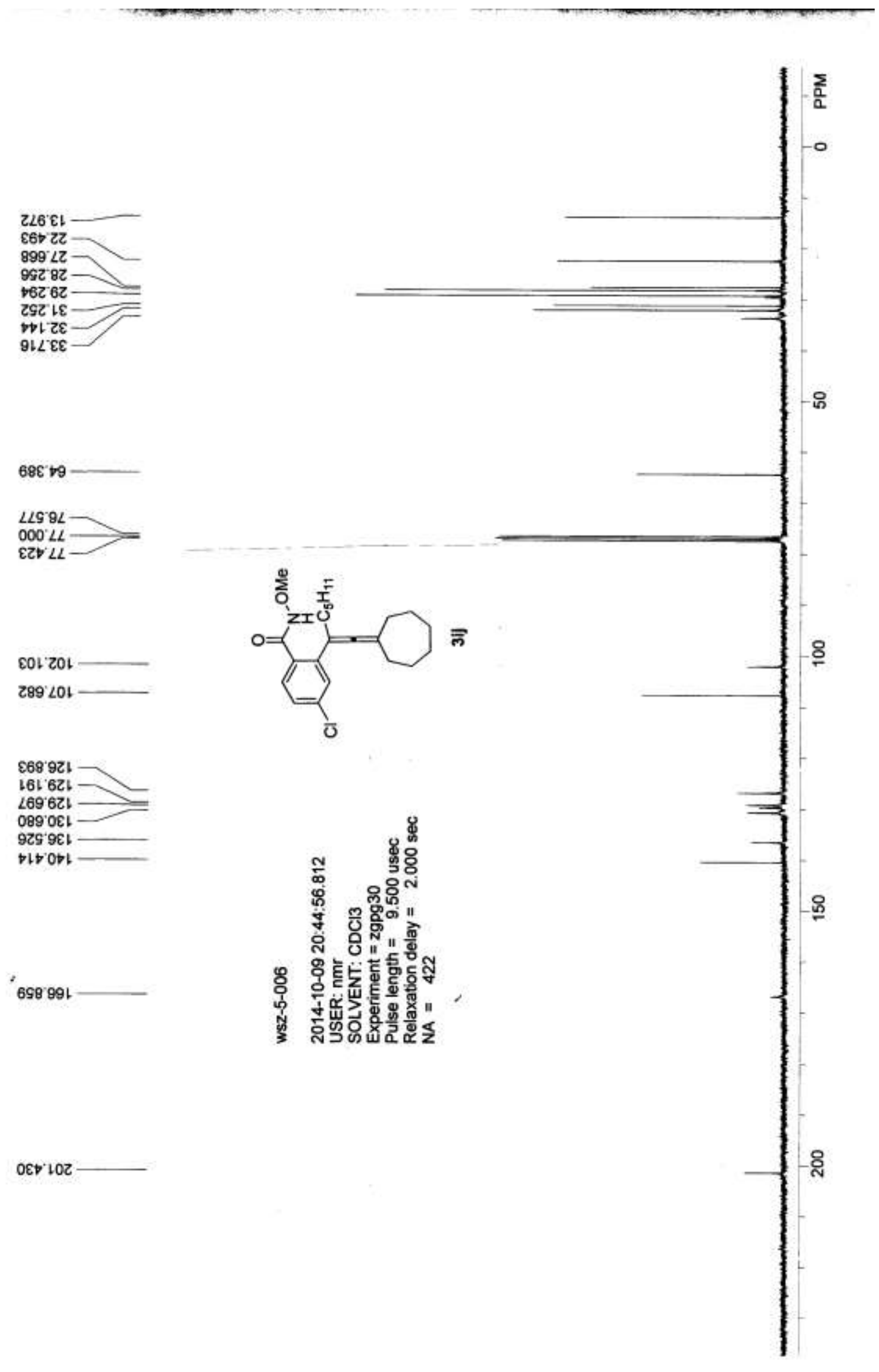

Supplementary Figure 44. ^{13}C NMR (75 MHz, $CDCl_3$) spectrum for 3ij.

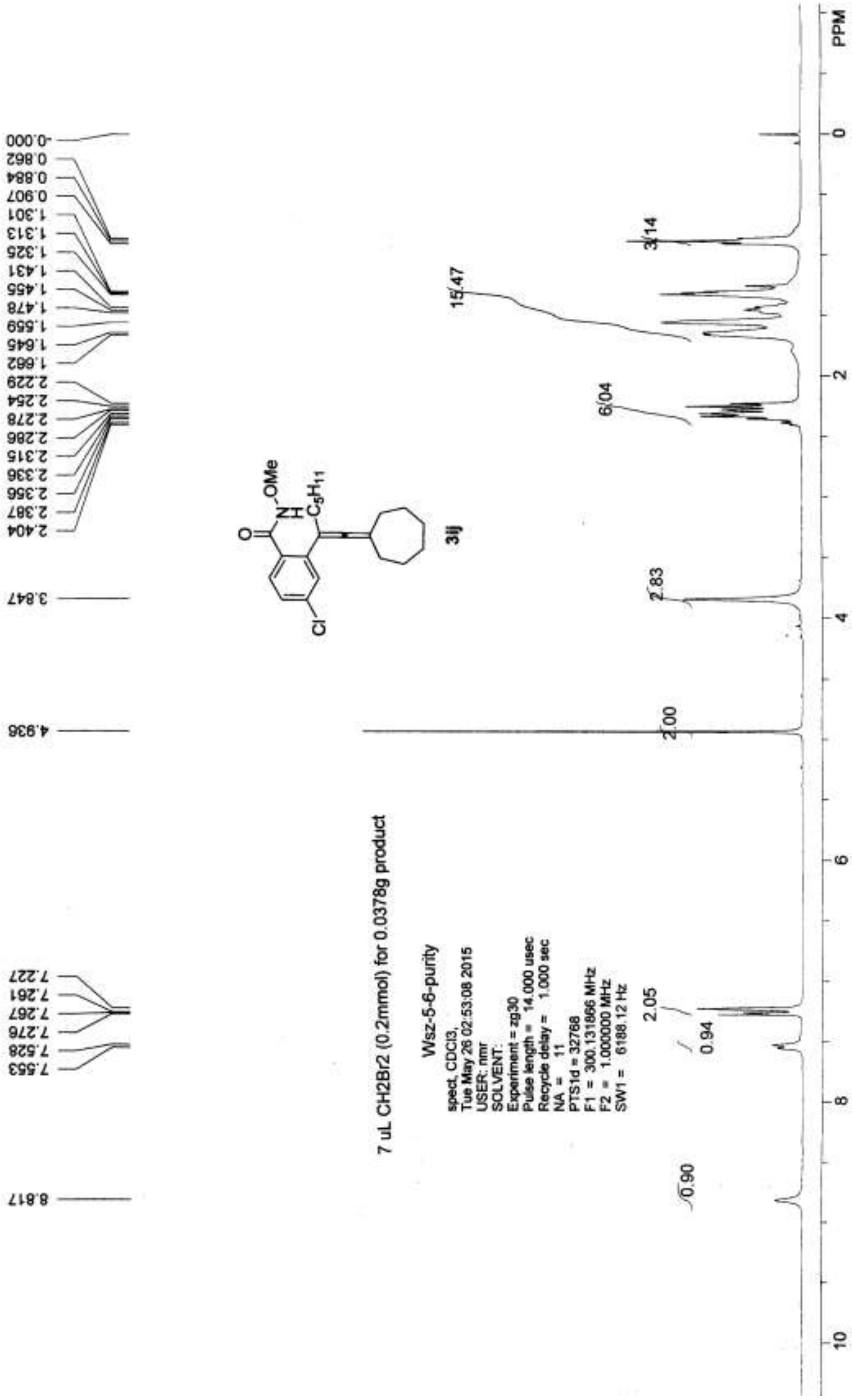

Supplementary Figure 45. ^{1}H NMR (300 MHz, $CDCl_3$) spectrum for the purity of 3ij.

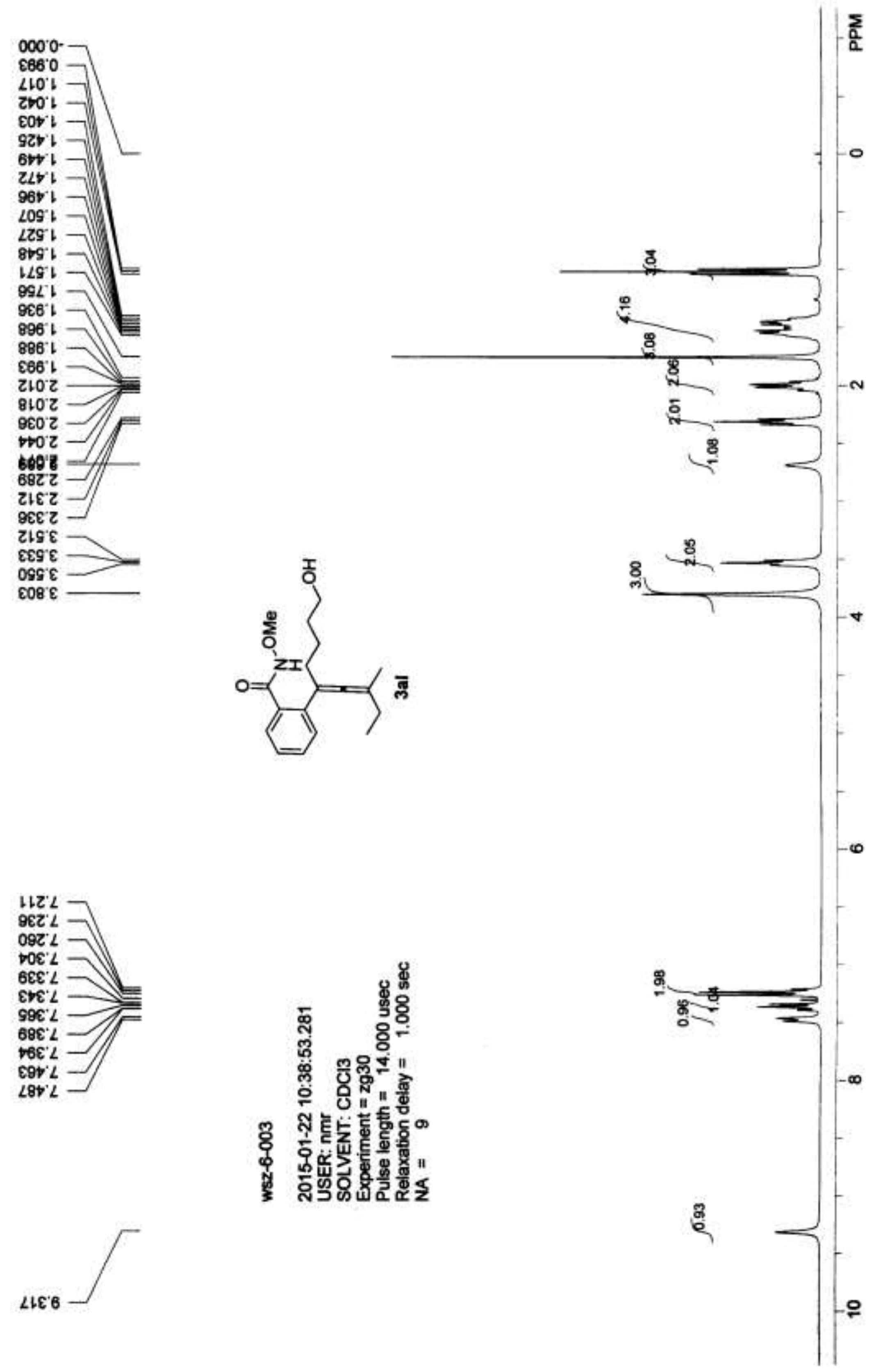

Supplementary Figure 46. **^{1}H NMR (300 MHz, $CDCl_3$) spectrum for 3al.**

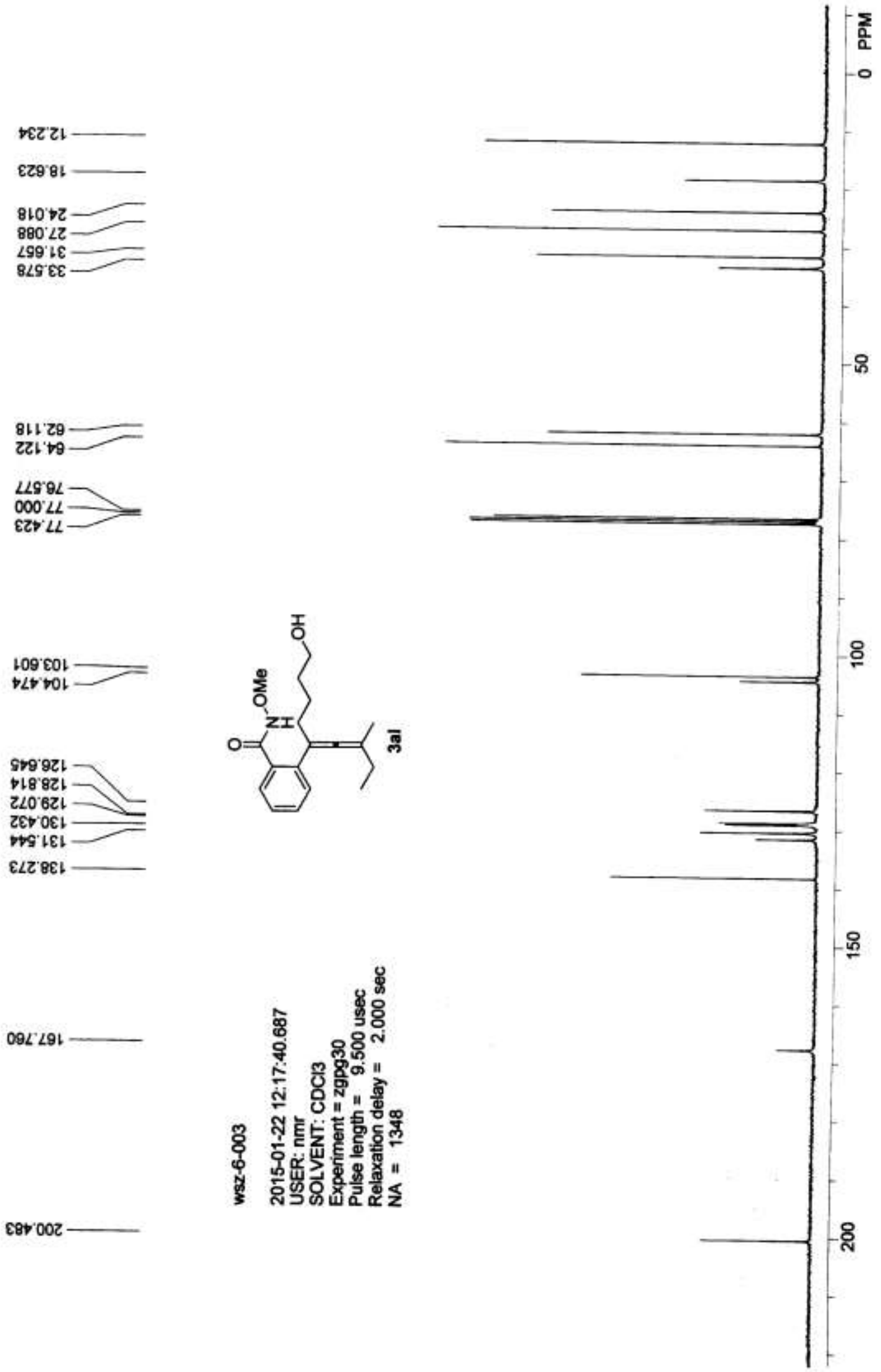

Supplementary Figure 47. ^{13}C NMR (75 MHz, $CDCl_3$) spectrum for 3al.

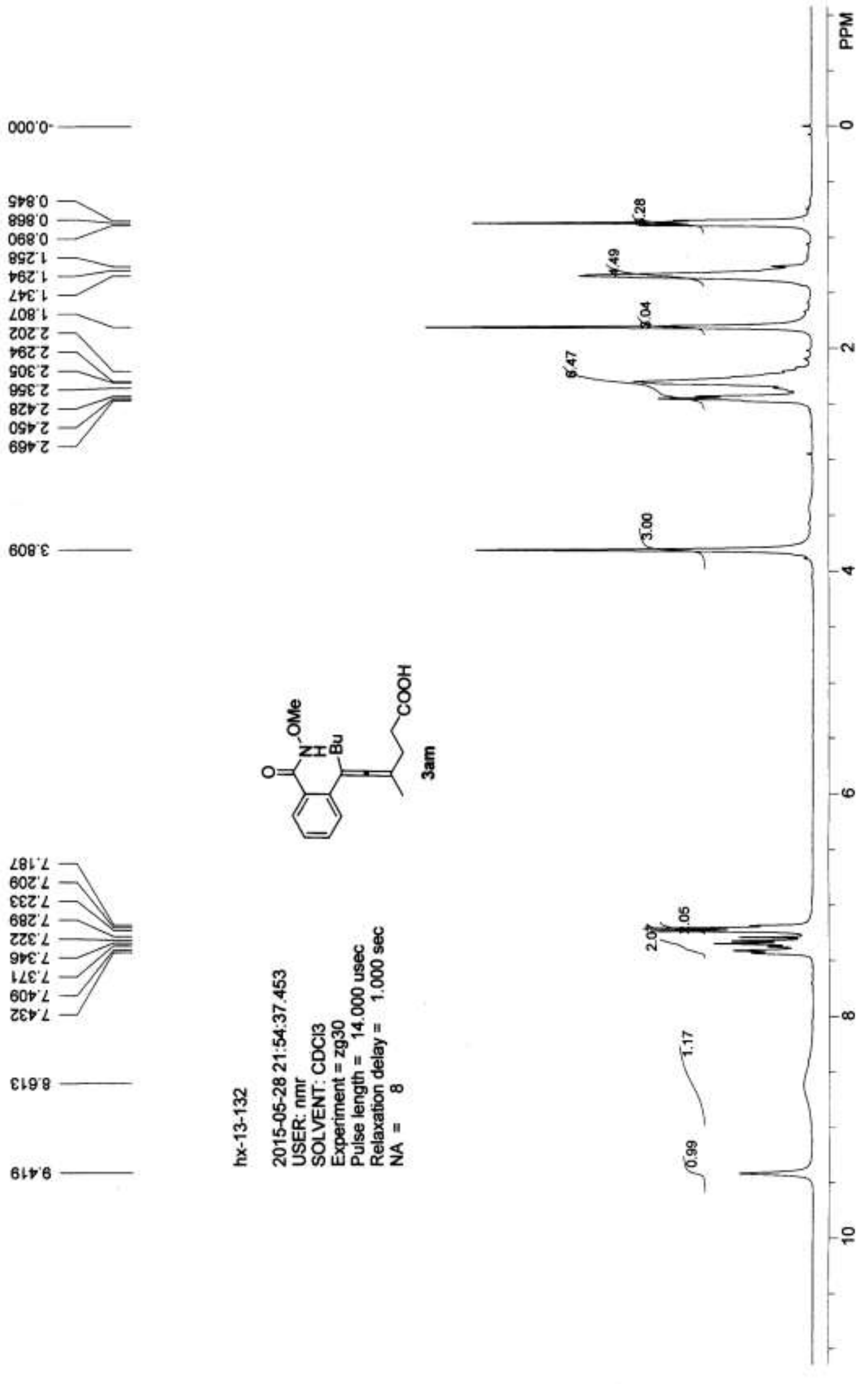

Supplementary Figure 48. **^{1}H NMR (300 MHz, $CDCl_3$) spectrum for 3am.**

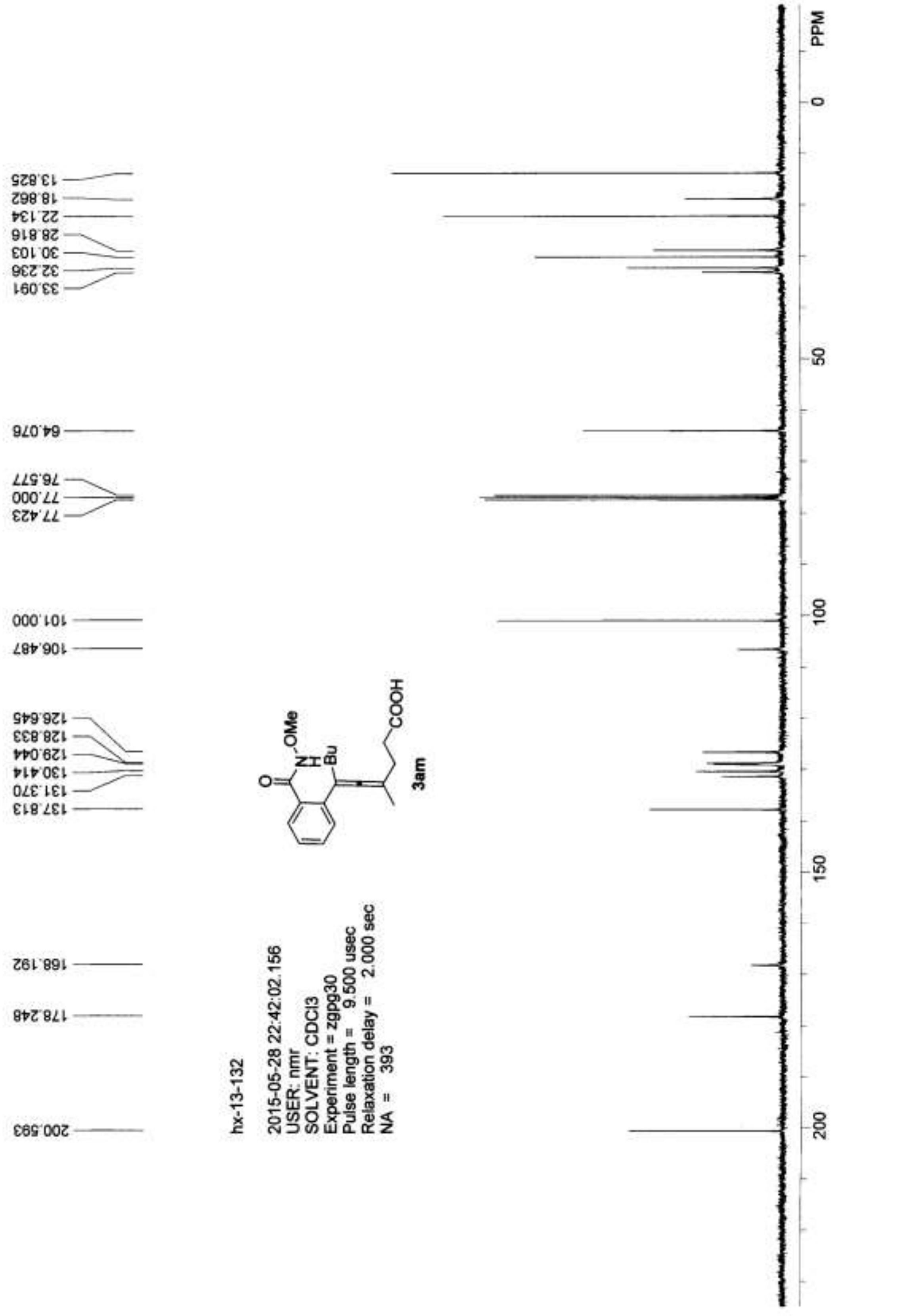

Supplementary Figure 49. ^{13}C NMR (75 MHz, $CDCl_3$) spectrum for 3am.

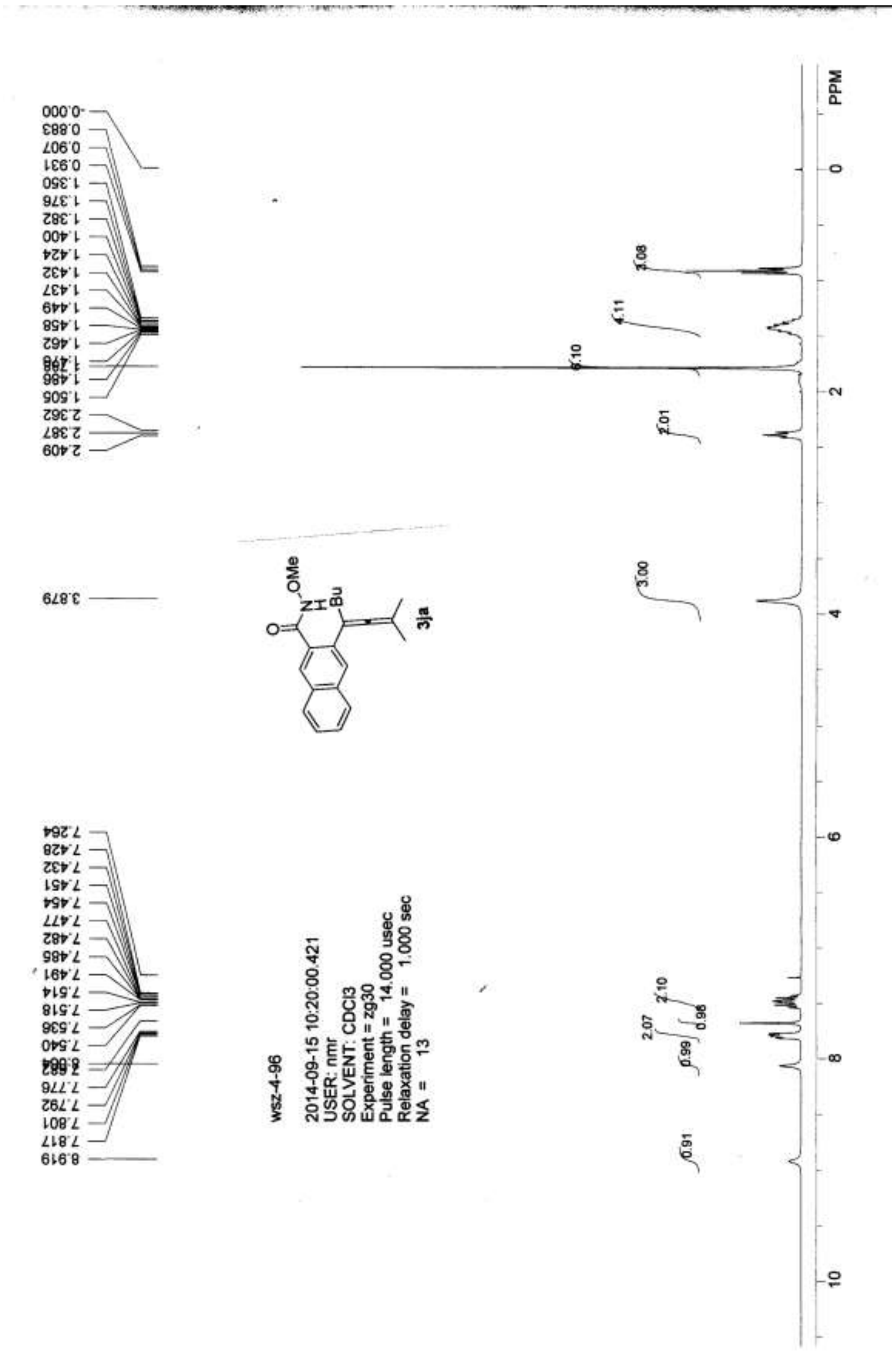

Supplementary Figure 50. ^{1}H NMR (300 MHz, $CDCl_3$) spectrum for 3ja.

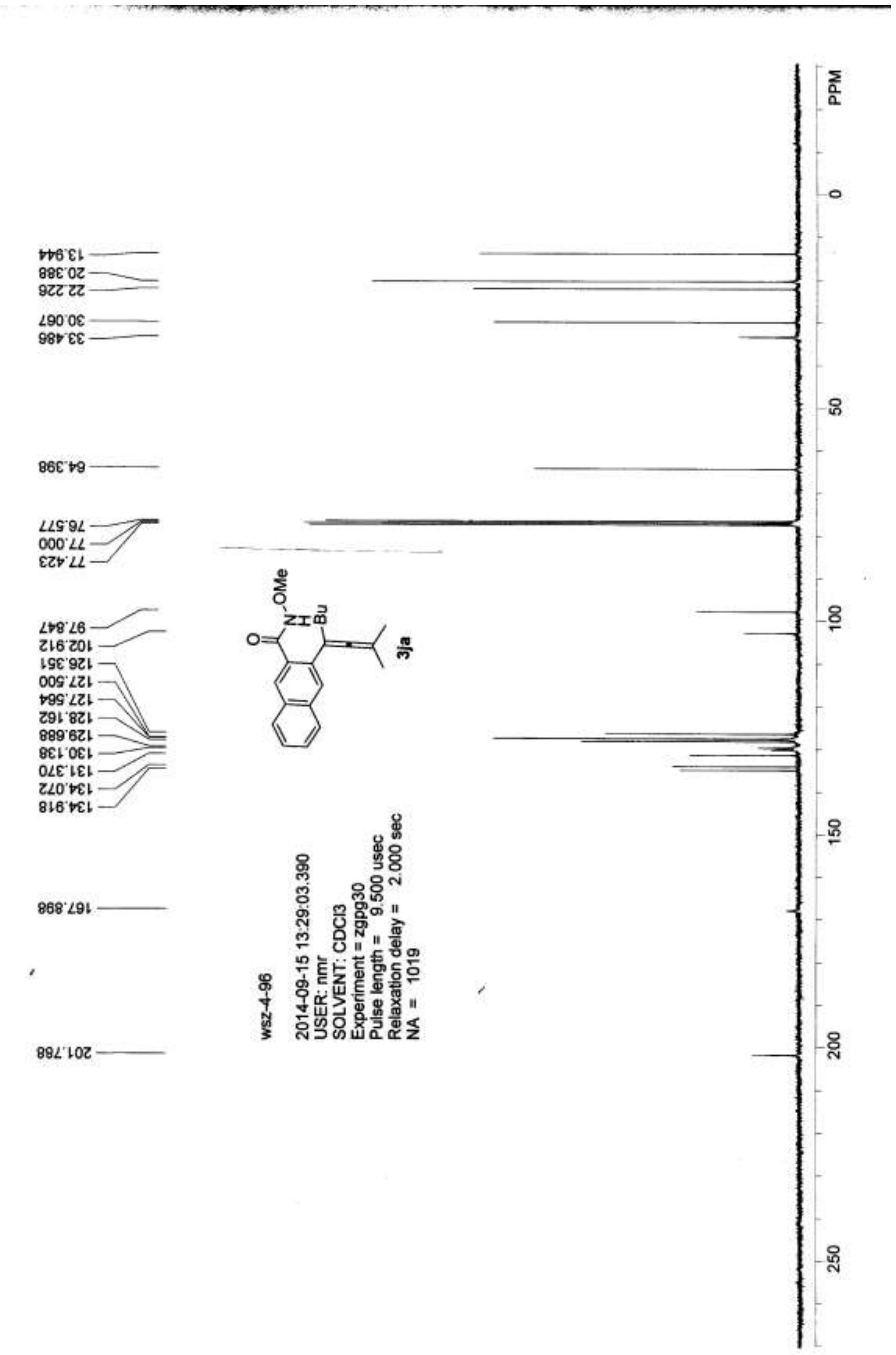

Supplementary Figure 51. ^{13}**C NMR (75 MHz,** $CDCl_3$**) spectrum for 3ja.**

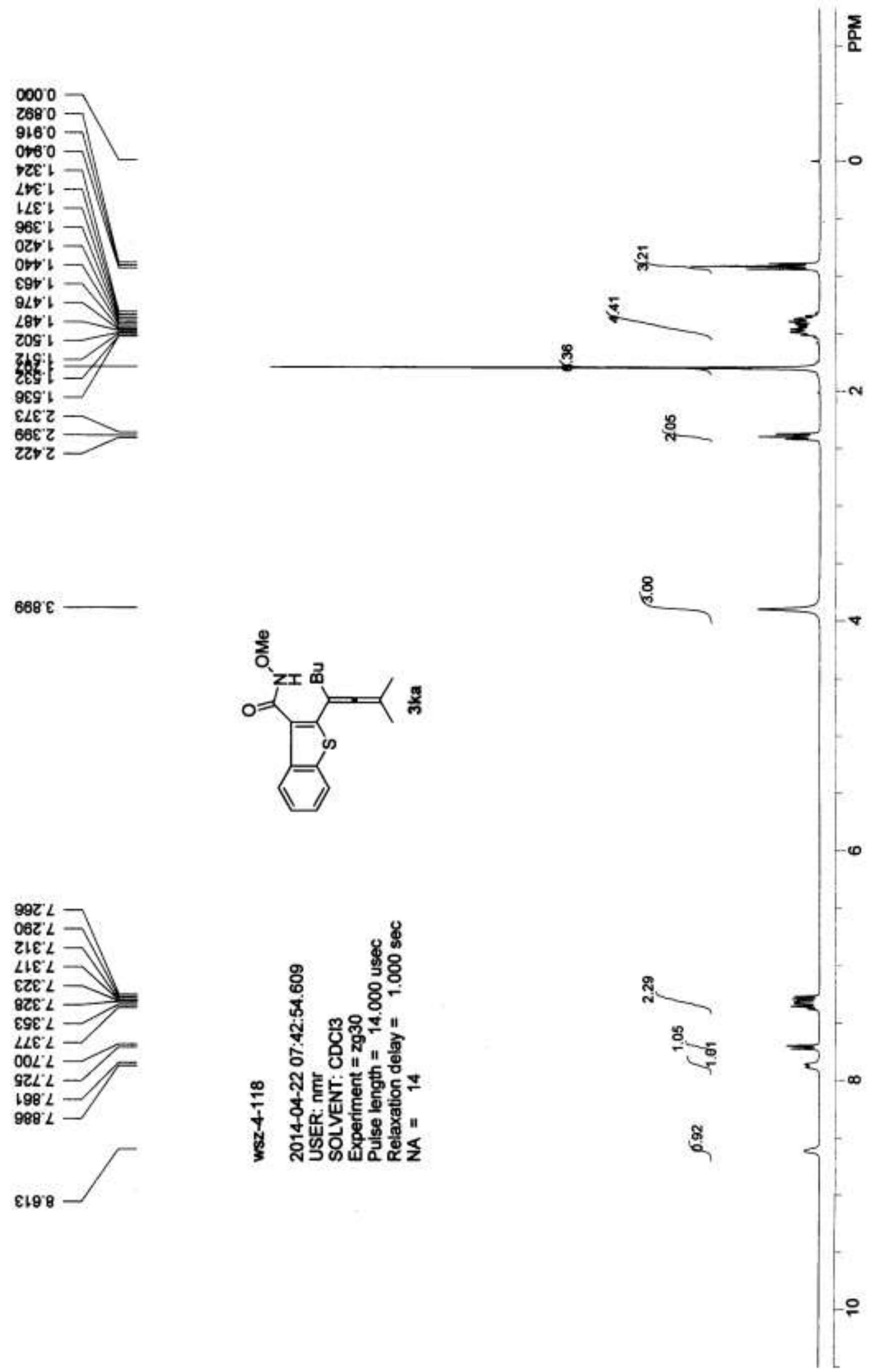

Supplementary Figure 52. ^{1}H NMR (300 MHz, $CDCl_3$) spectrum for 3ka.

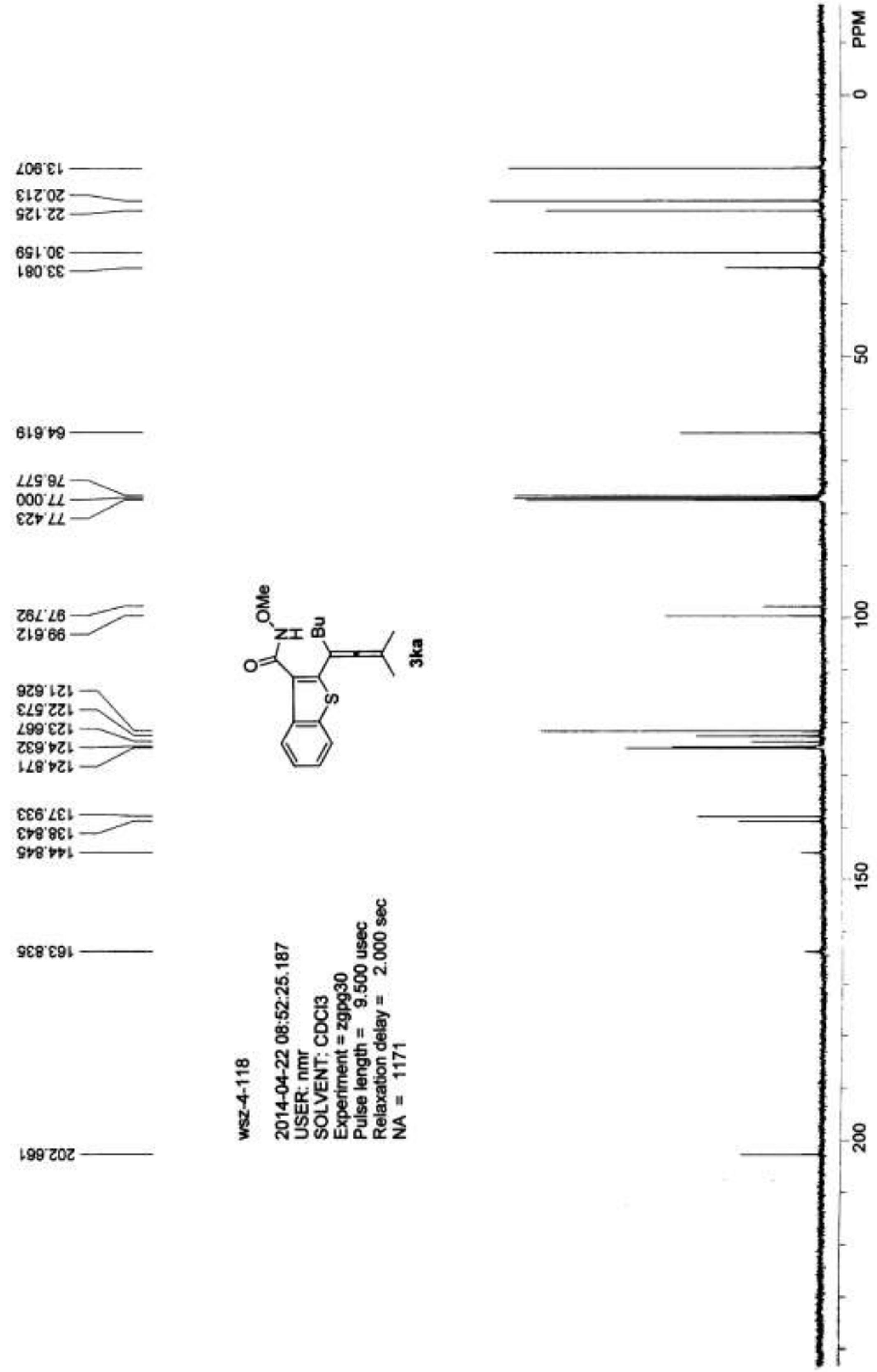

Supplementary Figure 53. ^{13}C NMR (75 MHz, $CDCl_3$) spectrum for 3ka.

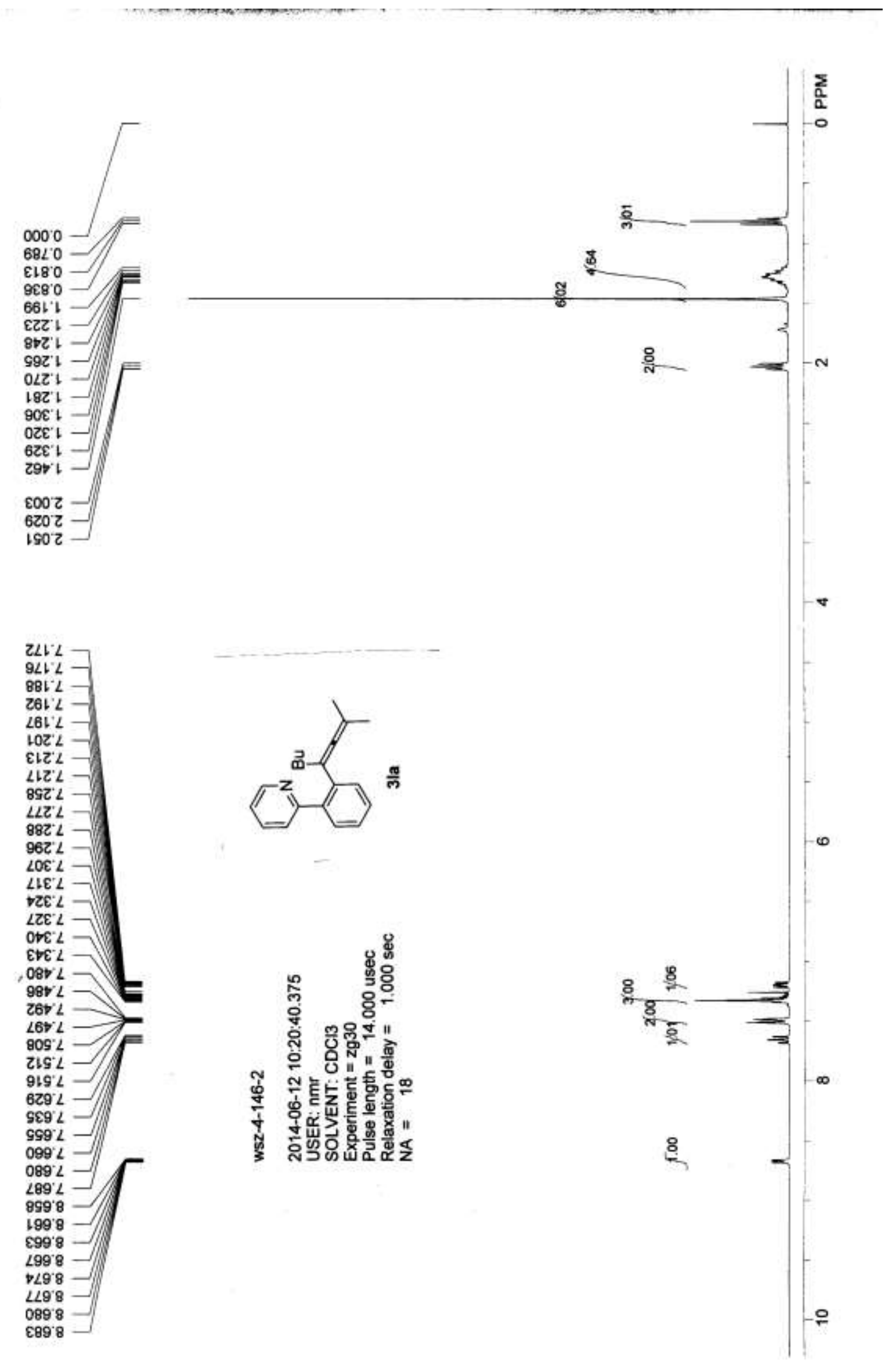

Supplementary Figure 54. ^{1}H NMR (300 MHz, $CDCl_3$) spectrum for 3la.

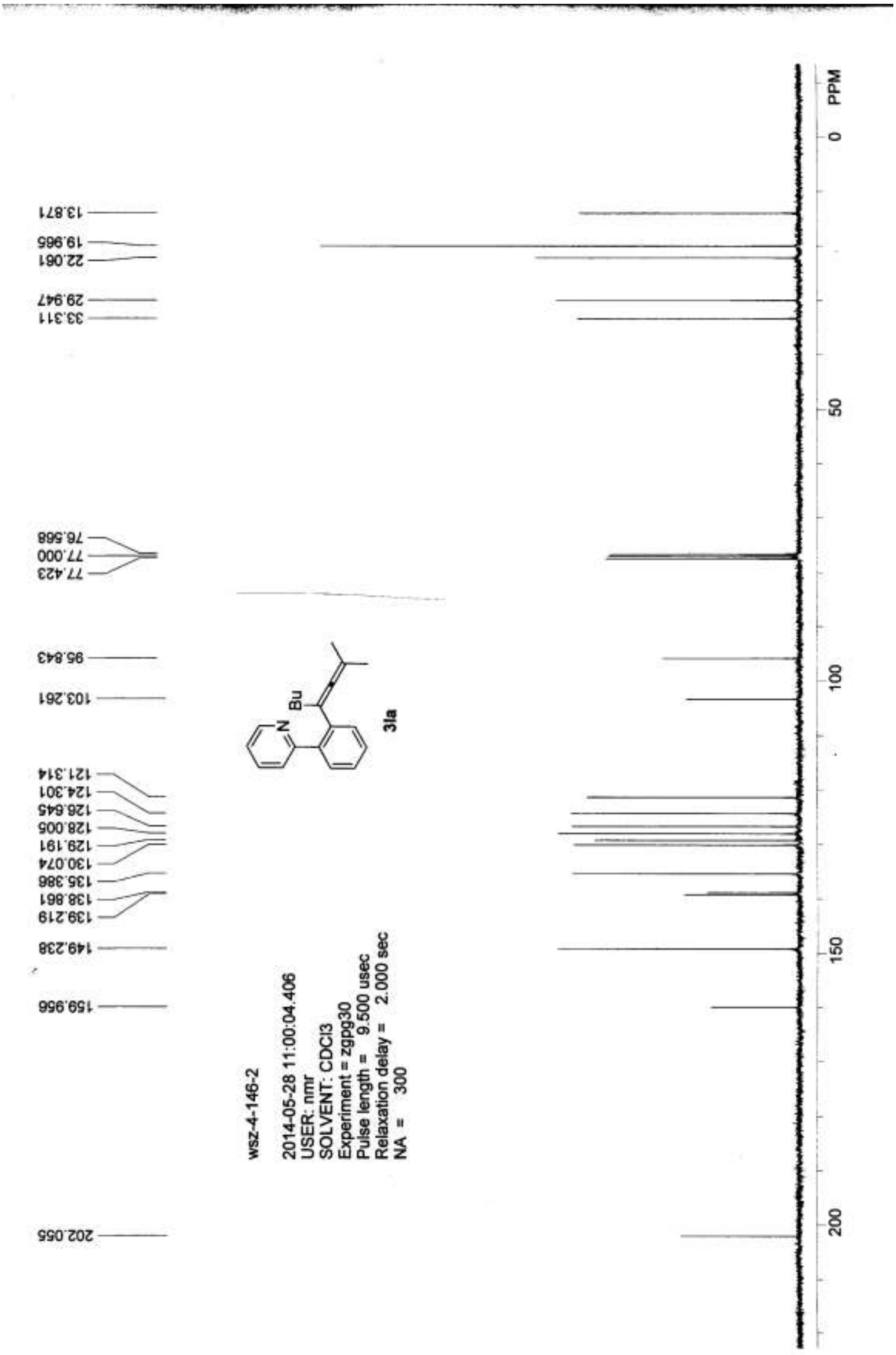

Supplementary Figure 55. ^{13}C NMR (75 MHz, $CDCl_3$) spectrum for 3la.

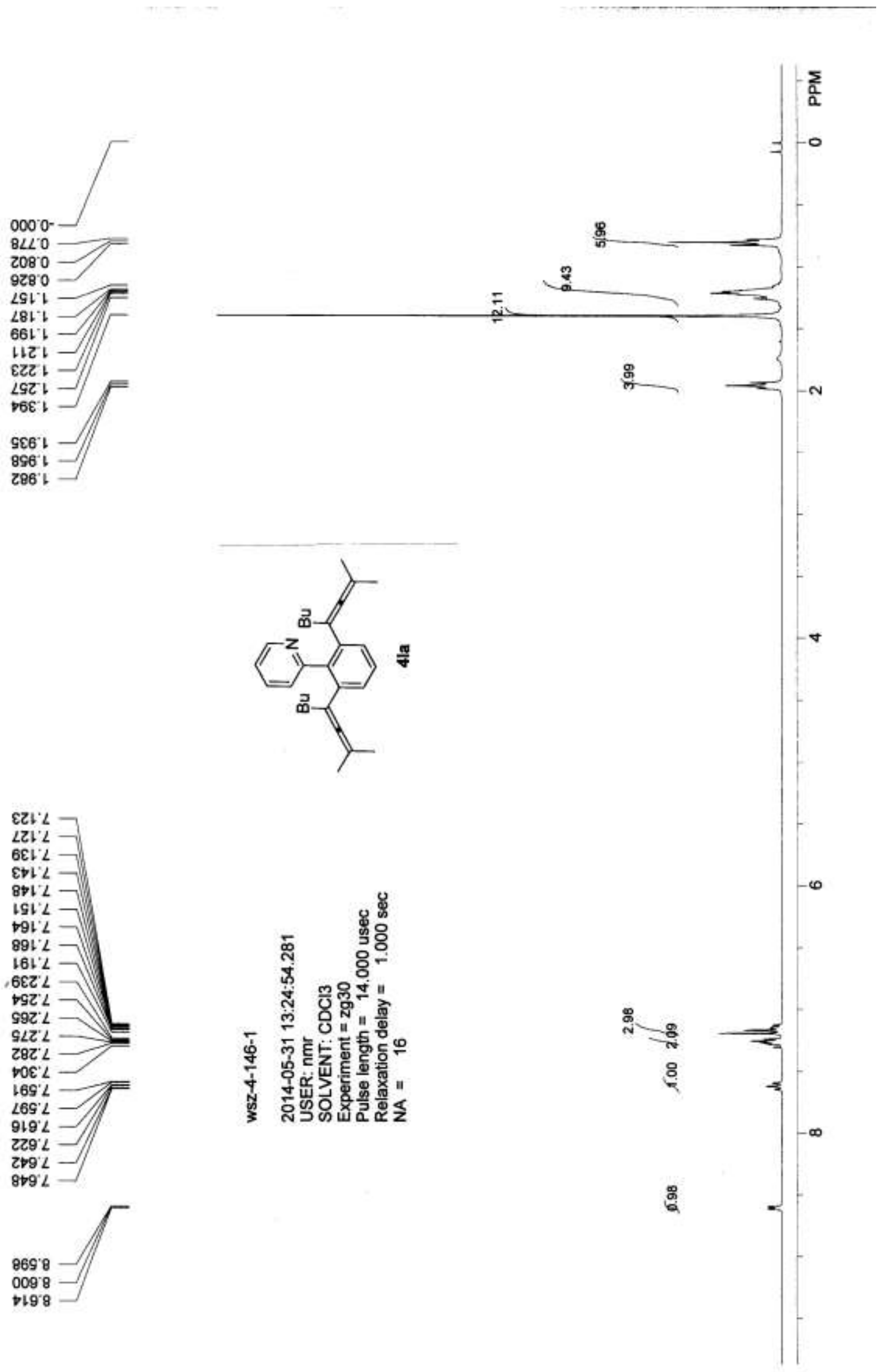

Supplementary Figure 56. ^{1}H NMR (300 MHz, $CDCl_3$) spectrum for 4la.

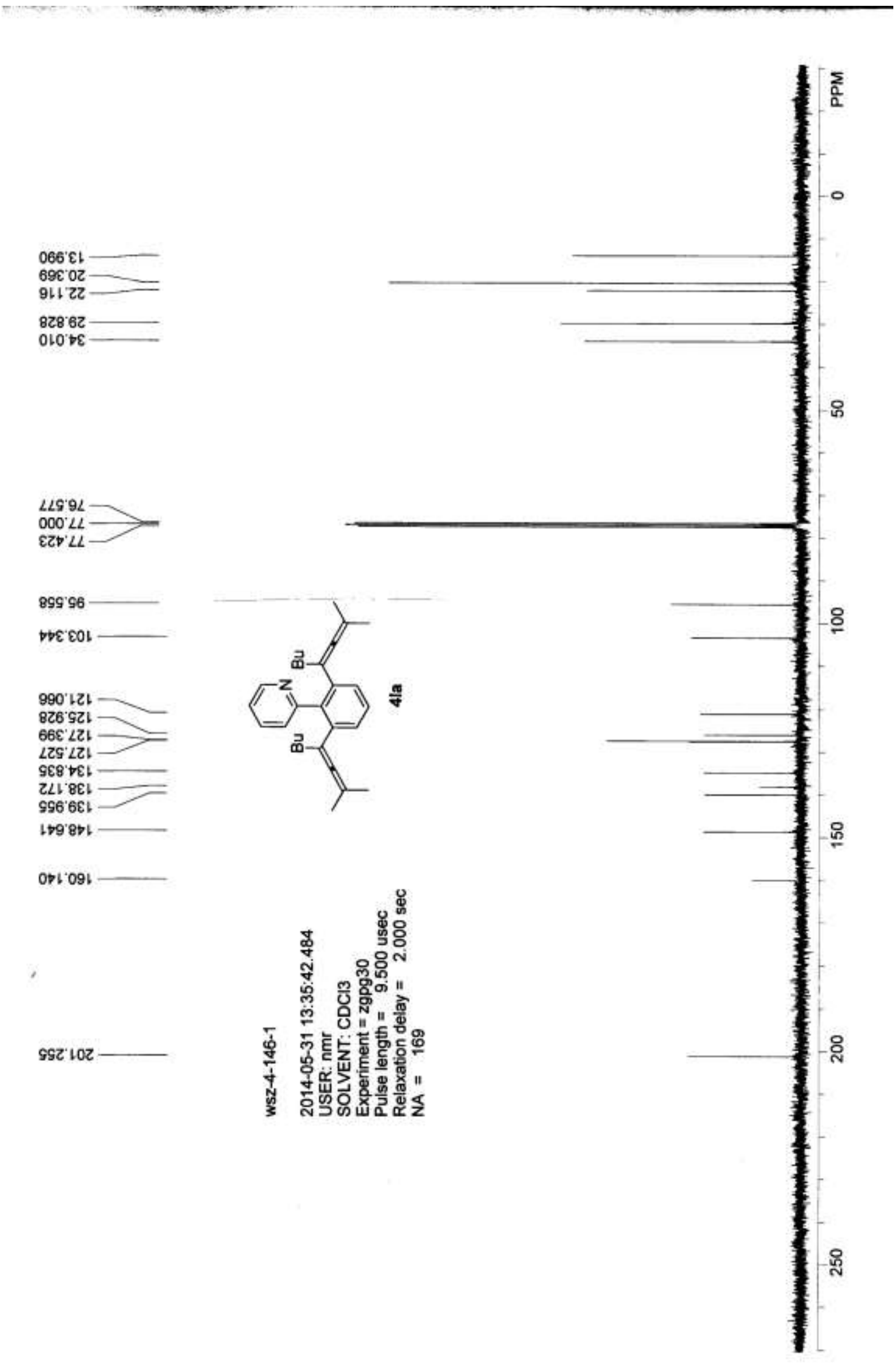

Supplementary Figure 57. ^{13}C NMR (75 MHz, $CDCl_3$) spectrum for 4la.

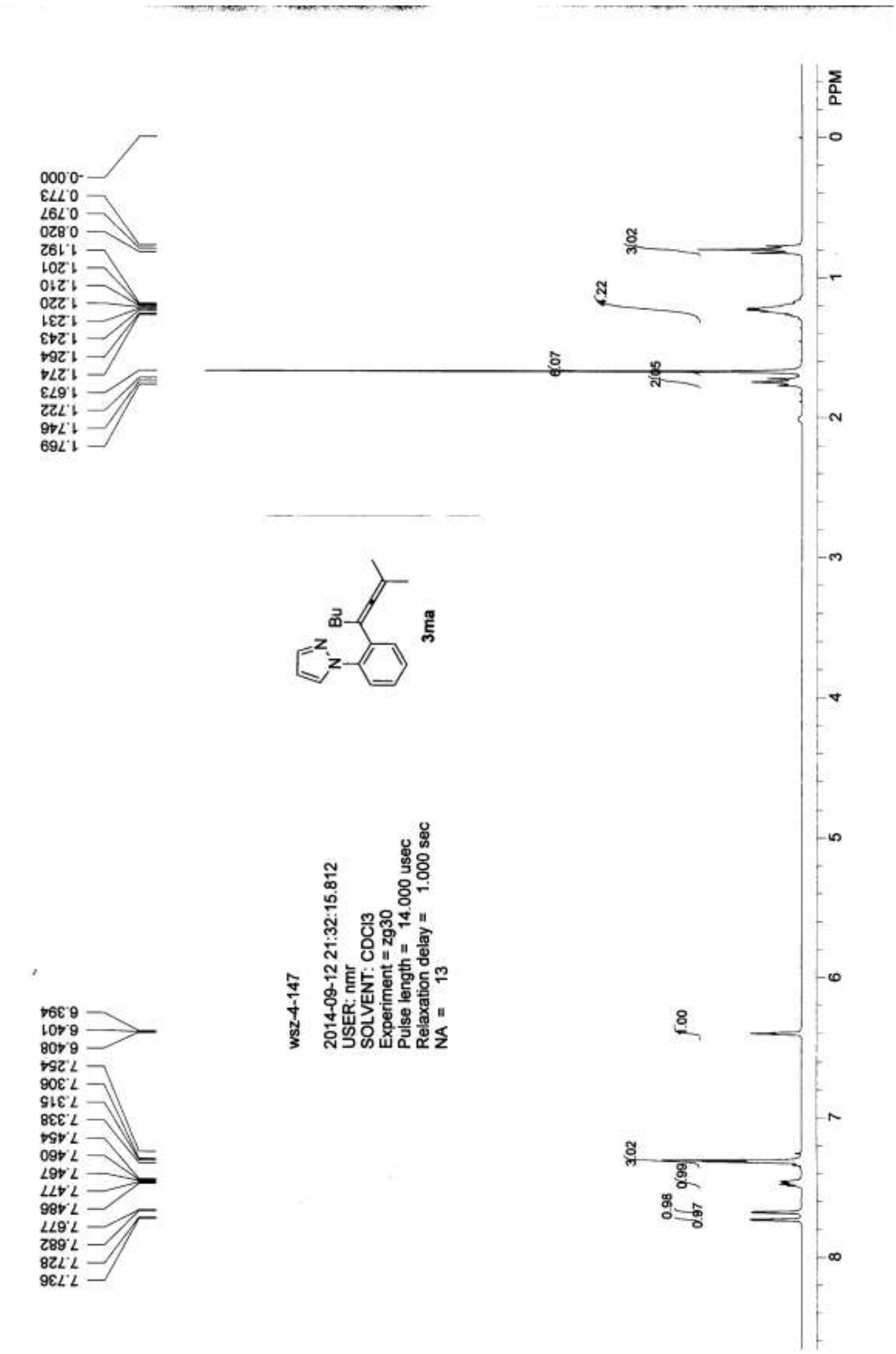

Supplementary Figure 58. ^{1}H NMR (300 MHz, $CDCl_3$) spectrum for 3ma.

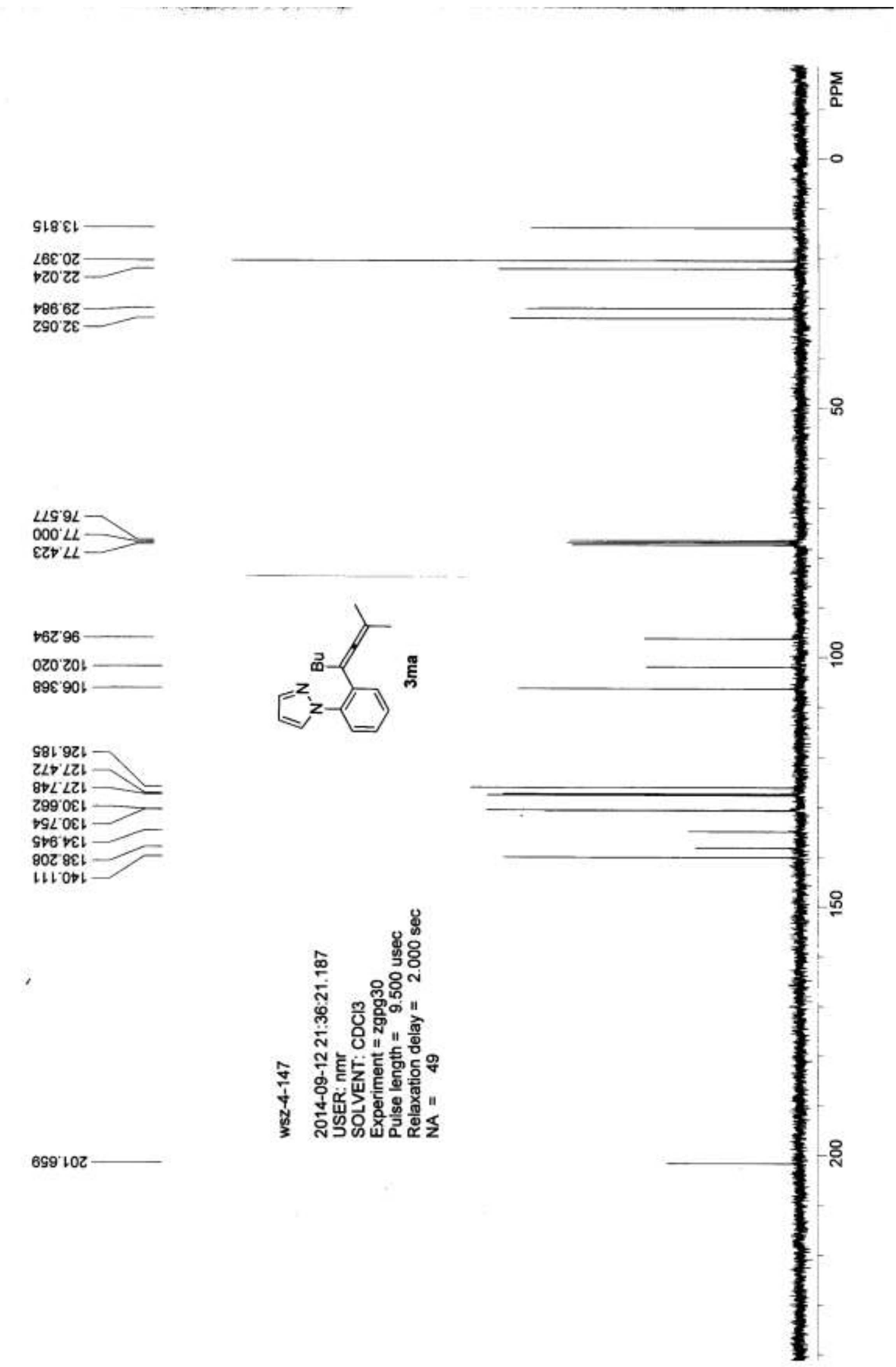

Supplementary Figure 59. ^{13}**C NMR (75 MHz, CDCl$_3$) spectrum for 3ma.**

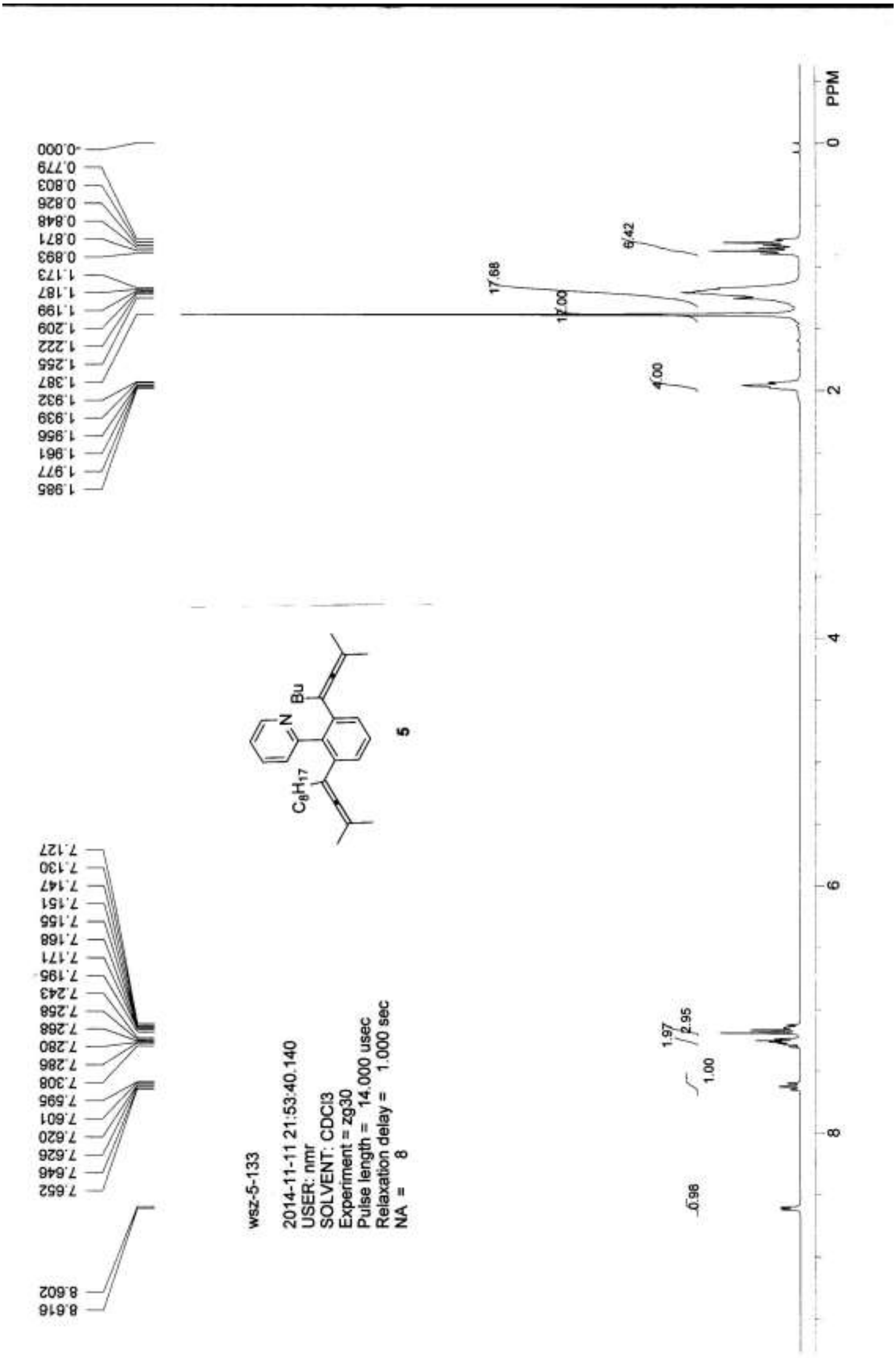

Supplementary Figure 60. ^{1}H NMR (300 MHz, $CDCl_3$) spectrum for 5.

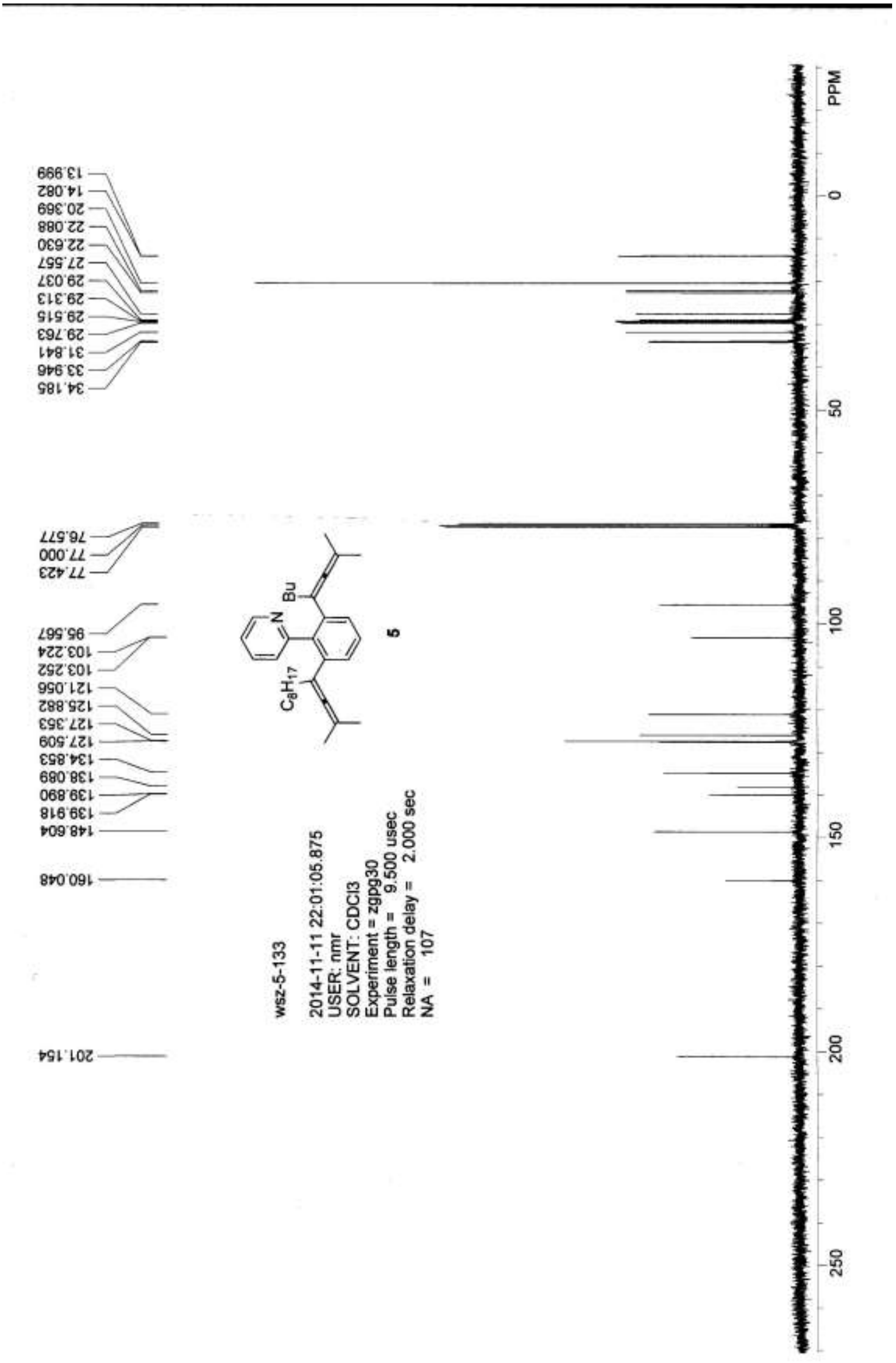

Supplementary Figure 61. ^{13}**C NMR (75 MHz, CDCl$_3$) spectrum for 5.**

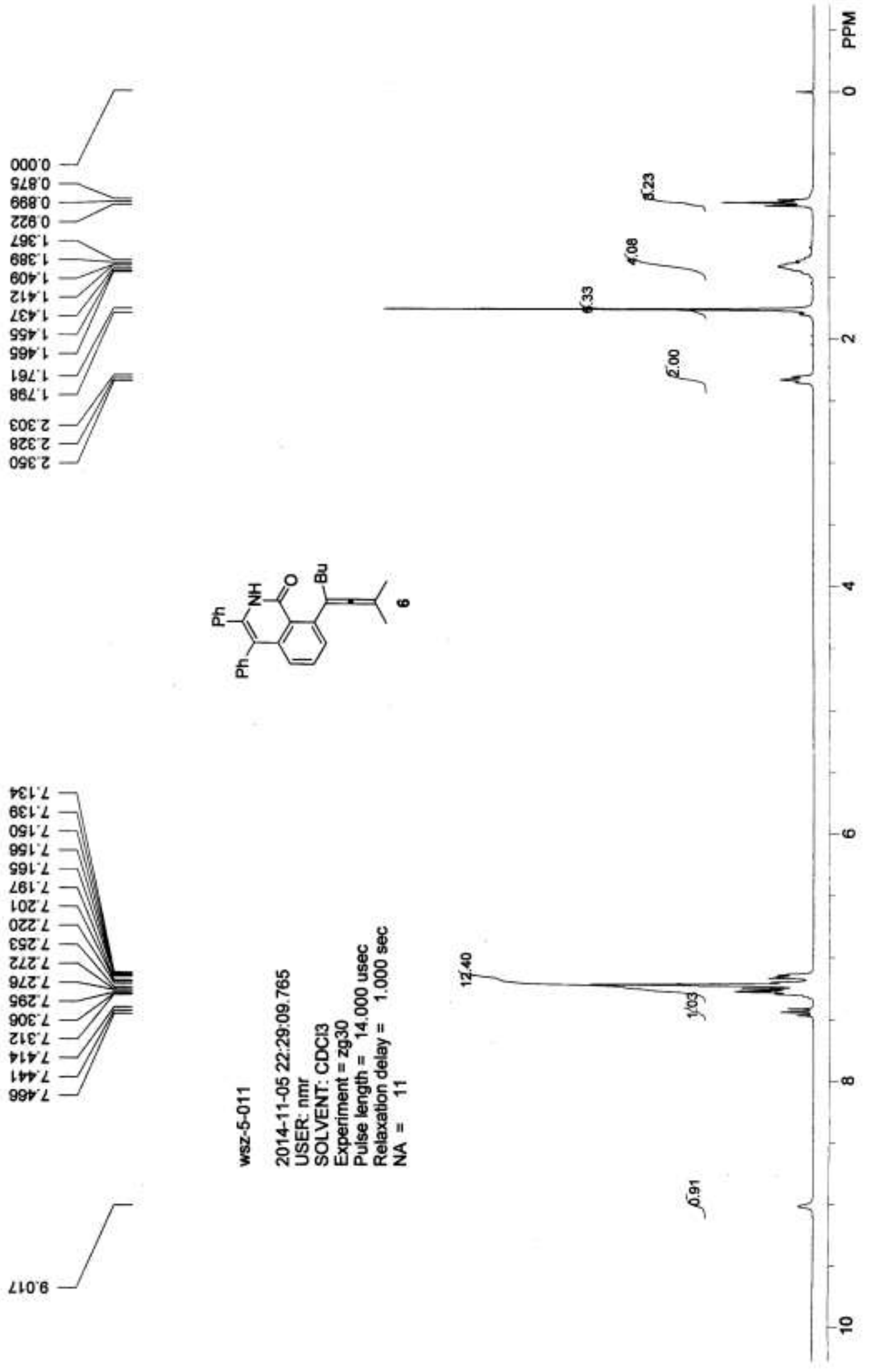

Supplementary Figure 62. ^{1}H NMR (300 MHz, $CDCl_3$) spectrum for 6.

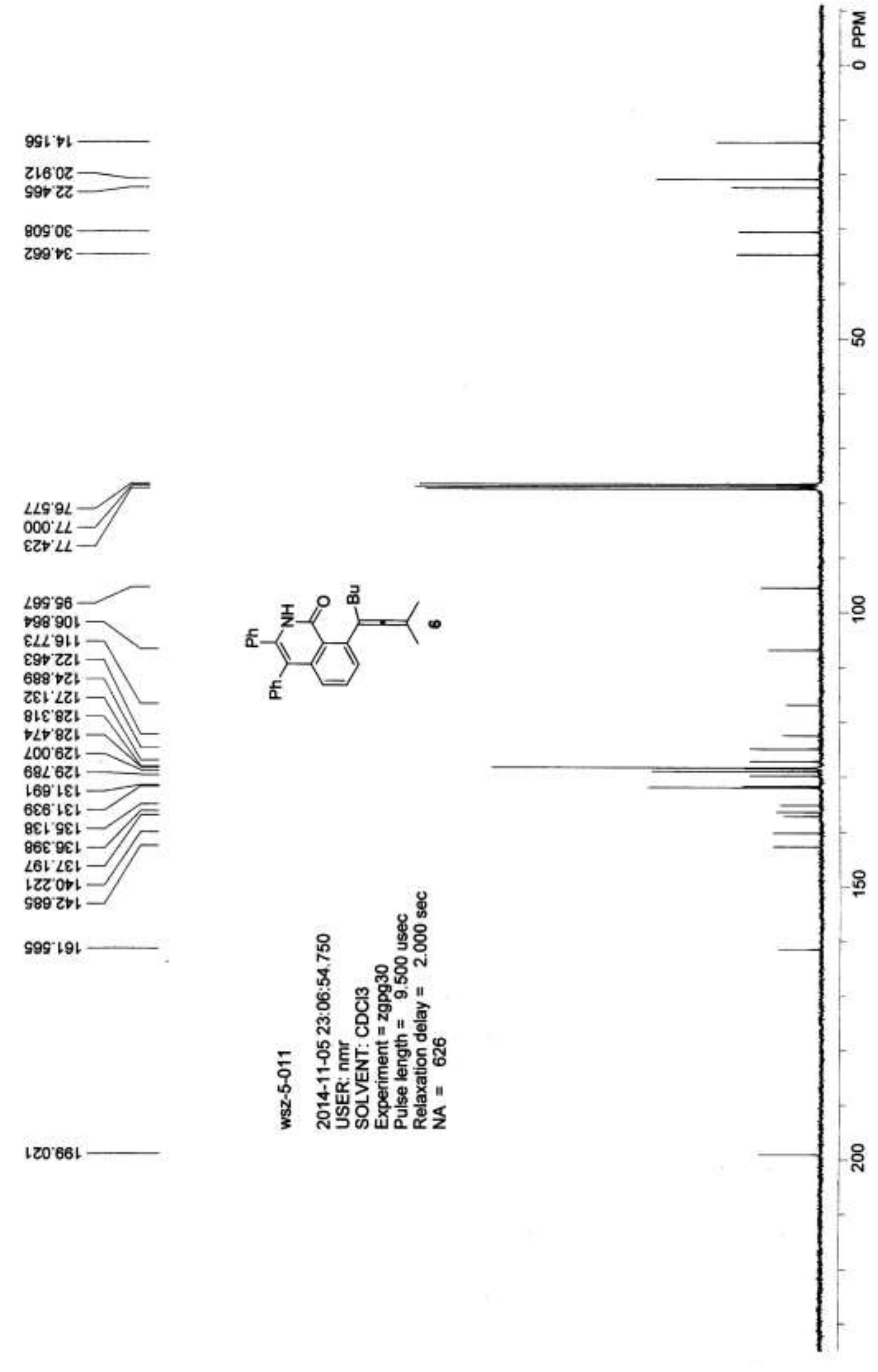

Supplementary Figure 63. ^{13}C NMR (75 MHz, $CDCl_3$) spectrum for 6.

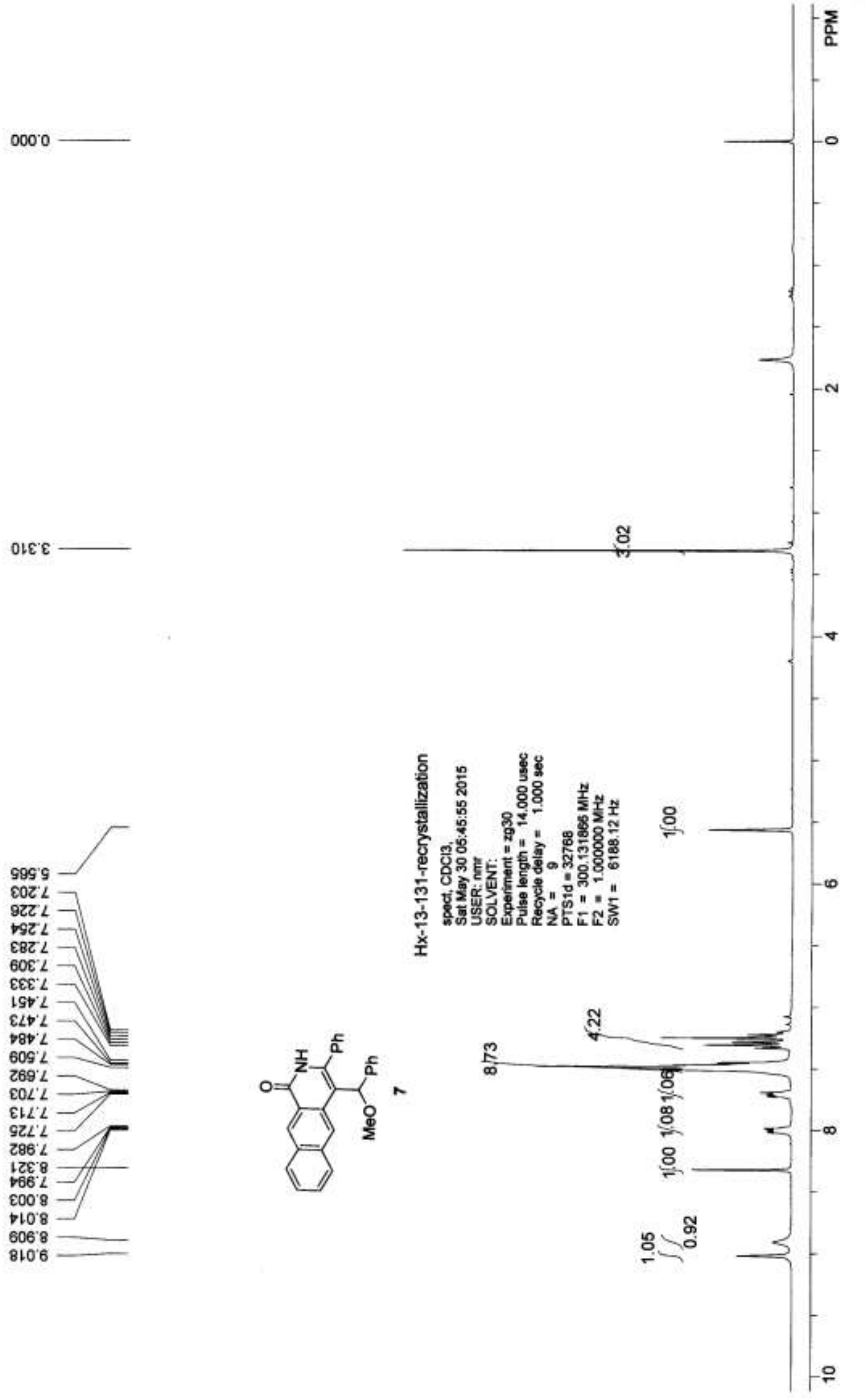

Supplementary Figure 64. ^{1}H NMR (300 MHz, $CDCl_3$) spectrum for 7.

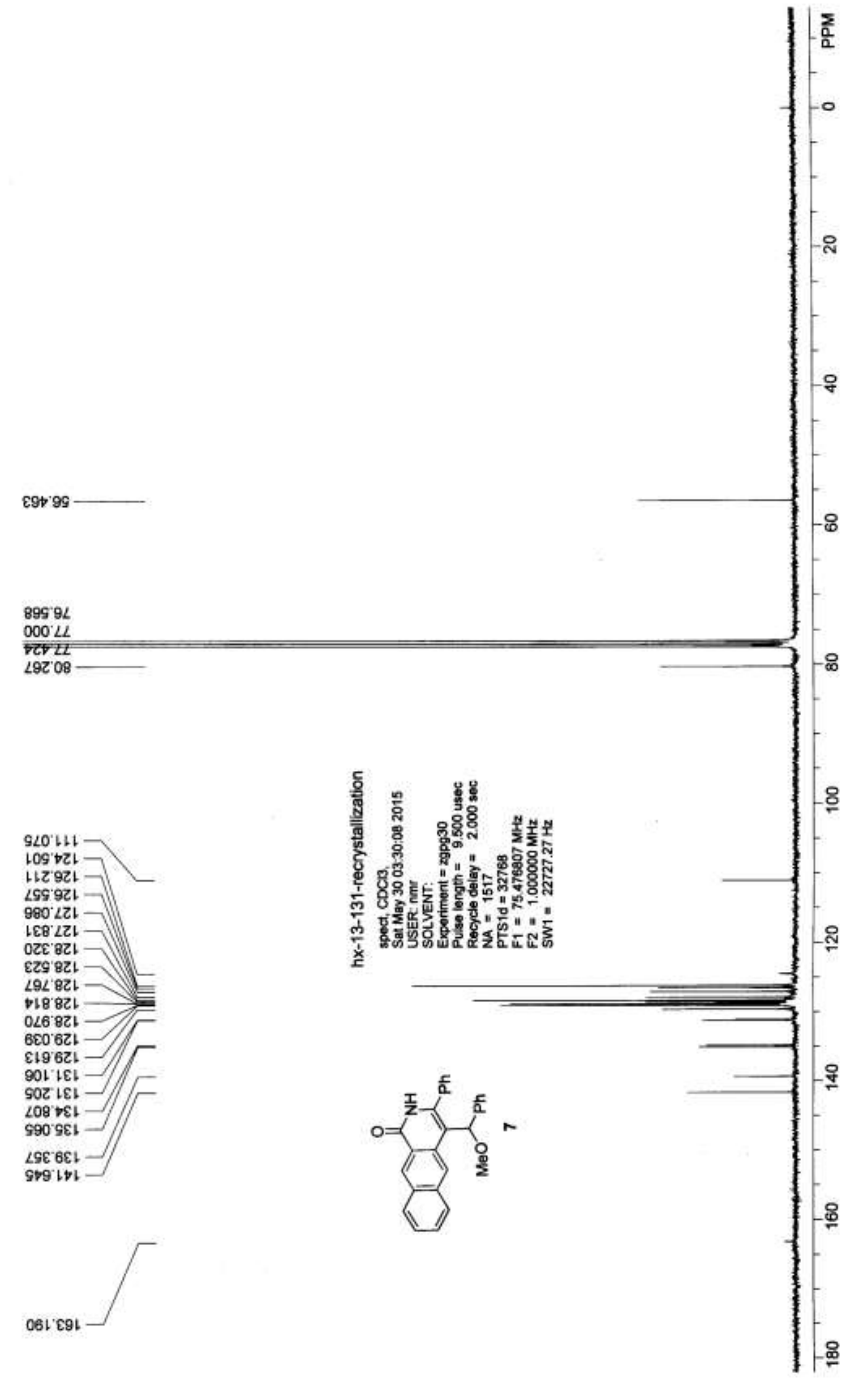

Supplementary Figure 65. ^{13}C **NMR (75 MHz,** $CDCl_3$**) spectrum for 7.**

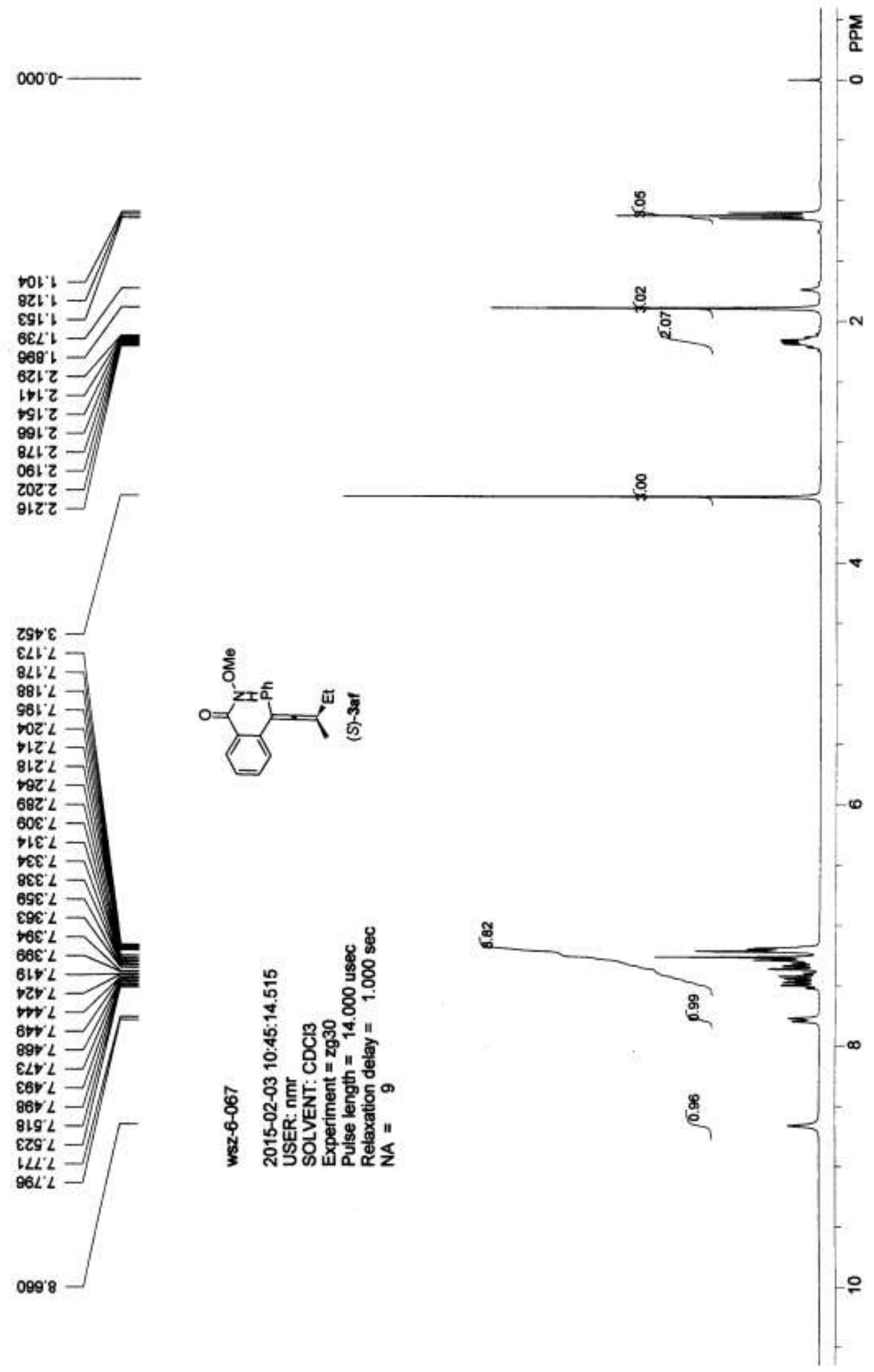

Supplementary Figure 66. ^{1}**H NMR (300 MHz, CDCl$_3$) spectrum for *S*-3af.**

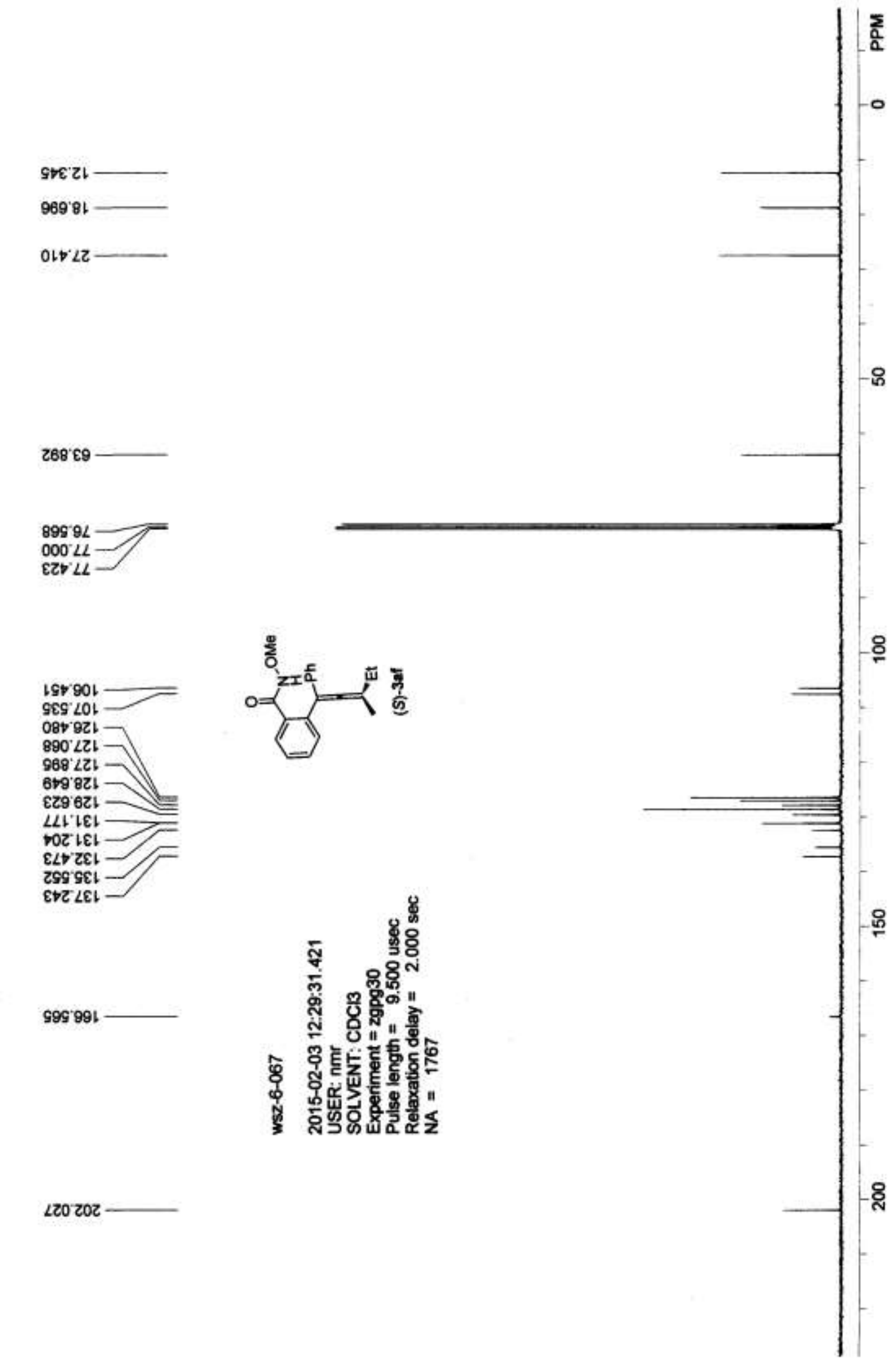

Supplementary Figure 67. ^{13}**C NMR (75 MHz, CDCl$_3$) spectrum for *S*-3af.**

N2000 数据工作站

wsz-6-67

实验单位：zju
实验时间：2015-01-29, 2:37:58
谱图文件:D:\浙大智达\N2000\样品\B0725.org

实验者：wsz
报告时间：2015-01-29, 3:13:29
积分方法:面积归一法

实验内容简介：
AD-H, n-hexane/i-PrOH = 10/1, 207 nm, 1.0 ml/min

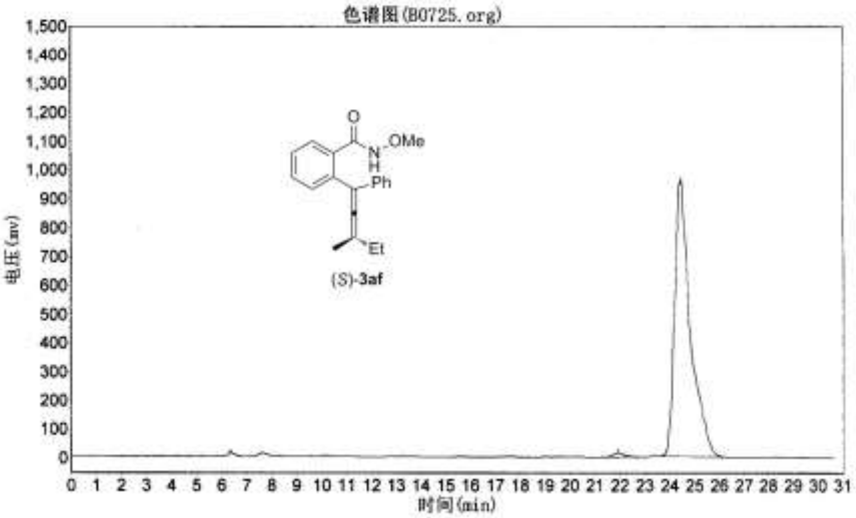

分析结果表

峰号	峰名	保留时间	峰高	峰面积	含量
1		21.910	13709.883	438647.063	1.0397
2		24.457	957278.375	41749156.000	98.9603
总计			970988.258	42187803.063	100.0000

2015-01-29 浙江大学智能信息研究所

Supplementary Figure 68. HPLC spectrum for *S*-3af.

N2000 数据工作站

wsz-5-169

实验单位：zju
实验时间：2015-01-29,6:08:56
谱图文件:D:\浙大智达\N2000\样品\B0731.org

实验者：wsz
报告时间：2015-01-29,6:40:40
积分方法:面积归一法

实验内容简介：
AD-H, n-hexane/i-PrOH = 10/1, 207 nm, 1.0 ml/min

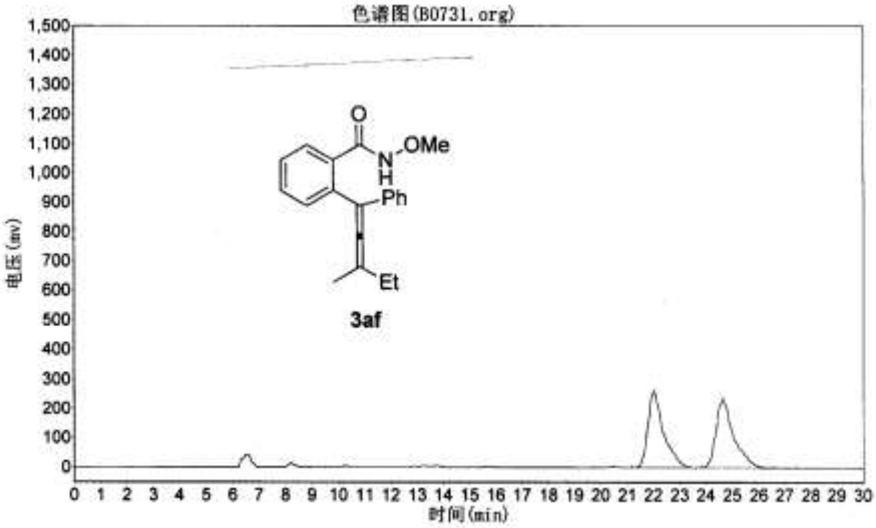

分析结果表

峰号	峰名	保留时间	峰高	峰面积	含量
1		22.040	253891.031	10589781.000	49.9524
2		24.645	227939.688	10609971.000	50.0476
总计			481830.719	21199752.000	100.0000

2015-01-29　　浙江大学智能信息研究所

Supplementary Figure 69. HPLC spectrum for 3af.

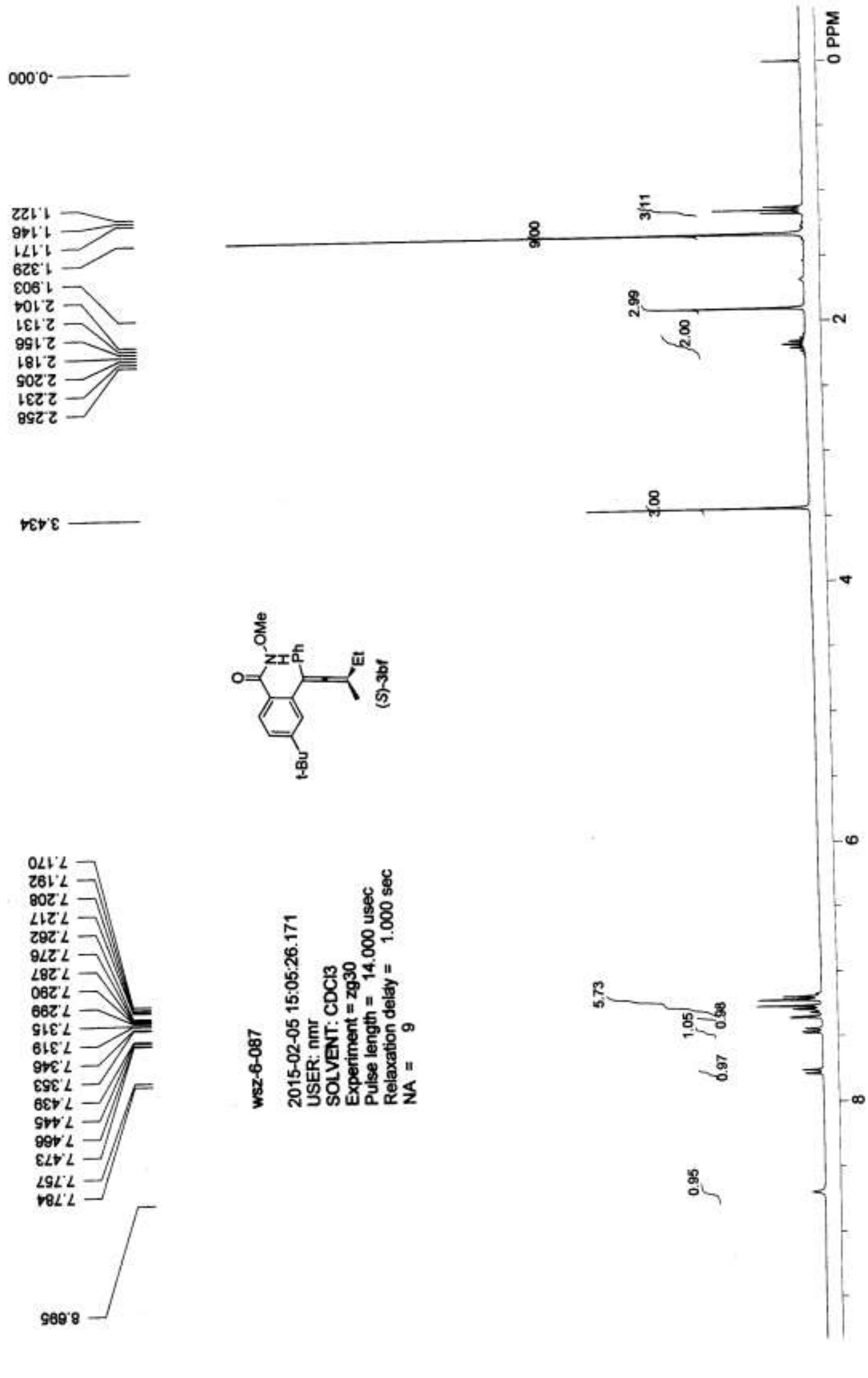

Supplementary Figure 70. ^{1}**H NMR (300 MHz, CDCl$_3$) spectrum for** ***S*-3bf.**

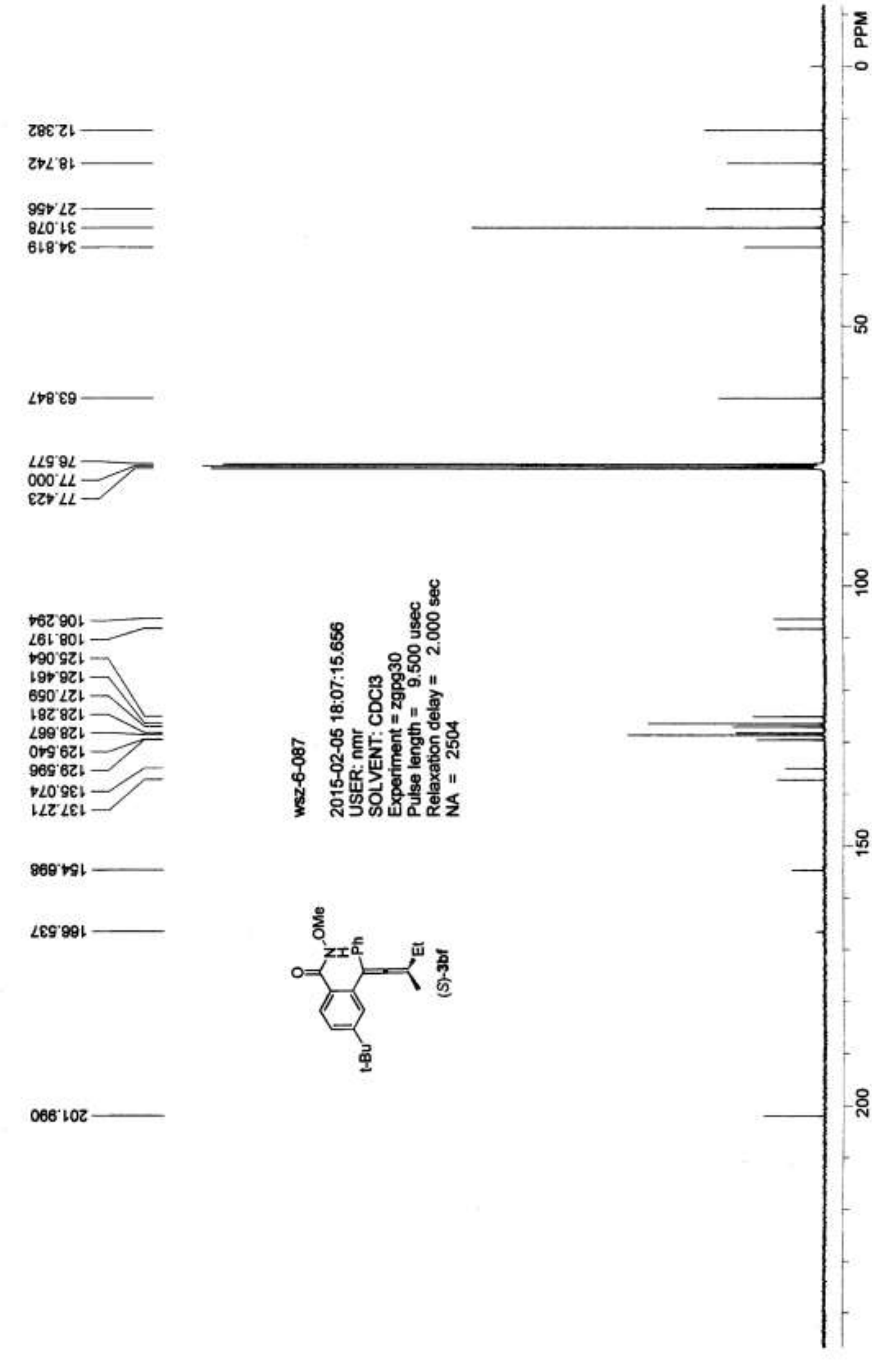

Supplementary Figure 71. ^{13}**C NMR (75 MHz, CDCl$_3$) spectrum for *S*-3bf.**

N2000 数据工作站

wsz-6-87

实验单位：zju
实验时间：2015-02-04, 23:15:41
谱图文件：D:\浙大智达\N2000\样品\B0766.org

实验者：wsz
报告时间：2015-02-04, 23:50:31
积分方法：面积归一法

实验内容简介：
AD-H, n-hexane/i-PrOH = 10/1, 207 nm, 0.8 ml/min

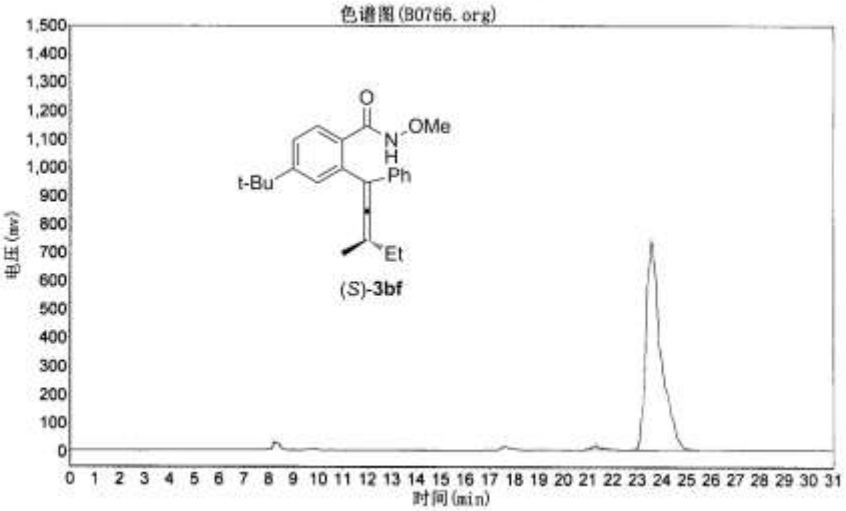

分析结果表

峰号	峰名	保留时间	峰高	峰面积	含量
1		21.307	11484.322	365238.688	1.1728
2		23.565	728824.063	30777076.000	98.8272
总计			740308.385	31142314.688	100.0000

2015-02-04　　浙江大学智能信息研究所

Supplementary Figure 72. HPLC spectrum for *S*-3bf.

N2000 数据工作站

wsz-6-84

实验单位: zju
实验时间: 2015-02-04, 18:12:39
谱图文件:D:\浙大智达\N2000\样品\B0758.org

实验者: wsz
报告时间: 2015-02-04, 19:34:22
积分方法:面积归一法

实验内容简介:
AD-H, n-hexane/i-PrOH = 10/1, 207 nm, 0.8 ml/min

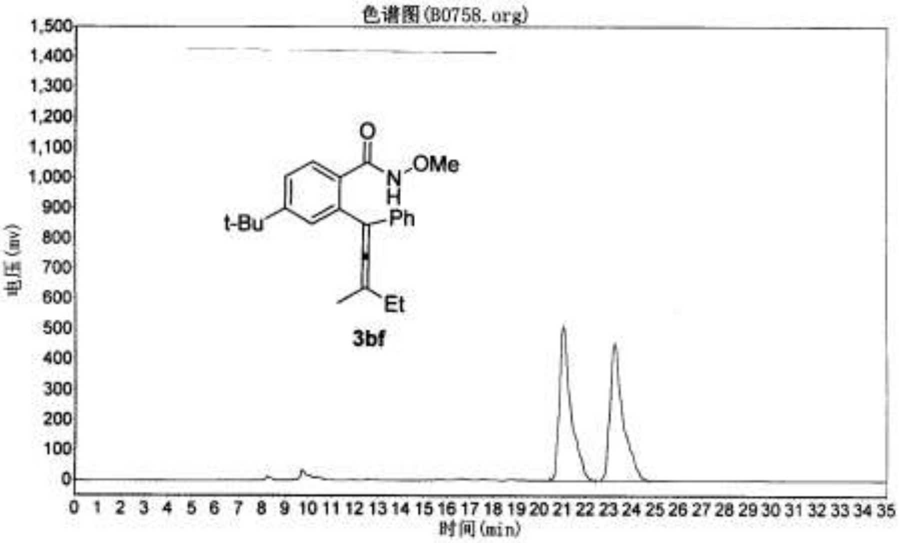

分析结果表

峰号	峰名	保留时间	峰高	峰面积	含量
1		21.045	503280.500	17802136.000	50.1567
2		23.253	445773.938	17690890.000	49.8433
总计			949054.438	35493026.000	100.0000

2015-02-04 浙江大学智能信息研究所

Supplementary Figure 73. HPLC spectrum for 3bf.

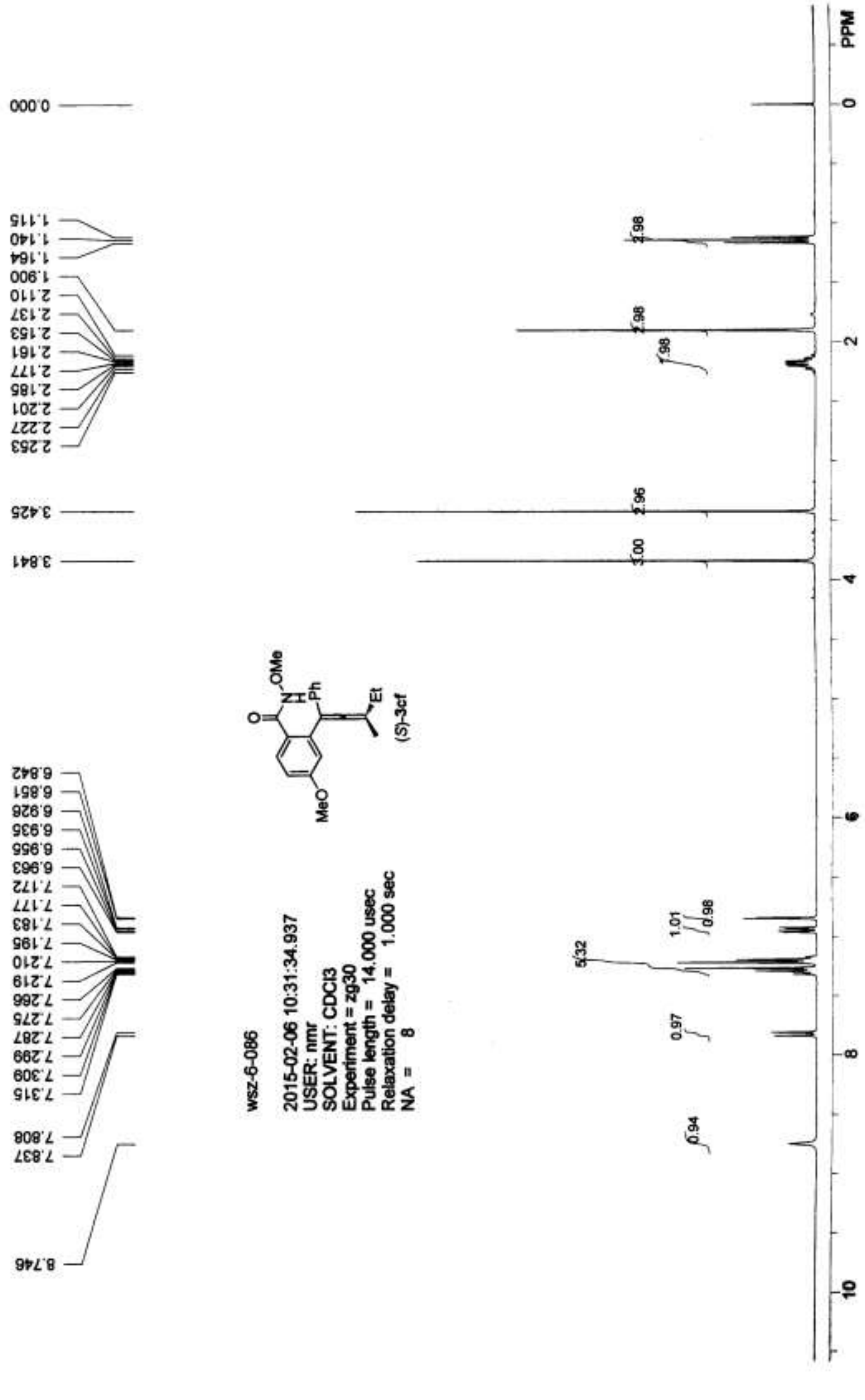

Supplementary Figure 74. **^{1}H NMR (300 MHz, $CDCl_3$) spectrum for *S*-3cf.**

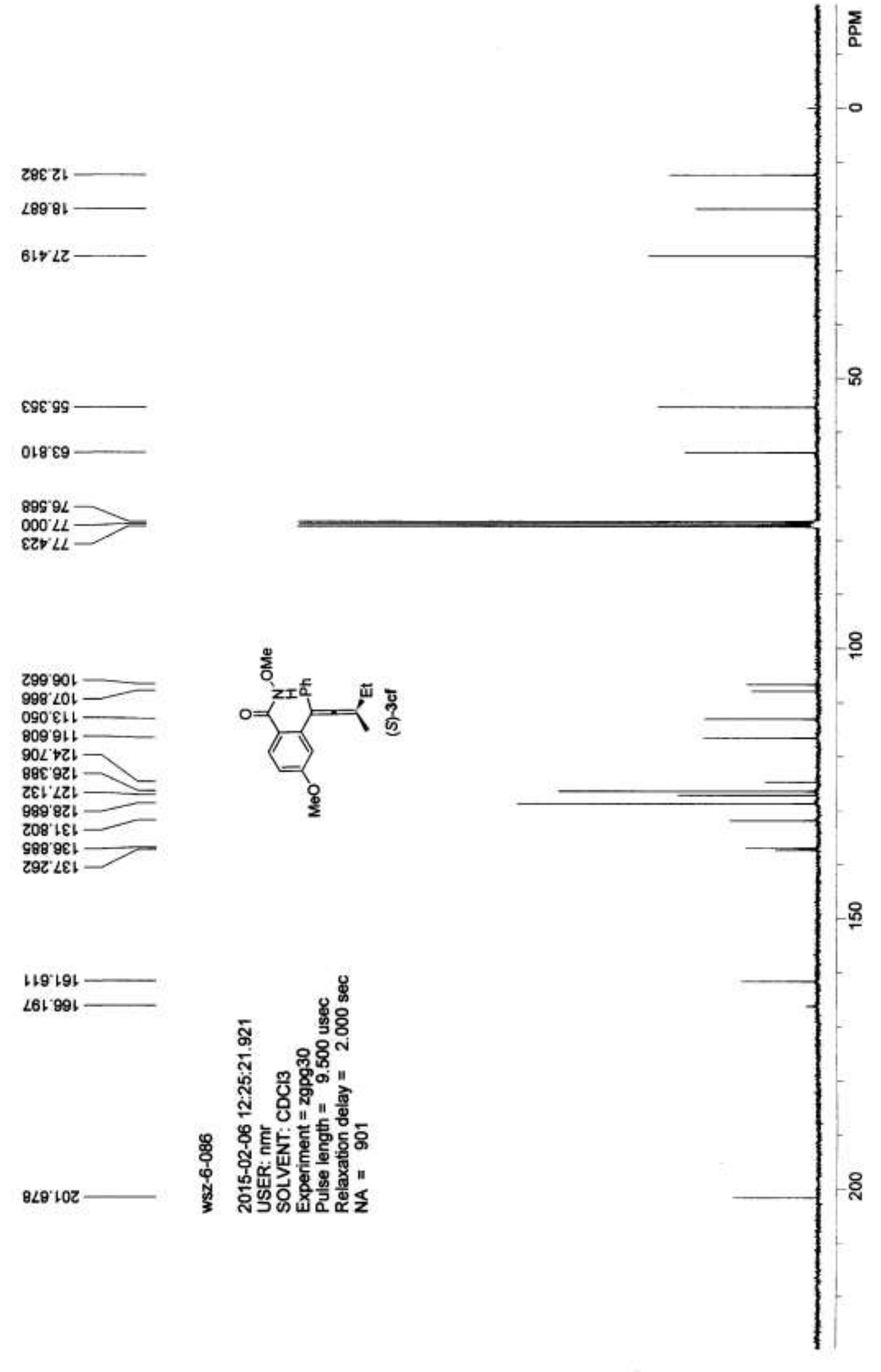

Supplementary Figure 75. ^{13}**C NMR (75 MHz, CDCl$_3$) spectrum for *S*-3cf.**

N2000 数据工作站

wsz-6-86

实验单位：zju
实验时间：2015/2/4, 2:05:44
谱图文件:D:\浙大智达\N2000\样品\B0745.org

实验者：wsz
报告时间：2015/2/4, 2:59:34
积分方法:面积归一法

实验内容简介：
AD-H, n-hexane/i-PrOH = 10/1, 207 nm, 1.0 ml/min

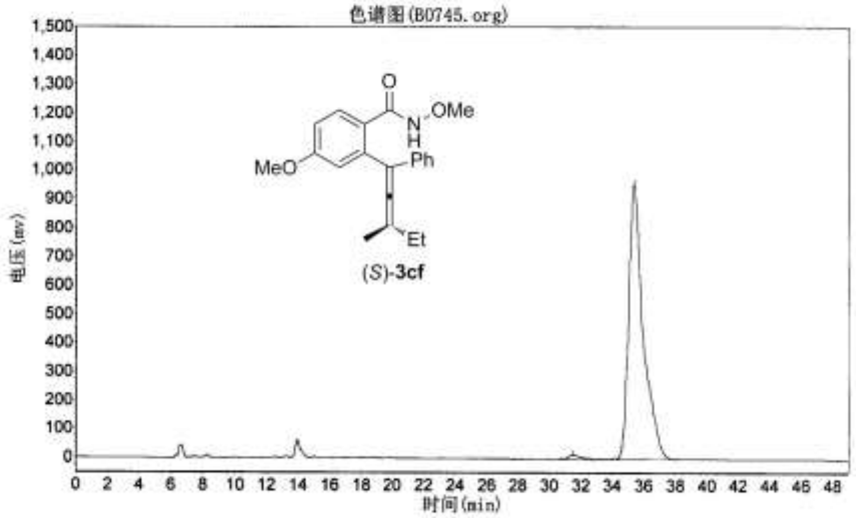

分析结果表

峰号	峰名	保留时间	峰高	峰面积	含量
1		31.517	13009.511	649407.000	1.0540
2		35.360	954973.000	60962120.000	98.9460
总计			967982.511	61611527.000	100.0000

2015/2/4 浙江大学智能信息研究所

Supplementary Figure 76. HPLC spectrum for *S*-3cf.

N2000 数据工作站

wsz-6-83

实验单位：zju
实验时间：2015/2/3, 20:06:50
谱图文件:D:\浙大智达\N2000\样品\B0737.org

实验者：wsz
报告时间：2015/2/3, 20:54:22
积分方法:面积归一法

实验内容简介：
AD-H, n-hexane/i-PrOH = 10/1, 207 nm, 1.0 ml/min

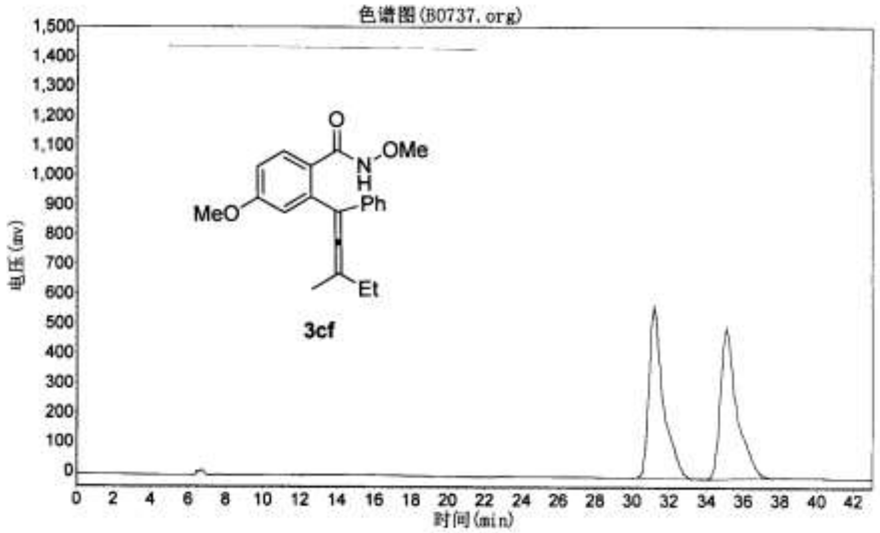

分析结果表

峰号	峰名	保留时间	峰高	峰面积	含量
1		31.185	571564.375	31181156.000	49.8129
2		35.090	503542.625	31415440.000	50.1871
总计			1075107.000	62596596.000	100.0000

2015/2/3 浙江大学智能信息研究所

Supplementary Figure 77. HPLC spectrum for 3cf.

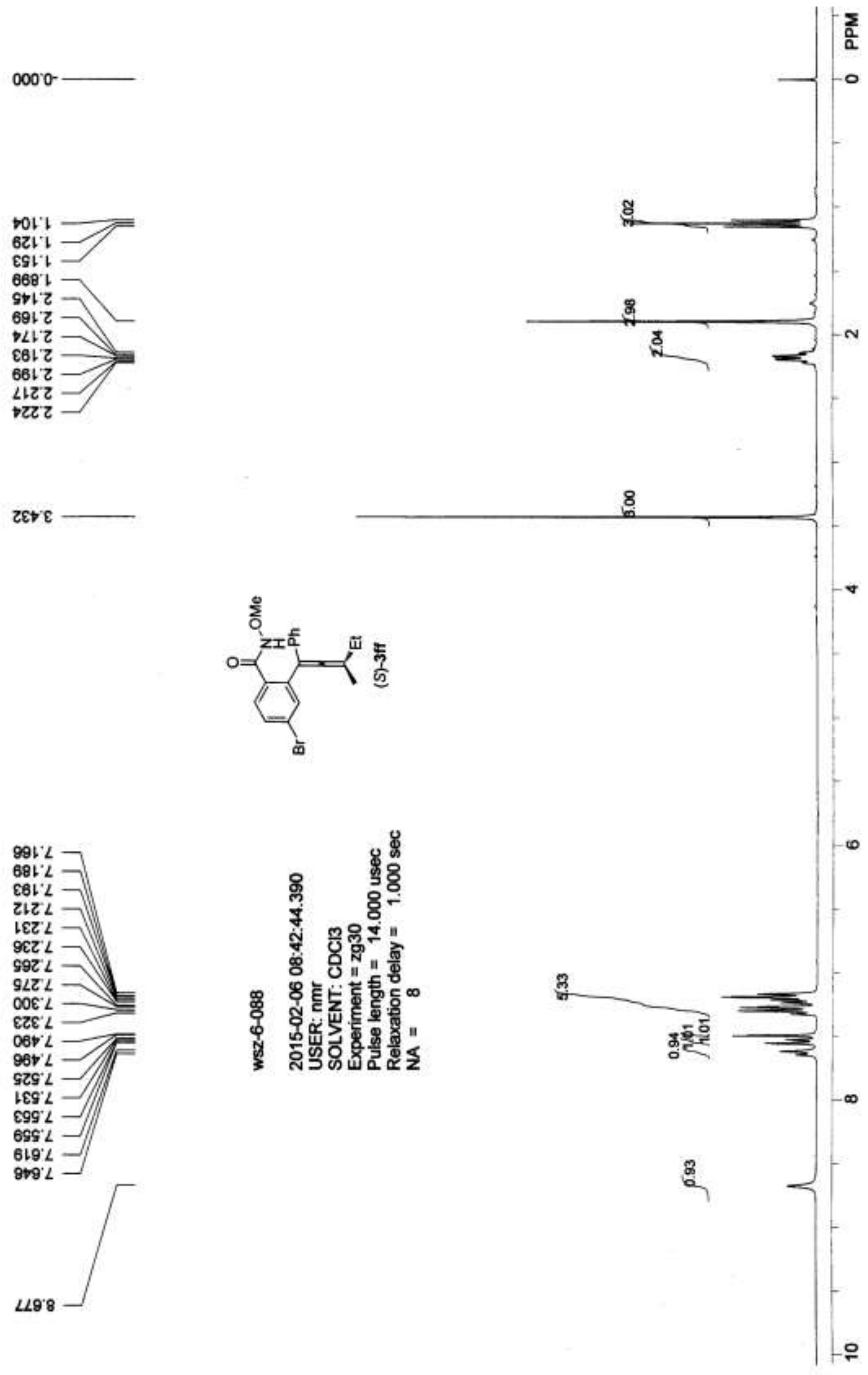

Supplementary Figure 78. ^{1}**H NMR (300 MHz,** $CDCl_3$**) spectrum for** ***S*****-3ff.**

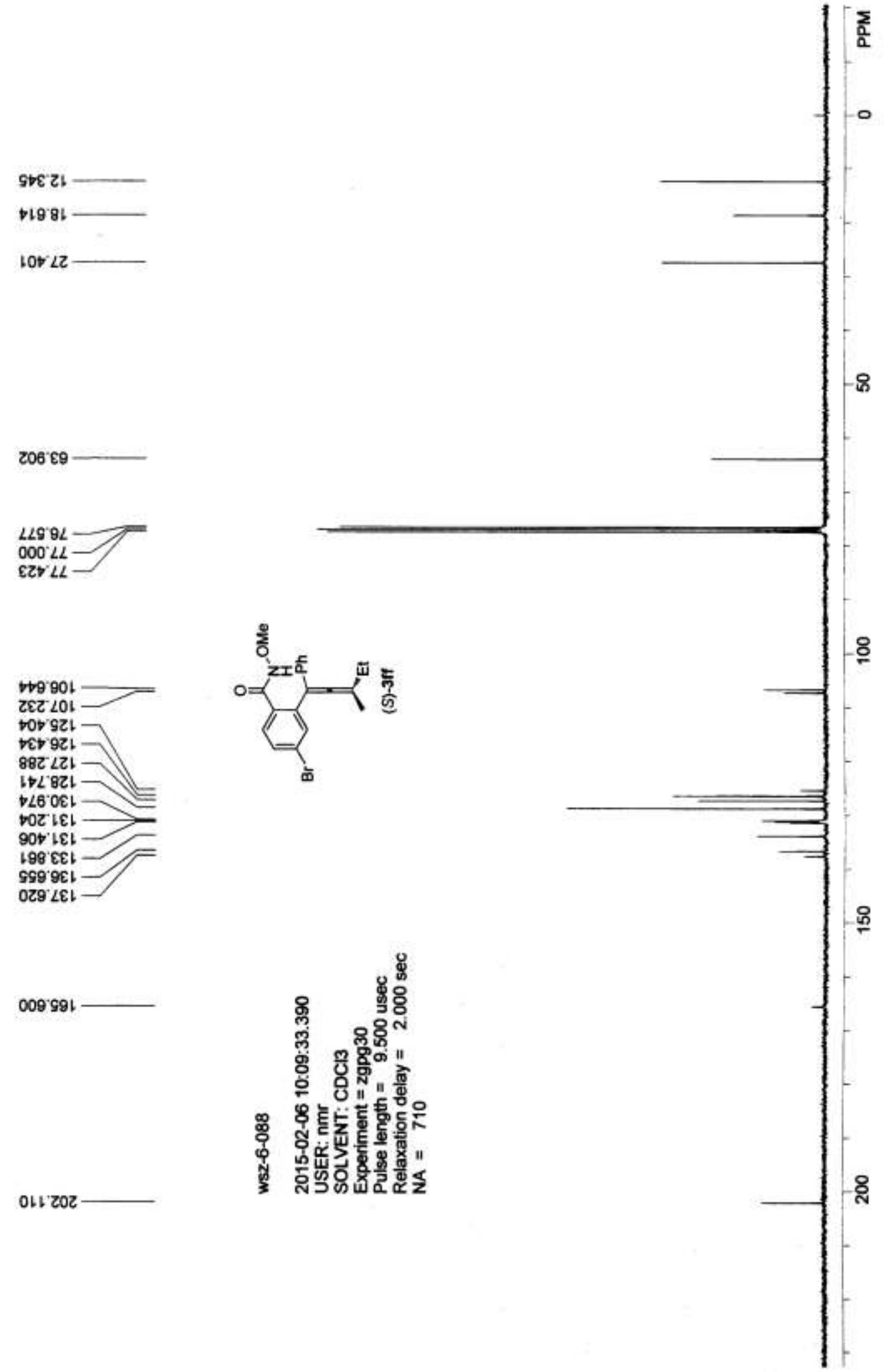

Supplementary Figure 79. ^{13}C NMR (75 MHz, $CDCl_3$) spectrum for *S*-3ff.

wsz-6-88

实验单位：zju
实验时间：2015-02-05, 18:10:31
谱图文件:D:\浙大智达\N2000\样品\B0778.org

实验者：wsz
报告时间：2015-02-05, 19:08:14
积分方法:面积归一法

实验内容简介：
AD-H, n-hexane/i-PrOH = 10/1, 207 nm, 0.7 ml/min

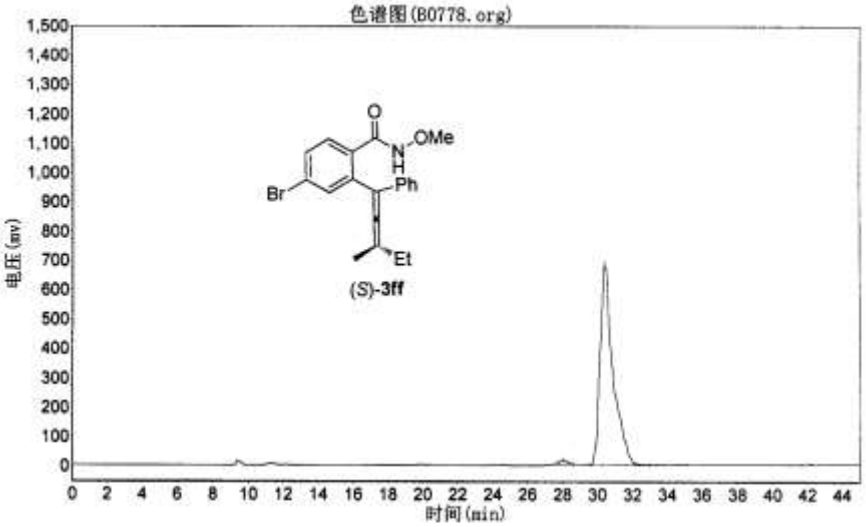

分析结果表

峰号	峰名	保留时间	峰高	峰面积	含量
1		28.020	13423.392	440054.281	1.2453
2		30.390	676632.688	34896604.000	98.7547
总计			690056.079	35336658.281	100.0000

2015-02-05 浙江大学智能信息研究所

Supplementary Figure 80. HPLC spectrum for *S*-3ff.

wsz-6-85

实验单位：zju
实验时间：2015-02-05, 12:29:24
谱图文件:D:\浙大智达\N2000\样品\B0771.org

实验者：wsz
报告时间：2015-02-05, 13:37:45
积分方法:面积归一法

实验内容简介：
AD-H, n-hexane/i-PrOH = 10/1, 207 nm, 0.7 ml/min

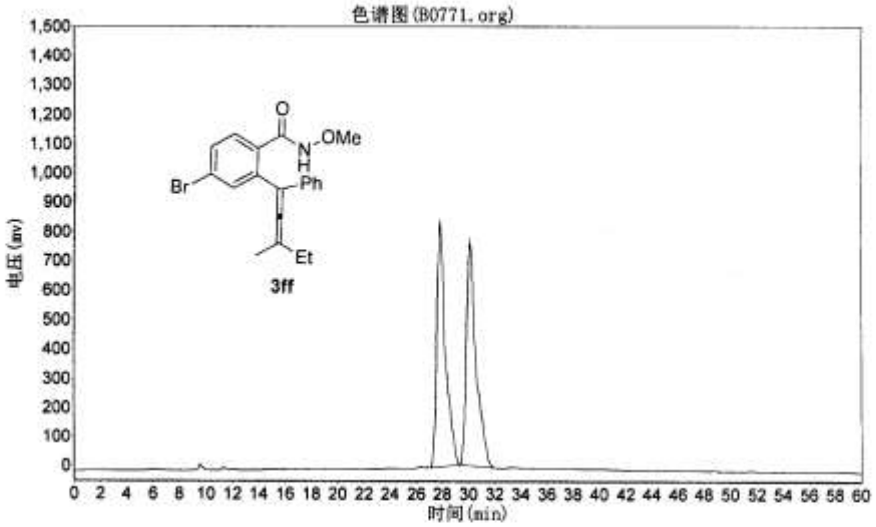

分析结果表

峰号	峰名	保留时间	峰高	峰面积	含量
1		27.818	832422.375	38595008.000	49.7093
2		30.090	761037.500	39046380.000	50.2907
总计			1593459.875	77641388.000	100.0000

Supplementary Figure 81. HPLC spectrum for 3ff.

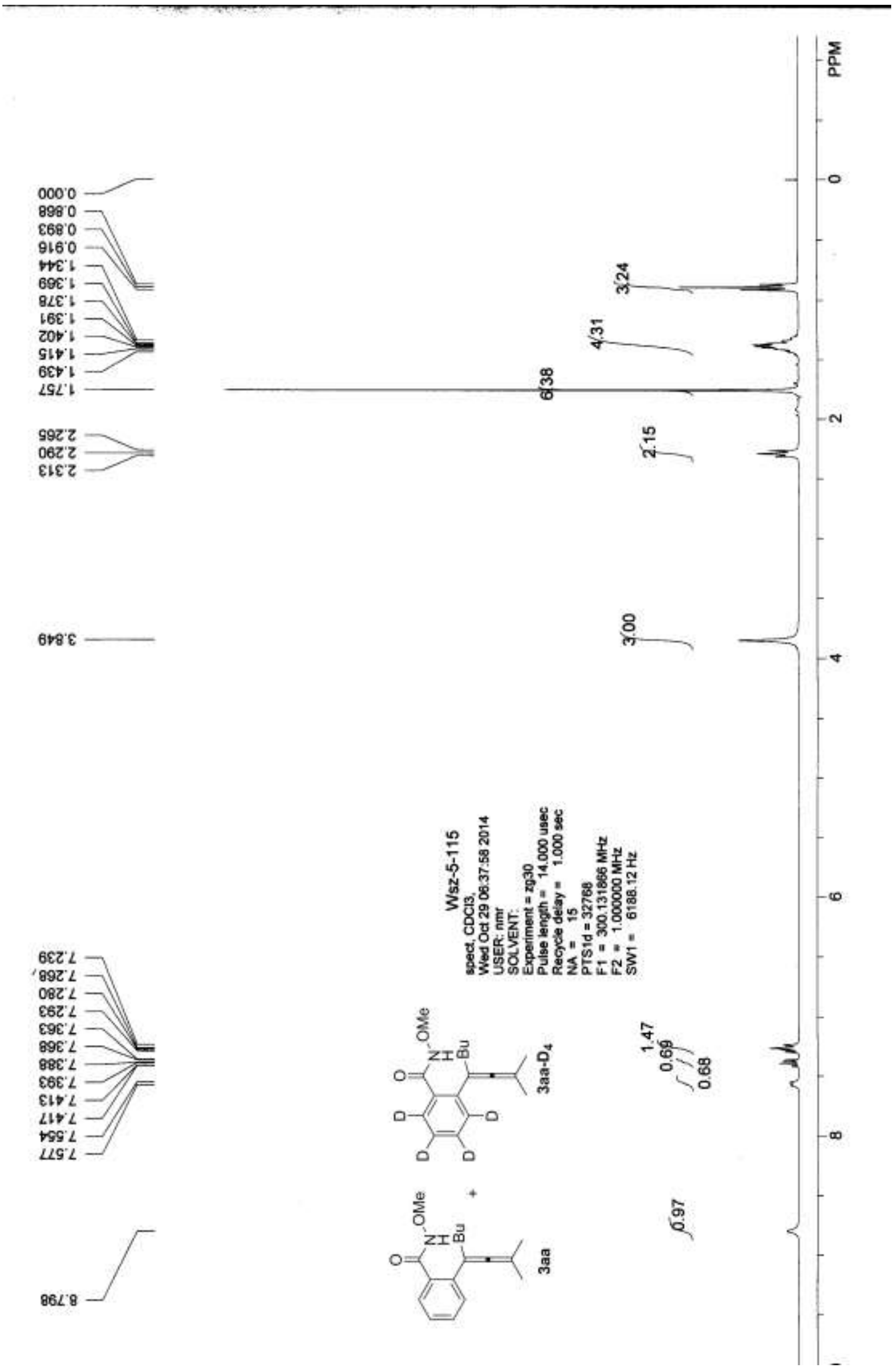

Supplementary Figure 82. ^{1}H NMR (300 MHz, $CDCl_3$) spectrum for KIE.

Supplementary Table 1. Optimization of reaction conditions: the Rh-catalyzed allenylation of 1a with 2a[a].

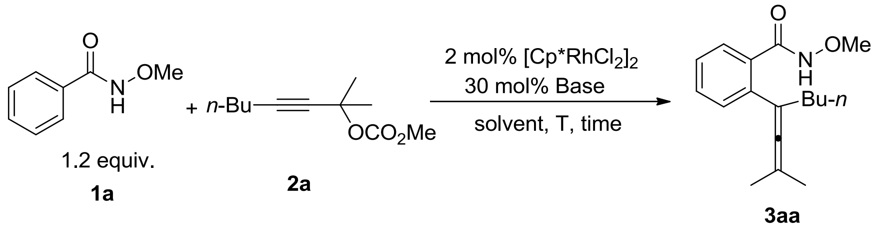

Entry	Base	Solvent	T (°C)	Time (h)	NMR yield of **3aa** (%)[b]	Recovery of **2a** (%)
1[c]	NaOAc	MeOH/H_2O[d]	rt	14	76	0
2[c]	NaOAc	MeOH/H_2O[d]	0	72	70	16
3[c]	NaOAc	MeOH/H_2O[d]	0	23	86 (80)	0
4[c]	NaOAc	MeOH/H_2O[d]	-20	72	40	44
5	NaOAc	MeOH	0	23	68	18
6	NaOAc	DCE	0	23	57	29
7	NaOAc	THF	0	21	-	80
8	NaOAc	toluene	0	21	54	33
9	NaOAc	MeCN	0	21	-	84
10	KOAc	MeOH/H_2O[d]	0	25	78	8
11	CsOAc	MeOH/H_2O[d]	0	25	74	0
12	Na_2CO_3	MeOH/H_2O[d]	0	25	-	75
13	K_2CO_3	MeOH/H_2O[d]	0	25	-	65
14[e]	NaOAc	MeOH/H_2O[d]	0	16	-	87
15	-	MeOH/H_2O[d]	0	21	-	89

[a] The reaction was conducted with **1a** (0.24 mmol), **2a** (0.2 mmol), [Cp*$RhCl_2$]$_2$ (0.004 mmol), base (0.06 mmol), solvent (1.2 mL) and monitored by TLC. [b] Determined by ^{1}H NMR of the crude product using CH_2Br_2 as internal standard. Isolated yield is in the parenthesis. [c] 1.0 equiv. **1a** was used. [d] The ratio of MeOH/H_2O was 20/1. [e] The reaction was conducted in the absence of the Rh^{III} catalyst.

Supplementary methods

Synthesis of chiral starting materials

1. Preparation of (*S*)-methyl (3-methyl-1-phenylpent-1-yn-3-yl) carbonate *S*-**2f**. (wsz-6-63)

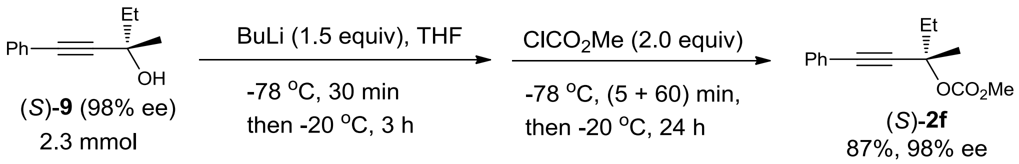

To a solution of (*S*)-**9**[1] (98% ee, 0.4012 g, 2.3mmol) in THF (15 mL) was added dropwise *n*-BuLi (2.5 M in hexane, 1.4 mL, 3.50 mmol) at -78 °C in 5 minutes. After being stirred for 30 min at -78 °C, the reaction mixture was warmed up to -20 °C and stirred for 3 h. Then methyl chloroformate (0.36 mL, d = 1.22 g/mL, 0.44 g, 4.6 mmol) was added dropwise at -78 °C in 5 minutes. After being stirred for 1 h at -78 °C, the reaction mixture was warmed up to -20 °C and stirred for 24 h as monitored by TLC (eluent: petroleum ether/ethyl acetate = 20/1). Then the reaction was quenched with saturated aqueous NH_4Cl (15 mL) at -20 °C and warmed up to room temperature. The resulted solution was extracted with ether (30 mL × 3). The combined organic layer was washed with brine and dried over anhydrous Na_2SO_4. After filtration, evaporation of the solvent and chromatography on silica gel (eluent: petroleum

ether/ethyl acetate (100/1 to 60/1)) afforded (*S*)-**2f** (0.4711 g, 87%) as an oil: 98% ee (HPLC conditions: Chiralcel OD-H column, hexane/*i*-PrOH = 200/1, 0.8 mL/min, λ = 207 nm, t_R(major) = 17.3 min, t_R(minor) = 21.0 min); $[\alpha]_D^{20}$ = -39.7 (*c* = 1.005, $CHCl_3$); ^{1}H NMR (300 MHz, $CDCl_3$) δ 7.48-7.40 (m, 2 H, ArH), 7.35-7.25 (m, 3 H, ArH), 3.77 (s, 3 H, Me), 2.17-1.86 (m, 2 H, CH_2), 1.78 (s, 3 H, Me), 1.11 (t, *J* = 7.4 Hz, 3 H, Me); ^{13}C NMR (75 MHz, $CDCl_3$) δ 153.5, 131.8, 128.4, 128.1, 122.4, 88.4, 85.5, 78.4, 54.2, 34.5, 25.8, 8.6; IR (neat, cm^{-1}) 3081, 3057, 2980, 2979, 2956, 2938, 2882, 2849, 2221, 1754, 1599, 1490, 1441, 1373, 1312, 1267, 1191, 1150, 1126, 1098, 1028; MS (EI): *m/z* (%) 232 (M^+, 10.16), 156 (100); HRMS calcd. for $C_{14}H_{16}O_3$ (M^+): 232.1099; Found: 232.1097.

Rh^{III}-Catalyzed Synthesis of Tetra-substituted allenes 3

1. Preparation of **3aa**. (Wsz-4-18)

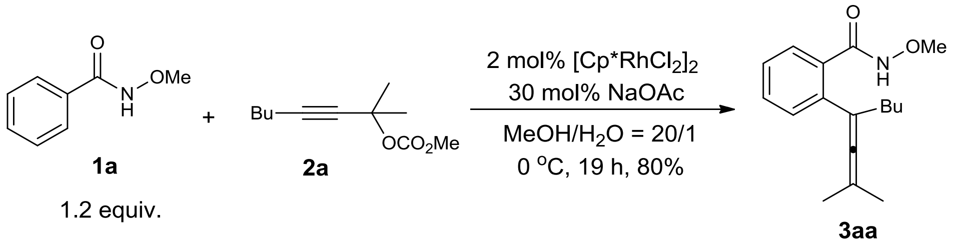

3aa

Typical Procedure: To a dried Schlenk tube equipped with a Teflon-coated magnetic stirring bar were added *N*-pivaloyloxybenzamide **1a** (182.1 mg, 1.2 mmol), $[Cp^*RhCl_2]_2$ (12.6 mg, 0.02 mmol), NaOAc (24.3 mg, 0.3 mmol), methyl (2-methyloct-3-yn-2-yl) carbonate **2a** (198.5 mg, 1 mmol), MeOH (6 mL), and H_2O (0.3 mL) sequentially at rt. After being stirred for 19 h at 0 °C, the reaction was complete as monitored by TLC. Filtration through a short column of silica gel (eluent: ethyl acetate 20 mL × 3) and evaporation afforded the crude product, which was purified by flash column chromatography on silica gel

(eluent: petroleum/ethyl acetate/dichloromethane = 10/1/0.1/ to 5/1/0.5) to afford **3aa** (218.6 mg, 80%): solid; m.p. 66.9-68.1 ^oC (hexane/ethyl acetate); ^1H NMR (300 MHz, $CDCl_3$) δ 8.74 (brs, 1 H, NH), 7.58 (d, J = 7.2 Hz, 1 H, Ar-H), 7.39 (td, J_1 = 7.5 Hz, J_2 = 1.3 Hz, 1 H, Ar-H), 7.32-7.21 (m, 2 H, Ar-H), 3.86 (s, 3 H, OCH_3), 2.29 (t, J = 7.1 Hz, 2 H, CH_2), 1.76 (s, 6 H, 2 × CH_3), 1.49-1.27 (m, 4 H, 2 × CH_2), 0.89 (t, J = 6.9 Hz, 3 H, CH_3); ^{13}C NMR (75 MHz, $CDCl_3$) δ 201.3, 167.9, 138.3, 131.5, 130.5, 129.1, 126.7, 102.9, 97.6, 64.3, 33.6, 30.0, 22.1, 20.3, 13.9; IR ν (neat, cm^{-1}) 3188, 2956, 2931, 2875, 2859, 1953, 1659, 1590, 1495, 1465, 1440, 1299, 1156, 1035; MS (EI, 70 eV) m/z (%) 273 (M^+, 4.18), 242 (100); Anal. Calcd for $C_{17}H_{23}NO_2$: C 74.69, H 8.48, N 5.12. Found: C 74.89, H 8.62, N 4.89.

The following compounds were prepared according to the Typical Procedure.

Preparation of **3aa** in 7 mmol scale. (Wsz-4-128)

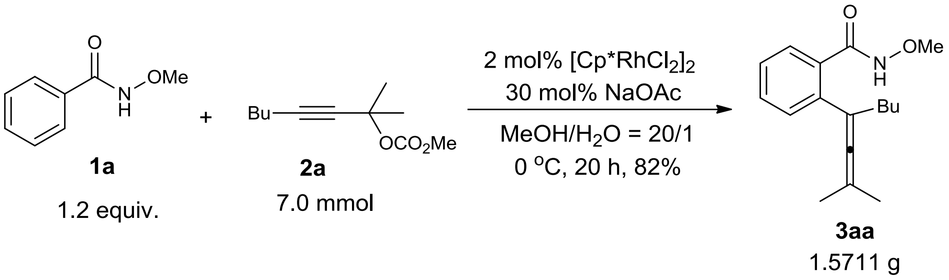

The reaction of **1a** (1269.0 mg, 8.4 mmol), $[Cp^*RhCl_2]_2$ (86.7 mg, 0.14 mmol), NaOAc (173.1 mg, 2.1 mmol), **2a** (1387.8 mg, 7 mmol), MeOH (42 mL), and H_2O (2.1 mL) at 0 ^oC afforded **3aa** (1571.1 mg, 82%) (eluent: dichloromethane/ethyl acetate = 10/1): ^1H NMR (300 MHz, $CDCl_3$) δ 8.88 (s, 1 H, NH), 7.54 (d, J = 7.5 Hz, 1 H, Ar-H), 7.38 (td, J_1 = 7.5 Hz, J_2 = 1.2 Hz, 1 H, Ar-H), 7.30-7.20 (m, 2 H, Ar-H), 3.83 (s, 3 H, OCH_3), 2.29 (t, J = 7.1 Hz, 2 H, CH_2), 1.75 (s, 6 H, CH_3 × 2), 1.49-1.27 (m, 4 H, CH_2 × 2), 0.89 (t, J = 6.9 Hz, 3 H, CH_3).

2. Preparation of **3ba**. (Wsz-4-125)

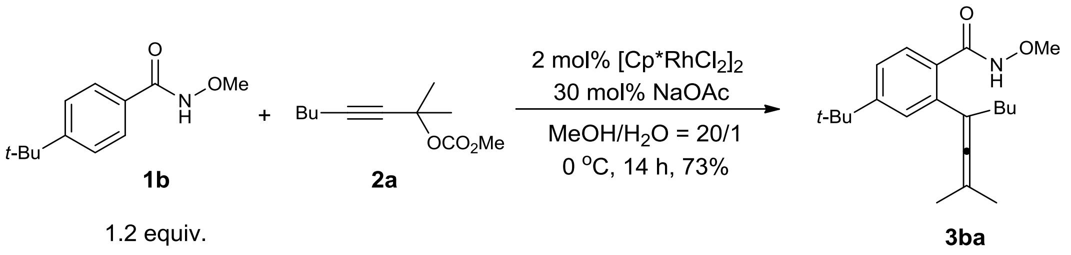

The reaction of **1b** (249.3 mg, 1.2 mmol), $[Cp^*RhCl_2]_2$ (12.6 mg, 0.02 mmol), NaOAc (24.1 mg, 0.3 mmol), **2a** (199.5 mg, 1 mmol), MeOH (6 mL), and H_2O (0.3 mL) at 0 ^oC afforded **3ba** (243.0 mg, 73%) (eluent: petroleum/ethyl acetate/dichloromethane = 10/1/0.1/ to 5/1/0.1): oil; ^1H NMR (300 MHz, $CDCl_3$) δ 8.82 (brs, 1 H, NH), 7.54 (d, J = 8.1 Hz, 1 H, Ar-H), 7.30 (dd, J_1 = 8.1 Hz, J_2 = 1.8 Hz, 1 H, Ar-H), 7.23 (d, J = 1.8 Hz, 1 H, Ar-H), 3.84 (s, 3 H, OCH_3), 2.29 (t, J = 7.2 Hz, 2 H, CH_2), 1.76 (s, 6 H, 2 × CH_3), 1.51-1.33 (m, 4 H, 2 × CH_2), 1.31 (s, 9 H, 3 × CH_3), 0.90 (t, J = 7.1 Hz, 3 H, CH_3); ^{13}C NMR (75 MHz, $CDCl_3$) δ 201.1, 167.9, 154.0, 138.0, 129.0, 128.6, 126.1, 124.0, 103.6, 97.4, 64.3, 34.7, 33.8, 31.0, 30.0, 22.2, 20.4, 13.9; IR ν (neat, cm^{-1}) 3192, 2960, 2932, 2870, 1962, 1659, 1601, 1464, 1375, 1362, 1301, 1261, 1103, 1038; MS (EI, 70 eV) m/z (%) 330 (M^++1, 12.91), 329 (M^+, 55.81), 70 (100); HRMS Calcd for $C_{21}H_{31}NO_2$ (M^+): 329.2355. Found: 329.2354.

3. Preparation of **3ca**. (Wsz-4-111)

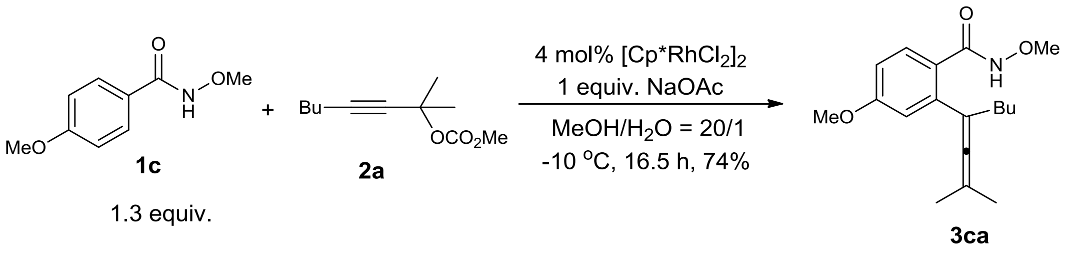

The reaction of **1c** (236.5 mg, 1.3 mmol), $[Cp^*RhCl_2]_2$ (24.5 mg, 0.04 mmol), NaOAc (82.5 mg, 1 mmol), **2a** (197.9 mg, 1 mmol), MeOH (6 mL), and H_2O (0.3 mL) at -10 ^oC

afforded **3ca** (223.9 mg, 74%) (eluent: petroleum/ethyl acetate/dichloromethane = 5/1/0.5): oil; ^{1}H NMR (300 MHz, $CDCl_3$) δ 8.83 (brs, 1 H, NH), 7.61 (d, J = 8.7 Hz, 1 H, Ar-H), 6.79 (dd, J_1 = 8.6 Hz, J_2 = 2.6 Hz, 1 H, Ar-H), 6.72 (d, J = 2.7 Hz, 1 H, Ar-H), 3.83 (s, 3 H, OCH_3), 3.81 (s, 3 H, OCH_3), 2.25 (t, J = 7.2 Hz, 2 H, CH_2), 1.75 (s, 6 H, 2 × CH_3), 1.46-1.26 (m, 4 H, 2 × CH_2), 0.87 (t, J = 7.1 Hz, 3 H, CH_3); ^{13}C NMR (75 MHz, $CDCl_3$) δ 200.9, 167.6, 161.2, 140.2, 131.3, 123.7, 115.1, 111.8, 103.4, 97.7, 64.4, 55.3, 33.8, 30.1, 22.2, 20.4, 13.9; IR ν (neat, cm^{-1}) 3195, 2956, 2932, 2872, 2855, 1953, 1658, 1601, 1567, 1465, 1312, 1280, 1242, 1198, 1173, 1105, 1030; MS (EI, 70 eV) m/z (%) 304 (M^++1, 6.99), 303 (M^+, 34.50), 218 (100); HRMS Calcd for $C_{18}H_{25}NO_3$ (M^+): 303.1834. Found: 303.1838.

4. Preparation of **3da**. (Wsz-4-69)

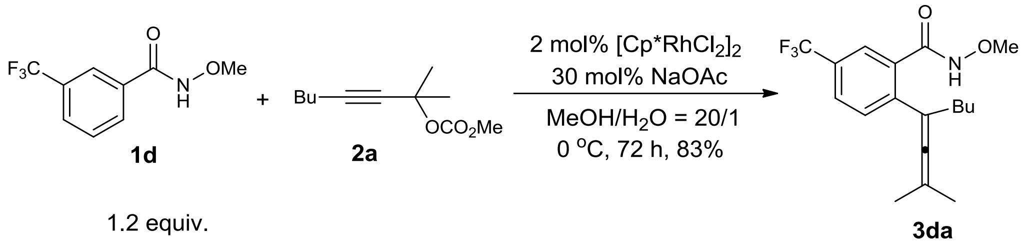

The reaction of **1d** (262.8 mg, 1.2 mmol), $[Cp^*RhCl_2]_2$ (12.6 mg, 0.02 mmol), NaOAc (24.3 mg, 0.3 mmol), **2a** (198.8 mg, 1 mmol), MeOH (6 mL), and H_2O (0.3 mL) at 0 °C afforded **3da** (284.4 mg, 83%) (eluent: dichloromethane/ethyl acetate = 60/1 to 20/1): solid; m.p. 88.2-89.3 °C (hexane/ethyl acetate); ^{1}H NMR (300 MHz, $CDCl_3$) δ 8.78 (brs, 1 H, NH), 7.79 (s, 1 H, Ar-H), 7.63 (d, J = 8.4 Hz, 1 H, Ar-H), 7.39 (d, J = 7.8 Hz, 1 H, Ar-H), 3.87 (s, 3 H, OCH_3), 2.31 (t, J = 6.8 Hz, 2 H, CH_2), 1.76 (s, 6 H, 2 × CH_3), 1.50-1.28 (m, 4 H, 2 × CH_2), 0.90 (t, J = 6.9 Hz, 3 H, CH_3); ^{13}C NMR (75 MHz, $CDCl_3$) δ 202.0, 166.7, 142.16 (d, J = 1.4 Hz), 132.3, 129.4, 128.9 (q, J = 32.1 Hz), 127.1, 126.2, 123.6 (q, J = 270.6 Hz), 101.9, 98.9,

64.5, 33.1, 30.0, 22.2, 20.2, 13.9; ^{19}F NMR (282 MHz, $CDCl_3$) δ -63.1; IR ν (neat, cm^{-1}) 3195, 2960, 2935, 2873, 1953, 1660, 1615, 1510, 1465, 1442, 1381, 1362, 1336, 1300, 1271, 1174, 1157, 1129, 1081, 1045; MS (EI, 70 eV) *m/z* (%) 342 (M^++1, 2.44), 341 (M^+, 4.73), 43 (100); Anal. Calcd for $C_{18}H_{22}F_3NO_2$: C 63.33, H 6.50, N 4.10. Found: C 63.11, H 6.45, N 3.86.

5. Preparation of **3ea**. (Wsz-4-117)

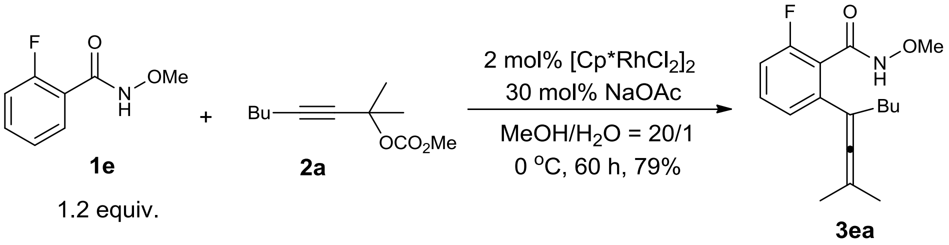

The reaction of **1e** (203.2 mg, 1.2 mmol), $[Cp^*RhCl_2]_2$ (12.5 mg, 0.02 mmol), NaOAc (24.1 mg, 0.3 mmol), **2a** (197.5 mg, 1 mmol), MeOH (6 mL), and H_2O (0.3 mL) at 0 ^oC afforded **3ea** (228.8 mg, 79%) (eluent: hexane/ethyl acetate/dichloromethane = 10/1/0.5): solid; m.p. 99.9-100.8 ^oC (hexane/ethyl acetate); ^{1}H NMR (300 MHz, $CDCl_3$) δ 8.42 (s, 1 H, NH), 7.36-7.25 (m, 1 H, Ar-H), 7.08 (d, J = 7.8 Hz, 1 H, Ar-H), 6.94 (t, J = 8.6 Hz, 1 H, Ar-H), 3.86 (s, 3 H, CH_3), 2.31 (t, J = 6.6 Hz, 2 H, CH_2), 1.74 (s, 6 H, CH_3 × 2), 1.48-1.25 (m, 4 H, CH_2 × 2), 0.90 (t, J = 6.9 Hz, 3 H, CH_3); ^{13}C NMR (75 MHz, $CDCl_3$) δ 201.8, 163.0, 160.0 (d, J = 260.0 Hz), 140.8, 131.0 (d, J = 7.6 Hz), 123.7, 120.7 (d, J = 17.9 Hz), 113.5 (d, J = 22.1 Hz), 100.9 (d, J = 2.1 Hz), 98.6, 64.4, 32.8, 30.0, 22.2, 20.2, 13.9; ^{19}F NMR(282 MHz, $CDCl_3$) δ -116.1 (s, 1 F); IR ν (neat, cm^{-1}) 3183, 2953, 2933, 2872, 2855, 1962, 1666, 1608, 1569, 1495, 1446, 1361, 1297, 1258, 1234, 1148, 1037; MS (EI, 70 eV) *m/z* (%) 291 (M^+, 13.56), 218 (100); Anal. Calcd for $C_{17}H_{22}FNO_2$: C 70.08, H 7.61, N 4.81. Found: C 70.01, H 7.31, N 4.73.

6. Preparation of **3ac**. (Wsz-4-110)

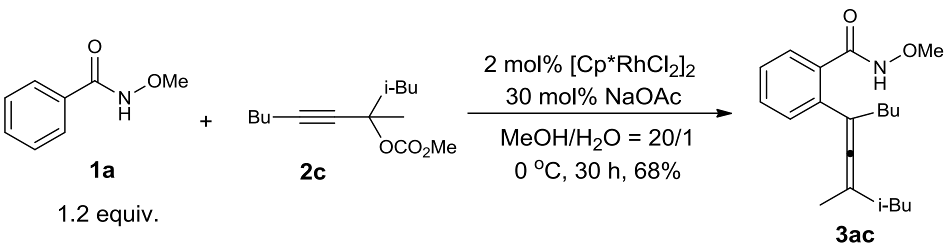

The reaction of **1a** (182.0 mg, 1.2 mmol), $[Cp^*RhCl_2]_2$ (12.5 mg, 0.02 mmol), NaOAc (24.9 mg, 0.3 mmol), **2c** (240.5 mg, 1 mmol), MeOH (6 mL), and H_2O (0.3 mL) at 0 ^{o}C afforded **3ac** (215.3 mg, 68%) (eluent: hexane/ethyl acetate/dichloromethane = 10/1/0.5): oil; ^{1}H NMR (300 MHz, $CDCl_3$) δ 8.84 (s, 1 H, NH), 7.62 (d, J = 7.5 Hz, 1 H, Ar-H), 7.38 (td, J_1 = 7.5 Hz, J_2 = 1.5 Hz, 1 H, Ar-H), 7.32-7.20 (m, 2 H, Ar-H), 3.85 (s, 3 H, CH_3), 2.40-2.19 (m, 2 H, CH_2), 1.91 (d, J = 7.2 Hz, 2 H, CH_2), 1.83-1.65 (m, 4 H, CH and CH_3), 1.49-1.24 (m, 4 H, CH_2 × 2), 0.94-0.80 (m, 9 H, CH_3 × 3); ^{13}C NMR (75 MHz, $CDCl_3$) δ 201.5, 167.8, 138.4, 131.3, 130.7, 129.5, 129.2, 126.9, 103.9, 100.5, 64.3, 43.9, 34.0, 30.3, 26.4, 22.6, 22.5, 22.2, 18.8, 13.8; IR ν (neat, cm^{-1}) 3196, 3062, 2955, 2926, 2869, 1953, 1660, 1595, 1464, 1439, 1381, 1366, 1300, 1159, 1035; MS (EI, 70 eV) m/z (%) 315 (M^+, 5.68), 284 (100); HRMS Calcd for $C_{20}H_{29}NO_2$ (M^+): 315.2198. Found: 315.2195.

7. Preparation of **3ad**. (Wsz-5-44)

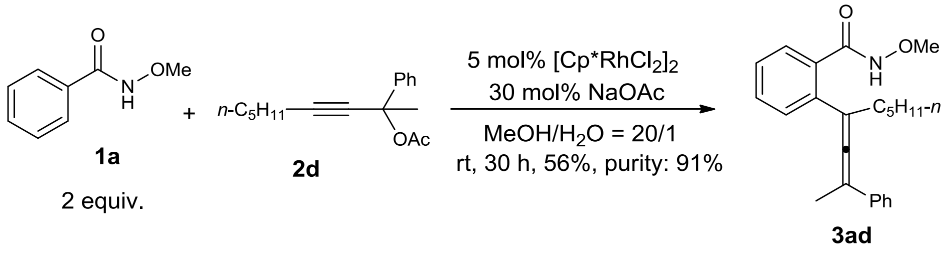

The reaction of **1a** (302.5 mg, 2 mmol), $[Cp^*RhCl_2]_2$ (30.2 mg, 0.05 mmol), NaOAc

(24.3 mg, 0.3 mmol), **2d** (258.1 mg, 1 mmol), MeOH (6 mL), and H_2O (0.3 mL) at rt afforded **3ad** (215.7 mg, 56%, purity: 91%) (eluent: hexane/ethyl acetate/dichloromethane = 10/1/0.5): oil; ^{1}H NMR (300 MHz, $CDCl_3$) δ 8.73 (s, 1 H, NH), 7.51 (d, J = 6.9 Hz, 1 H, Ar-H), 7.46-7.13 (m, 8 H, Ar-H), 3.83 (s, 3 H, OCH_3), 2.53-2.34 (m, 2 H, CH_2), 2.18 (s, 3 H, CH_3), 1.58-1.43 (m, 2 H, CH_2), 1.41-1.17 (m, 4 H, 2 × CH_2), 0.84 (t, J = 7.1 Hz, 3 H, CH_3); ^{13}C NMR (75 MHz, $CDCl_3$) δ 203.8, 167.6, 137.5, 137.2, 131.6, 130.7, 129.4, 128.9, 128.4, 127.2, 126.8, 125.7, 107.5, 102.7, 64.4, 34.0, 31.4, 27.5, 22.4, 16.8, 14.0; IR ν (neat, cm^{-1}) 3193, 3060, 2956, 2930, 2857, 1941, 1661, 1596, 1493, 1463, 1441, 1370, 1301, 1159, 1028; MS (EI, 70 eV) m/z (%) 350 (M^++1, 3.60), 349 (M^+, 14.07), 316 (100); HRMS Calcd for $C_{23}H_{27}NO_2$ (M^+): 349.2042. Found: 349.2040.

8. Preparation of **3ae**. (Wsz-4-63)

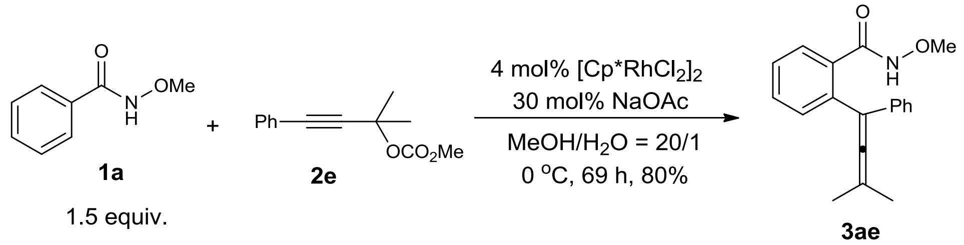

The reaction of **1a** (226.7 mg, 1.5 mmol), $[Cp^*RhCl_2]_2$ (24.5 mg, 0.04 mmol), NaOAc (25.1 mg, 0.3 mmol), **2e** (219.0 mg, 1 mmol), MeOH (6 mL), and H_2O (0.3 mL) at 0 °C afforded **3ae** (235.2 mg, 80%) (eluent: petroleum/ethyl acetate/dichloromethane = 15/1/0.1 to 8/1/0.1): solid; m.p. 126.8-127.7 °C (hexane/ethyl acetate); ^{1}H NMR (300 MHz, $CDCl_3$) δ 8.58 (brs, 1 H, NH), 7.74 (d, J = 7.5 Hz, 1 H, Ar-H), 7.53-7.37 (m, 2 H, Ar-H), 7.36-7.25 (m, 3 H, Ar-H), 7.24-7.15 (m, 3 H, Ar-H), 3.49 (s, 3 H, OCH_3), 1.88 (s, 6 H, 2 × CH_3); ^{13}C NMR (75 MHz, $CDCl_3$) δ 202.8, 166.8, 137.3, 135.6, 132.7, 131.1, 131.0, 129.5, 128.6, 127.8,

127.0, 126.8, 105.4, 100.0, 64.0, 20.2; IR ν (neat, cm^{-1}) 3203, 3057, 2977, 2935, 2902, 2858, 2810, 1953, 1661, 1595, 1491, 1440, 1376, 1361, 1299, 1182, 1153, 1032, 1017; MS (EI, 70 eV) *m*/*z* (%) 294 (M^++1, 3.56), 293 (M^+, 4.34), 262 (100); Anal. Calcd for $C_{19}H_{19}NO_2$: C 77.79, H 6.53, N 4.77. Found: C 77.90, H 6.46, N 4.53.

9. Preparation of **3af**. (Wsz-5-169)

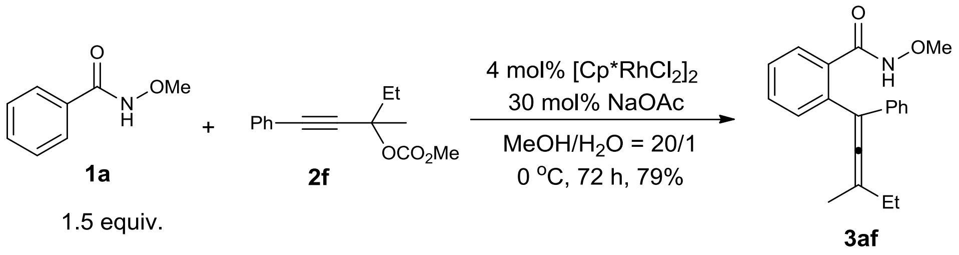

The reaction of **1a** (226.0 mg, 1.5 mmol), $[Cp^*RhCl_2]_2$ (24.6 mg, 0.04 mmol), NaOAc (24.9 mg, 0.3 mmol), **2f** (231.8 mg, 1 mmol), MeOH (6 mL), and H_2O (0.3 mL) at 0 °C afforded **3af** (242.0 mg, 79%) (eluent: petroleum/ethyl acetate/dichloromethane = 10/1/0.2 to 5/1/0.2): solid; m.p. 105.9-107.5 °C (hexane/ethyl acetate); ^1H NMR (300 MHz, $CDCl_3$) δ 8.73 (brs, 1 H, NH), 7.75 (d, J = 7.2 Hz, 1 H, Ar-H), 7.54-7.12 (m, 8 H, Ar-H), 3.44 (s, 3 H, OCH_3), 2.25-2.09 (m, 2 H, CH_2), 1.89 (s, 3 H, CH_3), 1.13 (t, J = 7.4 Hz, 3 H, CH_3); ^{13}C NMR (75 MHz, $CDCl_3$) δ 202.0, 166.5, 137.2, 135.6, 132.5, 131.1, 131.0, 129.5, 128.5, 127.8, 127.0, 126.4, 107.5, 106.3, 63.8, 27.4, 18.6, 12.3; IR ν (neat, cm^{-1}) 3194, 3058, 2966, 2933, 1947, 1663, 1595, 1491, 1456, 1439, 1369, 1302, 1158, 1032; MS (EI, 70 eV) *m*/*z* (%) 307 (M^+, 3.54), 246 (100); Anal. Calcd for $C_{20}H_{21}NO_2$: C 78.15, H 6.89, N 4.56. Found: C 78.10, H 6.60, N 4.49.

10. Preparation of **3ag**. (Wsz-6-34)

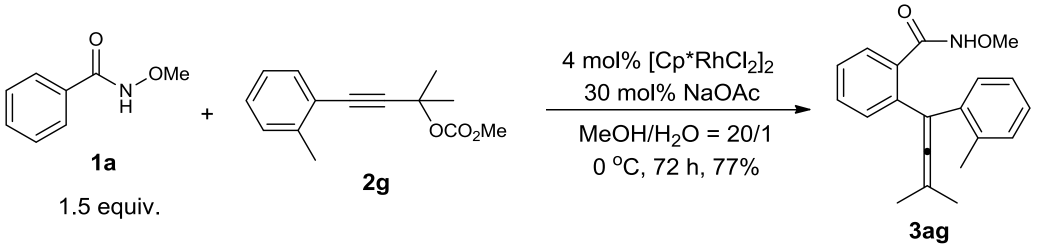

The reaction of **1a** (226.6 mg, 1.5 mmol), $[Cp^*RhCl_2]_2$ (24.8 mg, 0.04 mmol), NaOAc (24.2 mg, 0.3 mmol), **2g** (231.8 mg, 1 mmol), MeOH (6 mL), and H_2O (0.3 mL) at 0 ^oC afforded **3ag** (235.8 mg, 77%) (eluent: petroleum/ethyl acetate/dichloromethane = 10/1/0.1 to 5/1/0.3): oil; ^1H NMR (300 MHz, $CDCl_3$) δ 8.58 (s, 1 H, NH), 7.44-7.28 (m, 2 H, Ar-H), 7.28-6.94 (m, 6 H, Ar-H), 3.50 (s, 3 H, CH_3), 2.33 (s, 3 H, CH_3), 1.81 (s, 6 H, CH_3 × 2); ^{13}C NMR (75 MHz, $CDCl_3$) δ 204.4, 167.5, 138.3, 137.1, 136.7, 132.3, 130.7, 130.3, 129.5, 128.7, 126.9, 125.6, 103.5, 97.7, 64.0, 20.9, 20.0; IR ν (neat, cm^{-1}) 3198, 3060, 2977, 2934, 2905, 2855, 2813, 1956, 1659, 1590, 1488, 1440, 1376, 1296, 1165, 1035; MS (EI, 70 eV) *m/z* (%) 307 (M^+, 69.68), 260 (100); HRMS Calcd for $C_{20}H_{21}NO_2$ (M^+): 307.1572. Found: 305.1575.

11. Preparation of **3ah**. (Wsz-5-12)

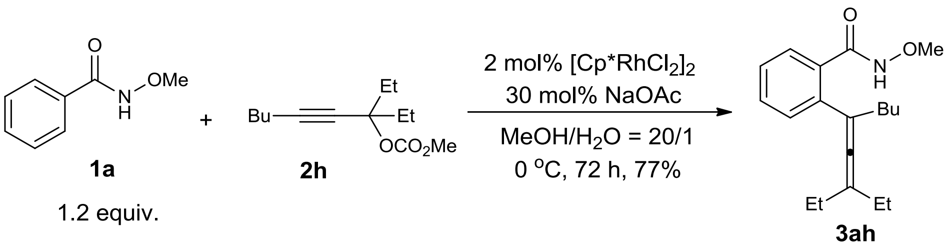

The reaction of **1a** (182.0 mg, 1.2 mmol), $[Cp^*RhCl_2]_2$ (12.6 mg, 0.02 mmol), NaOAc (24.9 mg, 0.3 mmol), **2h** (226.5 mg, 1 mmol), MeOH (6 mL), and H_2O (0.3 mL) at 0 ^oC afforded **3ah** (232.9 mg, 77%) (eluent: hexane/ethyl acetate/dichloromethane = 50/5/1): oil;

^1H NMR (300 MHz, $CDCl_3$) δ 9.19 (s, 1 H, NH), 7.61 (d, *J* = 7.5 Hz, 1 H, Ar-H), 7.38 (td, J_1 = 7.5 Hz, J_2 = 1.4 Hz, 1 H, Ar-H), 7.32-7.20 (m, 2 H, Ar-H), 3.83 (s, 3 H, OCH_3), 2.29 (t, *J* = 7.4 Hz, 2 H, CH_2), 2.13-1.97 (m, 4 H, CH_2 × 2), 1.49-1.22 (m, 4 H, CH_2 × 2), 1.09 (t, *J* = 7.4 Hz, 6 H, CH_3 × 2), 0.87 (t, *J* = 6.9 Hz, 3 H, CH_3); ^{13}C NMR (75 MHz, $CDCl_3$) δ 199.7, 167.4, 138.5, 130.9, 130.6, 129.7, 129.1, 126.8, 110.3, 107.8, 64.1, 34.2, 30.3, 25.8, 22.2, 13.8, 12.4; IR ν (neat, cm^{-1}) 3200, 3061, 2962, 2932, 2873, 1950, 1660, 1594, 1570, 1495, 1459, 1376, 1297, 1158, 1035; MS (EI, 70 eV) *m/z* (%) 301 (M^+, 29.23), 188 (100); HRMS Calcd for $C_{19}H_{27}NO_2$ (M^+): 301.2042. Found: 301.2038.

12. Preparation of **3bf**. (Wsz-6-84)

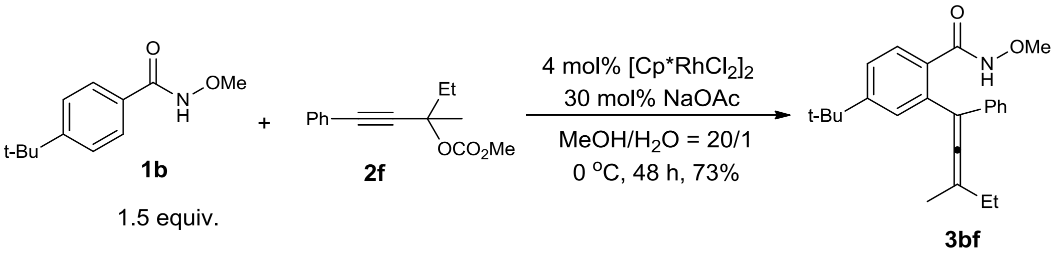

The reaction of **1b** (310.7 mg, 1.5 mmol), $[Cp^*RhCl_2]_2$ (24.8 mg, 0.04 mmol), NaOAc (25.3 mg, 0.3 mmol), **2f** (232.6 mg, 1 mmol), MeOH (6 mL), and H_2O (0.3 mL) at 0 °C afforded **3bf** (265.1 mg, 73%) (eluent: petroleum/ethyl acetate/dichloromethane = 4.5/1/1): oil; ^1H NMR (300 MHz, $CDCl_3$) δ 8.81 (s, 1 H, NH), 7.74 (d, *J* = 8.1 Hz, 1 H, Ar-H), 7.44 (dd, *J* = 8.3 Hz, *J* = 2.0 Hz, 1 H, Ar-H), 7.36 (d, *J* = 2.1 Hz, 1 H, Ar-H), 7.32-7.14 (m, 5 H, Ar-H), 3.41 (s, 3 H, OCH_3), 2.29-2.05 (m, 2 H, CH_2), 1.90 (s, 3 H, CH_3), 1.33 (s, 9 H, CH_3 × 3), 1.15 (t, *J* = 7.4 Hz, 3 H, CH_3); ^{13}C NMR (75 MHz, $CDCl_3$) δ 201.9, 166.4, 154.5, 137.2, 135.0, 129.4, 128.5, 128.2, 126.9, 126.4, 124.9, 108.1, 106.1, 63.7, 34.7, 31.0, 27.3, 18.7, 12.3; IR ν (neat, cm^{-1}) 3195, 3058, 3021, 2964, 2929, 2899, 2869, 1950, 1659, 1600, 1491,

1459, 1363, 1305, 1253, 1095, 1035; MS (EI, 70 eV) *m/z* (%) 363 (M^+, 1.35), 302 (100); HRMS calcd. for $C_{24}H_{29}NO_2$ (M^+): 363.2198; Found: 363.2191.

13. Preparation of **3cf**. (Wsz-6-83)

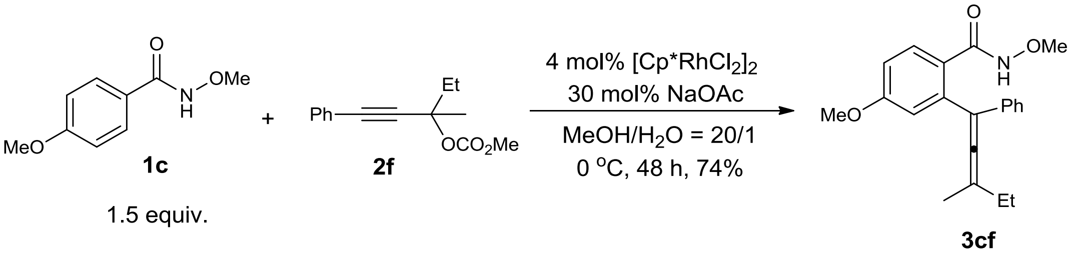

The reaction of **1c** (272.2 mg, 1.5 mmol), $[Cp^*RhCl_2]_2$ (24.4 mg, 0.04 mmol), NaOAc (24.5 mg, 0.3 mmol), **2f** (233.3 mg, 1 mmol), MeOH (6 mL), and H_2O (0.3 mL) at 0 °C afforded **3cf** (251.8 mg, 74%) (eluent: petroleum/ethyl acetate/dichloromethane = 3.5/1/1): oil; ^{1}H NMR (300 MHz, $CDCl_3$) δ 8.79 (brs, 1 H, NH), 7.81 (d, J = 8.4 Hz, 1 H, Ar-H), 7.33-7.24 (m, 2 H, Ar-H), 7.24-7.15 (m, 3 H, Ar-H), 6.94 (dd, J_1 = 8.4 Hz, J_2 = 2.7 Hz, 1 H, Ar-H), 6.85 (d, J = 2.7 Hz, 1 H, Ar-H), 3.84 (s, 3 H, OCH_3), 3.42 (s, 3 H, OCH_3), 2.27-2.06 (m, 2 H, CH_2), 1.90 (s, 3 H, CH_3), 1.14 (t, J = 7.4 Hz, 3 H, CH_3); ^{13}C NMR (75 MHz, $CDCl_3$) δ 201.6, 166.1, 161.5, 137.2, 136.9, 131.7, 128.6, 127.1, 126.4, 124.7, 116.6, 113.0, 107.8, 106.6, 63.8, 55.3, 27.4, 18.7, 12.4; IR ν (neat, cm^{-1}) 3201, 3078, 3057, 2965, 2934, 2839, 1944, 1659, 1599, 1568, 1491, 1458, 1418, 1368, 1316, 1287, 1224, 1109, 1028; MS (EI, 70 eV) *m/z* (%) 337 (M^+, 1.82), 276 (100); HRMS Calcd for $C_{21}H_{23}NO_3$ (M^+): 337.1678. Found: 337.1682.

14. Preparation of **3fa**. (Wsz-4-43)

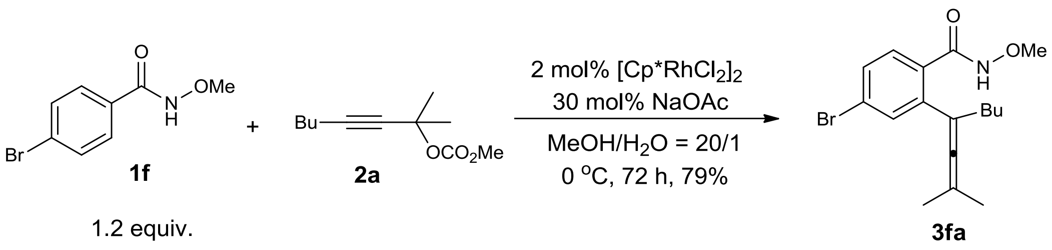

The reaction of **1f** (276.6 mg, 1.2 mmol), $[Cp^*RhCl_2]_2$ (13.0 mg, 0.02 mmol), NaOAc (24.3 mg, 0.3 mmol), **2a** (198.8 mg, 1 mmol), MeOH (6 mL), and H_2O (0.3 mL) at 0 ^oC afforded **3fa** (278.6 mg, 79%) (eluent: petroleum/ethyl acetate/dichloromethane = 15/1/0.1 to 8/1/0.1): solid; m.p. 91.8-92.2 ^oC (hexane/ethyl acetate); ^1H NMR (300 MHz, $CDCl_3$) δ 8.81 (brs, 1 H, NH), 7.39 (bs, 3 H, Ar-H), 3.84 (s, 3 H, OCH_3), 2.26 (t, J = 6.9 Hz, 2 H, CH_2), 1.76 (s, 6 H, 2 × CH_3), 1.50-1.27 (m, 4 H, 2 × CH_2), 0.90 (t, J = 6.9 Hz, 3 H, CH_3); ^{13}C NMR (75 MHz, $CDCl_3$) δ 201.5, 167.0, 140.4, 131.8, 130.6, 130.4, 129.8, 124.9, 102.0, 98.6, 64.4, 33.3, 30.0, 22.2, 20.3, 13.9; IR ν (neat, cm^{-1}) 3184, 2957, 2932, 2872, 2859, 1956, 1659, 1581, 1556, 1466, 1376, 1361, 1300, 1245, 1082, 1039; MS (EI, 70 eV) m/z (%) 353 ($M^+(Br^{81})$, 4.51), 351 ($M^+(Br^{79})$, 5.27), 43 (100); Anal. Calcd for $C_{17}H_{22}BrNO_2$: C 57.96, H 6.29, N 3.98. Found: C 58.27, H 6.33, N 3.80.

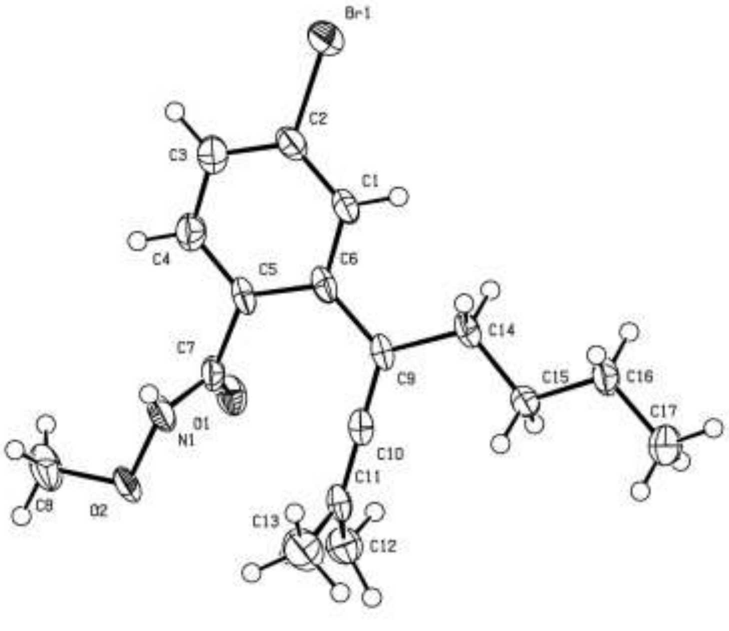

3fa: $C_{17}H_{22}BrNO_2$, MW = 352.27, triclinic, space group P -1, final R indices [I > 2σ(I)], R1 = 0.0882, wR2 = 0.2330; Rindices (all data), R1 = 0.1201, wR2 = 0.2562; a = 8.5427(8) Å, b = 12.5727(13) Å, c = 16.5276(13) Å, α = 82.936(7)°, β = 80.423(7)°, γ = 77.308(8)°, V = 1700.7(3) $Å^3$, T = 293(2) K, Z = 4, reflections collected/unique 10652/6217 (R_{int} = 0.0521), number of observations [> 2σ(I)]: 4293, parameters: 387. Supplementary crystallographic data have been deposited at the Cambridge Crystallographic Data Centre, CCDC 1030064.

15. Preparation of **3ff**. (Wsz-6-85)

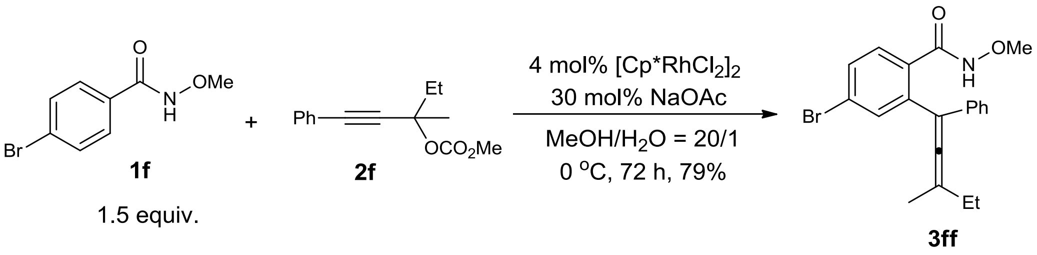

The reaction of **1f** (344.6 mg, 1.5 mmol), $[Cp^*RhCl_2]_2$ (24.9 mg, 0.04 mmol), NaOAc (24.3 mg, 0.3 mmol), **2f** (232.8 mg, 1 mmol), MeOH (6 mL), and H_2O (0.3 mL) at 0 °C afforded **3ff** (305.2 mg, 79%) (eluent: petroleum/ethyl acetate/dichloromethane = 5/1/1): solid; m.p. 134.6-135.8 °C (hexane/ethyl acetate); ^1H NMR (300 MHz, $CDCl_3$) δ 8.67 (brs, 1 H, NH), 7.63 (d, J = 8.7 Hz, 1 H, Ar-H), 7.59-7.46 (m, 2 H, Ar-H), 7.36-7.11 (m, 5 H, Ar-H), 3.43 (s, 3 H, OCH_3), 2.23-2.09 (m, 2 H, CH_2), 1.90 (s, 3 H, CH_3), 1.13 (t, J = 7.4 Hz, 3 H, CH_3); ^{13}C NMR (75 MHz, $CDCl_3$) δ 202.1, 165.6, 137.6, 136.6, 133.9, 131.4, 131.2, 131.0, 128.7, 127.3, 126.4, 125.4, 107.2, 106.6, 63.9, 27.4, 18.6, 12.4; IR ν (neat, cm^{-1}) 3185, 3081, 3054, 2967, 2935, 1944, 1655, 1580, 1556, 1492, 1456, 1307, 1263, 1156, 1080, 1035; MS (EI, 70 eV) m/z (%) 387 ($M^+(Br^{81})$, 3.27), 385 ($M^+(Br^{79})$, 2.63), 324 (100); Anal. Calcd for $C_{20}H_{20}BrNO_2$: C 62.19, H 5.22, N 3.63. Found: C 62.17, H 5.10, N 3.54.

16. Preparation of **3ga**. (Wsz-4-39)

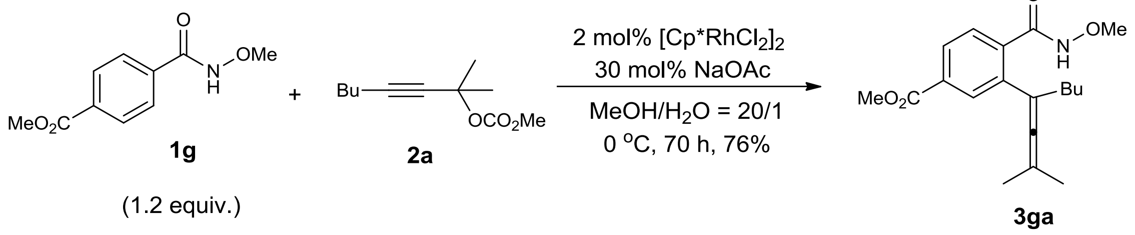

The reaction of **1g** (252.1 mg, 1.2 mmol), $[Cp^*RhCl_2]_2$ (12.6 mg, 0.02 mmol), NaOAc (24.3 mg, 0.3 mmol), **2a** (197.3 mg, 1 mmol), MeOH (6 mL), and H_2O (0.3 mL) at 0 ^{o}C afforded **3ga** (250.2 mg, 76%) (eluent: petroleum/ethyl acetate/dichloromethane = 10/1/0.1 to 5/1/0.5): oil; ^{1}H NMR (300 MHz, $CDCl_3$) δ 8.82 (brs, 1 H, NH), 7.98-7.84 (m, 2 H, Ar-H), 7.56 (d, J = 7.2 Hz, 1 H, Ar-H), 3.93 (s, 3 H, OCH_3), 3.86 (s, 3 H, OCH_3), 2.32 (t, J = 6.6 Hz, 2 H, CH_2), 1.76 (s, 6 H, 2 × CH_3), 1.50-1.28 (m, 4 H, 2 × CH_2), 0.90 (t, J = 6.9 Hz, 3 H, CH_3); ^{13}C NMR (75 MHz, $CDCl_3$) δ 201.6, 167.1, 166.3, 138.7, 135.8, 131.7, 129.9, 129.2, 127.6, 101.9, 98.5, 64.4, 52.3, 33.2, 30.0, 22.2, 20.3, 20.1, 13.9; IR ν (neat, cm^{-1}) 3209, 2955, 2934, 2871, 1953, 1727, 1663, 1495, 1437, 1362, 1294, 1247, 1112, 1051; MS (EI, 70 eV) *m*/*z* (%) 332 (M^++1, 1.77), 331 (M^+, 4.01), 300 (100); HRMS Calcd for $C_{19}H_{25}NO_4$ (M^+): 331.1784. Found: 331.1786.

17. Preparation of **3hi**. (Wsz-4-114)

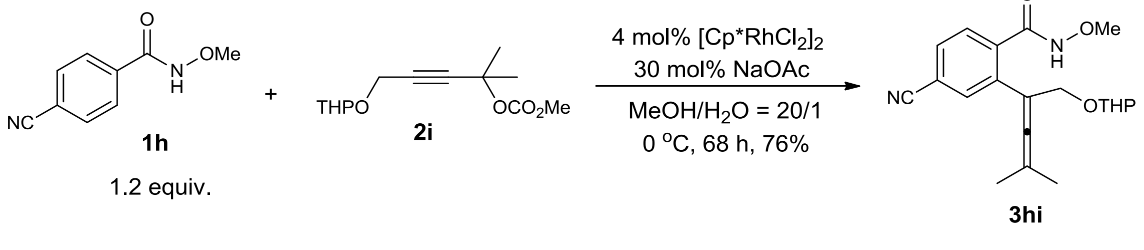

The reaction of **1h** (210.8 mg, 1.2 mmol), $[Cp^*RhCl_2]_2$ (24.8 mg, 0.04 mmol), NaOAc

(25.1 mg, 0.3 mmol), **2i** (256.6 mg, 1 mmol), MeOH (6 mL), and H_2O (0.3 mL) at 0 ^oC afforded **3hi** (271.8 mg, 76%) (eluent: hexane/ethyl acetate/dichloromethane = 5/1/0.5 to dichloromethane/ethyl acetate = 1/1): oil; ^1H NMR (300 MHz, $CDCl_3$) δ 10.33 (s, 1 H, NH), 7.74 (d, *J* = 8.1 Hz, 1 H, Ar-H), 7.69-7.58 (m, 2 H, Ar-H), 4.71 (s, 1 H, CH), 4.55 (d, *J* = 10.8 Hz, 1 H, one proton of CH_2), 4.39 (d, *J* = 10.8 Hz, 1 H, one proton of CH_2), 3.84 (s, 3 H, OCH_3), 3.60-3.39 (m, 2 H, CH_2), 1.80-1.40 (m, 12 H, CH_2 × 3 and CH_3 × 2); ^{13}C NMR (75 MHz, $CDCl_3$) δ 203.0, 166.2, 137.9, 137.1, 131.5, 130.5, 130.2, 117.8, 113.8, 99.0, 98.8, 96.2, 70.4, 64.2, 61.9, 30.1, 24.8, 19.8, 19.6, 18.8; IR ν (neat, cm^{-1}) 3222, 2941, 2869, 2231, 1956, 1667, 1504, 1456, 1441, 1363, 1285, 1262, 1201, 1183, 1119, 1074, 1040, 1022; MS (EI, 70 eV) *m/z* (%) 356 (M^+, 3.60), 84 (100); HRMS Calcd for $C_{20}H_{24}N_2O_4$ (M^+): 356.1736. Found: 356.1740.

18. Preparation of **3ij**. (Wsz-5-6)

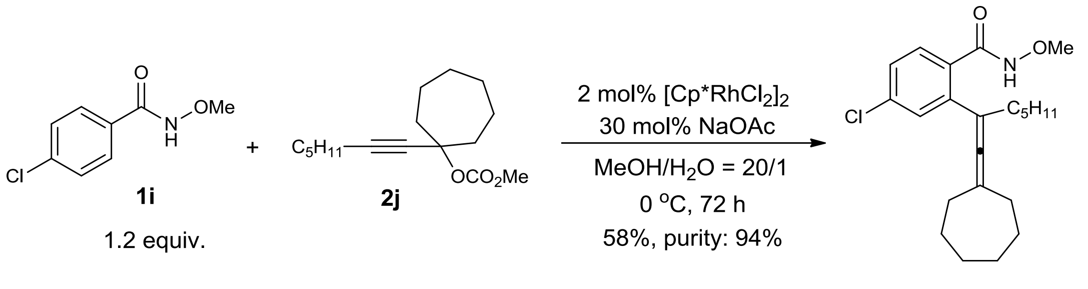

The reaction of **1i** (223.1 mg, 1.2 mmol), $[Cp^*RhCl_2]_2$ (12.6 mg, 0.02 mmol), NaOAc (24.1 mg, 0.3 mmol), **2j** (266.0 mg, 1 mmol), MeOH (6 mL), and H_2O (0.3 mL) at 0 ^oC afforded **3ij** (232.5 mg, 58%, purity: 94%) (eluent: hexane/ethyl acetate/dichloromethane = 10/1/0.5): oil; ^1H NMR (300 MHz, $CDCl_3$) δ 8.88 (brs, 1 H, NH), 7.52 (d, *J* = 6.6 Hz, 1 H, Ar-H), 7.30-7.17 (m, 2 H, Ar-H), 3.84 (s, 3 H, CH_3), 2.45-2.18 (m, 6 H, CH_2 × 3), 1.77-1.15

(m, 14 H, CH_2 × 7), 0.88 (t, *J* = 6.5 Hz, 3 H, CH_3); ^{13}C NMR (75 MHz, $CDCl_3$) δ 201.4, 166.9, 140.4, 136.5, 130.7, 129.7, 129.2, 126.9, 107.7, 102.1, 64.4, 33.7, 32.1, 31.3, 29.3, 28.3, 27.7, 22.5, 14.0; IR *ν* (neat, cm^{-1}) 3184, 2926, 2849, 1941, 1659, 1586, 1559, 1466, 1441, 1302, 1089, 1039; MS (EI, 70 eV) *m/z* (%) 377 (M(^{37}Cl)$^+$, 3.83), 375 (M(^{35}Cl)$^+$, 11.62), 123 (100); HRMS Calcd for $C_{22}H_{30}NO_2{}^{35}Cl$ (M(^{35}Cl)$^+$): 375.1965. Found: 375.1961.

19. Preparation of **3al**. (Wsz-6-3)

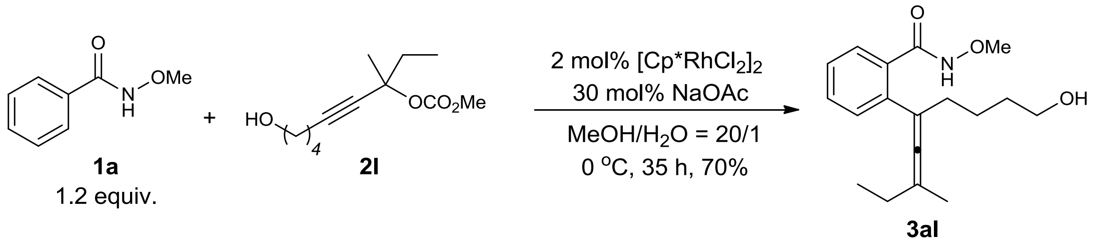

The reaction of **1a** (181.6 mg, 1.2 mmol), $[Cp^*RhCl_2]_2$ (12.5 mg, 0.02 mmol), NaOAc (24.1 mg, 0.3 mmol), **2l** (228.0 mg, 1 mmol), MeOH (6 mL), and H_2O (0.3 mL) at 0 °C afforded **3al** (212.3 mg, 70%) (eluent: petroleum/ethyl acetate/dichloromethane = 3/1/0.3 to dichloromethane/ethyl acetate = 3/1): oil; ^{1}H NMR (300 MHz, $CDCl_3$) δ 9.32 (s, 1 H, NH), 7.48 (d, *J* = 7.2 Hz, 1 H, Ar-H), 7.41-7.32 (m, 1 H, Ar-H), 7.29-7.18 (m, 2 H, Ar-H), 3.80 (s, 3 H, OCH_3), 3.53 (t, *J* = 5.7 Hz, 2 H, OCH_2), 2.69 (brs, 1 H, OH), 2.31 (t, *J* = 7.1 Hz, 2 H, CH_2), 2.10-1.90 (m, 2 H, CH_2), 1.76 (s, 3 H, CH_3), 1.62-1.38 (m, 4 H, CH_2 × 2), 1.02 (t, *J* = 7.4 Hz, 3 H, CH_3); ^{13}C NMR (75 MHz, $CDCl_3$) δ 200.5, 167.8, 138.3, 131.5, 130.4, 129.1, 128.8, 126.6, 104.5, 103.6, 64.1, 62.1, 33.6, 31.7, 27.1, 24.0, 18.6, 12.2; IR *ν* (neat, cm^{-1}) 3345, 3194, 3060, 2965, 2934, 1947, 1663, 1593, 1478, 1456, 1440, 1304, 1153, 1055, 1035; MS (EI, 70 eV) *m/z* (%) 303 (M$^+$, 1.49), 57 (100); HRMS Calcd for $C_{18}H_{25}NO_3$ (M$^+$): 303.1834. Found: 303.1829.

20. Preparation of **3am**. (Wsz-6-27, Hx-13-132)

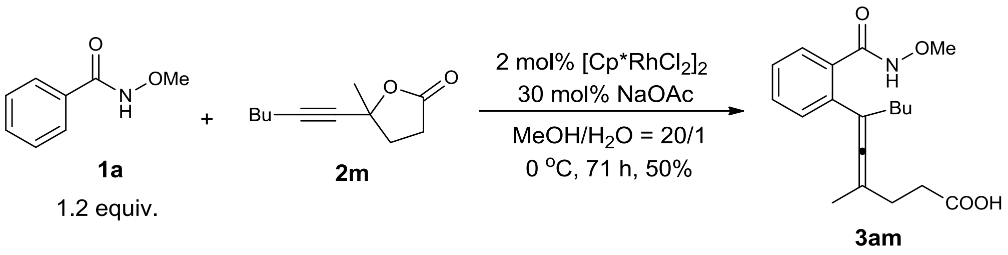

The reaction of **1a** (181.4 mg, 1.2 mmol), $[Cp^*RhCl_2]_2$ (12.5 mg, 0.02 mmol), NaOAc (24.8 mg, 0.3 mmol), **2m** (180.3 mg, 1 mmol), MeOH (6 mL), and H_2O (0.3 mL) at 0 ^oC afforded **3am** (164.0 mg, 50%) (eluent: dichloromethane/ diethyl ether = 10/1 to 5/1 to 2/1 for the first round to get one part of pure **3am** and one part of not pure **3am**, which was further purified by using chromatography: dichloromethane/ diethyl ether = 5/1): oil; ^1H NMR (300 MHz, $CDCl_3$) δ 9.42 (brs, 1 H, NH), 8.61 (bs, 1 H, COOH), 7.50-7.31 (m, 2 H, Ar-H), 7.26-7.15 (m, 2 H, Ar-H), 3.81 (s, 3 H, OCH_3), 2.55-2.15 (m, 6 H, 3 × CH_2), 1.81 (s, 3 H, CH_3), 1.46-1.22 (m, 4 H, 2 × CH_2), 0.87 (t, J = 6.8 Hz, 3 H, CH_3); ^{13}C NMR (75 MHz, $CDCl_3$) δ 200.6, 178.2, 168.2, 137.8, 131.4, 130.4, 129.0, 128.8, 126.6, 106.5, 101.0, 64.1, 33.1, 32.2, 30.1, 28.8, 22.1, 18.9, 13.8; IR ν (neat, cm^{-1}) 3189, 2956, 2932, 2875, 2852, 1953, 1709, 1654, 1635, 1462, 1440, 1301, 1257, 1153, 1034; MS (EI, 70 eV) m/z (%) 332 (M^++1, 2.88), 331 (M^+, 5.69), 300 (100); HRMS Calcd for $C_{19}H_{25}NO_4$ (M^+): 331.1784. Found: 331.1782.

21. Preparation of **3ja**. (Wsz-4-96)

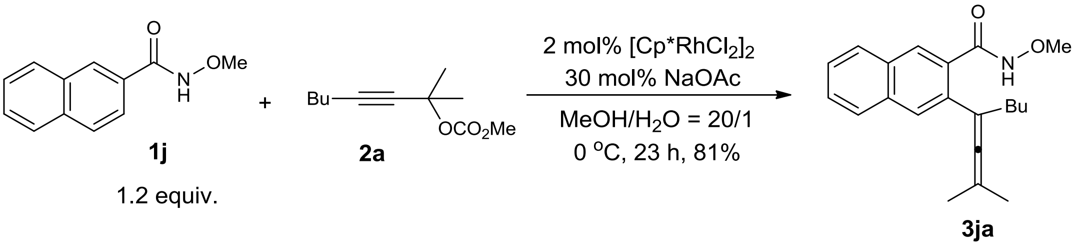

The reaction of **1j** (242.5 mg, 1.2 mmol), $[Cp^*RhCl_2]_2$ (12.7 mg, 0.02 mmol), NaOAc (24.5 mg, 0.3 mmol), **2a** (199.2 mg, 1 mmol), MeOH (6 mL), and H_2O (0.3 mL) at 0 ^{o}C afforded **3ja** (263.6 mg, 81%) (eluent: hexane/ ethyl acetate/dichloromethane = 10/1/0.5): solid; m.p. 101.6-103.3 ^{o}C (hexane/ethyl acetate); ^{1}H NMR (300 MHz, $CDCl_3$) δ 8.92 (s, 1 H, NH), 8.06 (s, 1 H, Ar-H), 7.80 (dd, J_1 = 7.5 Hz, J_2 = 4.8 Hz, 2 H, Ar-H), 7.68 (s, 1 H, Ar-H), 7.55-7.40 (m, 2 H, Ar-H), 3.88 (s, 3 H, OCH_3), 2.39 (t, J = 7.1 Hz, 2 H, CH_2), 1.79 (s, 6 H, CH_3 × 2), 1.52-1.30 (m, 4 H, CH_2 × 2), 0.91 (t, J = 7.2 Hz, 3 H, CH_3); ^{13}C NMR (75 MHz, $CDCl_3$) δ 201.8, 167.9, 134.9, 134.1, 131.4, 130.1, 129.7, 128.2, 127.6, 127.5, 126.4, 102.9, 97.8, 64.4, 34.5, 30.1, 22.2, 20.4, 13.9; IR ν (neat, cm^{-1}) 3193, 3055, 2956, 2931, 2870, 2855, 1956, 1660, 1590, 1492, 1440, 1376, 1361, 1293, 1188, 1147, 1101, 1034; MS (EI, 70 eV) *m/z* (%) 323 (M^+, 29.23), 292 (100); Anal. Calcd for $C_{21}H_{25}NO_2$: C 77.98, H 7.79, N 4.33. Found: C 77.64, H 7.75, N 4.31.

22. Preparation of **3ka**. (Wsz-4-118)

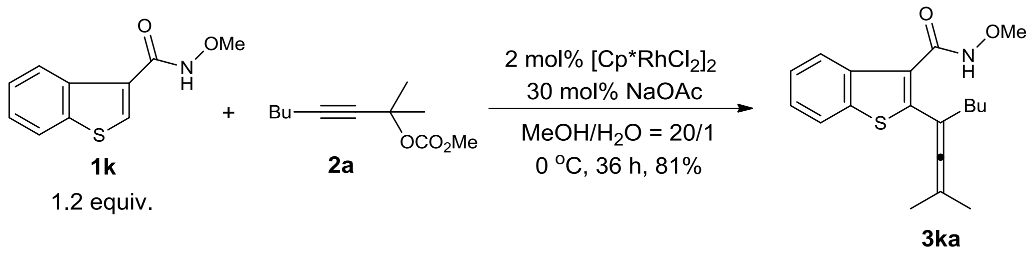

The reaction of **1k** (248.3 mg, 1.2 mmol), $[Cp^*RhCl_2]_2$ (12.6 mg, 0.02 mmol), NaOAc (24.9 mg, 0.3 mmol), **2a** (199.8 mg, 1 mmol), MeOH (6 mL), and H_2O (0.3 mL) at 0 ^{o}C afforded **3ka** (268.7 mg, 81%) (eluent: dichloromethane/ethyl acetate/triethylamine = 50/1/0.5): solid; m.p. 87.4-89.0 ^{o}C (hexane/ethyl acetate); ^{1}H NMR (300 MHz, $CDCl_3$) δ 8.61(s, 1 H, NH), 7.87 (d, J = 7.5 Hz, 1 H, Ar-H), 7.71 (d, J = 7.5 Hz, 1 H, Ar-H), 7.40-7.25

(m, 2 H, Ar-H), 3.90 (s, 3 H, OCH_3), 2.40 (t, J = 7.4 Hz, 2 H, CH_2), 1.80 (s, 6 H, CH_3 × 2), 1.55-1.30 (m, 4 H, CH_2 × 2), 0.92 (t, J = 7.2 Hz, 3 H, CH_3); ^{13}C NMR (75 MHz, $CDCl_3$) δ 202.7, 163.8, 144.8, 138.8, 137.9, 124.9, 124.6, 123.7, 122.6, 121.6, 99.6, 97.8, 64.6, 33.1, 30.2, 22.1, 20.2, 13.9; IR ν (neat, cm^{-1}) 3177, 3060, 2956, 2932, 2872, 2852, 1947, 1651, 1529, 1458, 1435, 1377, 1361, 1275, 1247, 1182, 1085, 1063, 1013; MS (EI, 70 eV) m/z (%) 329 (M^+, 2.17), 69 (100); Anal. Calcd for $C_{19}H_{23}NO_2S$: C 69.27, H 7.04, N 4.25. Found: C 69.26, H 7.06, N 4.13.

23. Preparation of **3la** and **4la**. (Wsz-4-146)

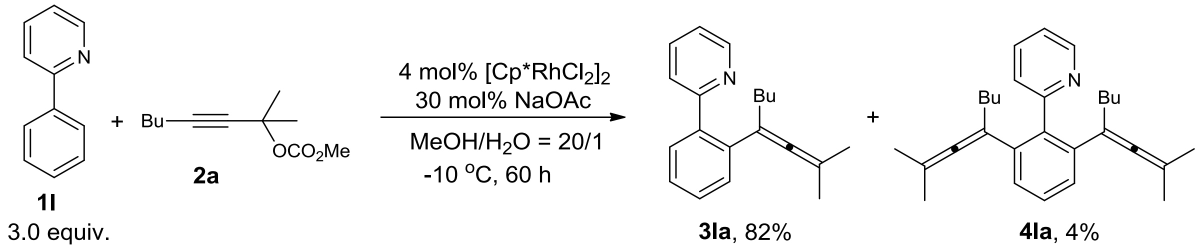

The reaction of **1l** (2327.6 mg, 15.0 mmol), $[Cp^*RhCl_2]_2$ (123.5 mg, 0.2 mmol), NaOAc (124.1 mg, 1.5 mmol), **2a** (992.3 mg, 5 mmol), MeOH (30 mL), and H_2O (1.5 mL) at -10 °C afforded **3la** (1142.6 mg, 82%) and **4la** (79.0 mg, 4%) (eluent: petroleum/ethyl acetate = 100/1).

3la: oil; ^1H NMR (300 MHz, $CDCl_3$) δ 8.70-8.66 (m, 1 H, Ar-H), 7.66 (td, J_1 = 7.8 Hz, J_2 = 1.8 Hz, 1 H, Ar-H), 7.53-7.46 (m, 2 H, Ar-H), 7.36-7.26 (m, 3 H, Ar-H), 7.23-7.17 (m, 1 H, Ar-H), 2.03 (t, J = 7.2 Hz, 2 H, CH_2), 1.46 (s, 6 H, CH_3 × 2), 1.36-1.16 (m, 4 H, CH_2 × 2), 0.81 (t, J = 7.1 Hz, 3 H, CH_3); ^{13}C NMR (75 MHz, $CDCl_3$) δ 202.1, 160.0, 149.2, 139.2, 138.9, 135.4, 130.1, 129.2, 128.0, 126.6, 124.3, 121.3, 103.3, 95.8, 33.3, 29.9, 22.1, 20.0, 13.9; IR ν (neat, cm^{-1}) 3061, 2956, 2929, 2857, 1950, 1584, 1569, 1557, 1462, 1423, 1361,

1293, 1147, 1022; MS (EI, 70 eV) *m/z* (%) 277 (M^+, 26.74), 234 (100); HRMS Calcd for $C_{20}H_{23}N$ (M^+): 277.1830. Found: 277.1833.

4la: oil; ^{1}H NMR (300 MHz, $CDCl_3$) δ 8.64-8.57 (m, 1 H, Ar-H), 7.62 (td, J_1 = 7.7 Hz, J_2 = 1.8 Hz, 1 H, Ar-H), 7.29-7.23 (m, 2 H, Ar-H), 7.21-7.12 (m, 3 H, Ar-H), 1.96 (t, *J* = 7.1 Hz, 4 H, CH_2 × 2), 1.39 (s, 12 H, CH_3 × 4), 1.30-1.12 (m, 8 H, CH_2 × 4), 0.80 (t, *J* = 7.2 Hz, 6 H, CH_3 × 2); ^{13}C NMR (75 MHz, $CDCl_3$) δ 201.3, 160.1, 148.6, 140.0, 138.2, 134.8, 127.5, 127.4, 125.9, 121.1, 103.3, 95.6, 34.0, 29.8, 22.1, 20.4, 14.0; IR *ν* (neat, cm^{-1}) 3058, 2956, 2929, 2857, 1962, 1588, 1571, 1561, 1450, 1419, 1376, 1361, 1261, 1185, 1094, 1023; MS (EI, 70 eV) *m/z* (%) 399 (M^+, 2.72), 315 (100); HRMS Calcd for $C_{29}H_{37}N$ (M^+): 399.2926. Found: 399.2925.

24. Preparation of **3ma**. (Wsz-4-147)

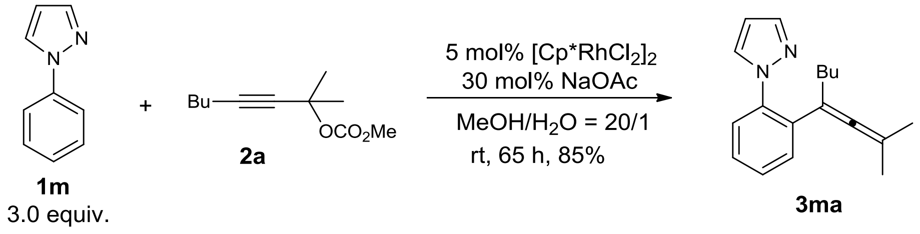

The reaction of **1m** (432.5 mg, 3.0 mmol), $[Cp^*RhCl_2]_2$ (30.8 mg, 0.05 mmol), NaOAc (25.2 mg, 0.3 mmol), **2a** (197.6 mg, 1 mmol), MeOH (6 mL), and H_2O (0.3 mL) at rt afforded **3ma** (224.9 mg, 85%) (eluent: petroleum/ethyl acetate = 100/1): oil; ^{1}H NMR (300 MHz, $CDCl_3$) δ 7.73 (d, *J* = 2.4 Hz, 1 H, Ar-H), 7.68 (d, *J* = 1.5 Hz, 1 H, Ar-H), 7.50-7.43 (m, 1 H, Ar-H), 7.36-7.27 (m, 3 H, Ar-H), 6.40 (t, *J* = 2.1 Hz, 1 H, Ar-H), 1.75 (t, *J* = 7.1 Hz, 2 H, CH_2), 1.67 (s, 6 H, CH_3 × 2), 1.33-1.12 (m, 4 H, CH_2 × 2), 0.80 (t, *J* = 7.1 Hz, 3 H, CH_3); ^{13}C NMR (75 MHz, $CDCl_3$) δ 201.7, 140.1, 138.2, 134.9, 130.8, 130.7, 127.7, 127.5, 126.2,

106.4, 102.0, 96.3, 32.1, 30.0, 22.0, 20.4, 13.8; IR ν (neat, cm^{-1}) 3101, 3060, 2956, 2930, 2872, 2858, 1959, 1599, 1572, 1518, 1489, 1460, 1394, 1362, 1330, 1191, 1096, 1043, 1021; MS (EI, 70 eV) *m*/*z* (%) 266 (M^+, 6.62), 223 (100); HRMS Calcd for $C_{18}H_{22}N_2$ (M^+): 266.1783. Found: 266.1785.

Synthetic application of the products

1. Preparation of **5**. (Wsz-5-133)

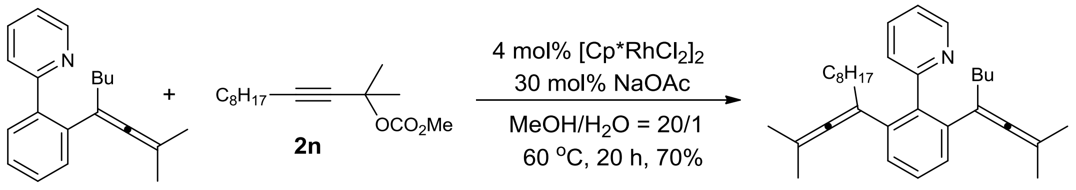

3la
2.0 equiv

5

To a dried Schlenk tube equipped with a Teflon-coated magnetic stirring bar were added $[Cp^*RhCl_2]_2$ (12.5 mg, 0.02 mmol), NaOAc (12.8 mg, 0.15 mmol), **3la** (277.5 mg, 1.0 mmol), **2n** (126.8 mg, 0.5 mmol), MeOH (3 mL), and H_2O (0.15 mL) sequentially at rt. The Schlenk tube was then equipped with a condenser. After being stirred for 20 h at 60 °C, the reaction was complete as monitored by TLC (eluent: petroleum ether/ethyl acetate = 20/1). Filtration through a short column of silica gel (eluent: ethyl acetate (20 mL × 3) and evaporation afforded the crude product, which was purified by flash column chromatography on silica gel (eluent: hexane/ethyl acetate = 100/1) to afford **5** (158.4 mg, 70%): oil; ^{1}H NMR (300 MHz, $CDCl_3$) δ 8.61 (d, J = 4.2 Hz, 1 H, Ar-H), 7.62 (td, J_1 = 7.8 Hz, J_2 = 1.8 Hz, 1 H, Ar-H), 7.30-7.22 (m, 2 H, Ar-H), 7.21-7.11 (m, 3 H, Ar-H), 2.01-1.90 (m, 4 H, CH_2 × 2), 1.39 (s, 12 H, CH_3 × 4), 1.32-1.10 (m, 16 H, CH_2 × 8), 0.87 (t, J = 6.8 Hz, 3 H, CH_3), 0.80 (t, J = 7.1 Hz, 3 H, CH_3); ^{13}C NMR (75 MHz, $CDCl_3$) δ 201.2, 160.0, 148.6, 139.92, 139.89, 138.1, 134.9,

127.5, 127.4, 125.9, 121.1, 103.3, 103.2, 95.6, 34.2, 33.9, 31.8, 29.8, 29.5, 29.3, 29.0, 27.6, 22.6, 22.1, 20.4, 14.1, 14.0; IR ν (neat, cm^{-1}) 3059, 2955, 2926, 2854, 1962, 1932, 1588, 1571, 1561, 1452, 1419, 1377, 1361, 1188, 1023; MS (EI, 70 eV) *m/z* (%) 455 (M^+, 18.96), 84 (100); HRMS Calcd for $C_{33}H_{45}N$ (M^+): 455.3552. Found: 455.3553.

2. Preparation of **6**.[2] (Wsz-5-11)

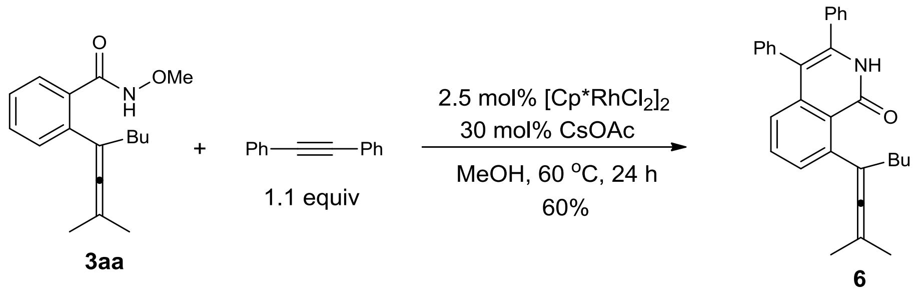

To a dried Schlenk tube equipped with a Teflon-coated magnetic stirring bar were added **3aa** (109.3 mg, 0.4 mmol), 1,2-diphenylethyne (78.6 mg, 0.44 mmol), $[Cp^*RhCl_2]_2$ (6.2 mg, 0.01 mmol), CsOAc (22.4 mg, 0.12 mmol), and MeOH (2 mL) sequentially at rt. The Schlenk tube was then equipped with a condenser. After being stirred for 24 h at 60 ^oC, the reaction was complete as monitored by TLC (eluent: petroleum ether/ethyl acetate = 3/1). Filtration through a short column of silica gel (eluent: (dichloromethane/ethyl acetate = 1/1) (20 mL × 3)) and evaporation afforded the crude product, which was purified by flash column chromatography on silica gel (eluent: dichloromethane/ethyl acetate = 20/1) to afford **6** (101.1 mg, 60%): solid; m.p. 199.0-200.4 ^oC (hexane/ethyl acetate); ^1H NMR (300 MHz, $CDCl_3$) δ 9.02 (s, 1 H, Ar-H), 7.44 (t, J = 7.8 Hz, 1 H, Ar-H), 7.34-7.10 (m, 12 H, Ar-H), 2.33 (t, J = 7.1 Hz, 2 H, CH_2), 1.76 (s, 6 H, CH_3 × 2), 1.53-1.29 (m, 4 H, CH_2 × 2), 0.90 (t, J = 7.1 Hz, 3 H, CH_3); ^{13}C NMR (75 MHz, $CDCl_3$) δ 199.0, 161.6, 142.7, 140.2, 137.2, 136.4, 135.1, 131.9,

131.7, 129.8, 129.0, 128.5, 128.3, 127.1, 124.9, 122.5, 116.8, 106.9, 95.6, 34.7, 30.5, 22.5, 20.9, 14.2; IR ν (neat, cm^{-1}) 3453, 3165, 3027, 2947, 2929, 2869, 2849, 1642, 1596, 1584, 1486, 1462, 1441, 1311, 1144; MS (EI, 70 eV) *m/z* (%) 419 (M^+, 31.3), 376 (100); HRMS Calcd for $C_{30}H_{29}NO$ (M^+): 419.2249. Found: 419.2247. Anal. Calcd for $C_{30}H_{29}NO$: C 85.88, H 6.97, N 3.34. Found: C 84.90, H 6.96, N 3.31.

Mechanistic studies

Steric effect

The formation of **7**. (Wsz-5-32)

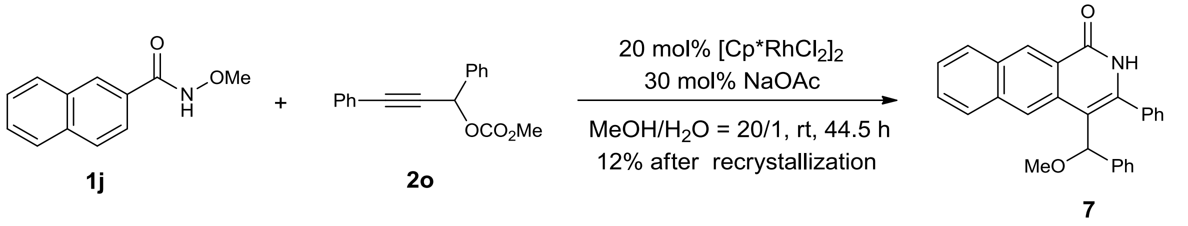

Following the typical procedure, the reaction of **1j** (303.1 mg, 1.5 mmol), $[Cp^*RhCl_2]_2$ (185.5 mg, 0.3 mmol), NaOAc (36.9 mg, 0.45 mmol), **2o** (400.1 mg, 1.5 mmol), MeOH (9 mL), and H_2O (0.45 mL) at rt afforded impure **7** (136.8 mg) (eluent: hexane/ethyl acetate/dichloromethane = 10/1/0.2 to 8/1/0.5), which was further purified by recrystallization (hexane/THF) afford pure **7** (69.7 mg): solid; m.p. 117.5-119.0 ^{o}C (hexane/THF); ^{1}H NMR (300 MHz, $CDCl_3$) δ 9.02 (s, 1 H, Ar-H), 8.91 (bs, 1 H, NH), 8.32 (s, 1 H, Ar-H), 8.06-7.94 (m, 1 H, Ar-H), 7.75-7.66 (m, 1 H, Ar-H), 7.55-7.40 (m, 8 H, Ar-H), 7.36-7.17 (m, 4 H, Ar-H), 5.57 (s, 1 H, CH), 3.31 (s, 3 H, CH_3); ^{13}C NMR (75 MHz, $CDCl_3$) δ 163.2, 141.6, 139.4, 135.1, 134.8, 131.2, 131.1, 129.6, 129.04, 128.97, 128.81, 128.77, 128.5, 128.3, 127.8, 127.1, 126.6, 126.2, 124.5, 111.1, 80.3, 56.5; IR ν (neat, cm^{-1}) 3181,

3057, 3027, 2927, 2890, 2819, 1962, 1659, 1625, 1600, 1493, 1448, 1355, 1312, 1089, 1022; MS (EI, 70 eV) *m/z* (%) 392 (M^++1, 30.89), 391 (M^+, 100); HRMS Calcd for $C_{27}H_{21}NO_2$ (M^+): 391.1572. Found: 391.1571.

The sample for X-ray diffraction study was obtained via column chromatographic separation (eluent: hexane/ethyl acetate/CH_2Cl_2 = 10:1:0.5) followed by recrystallizaion with hexane/ethyl acetate.

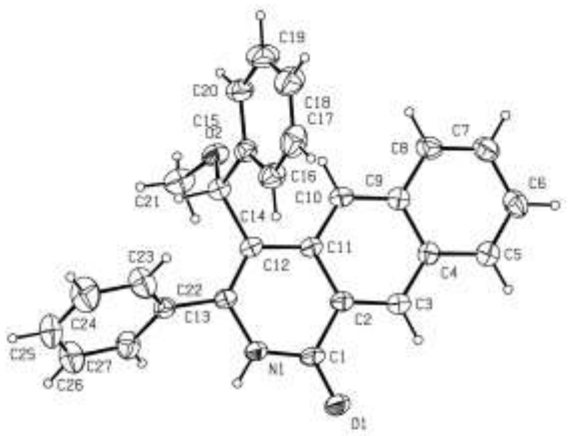

7: $C_{27}H_{21}NO_2$, MW = 391.45, monoclinic, space group P 1 21/c 1, final R indices [I > 2σ(I)], R1 = 0.0829, wR2 = 0.2230; Rindices (all data), R1 = 0.1776, wR2 = 0.2687; a = 16.9004(15) Å, b = 17.8357(15) Å, c = 18.4349(18) Å, α = 90.00°, β = 116.962(12)°, γ = 90.00°, V = 4952.8(8) $Å^3$, T = 293(2) K, Z = 8, reflections collected/unique 31620/9060 (R_{int} = 0.0792), number of observations [> 2σ(I)]: 3484, parameters: 543. Supplementary crystallographic data have been deposited at the Cambridge Crystallographic Data Centre, CCDC 1030128.

Highly entioselective synthesis of tetral-substituted allenes by chirality transfer

1. Preparation of (*S*)-**3af**. (Wsz-6-67)

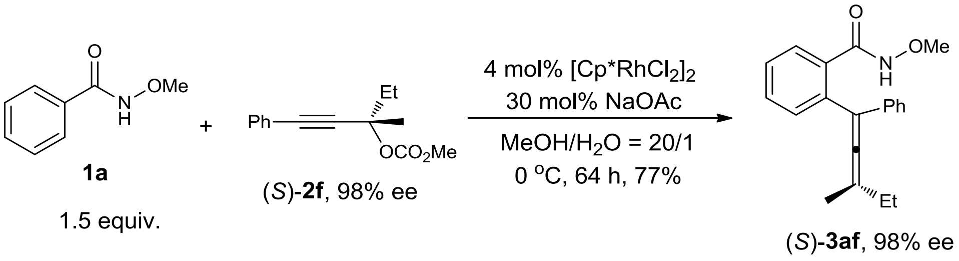

Following the **Typical Procedure**, the reaction of **1a** (45.6 mg, 0.3 mmol), $[Cp^*RhCl_2]_2$ (4.8 mg, 0.008 mmol), NaOAc (5.3 mg, 0.06 mmol), (*S*)-**2f** (98% ee, 45.9 mg, 0.2 mmol), MeOH (1.2 mL), and H_2O (0.06 mL) at 0 ^oC afforded (*S*)-**3af** (46.6 mg, 77%) (eluent: petroleum/ethyl acetate/dichloromethane = 5/1/0.5): 98% ee (HPLC conditions: Chiralcel AD-H column, hexane/*i*-PrOH = 10/1, 1.0 mL/min, λ = 207 nm, t_R(minor) = 21.9 min, t_R(major) = 24.5 min); solid; m.p. 99.9-101.1 ^oC (hexane/ethyl acetate); ^1H NMR (300 MHz, $CDCl_3$) δ 8.66 (s, 1 H, NH), 7.78 (d, *J* = 7.5 Hz, 1 H, Ar-H), 7.53-7.14 (m, 8 H, Ar-H), 3.45 (s, 3 H, OCH_3), 2.27-2.06 (m, 2 H, CH_2), 1.90 (s, 3 H, CH_3), 1.13 (t, *J* = 7.4 Hz, 3 H, CH_3); ^{13}C NMR (75 MHz, $CDCl_3$) δ 202.0, 166.6, 137.2, 135.6, 132.5, 131.20, 131.18, 129.6, 128.6, 127.9, 127.1, 126.5, 107.5, 106.5, 63.9, 27.4, 18.7, 12.3; IR ν (neat, cm^{-1}) 3199, 3058, 3018, 2966, 2932, 1947, 1659, 1595, 1491, 1456, 1439, 1310, 1159, 1031; MS (EI, 70 eV) *m*/*z* (%) 307 (M^+, 1.69), 246 (100); Anal. Calcd for $C_{20}H_{21}NO_2$: C 78.15, H 6.89, N 4.56. Found: C 77.98, H 6.88, N 4.34.

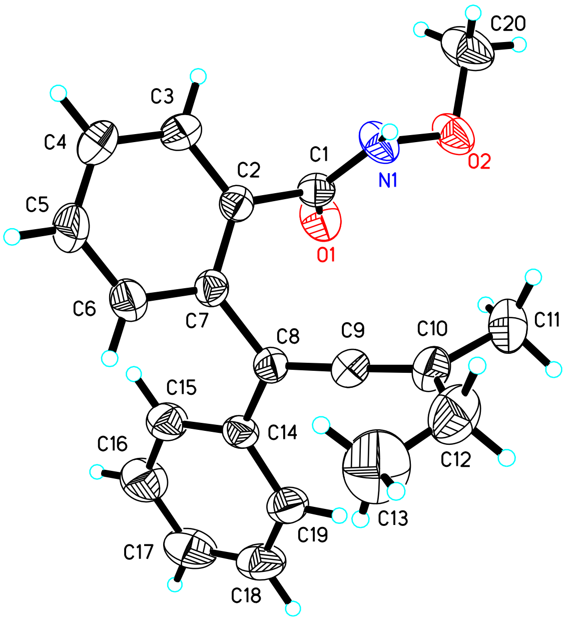

(*S*)-**3af**: $C_{20}H_{21}NO_2$, MW = 307.38, monoclinic, space group P 21, final R indices [I > 2σ(I)], R1 = 0.0350, wR2 = 0.1001; Rindices (all data), R1 = 0.0357, wR2 = 0.1012; a = 9.2428(10) Å, b = 8.1334(9) Å, c = 12.1422(13) Å, α = 90.00°, β = 97.677(7)°, γ = 90.00°, V = 904.61(17) $Å^3$, T = 296(2) K, Z = 2, reflections collected/unique 7061/2925 (R_{int} = 0.0192), number of observations [> 2σ(I)]: 2835, parameters: 216. Supplementary crystallographic data have been deposited at the Cambridge Crystallographic Data Centre, CCDC 1049557.

2. Preparation of (*S*)-**3bf**. (Wsz-6-87)

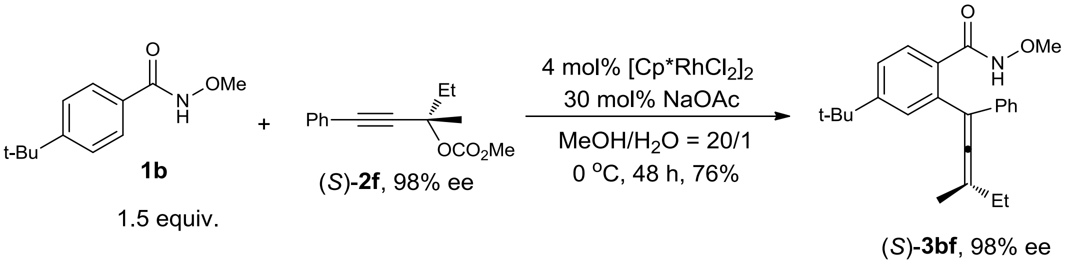

Following the **Typical Procedure**, the reaction of **1b** (62.9 mg, 0.3 mmol), $[Cp^*RhCl_2]_2$

(4.9 mg, 0.008 mmol), NaOAc (4.6 mg, 0.06 mmol), (*S*)-**2f** (98% ee, 45.8 mg, 0.2 mmol), MeOH (1.2 mL), and H_2O (0.06 mL) at 0 ^oC afforded (*S*)-**3bf** (54.4 mg, 76%) (eluent: petroleum/ethyl acetate/dichloromethane = 4.5/1/1): 98% ee (HPLC conditions: Chiralcel AD-H column, hexane/*i*-PrOH = 10/1, 0.8 mL/min, λ = 207 nm, t_R(minor) = 21.3 min, t_R(major) = 23.6 min); oil; ^1H NMR (300 MHz, $CDCl_3$) δ 8.70 (s, 1 H, NH), 7.77 (d, *J* = 8.1 Hz, 1 H, Ar-H), 7.46 (dd, *J* = 8.3 Hz, *J* = 2.0 Hz, 1 H, Ar-H), 7.35 (d, *J* = 2.1 Hz, 1 H, Ar-H), 7.33-7.16 (m, 5 H, Ar-H), 3.43 (s, 3 H, CH_3), 2.29-2.05 (m, 2 H, CH_2), 1.90 (s, 3 H, CH_3), 1.33 (s, 9 H, CH_3 × 3), 1.15 (t, *J* = 7.4 Hz, 3 H, CH_3); ^{13}C NMR (75 MHz, $CDCl_3$) δ 202.0, 166.5, 154.7, 137.3, 135.1, 129.6, 129.5, 128.7, 128.3, 127.1, 126.5, 125.1, 108.2, 106.3, 63.8, 34.8, 31.1, 27.5, 18.7, 12.4; IR *ν* (neat, cm^{-1}) 3195, 3058, 3021, 2964, 2932, 2902, 2872, 1944, 1660, 1600, 1492, 1460, 1395, 1364, 1305, 1254, 1095, 1035; MS (EI, 70 eV) *m*/*z* (%) 363 (M^+, 1.99), 331 (100); HRMS calcd. for $C_{24}H_{29}NO_2$ (M^+): 363.2198; Found: 363.2195.

3. Preparation of (*S*)-**3cf**. (Wsz-6-86)

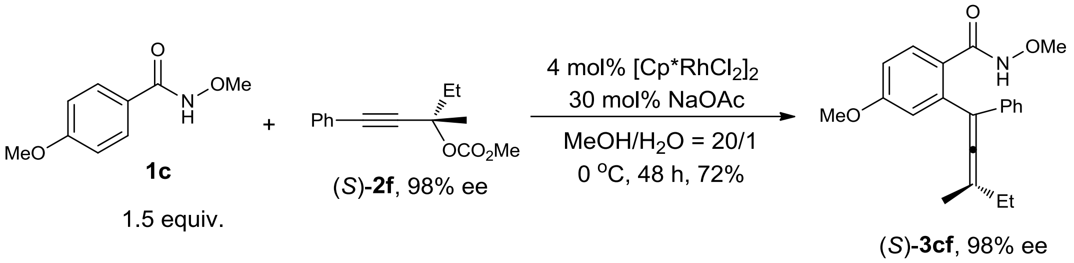

Following the **Typical Procedure**, the reaction of **1c** (54.2 mg, 0.3 mmol), $[Cp^*RhCl_2]_2$ (4.7 mg, 0.008 mmol), NaOAc (5.0 mg, 0.06 mmol), (*S*)-**2f** (98% ee, 46.1 mg, 0.2 mmol), MeOH (1.2 mL), and H_2O (0.06 mL) at 0 ^oC afforded (*S*)-**3cf** (48.4 mg, 72%) (eluent: petroleum/ethyl acetate/dichloromethane = 3.5/1/1): 98% ee (HPLC conditions: Chiralcel AD-H column, hexane/*i*-PrOH = 10/1, 1.0 mL/min, λ = 207 nm, t_R(minor) = 31.5 min,

t_R(major) = 35.4 min); oil; ^{1}H NMR (300 MHz, $CDCl_3$) δ 8.75 (s, 1 H, NH), 7.82 (d, *J* = 8.7 Hz, 1 H, Ar-H), 7.33-7.15 (m, 5 H, Ar-H), 6.94 (dd, J_1 = 8.6 Hz, J_2 = 2.6 Hz, 1 H, Ar-H), 6.85 (d, *J* = 2.7 Hz, 1 H, Ar-H), 3.84 (s, 3 H, OCH_3), 3.43 (s, 3 H, OCH_3), 2.28-2.06 (m, 2 H, CH_2), 1.90 (s, 3 H, CH_3), 1.14 (t, *J* = 7.4 Hz, 3 H, CH_3); ^{13}C NMR (75 MHz, $CDCl_3$) δ 201.7, 166.2, 161.6, 137.3, 136.9, 131.8, 128.7, 127.1, 126.4, 124.7, 116.6, 113.1, 107.9, 106.7, 63.8, 55.4, 27.4, 18.7, 12.4; IR *ν* (neat, cm^{-1}) 3203, 3081, 3057, 2965, 2934, 2839, 1947, 1660, 1599, 1568, 1490, 1462, 1368, 1287, 1224, 1182, 1150, 1109, 1028; MS (EI, 70 eV) *m/z* (%) 337 (M^+, 3.21), 276 (100); HRMS Calcd for $C_{21}H_{23}NO_3$ (M^+): 337.1678. Found: 337.1676.

4. Preparation of (*S*)-**3ff**. (Wsz-6-88)

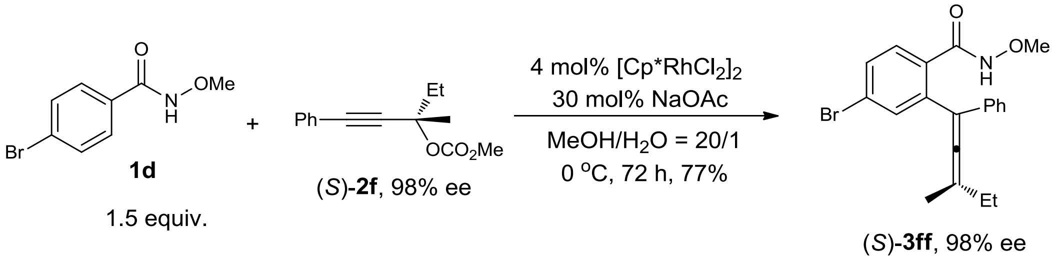

Following the **Typical Procedure**, the reaction of **1f** (68.8 mg, 0.3 mmol), $[Cp^*RhCl_2]_2$ (5.0 mg, 0.008 mmol), NaOAc (5.3 mg, 0.06 mmol), (*S*)-**2f** (98% ee, 46.7 mg, 0.2 mmol), MeOH (1.2 mL), and H_2O (0.06 mL) at 0 ^{o}C afforded (*S*)-**3ff** (59.8 mg, 77%) (eluent: petroleum/ethyl acetate/dichloromethane = 5/1/1): 98% ee (HPLC conditions: Chiralcel AD-H column, hexane/*i*-PrOH = 10/1, 0.7 mL/min, λ = 207 nm, t_R(minor) = 28.0 min, t_R(major) = 30.4 min); solid; m.p. 121.7-123.1 ^{o}C (hexane/ethyl acetate); ^{1}H NMR (300 MHz, $CDCl_3$) δ 8.68 (s, 1 H, NH), 7.63 (d, *J* = 8.1 Hz, 1 H, Ar-H), 7.54 (dd, J_1 = 8.4 Hz, J_2 = 1.8 Hz, 1 H, Ar-H), 7.49 (d, *J* = 1.8 Hz, 1 H, Ar-H), 7.34-7.13 (m, 5 H, Ar-H), 3.43 (s, 3 H, OCH_3), 2.26-2.08 (m, 2 H, CH_2), 1.90 (s, 3 H, CH_3), 1.13 (t, *J* = 7.4 Hz, 3 H, CH_3); ^{13}C NMR

(75 MHz, $CDCl_3$) δ 202.1, 165.6, 137.6, 136.7, 133.9, 131.4, 131.2, 131.0, 128.7, 127.3, 126.4, 125.4, 107.2, 106.6, 63.9, 27.4, 18.6, 12.3; IR ν (neat, cm^{-1}) 3187, 2966, 2933, 1944, 1659, 1580, 1556, 1492, 1456, 1364, 1301, 1159, 1080, 1035; MS (EI, 70 eV) *m/z* (%) 387 (M^+(Br^{81}), 1.16), 385 (M^+(Br^{79}), 1.39), 353 (100); Anal. Calcd for $C_{20}H_{20}BrNO_2$: C 62.19, H 5.22, N 3.63. Found: C 62.08, H 5.26, N 3.42.

Kinetic isotope effect (parallel experiment) (Wsz-5-115, Wsz-5-116)

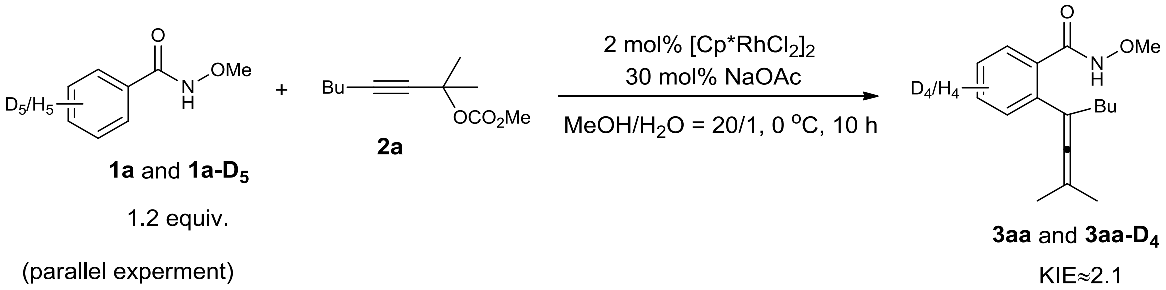

To a dried Schlenk tube equipped with a Teflon-coated magnetic stirring bar were added **1a** (91.2 mg, 0.6 mmol), [Cp*$RhCl_2$]$_2$ (6.5 mg, 0.01 mmol), NaOAc (12.6 mg, 0.15 mmol), **2a** (99.4 mg, 0.5 mmol), MeOH (3 mL), and H_2O (0.15 mL) sequentially at rt (Wsz-5-115). After being stirred for 10 h at 0 ^oC, the resulting mixture was mix with (Wsz-5-116).

In another dried Schlenk tube, the reaction of **1a-D$_5$** (93.7 mg, 0.6 mmol), [Cp*$RhCl_2$]$_2$ (6.5 mg, 0.01 mmol), NaOAc (12.0 mg, 0.15 mmol), **2a** (99.5 mg, 0.5 mmol), MeOH (3 mL), and H_2O (0.15 mL) sequentially at rt (Wsz-5-116). After being stirred for 10 h at 0 ^oC, the resulting mixture was mix with (Wsz-5-115).

The mixed resulting mixture was evaporated to afford the crude product, which was purified by flash column chromatography on silica gel (eluent: petroleum/ethyl

acetate/dichloromethane = 10/1/0.2) to afford a mixture of **3aa** and **3aa-D$_4$** (120.2 mg, 44%): solid; m.p. 68.1-68.9 ^{o}C (hexane/ethyl acetate); ^{1}H NMR (300 MHz, $CDCl_3$) δ 8.80 (brs, 1 H, NH), 3.85 (s, 3 H, OCH_3), 2.29 (t, *J* = 7.2 Hz, 2 H, CH_2), 1.76 (s, 6 H, 2 × CH_3), 1.49-1.28 (m, 4 H, 2 × CH_2), 0.89 (t, *J* = 7.2 Hz, 3 H, CH_3); the following signal is discernible for **3aa**: δ 7.58 (d, *J* = 7.2 Hz, 0.68 H, Ar-H), 7.39 (td, J_1 = 6.9 Hz, J_2 = 1.3 Hz, 0.69 H, Ar-H), 7.32-7.21 (m, 1.47 H, Ar-H); IR ν (neat, cm^{-1}) 3188, 3057, 2962, 2931, 2866, 2858, 1659, 1593, 1504, 1459, 1440, 1296, 1156, 1035; MS (EI, 70 eV) *m/z* (%) 277 ($M^+(D_4)$, 30.57), 273 (M^+, 42.18), 198 (100). HRMS Calcd for $C_{17}H_{19}D_4NO_2$ (M^+): 277.1980. Found: 277.1985. HRMS Calcd for $C_{17}H_{23}NO_2$ (M^+): 273.1729. Found: 273.1733.

Supplementary References:

1. Forrat, V. J., Prieto, O., Ramón, D. J. & Yus, M. *trans*-1-Sulfonylamino-2-isoborneolsulfonylaminocyclohexane derivatives: excellent chiral ligands for the catalytic cnantioselective addition of organozinc reagents to ketones. *Chem. Eur. J.*, **12**, 4431-4445 (2006).
2. Guimond, N., Gouliaras, C. & Fagnou, K. Rhodium(III)-catalyzed isoquinolone synthesis: the N-O bond as a handle for C-N bond formation and catalyst turnover. *J. Am. Chem. Soc.* **132**, 6908-6909 (2010).
